# Specific, targetable interactions with the microenvironment influence imatinib-resistant chronic myeloid leukemia

**DOI:** 10.1038/s41375-020-0866-1

**Published:** 2020-05-21

**Authors:** Rahul Kumar, Raquel S. Pereira, Costanza Zanetti, Valentina R. Minciacchi, Maximilian Merten, Melanie Meister, Julian Niemann, Marina S. Dietz, Nina Rüssel, Frank Schnütgen, Minori Tamai, Koshi Akahane, Takeshi Inukai, Thomas Oellerich, Hans Michael Kvasnicka, Heike Pfeifer, Franck E. Nicolini, Mike Heilemann, Richard A. Van Etten, Daniela S. Krause

**Affiliations:** 1 Georg-Speyer-Haus, Institute for Tumor Biology and Experimental Therapy, 60596 Frankfurt am Main, Germany; 2 Institute for Physical and Theoretical Chemistry, Goethe-University Frankfurt, Frankfurt am Main, Germany; 3 Department of Internal Medicine, Hematology/Oncology, Goethe University, Frankfurt am Main, Germany; 4 Frankfurt Cancer Institute (FCI), Goethe University, Frankfurt am Main, Germany; 5 Department of Pediatrics, School of Medicine, University of Yamanashi, Chuo, Japan; 6 German Cancer Consortium (DKTK), Heidelberg, Germany; 7 German Cancer Research Center (DKFZ), Heidelberg, Germany; 8 Senckenberg Institute of Pathology, Goethe University Frankfurt, 60590 Frankfurt am Main, Germany; 9 Department of Hematology and INSERM U 1052, CRCL, Centre Léon Bérard, 69373 Lyon Cedex, France; 10 Chao Family Comprehensive Cancer Center, University of California, Irvine, CA 92697 USA

**Keywords:** Preclinical research, Cancer microenvironment

## Abstract

Therapy resistance in leukemia may be due to cancer cell-intrinsic and/or -extrinsic mechanisms. Mutations within *BCR-ABL1*, the oncogene giving rise to chronic myeloid leukemia (CML), lead to resistance to tyrosine kinase inhibitors (TKI), and some are associated with clinically more aggressive disease and worse outcome. Using the retroviral transduction/transplantation model of CML and human cell lines we faithfully recapitulate accelerated disease course in TKI resistance. We show in various models, that murine and human imatinib-resistant leukemia cells positive for the oncogene *BCR-ABL1*^*T315I*^ differ from *BCR-ABL1* native (*BCR-ABL1*) cells with regards to niche location and specific niche interactions. We implicate a pathway via integrin β3, integrin-linked kinase (ILK) and its role in deposition of the extracellular matrix (ECM) protein fibronectin as causative of these differences. We demonstrate a trend towards a reduced BCR-ABL1^T315I+^ tumor burden and significantly prolonged survival of mice with BCR-ABL1^T315I+^ CML treated with fibronectin or an ILK inhibitor in xenogeneic and syngeneic murine transplantation models, respectively. These data suggest that interactions with ECM proteins via the integrin β3/ILK-mediated signaling pathway in BCR-ABL1^T315I+^ cells differentially and specifically influence leukemia progression. Niche targeting via modulation of the ECM may be a feasible therapeutic approach to consider in this setting.

## Introduction

Resistance to cancer treatments may be due to cancer cell-intrinsic mechanisms, such as alteration of the drug target, deregulation of (anti-) apoptotic pathways, drug inactivation or shuttling of the drug out of the cancer cell [1–3]. Cancer cell-extrinsic mechanisms of chemoresistance may be mediated by alterations of drug binding to plasma proteins or their catabolism or the tumor microenvironment [4]. However, whether leukemia cell-intrinsic mutations conferring resistance to therapy lead to differential interactions with the bone marrow (BM) microenvironment (BMM), a complex arrangement of various cell types, extracellular matrix (ECM) proteins, and other factors [5], has not been shown.

The *BCR-ABL1* oncogene, generated by the translocation between chromosomes 9 and 22, results in deregulated activity of the BCR-ABL1 tyrosine kinase driving chronic myeloid leukemia (CML) at early disease stages [6]. BCR-ABL1 is targeted by tyrosine kinase inhibitors (TKIs) [7]. However, resistance to TKIs such as imatinib may occur due to the *BCR-ABL1*^*T315I*^ and other mutations [8, 9]. *BCR-ABL1*^*T315I*^ arises in the ABL kinase domain interfering with binding to TKIs like imatinib and others [8, 9] accounting for 15–20% of mutations found in CML patients [10, 11]. Patients with imatinib resistance due to BCR-ABL1^T315I^ have a rapid clinical course and poor prognosis [11–13], although other mechanisms may be contributory [14]. In the case of BCR-ABL1^T315I^ kinase activity does not correlate with increased transformation potency [15, 16]. Global phosphoproteome analysis of BCR-ABL1^T315I+^ cells identified a unique signature of phosphosubstrates compared with cells positive for native BCR-ABL1 or other imatinib-resistance conferring mutations leading to altered biological properties [15]. However, exactly how these leukemia cell-intrinsic alterations might influence disease outcome, for instance via altered interactions with the BMM, has not been demonstrated.

Niche occupation in the BMM by normal hematopoietic stem and progenitor cells (HSPC) [17] or leukemic stem cells (LSC) in acute myeloid leukemia (AML) [18] depends on their maturation stage or the state of disease progression, respectively. We hypothesized that differences in clinical outcome of patients with CML due to imatinib-resistant mutations in *BCR-ABL1* may correlate with LSC location in the BMM and specific interactions with the BMM. Indeed, here we show that interactions of leukemic murine and human cells with the BMM via the fibronectin/integrin β3/integrin-linked kinase (ILK)-mediated signaling pathway influence leukemia progression and clinical outcome in BCR-ABL1^T315I+^ imatinib-resistant CML in vivo. Targeting these interactions may offer a beneficial, innovative, additive treatment strategy for patients with BCR-ABL1^T315I+^ CML, and possibly other leukemias.

## Materials and methods

### Statistical analysis

All statistical analyses were performed using GraphPad Prism software. Survival curves were analyzed by Kaplan–Meier-style curves and Log-rank (Mantel–Cox) or Gehan–Breslow–Wilcoxon tests. Differences between groups were assessed by student’s *t*-test. When multiple hypotheses were tested, one-way ANOVA and a Tukey test were used as post-hoc test. The data were presented as mean ± s.d. *P* values ≤ 0.05 were considered significant.

## Results

### BCR-ABL1^T315I+^ differ from BCR-ABL1^+^ cells with regards to diverse biological functions

**Fig. 1:**
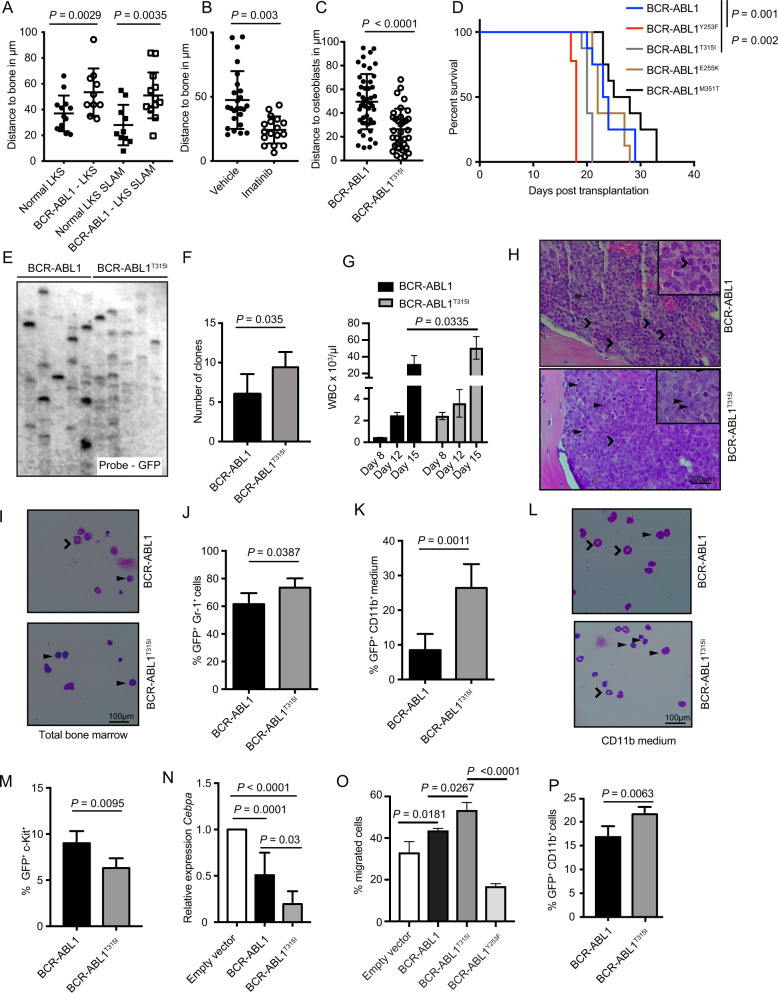
BCR-ABL1^T315I+^ differ from BCR-ABL1^+^ cells with regards to diverse biological functions. **a**–**c** Measurement of the shortest three-dimensional distance of (**a**) normal (black circles) or BCR-ABL1^+^ LKS (open circles) (*P* = 0.0029, *t*-test) or LKS CD150^+^ CD48^−^ (LKS SLAM) (black (normal) or open (BCR-ABL1^+^) squares) (*P* = 0.0035, *t*-test) cells or (**b**) BCR-ABL1^+^ LKS in vitro treated with vehicle (black circles) or 10 μM imatinib [56] for 4 h (open circles) (*P* = 0.003, *t*-test) to bone and (**c**) BCR-ABL1^+^ (black circles) versus BCR-ABL1^T315I+^ (open circles) LKS cells to osteoblasts (*P* < 0.0001, *t*-test) in μm. Hematopoietic cells were labeled with the lipophilic dye DiD and injected into unirradiated Col2.3 kb GFP mice. Imaging was performed 2 h after injection. The horizontal black line represents the mean. Each symbol represents a distinct cell from three separate experiments. **d** Kaplan–Meier-style survival curve of untreated BALB/c recipient mice transplanted with 2.5 × 10^5^ BCR-ABL1-(blue), BCR-ABL1^Y253F^-(red), BCR-ABL1^T315I^-(gray), BCR-ABL1^E255K^-(brown), or BCR-ABL1^M351T^-(black) transduced bone marrow. The difference in survival between BCR-ABL1^+^ and BCR-ABL1^T315I+^ (*P* = 0.002, Log-rank test) or BCR-ABL1^Y253F+^ (*P* = 0.001, Log-rank test) CML is significant (*n* = 8-9). **e**, **f** Southern blot showing distinct proviral integration events (**e**) and disease clonality (**f**) in spleens of BALB/c recipients of BCR-ABL1-(lanes 1–5) or BCR-ABL1^T315I^-(lanes 6–10) transduced bone marrow at the time of death (*P* = 0.035, *t*-test). **g** Leukocyte counts (WBC) × 10^3^ per μl in the peripheral blood of BALB/c recipient mice transplanted with BCR-ABL1-(black) or BCR-ABL1^T315I^-(gray) transduced bone marrow on days 8, 12, and 15 after transplantation (*P* = 0.0335; ANOVA, Tukey test, *n* = 4–5). **h** Hematoxylin and eosin stain of bone sections of mice with BCR-ABL1^+^ (top) or BCR-ABL1^T315I+^ (bottom) CML. The open arrows are pointing toward mature myeloid cells, while the closed arrows are pointing toward blasts. The scale bar depicts 200 μm (*n* = 5). **i** Giemsa stain of the cytospins of total bone marrow of representative BALB/c recipient mice transplanted with BCR-ABL1- or BCR-ABL1^T315I^-transduced bone marrow on day 15 after transplantation. A total of 50,000 bone marrow cells had been plated. The open arrows are pointing towards mature myeloid cells, while the closed arrows are pointing towards blasts. The scale bar depicts 100 μm (*n* = 5). **j** Percentage of GFP^+^ (BCR-ABL1^+^) Gr-1^+^ myeloid cells in peripheral blood of mice with BCR-ABL1^+^ (black) or BCR-ABL1^T315I+^ (gray) CML on day 15 after transplantation (*P* = 0.0387; *t*-test, *n* = 4–6). **k** Percentage of GFP^+^ (BCR-ABL1^+^) CD11b^medium+^ myeloid cells in peripheral blood of mice with BCR-ABL1^+^ (black) or BCR-ABL1^T315I+^ (gray) CML 15 days after transplantation (*P* = 0.0011; *t*-test, *n* = 4–6). **l** Giemsa stain of the cytospins of sorted CD11b medium^+^ bone marrow cells from representative BALB/c recipient mice transplanted with BCR-ABL1- or BCR-ABL1^T315I^-transduced bone marrow on day 15 after transplantation. A total of 10,000 CD11b medium^+^ bone marrow cells had been plated. The open arrows are pointing toward mature myeloid cells, while the closed arrows are pointing towards blasts. The scale bar depicts 100 μm (*n* = 3). **m** Percentage of GFP^+^ (BCR-ABL1^+^) c-Kit^+^ cells in the bone marrow of mice with BCR-ABL1^+^ (black) or BCR-ABL1^T315I+^ (gray) CML (*P* = 0.0095; *t*-test, *n* = 4–6) on day 15 after transplantation. **n** Relative expression of *Cebpa* in total bone marrow of murine recipients of empty vector (white)-, BCR-ABL1^+^ (black)-, or BCR-ABL1^T315I+^ (dark gray)-donor bone marrow 15 days after transplantation (*P* = 0.03; ANOVA, Tukey test, *n* = 5). **o** Percentage of empty vector (white)-, BCR-ABL1^+^ (black)-, BCR-ABL1^T315I+^ (dark gray)- or BCR-ABL1^Y253F+^ (light gray)-BA/F3 cells which migrated to the bottom chamber containing MS-5 stroma cells in medium containing 10% serum in a transwell migration assay after 8 h (*P* = 0.0267; ANOVA, Tukey test, *n* = 3). 10^5^ cells had been plated. **p** Percentage of BCR-ABL1^+^ (black) or BCR-ABL1^T315I+^ (gray) (GFP^+^) myeloid CD11b^+^ cells adhering to MS-5 stroma cells in vitro (*P* = 0.0063; *t*-test, *n* = 3). 1.5 × 10^5^ cells had been plated and allowed to adhere for 72 h. The data are representative of three independent experiments.

To test the location of CML-initiating cells (LIC) in the BMM, we performed in vivo confocal 2-photon microscopy of the murine calvarium. Measuring the shortest three-dimensional distance to the endosteum [17], we demonstrated that transplanted BCR-ABL1^+^ Lin^−^ c-Kit^+^ Sca-1^+^ (LKS) cells, which harbor the LSC [19] in the retroviral transduction/transplantation model of CML [20], and, particularly, LKS CD150^+^ CD48^−^ (SLAM) cells, were located significantly further away from the endosteum than control cells (*P* = 0.0029 and *P* = 0.0035, respectively, Figs. 1a, S1A and Supplementary Table 1). Prior in vitro treatment of BCR-ABL1^+^ LKS cells with imatinib led to closer localization of imatinib-treated LKS cells to the endosteum (*P* = 0.003, Figs. 1b and S1B). However, BCR-ABL1^T315I+^ LIC, which are resistant to all TKIs apart from ponatinib and the allosteric inhibitor of the ABL1 kinase, asciminib [21], localized closer to osteoblastic cells than BCR-ABL1^+^ LKS cells (*P* < 0.0001, Fig. 1c). We tested whether murine recipients of BM transduced with BCR-ABL1, BCR-ABL1^T315I^, BCR-ABL1^M351T^, BCR-ABL1^Y253F^, or BCR-ABL1^E255K^ (the latter both P-loop mutations) [20, 22] may recapitulate the accelerated disease course in patients. Indeed, the survival of untreated recipients of BCR-ABL1^T315I^− or BCR-ABL1^Y253F^− transduced BM was significantly shortened compared with recipients of BCR-ABL1^+^ or BCR-ABL1^M351T+^ BM (Fig. 1d). Consistent with increased engraftment of BCR-ABL1^T315I+^ proviral clones and more aggressive disease of BCR-ABL1^T315I+^ CML [20, 23], the disease clonality was significantly higher in recipients of BCR-ABL1^T315I+^ than BCR-ABL1^+^ BM (*P* = 0.035, Fig. 1e, f). CML was detectable 8 days after transplantation (Fig. S1C–D), and the leukocyte count was higher in recipients of BCR-ABL1^T315I+^ than BCR-ABL1^+^ BM (*P* = 0.0335, Fig. 1g). Similar to human imatinib-resistant patients with BCR-ABL1^T315I+^ disease, blasts were increased in the peripheral blood (Fig. S1E) and the BM of mice with BCR-ABL1^T315I+^ CML (Fig. 1h, i). GFP^+^ (BCR-ABL1^+^ or BCR-ABL1^T315I+^) Gr-1^+^ (Fig. 1j) and in particular GFP^+^ CD11b^medium+^ (Figs. 1k and S1F) myeloid cells, which likely represent the blasts (Fig. 1l), were higher in mice with BCR-ABL1^T315I+^ than BCR-ABL1^+^ CML. BCR-ABL1^T315I+^ CD11b^medium+^ cells expressed the highest levels of c-Kit (*P* < 0.0001, Fig. S1G), but lower levels of myeloperoxidase (*P* = 0.0121, Fig. S1H–I). Overall, a decrease of the percentage of GFP^+^ c-Kit^+^ cells (*P* = 0.0095, Fig. 1m) and an increase of GFP^+^ CD34^+^ (*P* = 0.0003, Fig. S2A) and GFP^+^ CD13^+^ (*P* = 0.0019, Fig. S2B) cells were observed in mice with BCR-ABL1^T315I+^ CML. The frequency of BCR-ABL1^T315I+^ (GFP^+^) LKS cells (*P* = 0.0045, Fig. S2C) and myeloid progenitor cells (Fig. S2D) in the BM were reduced. However, the percentage of BCR-ABL1^T315I+^ (GFP^+^) LKS SLAM did not differ compared with BCR-ABL1^+^ CML (Fig. S2E). Concordant with decreased myeloid maturation expression of the myeloid transcription factor *Cebpa* in total BM cells of mice with BCR-ABL1^T315I+^ CML was significantly decreased (*P* = 0.03, Fig. 1n and Supplementary Table 2). There was a trend toward reduced expression of *Spi1* (PU.1), another myeloid transcription factor, in BCR-ABL1^T315I+^ cells (Fig. S2F). The migration of BCR-ABL1^T315I+^ BA/F3 cells (*P* = 0.0267, Fig. 1o), a frequently used in vitro model [24, 25], and the adhesion of BCR-ABL1^T315I+^ CD11b^+^ cells to the stroma cell line MS-5 in vitro (*P* = 0.0063, Fig. 1p) were significantly increased compared with BCR-ABL1^+^ BA/F3 cells. In summary, these findings suggest that BCR-ABL1^+^ and BCR-ABL1^T315I+^ leukemia cells differ with respect to homing localization in the BMM, migration, adhesion, disease aggressiveness and (immuno-) phenotype.

### The actin cytoskeleton and expression and function of focal adhesion kinase differ between BCR-ABL1^T315I+^ and BCR-ABL1^+^ cells

**Fig. 2:**
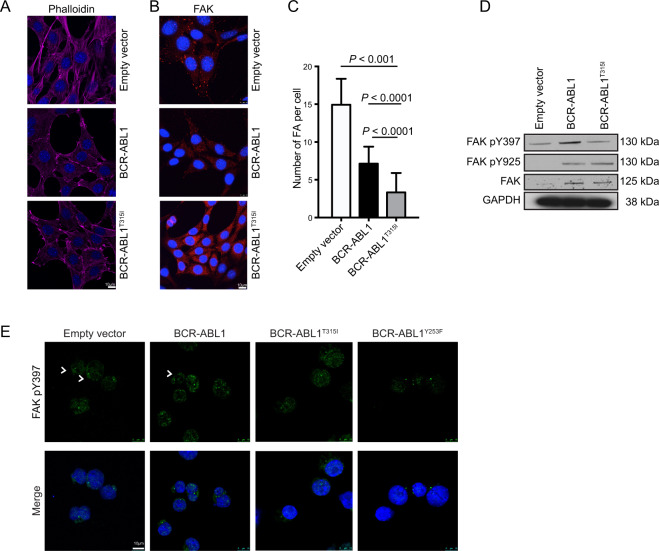
The actin cytoskeleton and expression of focal adhesion kinase differ between BCR-ABL1^T315I+^ and BCR-ABL1^+^ cells. Immunofluorescence studies on 3T3 fibroblasts transduced with empty vector, BCR-ABL1 or BCR-ABL1^T315I^ grown on coverslips and stained with phalloidin (**a**) or an antibody to FAK (**b**). **c** Quantification of the focal adhesions (FA) per 3T3 fibroblast transduced with empty vector (white), BCR-ABL1 (black), or BCR-ABL1^T315I^ (gray) from (b) (*P* < 0.0001; ANOVA, Tukey test, *n* = 3). The data in (**a–c**) are from three independent experiments. **d** Immunoblot showing the expression of FAKpY397 (130 kDa), FAKpY925 (130 kDa), FAK (125 kDa), or glyceraldehyde 3-phosphate dehydrogenase (GAPDH) (38 kDa) in lysates of BA/F3 cells transduced with empty vector, BCR-ABL1 or BCR-ABL1^T315I^. The immunoblot is representative of three experiments. **e** Immunofluorescence studies of BA/F3 cells transduced with empty vector-, BCR-ABL1-, BCR-ABL1^T315I^-, or BCR-ABL1^Y253F^-expressing retrovirus^,^ stained with an antibody to FAKpY397 and 4′,6-diamidino-2-phenylindole (DAPI). The data are representative of two experiments.

We hypothesized that differences in the actin cytoskeleton and/or focal adhesion kinase (FAK) [26], which is phosphorylated by BCR-ABL1 [27], underlie the increased migration (Fig. 1o) and adhesion (Fig. 1p) of BCR-ABL1^T315I+^ cells. Indeed, immunofluorescence staining of 3T3 fibroblasts, frequently used as model system to visualize the actin cytoskeleton in BCR-ABL1^+^ cells [28, 29], revealed that the evenly distributed and polymerized actin cytoskeleton in empty vector-transduced 3T3 cells was less finely arranged in BCR-ABL1^+^ and decreased in BCR-ABL1^T315+^ 3T3 cells (Figs. 2a and S3A). Staining of FAK, a focal adhesion protein involved in actin polymerization [30] and cytoskeletal stability [31], which lies downstream of the integrin receptors, in 3T3 cells transduced with empty vector-expressing retrovirus, was punctate, possibly consistent with an intact focal adhesosome. However, the punctae were reduced in BCR-ABL1^+^ and completely dispersed and granular in BCR-ABL1^T315I+^ 3T3 cells (*P* < 0.0001; Fig. 2b, c). Phosphorylation at phosphotyrosine pY397 in FAK, an autophosphorylation site [32], was higher in BCR-ABL1^+^ compared with BCR-ABL1^T315I+^ BA/F3 (Figs. 2d, e and S3B) and Lin^−^ cells (Fig. S3C), while phosphorylation at pY925 was similar. shRNA-mediated knockdown of *Ptk2* (FAK) (Fig. S3D) or the gene of another adapter protein at focal adhesion sites, paxillin (*Pxn*), (Fig. S3E) in BCR-ABL1^+^ or BCR-ABL1^T315I+^ donor BM did not lead to survival prolongation in recipient wildtype mice. Taken together, these data suggest that the cytoskeleton differs between BCR-ABL1^+^ versus BCR-ABL1^T315I+^ cells. Possibly due to differing phosphorylation of FAK by BCR-ABL1 versus BCR-ABL1^T315I^ focal adhesions may be dysfunctional in BCR-ABL1^T315I+^ cells.

### Integrin β3 expression on BCR-ABL1^T315I+^ cells influences CML progression

**Fig. 3:**
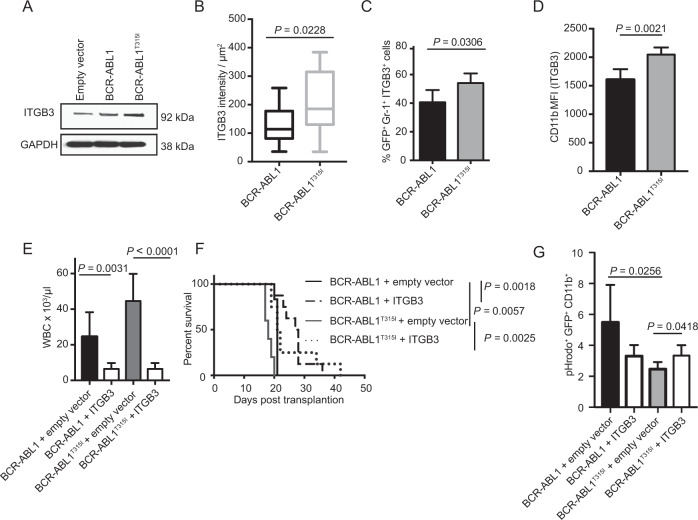
Integrin β3 is involved in progression of BCR-ABL1^T315I+^ CML. **a** Immunoblot showing the expression of integrin β3 (ITGB3) (92 kDa) or GAPDH (38 kDa) in lysates of BA/F3 cells transduced with empty vector, BCR-ABL1 or BCR-ABL1^T315I^. The immunoblot is representative of three experiments. **b** Results of super-resolution microscopy (dSTORM) for immunolabeled integrin β3 showing enhanced expression of the epitope on the surface of BA/F3 cells transduced with BCR-ABL1^T315I^ versus BCR-ABL1 (*P* = 0.0228; *t*-test, *n* = 3). The data are representative of three experiments. **c** Percentage of GFP^+^ (BCR-ABL1^+^) Gr-1^+^ integrin β3^+^ myeloid cells in peripheral blood of mice with BCR-ABL1^+^ (black) or BCR-ABL1^T315I+^ (gray) CML on day 15 after transplantation (*P* = 0.0306; *t*-test, *n* = 4–6). **d** Mean fluorescence intensity (MFI) of integrin β3 on CD11b^+^ cells from bone marrow of mice with BCR-ABL1^+^ (black) or BCR-ABL1^T315I+^ (gray) CML on day 15 after transplantation (*n* = 5). **e**, **f** Leukocyte counts (WBC) × 10^3^ per μl in peripheral blood (*P* < 0.0001 for BCR-ABL1^T315I+^; *t*-test, *n* = 7-8) (**e**) and Kaplan–Meier-style survival curve (**f**) of BALB/c recipient mice transplanted with bone marrow cotransduced with BCR-ABL1- or BCR-ABL1^T315I^-expressing retrovirus and integrin β3 (ITGB3)-overexpressing retrovirus (*P* = 0.0025 for BCR-ABL1^T315I^, Log-rank test). **g** Percentage of BCR-ABL1^+^ or BCR-ABL1^T315I+^ CD11b^+^ myeloid cells cotransduced with empty vector or integrin β3 (GFP^+^) from mice with established disease after incubation for 90 min with pHrodo-phycoerythrin (PE)-labeled *Escherichia coli* particles (*P* = 0.0256; *t*-test, *n* = 5).

Given our above observations, the known involvement of pY397 of FAK for FAK-mediated cell migration and the phosphorylation of FAK at pY397 upon clustering of integrins [32], we assessed expression of various integrins. While there was no difference in mean fluorescence intensity of integrin α5 (CD49e) (Fig. S4A), integrin α IIb (CD41), which forms a heterodimer with integrin β3 [33], was less expressed on BCR-ABL1^T315I+^ compared with BCR-ABL1^+^ cells (*P* = 0.0103, Fig. S4B). Further, integrin β3, previously implicated in the development and progression of AML [34], was more highly expressed on BCR-ABL1^T315I+^ versus BCR-ABL1^+^ or empty vector-transduced BA/F3 cells by immunoblotting (Figs. 3a and S4C) and super-resolution microscopy [35] (*P* = 0.0228, Figs. 3b and S4D). Primary Gr-1^+^ BCR-ABL1^T315I+^ myeloid cells (*P* = 0.0306, Fig. 3c) and total GFP^+^ BM cells (*P* = 0.0021, Fig. 3d) from CML mice also revealed significantly increased expression of integrin β3. This was also independently confirmed using stable isotope labeling with amino acids in cell culture-based quantitative mass spectrometry (Fig. S4E, Supplementary Table 3). Consistently, coimmunoprecipitation of BA/F3 cells transduced with BCR-ABL1 or BCR-ABL1^T315I^ with an antibody to integrin β3 revealed increased binding of FAK to integrin β3 in BCR-ABL1^T315I+^ cells (Fig. S4F). Exogenous overexpression of integrin β3 on BCR-ABL1^+^ or BCR-ABL1^T315I+^ donor BM by retroviral cotransduction [20] (Fig. S3G), led to significantly reduced leukocyte counts in peripheral blood (*P* = 0.0031 for BCR-ABL1^+^ and *P* < 0.0001 for BCR-ABL1^T315I+^, Fig. 3e) and prolonged survival (*P* = 0.0018 for BCR-ABL1^+^ and *P* = 0.0025 for BCR-ABL1^T315I+^, Fig. 3f) in most recipients of integrin β3^+^ compared with empty vector-transduced BCR-ABL1^+^ or BCR-ABL1^T315I+^ donor BM. Homing of empty vector- or integrin β3-overexpressing LIC did not differ (Fig. S4H–I). In contrast, knockdown of integrin β3 on BCR-ABL1^+^ or BCR-ABL1^T315I+^ donor BM did not alter survival (Fig. S4J–K). Testing phagocytic activity, characteristic of mature myeloid cells, we demonstrated that` phagocytosis was significantly reduced in BCR-ABL1^T315I+^ versus BCR-ABL1^+^ myeloid cells (*P* = 0.0256, Fig. 3g). However, overexpression of integrin β3 on BCR-ABL1^T315I+^ myeloid cells ‘rescued’ or restored the phagocytosis of bacterial particles (*P* = 0.0418, Fig. 3g). In summary, these data suggested that integrin β3 plays a role in the outcome of, particularly, BCR-ABL1^T315I+^ CML, while also influencing the differentiation of BCR-ABL1^+^ and BCR-ABL1^T315I+^ myeloid cells.

### Fibronectin is decreased in the BMM of mice with BCR-ABL1^T315I+^ CML

**Fig. 4:**
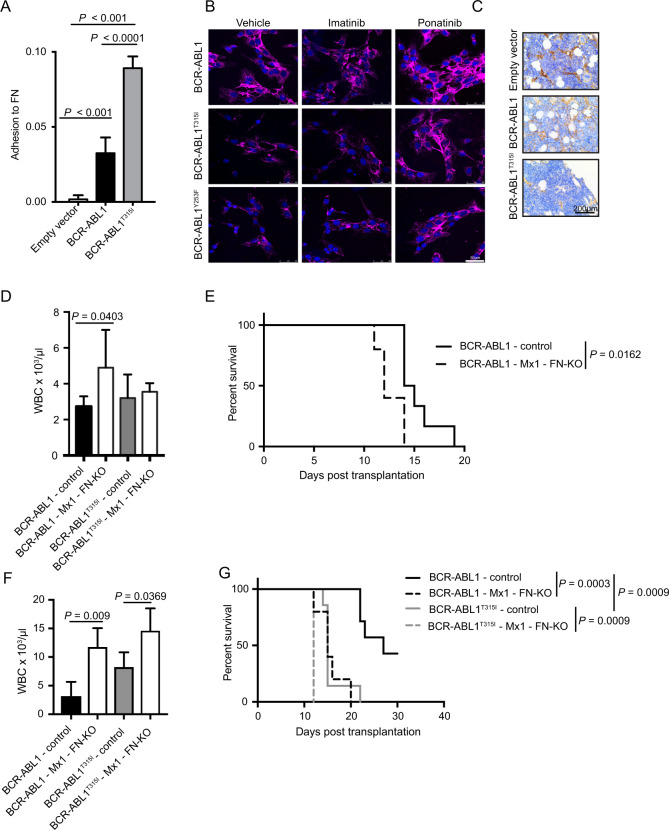
Fibronectin is decreased in the BMM of mice with BCR-ABL1^T315I+^ CML. **a** Optical density read at 570 nm after adhesion of sorted empty vector^+^, BCR-ABL1^+^, or BCR-ABL1^T315I+^ (GFP^+^) CD11b^+^ splenocytes to fibronectin (FN) in vitro (*P* < 0.0001 for BCR-ABL1 versus BCR-ABL1^T315I^; ANOVA, Tukey test, *n* = 3). 10^5^ cells had been plated and allowed to adhere for 72 h. The data are representative of three independent experiments. **b** Immunofluorescence of 3T3 fibroblasts transduced with BCR-ABL1-, BCR-ABL1^T315I^-, or BCR-ABL1^Y253F^-expressing retrovirus, treated with vehicle, 750 nM imatinib or 60 nM ponatinib for 6 h, stained with an antibody to fibronectin (pink). The nuclei are counterstained with DAPI. **c** Immunohistochemistry for fibronectin (detected by immunoperoxidase using yellow–brown horseradish-peroxidase chromogen) on bones of BALB/c recipient mice transplanted with empty vector-, BCR-ABL1-, or BCR-ABL1^T315I^-transduced bone marrow at time of death. The scale bar depicts 200 μm (*n* = 3). Leukocyte counts (WBC) × 10^3^ per μl in peripheral blood (*P* = 0.0403 for BCR-ABL1; *t*-test, *n* = 5–6) on day 12 after transplantation (**d**) and Kaplan–Meier-style survival curve (**e**) of C57BL/6 recipient mice transplanted with FN fl/fl Mx1-Cre^−^ or FN fl/fl Mx1-Cre^+^ bone marrow transduced with BCR-ABL1 (*P* = 0.0162, Log-rank test) (**e**) or BCR-ABL1^T315I^ (**d**). 10 mg/kg polyinosinic:polycytidylic acid (poly I:C) per dose was administered on days 1, 2, 3, and 5 after transplantation. **f**, **g** Leukocyte counts (WBC) × 10^3^ per μl in peripheral blood on day 12 after transplantation (*P* = 0.009 for BCR-ABL1^+^; *t*-test, *n* = 5–6) (**f**) and Kaplan–Meier-style survival curve (**g**) of C57BL/6 recipient mice transplanted with FN fl/fl Mx1-Cre^−^ or FN fl/fl Mx1-Cre^+^ bone marrow transduced with BCR-ABL1 (*P* = 0.0003, Log-rank test) or BCR-ABL1^T315I^ (**g**). Ten milligram per kilogram polyinosinic:polycytidylic acid (poly I:C) per dose was administered on days 8, 9, and 10 after transplantation.

Next, we tested the adhesion of empty vector-, BCR-ABL1- or BCR-ABL1^T315I+^ primary BM cells to the ECM protein fibronectin, one of the ligands of integrin β3. This revealed increased adhesion of BCR-ABL1^T315I+^ cells to fibronectin compared with empty vector- or BCR-ABL1-transduced cells (*P* < 0.0001, Fig. 4a). We hypothesized that BCR-ABL1^T315I+^ CML cells, similar to other cancers [29], may deposit less fibronectin. Indeed, 3T3 fibroblasts transduced with BCR-ABL1^T315I^ deposited significantly less fibronectin than BCR-ABL1^+^ fibroblasts (Fig. S5A). Deposition of fibronectin was kinase-dependent, as treatment of BCR-ABL1^+^, BCR-ABL1^Y253F+^, or BCR-ABL1^T315I+^ 3T3 fibroblasts with the TKI ponatinib significantly increased fibronectin deposition, while imatinib had no effect on imatinib-resistant BCR-ABL1 mutants (Fig. 4b). Less fibronectin was also found in the BMM of mice with CML due to BCR-ABL1^T315I^, BCR-ABL1^E255K^, or BCR-ABL1^Y253F^, but not BCR-ABL1^M351T^ compared with control mice (Figs. 4c and S5B). In order to test whether the generation of fibronectin by leukemia cells influences leukemia progression and accelerates BCR-ABL1^+^ CML similar to BCR-ABL1^T315I+^ CML, we transduced the BM of fibronectin flox/flox × Mx1-Cre (FN Mx1-Cre) mice with BCR-ABL1 or BCR-ABL1^T315I^, before transplantation into wildtype recipients and subsequent administration of poly I:C to induce Cre (Fig. S5C). This led to a significant increase of the number of leukocytes in the peripheral blood of mice that had received BCR-ABL1^+^ FN Mx1-Cre BM (*P* = 0.0403, Fig. 4d) and significant shortening of survival of those mice which received BCR-ABL1^+^ (*P* = 0.0162, Fig. 4e) or BCR-ABL1^T315I+^ FN Mx1-Cre BM compared with their respective controls (*P* = 0.0018, Fig. S5D). All mice succumbed to CML-like disease. Later deletion of fibronectin in LIC had the same effect (Fig. 4f, g). In contrast, no effect on survival was observed when BCR-ABL1^+^ or BCR-ABL1^T315I+^ wildtype BM was transplanted into fibronectin flox/flox × Col1a2-Cre (FN Col1a2-Cre) mice (Fig. S5E–F), characterized by lack of production of fibronectin by fibroblasts [36]. In summary, these data suggest that BCR-ABL1^T315I+^ CML cells produce less fibronectin than BCR-ABL1^+^ cells and that fibronectin production by leukemia cells influences CML progression.

### Integrin-linked kinase is involved in fibronectin deposition and influences survival in BCR-ABL1^T315I+^ CML

**Fig. 5:**
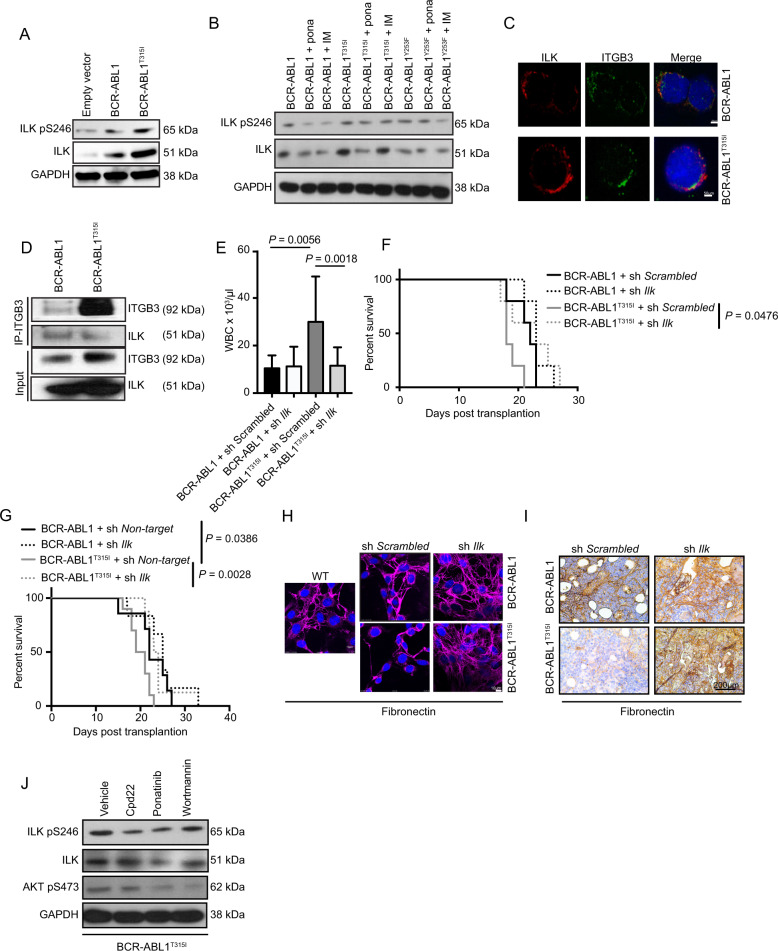
Integrin-linked kinase influences fibronectin deposition and survival in BCR-ABL1^T315I+^ CML. **a** Immunoblot showing the expression of ILK pS246 (65 kDa), ILK (51 kDa), or glycerinaldehyde-3-phosphate dehydrogenase (GAPDH) (38 kDa) in lysates of BA/F3 cells transduced with empty vector, BCR-ABL1 or BCR-ABL1^T315I^. The immunoblot is representative of three independent experiments. **b** Immunoblot showing the expression of ILK pS246 (65 kDa), ILK (51 kDa), or GAPDH (38 kDa) in lysates of BA/F3 cells transduced with BCR-ABL1- or BCR-ABL1^T315I^-expressing retrovirus treated with vehicle, 60 nM ponatinib or 750 nM imatinib for 4 h. The immunoblot is representative of three independent experiments. **c** Immunofluorescence of BA/F3 cells transduced with BCR-ABL1- or BCR-ABL1^T315I^-expressing retrovirus, stained with an antibody to ILK (red) or integrin β3 (green). The nuclei are counterstained with DAPI. The images are representative of three experiments. The scale bar represents 50 μm. **d** Coimmunoprecipitation (IP) of lysates of BA/F3 cells transduced with BCR-ABL1 or BCR-ABL1^T315I^ with an anti-integrin β3 (ITGB3) antibody. The immunoblot was performed with an antibody to integrin β3 (92 kDa) and ILK (51 kDa). **e**, **f** Leukocyte counts (WBC) × 10^3^ per μl in peripheral blood (*P* = 0.0018 for BCR-ABL1^T315I+^; *t*-test, *n* = 8–10) (**e**) and Kaplan–Meier-style survival curve (**f**) of BALB/c recipient mice transplanted with bone marrow cotransduced with BCR-ABL1- or BCR-ABL1^T315I^-expressing retrovirus and *Scrambled* or *Ilk* shRNA-expressing lentivirus (*P* = 0.0476 for BCR-ABL1^T315I+^, Log-rank test, *n* = 10). **g** Kaplan–Meier-style survival curve of BALB/c recipient mice transplanted with bone marrow cotransduced with BCR-ABL1- or BCR-ABL1^T315I^-expressing retrovirus and a lentivirus expressing inducible nontarget control or *Ilk* shRNA (*P* = 0.0028 for BCR-ABL1^T315I+^ and *P* = 0.0386 for BCR-ABL1, Log-rank test, *n* = 10). 50 mg/kg of doxcyclin to induce shRNA-expression was administered intraperitoneally to recipient mice on days 8, 9, 10, and 12 after transplantation. **h** Immunofluorescence of normal wildtype (WT) 3T3 fibroblasts or 3T3 fibroblasts transduced with BCR-ABL1- or BCR-ABL1^T315I^-expressing retrovirus and *Scrambled* or *Ilk* shRNA-expressing lentivirus. The nuclei are counterstained with DAPI. The images are representative of four independent experiments. **i** Immunohistochemistry for fibronectin (detected by immunoperoxidase using yellow–brown horseradish-peroxidase chromogen) on bones of representative BALB/c recipient mice transplanted with BCR-ABL1- or BCR-ABL1^T315I^- and sh *Scrambled* or sh *Ilk-*cotransduced bone marrow. **j** Western blot showing the expression of ILK pS246 (65 kDa), ILK (51 kDa), AKT pS473 (62 kDa), or GAPDH (38 kDa) as loading control in lysates of BA/F3 cells transduced with BCR-ABL1^T315I^ and treated with vehicle, 50 nM Cpd22, 60 nM ponatinib, or 40 nM wortmannin for 6 h.

Hypothesizing that BCR-ABL1^T315I+^ leukemia cells deposit less fibronectin than BCR-ABL1^+^ leukemia cells due to differences in signaling pathways, we focused on ILK, a pseudokinase belonging to the family of RAF-like kinases involved in integrin-mediated signal transduction [37] and fibronectin deposition [38, 39]. ILK is linked to the cytoplasmic domains of integrins β1 and β3 [40]. We demonstrated that levels of total ILK and ILK phosphorylated at its autophosphorylation site S246 (ILK pS246) were increased in BCR-ABL1^T315I+^ compared with BCR-ABL1^+^ BA/F3 (Figs. 5a and S6A–B) or Lin^−^ cells (Fig. S6C–D). Treatment with ponatinib reduced levels of ILK, and ILK pS246 in BCR-ABL1^+^ and BCR-ABL1^T315I+^ BA/F3 cells, suggesting that protein levels of ILK and possibly phosphorylation of ILK may be BCR-ABL1-dependent (Figs. 5b and S6E–F), though not directly mediated by the tyrosine kinase BCR-ABL1. ILK and integrin β3 colocalized in BCR-ABL1^+^ and BCR-ABL1^T315I+^ BA/F3 cells, but the staining pattern for ILK and integrin β3 was more diffuse and less punctate in BCR-ABL1^T315I+^ compared with BCR-ABL1^+^ cells (Figs. 5c and S6G). Consistently, in spite of the overexpression of integrin β3, binding between ILK and integrin β3 was reduced in BCR-ABL1^T315I+^ compared with BCR-ABL1^+^ cells (Figs. 5d and S6H). Cotransduction of donor BM with BCR-ABL1- versus BCR-ABL1^T315I^-expressing retrovirus and *scrambled*- or *Ilk* shRNA-expressing lentivirus (*P* = 0.0133, Fig. S6I) led to a significant reduction of leukocyte counts (*P* = 0.0018, Fig. 5e) and significant prolongation of survival (*P* = 0.0476, Figs. 5f and  S6I) in recipients of BCR-ABL1^T315I+^ sh *Ilk*^*+*^ donor BM compared with controls, also when knockdown of *Ilk* was induced at a later timepoint after transplantation (*P* = 0.0028, Figs. 5g and S6J). Knockdown of *Ilk* also led to an increase in fibronectin deposition by BCR-ABL1^T315I+^ sh *Ilk*^*+*^ 3T3 fibroblasts (Fig. 5h) and in the BMM of recipients of BCR-ABL1^T315I+^ sh *Ilk*^*+*^ donor BM compared with controls (Figs. 5i and S6K). As BCR-ABL1 only phosphorylates tyrosine and not serine residues, we tested the phosphorylation of ILK at pS246 after treatment of BCR-ABL1^T315I+^ BA/F3 cells with vehicle, the ILK inhibitor Cpd22, ponatinib or the phosphoinositide-3-kinase (PI3K) inhibitor wortmannin, as PI3K is activated by BCR-ABL1 [41]. This revealed that levels of pS246ILK were decreased by Cpd22, ponatinib, and wortmannin, while total ILK was mostly decreased by ponatinib (Figs. 5j and S6L–M), suggesting that PI3K may differentially phosphorylate ILK in BCR-ABL1^+^ versus BCR-ABL1^T315I+^ cells. Taken together, these data suggest that binding between ILK and integrin β3 is impaired in BCR-ABL1^T315I+^ cells and that ILK plays an important role in progression of BCR-ABL1^T315I+^ CML, at least partly via modulation of fibronectin levels in the BMM.

### Treatment with fibronectin prolongs survival in BCR-ABL1^T315I+^ CML

**Fig. 6:**
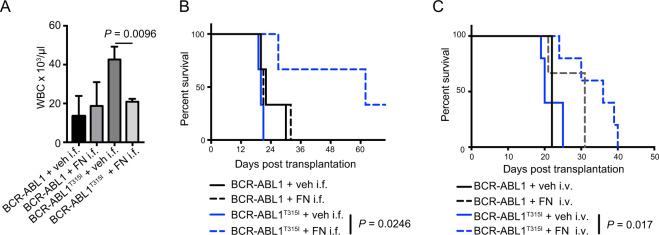
Treatment with fibronectin prolongs survival in BCR-ABL1^T315I+^ CML. **a**–**b** Leukocyte counts (WBC) × 10^3^ per μl in peripheral blood (*P* = 0.0096 for BCR-ABL1^T315I+^; *t*-test, *n* = 3) (**a**) and Kaplan–Meier-style survival curve (**b**) of BALB/c recipient mice transplanted with bone marrow transduced with BCR-ABL1 (black)- or BCR-ABL1^T315I^ (blue)-expressing retrovirus and treated with intrafemoral (i.f.) administrations of vehicle (solid line) or 50 μg fibronectin resuspended in 50 μl PBS (dashed line) per mouse per injection on days 0, 1, and 2 (*P* = 0.0246 for BCR-ABL1^T315I+^, Log-rank test). At the time of transplant (day 0), the leukemia-initiating cells had been resuspended in vehicle or fibronectin. **c** Kaplan–Meier-style survival curve of BALB/c recipient mice transplanted with bone marrow transduced with BCR-ABL1 (black)- or BCR-ABL1^T315I^ (blue)-expressing retrovirus and treated with intravenous (i.v.) administrations of vehicle (solid line) or 200 μg fibronectin per mouse per injection (dashed line) on days 9, 10, and 12 (*P* = 0.017 for BCR-ABL1^T315I+^, Log-rank test, *n* = 3–5).

Hypothesizing that replenishing reduced fibronectin levels in mice with BCR-ABL1^T315I+^ CML may decelerate leukemia progression, we transplanted LIC, resuspended in vehicle or fibronectin, intrafemorally into mice as proof of principle. This plus two further intrafemoral applications of fibronectin led to a significant reduction of leukocytes in peripheral blood (*P* = 0.0096; Fig. 6a) and significant survival prolongation (*P* = 0.0246; Fig. 6b) in mice with BCR-ABL1^T315I+^ CML compared with controls. Intravenous administration of fibronectin also significantly prolonged the survival of mice with BCR-ABL1^T315I+^ CML (*P* = 0.017; Fig. 6c) and increased fibronectin levels in the BM (Fig. S7A). However, administration of fibronectin to mice with BCR-ABL1^+^ B-cell acute lymphoblastic leukemia (Fig. S7B) [23] or MLL-AF9^+^ AML (Fig. S7C) [20, 42] did not lead to significant differences in survival. Taken together, these data suggest that fibronectin may be involved in regulating the progression of BCR-ABL1^T315I+^ CML.

### Treatment with the ILK inhibitor Cpd22 and ponatinib prolongs survival in BCR-ABL1^T315I+^ CML

**Fig. 7:**
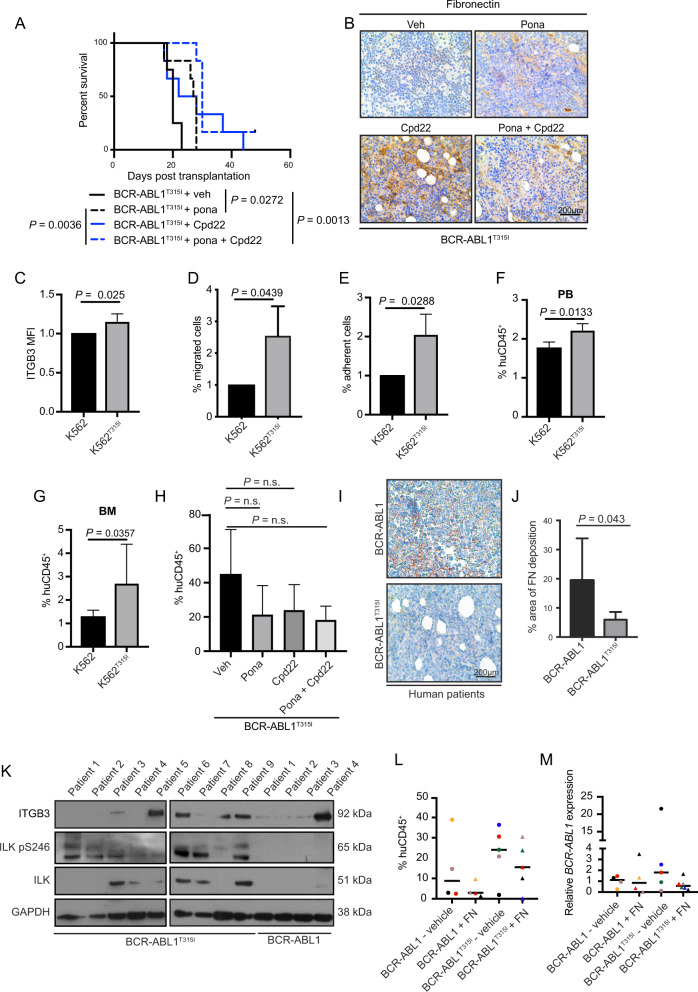
Levels of fibronectin are decreased and levels of integrin β3 and ILK are increased in human *BCR-ABL1*^*T315I+*^ CML cells. **a** Kaplan–Meier-style survival curve of BALB/c recipient mice transplanted with bone marrow transduced with BCR-ABL1^T315I^-expressing retrovirus and treated with vehicle (solid black line), 20 mg/kg ponatinib (dashed black line), 15 mg/kg Cpd22 (solid blue line), or ponatinib and Cpd22 (blue dashed line) (doses as above) (*P* = 0.0036 for ponatinib versus ponatinib plus Cpd22, Log-rank test, *n* = 5–6). Ponatinib was given daily on days 9–22, while Cpd22 was given daily on days 9–14 and every other day on days 14–22 after transplantation. **b** Immunohistochemistry for fibronectin (detected by immunoperoxidase using yellow–brown horseradish-peroxidase chromogen) on bones of BALB/c recipient mice transplanted with BCR-ABL1^T315I^-transduced bone marrow treated with vehicle, ponatinib, Cpd22, or the combination of ponatinib and Cpd22 as in (**a**). **c** Relative mean fluorescence intensity (MFI) of integrin β3 (ITGB3) on K562 (black) or K562^T315I^ (gray) cells (*P* = 0.025; *t*-test, *n* = 3). **d** Percentage of K562 (black) or K562^T315I^ (gray) cells which migrated to the bottom chamber containing MS-5 stroma cells in medium containing 10% serum in a transwell migration assay after 8 h (*P* = 0.0439; *t*-test, *n* = 4). In total, 10^5^ K562 cells and 30,000 MS-5 cells had been plated. **e** Percentage of K562 (black) or K562^T315I^ (gray) cells adhering to MS-5 stroma cells in vitro (*P* = 0.0288; *t*-test, *n* = 4). In total, 10^5^ K562 cells were plated on top of 30,000 MS-5 stroma cells. Cells had been allowed to adhere for 12 h. Percentage of human CD45^+^ leukocytes in the peripheral blood (PB) (**f**) or bone marrow (BM) (**g**) of NOD SCID interleukin-2 receptor γ knockout (NSG) mice transplanted with 3 × 10^6^ K562 (black) or K562^T315I^ (gray) cells on day 38 after transplantation. The mice had not been irradiated (*P* = 0.0133 for PB and *P* = 0.0357 for BM; *t*-test, *n* = 3–5). **h** Percentage of human CD45^+^ leukocytes in the bone marrow of NOD SCID interleukin-2 receptor γ knockout (NSG) mice transplanted with 3 × 10^6^ K562^T315I^ cells treated with vehicle, ponatinib, Cpd22 or ponatinib plus Cpd22 as in (**a**) at the time of death. The results are not significant (*n* = 4–7). Immunohistochemistry for fibronectin (detected by immunoperoxidase using yellow–brown horseradish-peroxidase chromogen) (**i**) and quantification of the area of fibronectin (FN) staining (**j**) in bone sections of patients with BCR-ABL1^+^ (black) or BCR-ABL1^T315I+^ (gray) CML (*n* = 4–11). **k** Immunoblot showing the expression of ILK pS246 (65 kDa), ILK (51 kDa), integrin β3 (92 kDa), or GAPDH (38 kDa) as loading control in lysates of leukocytes from the bone marrow or peripheral blood of patients with BCR-ABL1^+^ or BCR-ABL1^T315I+^ CML. Percentage of engraftment of human CD45^+^ (hCD45^+^) leukocytes (**l**), or relative levels of *BCR-ABL1*^*+*^ or *BCR-ABL1*^*T315I+*^ engraftment in BM aspirates from individual NSG recipients of primary human CML samples (peripheral blood or BM) on day 28 after transplantation (*n* = 4–5) (**m**). The recipient mice were treated with either saline or 200 μg/dose fibronectin on days 9, 10, 11, 13, and 15 after transplantation. Recipients of peripheral blood or BM grafts from the same human CML sample are indicated by the same individual symbols. Each human patient sample was transplanted into one vehicle- and two to three fibronectin-treated recipient mice.

In order to test whether inhibition of ILK may have a role for the treatment of BCR-ABL1^T315I+^ CML, we treated mice with BCR-ABL1^T315I+^ CML with vehicle, ponatinib, the ILK inhibitor Cpd22 [43], or the combination of Cpd22 and ponatinib. Cotreatment with the ILK inhibitor Cpd22 and ponatinib led to a modest, but significant prolongation of survival compared with treatment with ponatinib alone (*P* = 0.0036; Fig. 7a). Consistent with a presumed role of ILK in fibronectin deposition, treatment with Cpd22 alone, ponatinib alone, or the combination of Cpd22 and ponatinib increased the levels of fibronectin in bone sections (Fig. 7b). Treatment with Cpd22 or ponatinib increased the percentage of integrin β3^+^ BCR-ABL1^+^ (Fig. S8A) and BCR-ABL1^T315I+^ (Fig. S8B) cells. Integrin β3 clusters per area, which fortify the interaction with focal adhesosomes [44], also significantly increased by treatment of BCR-ABL1^T315I+^ cells with Cpd22 (*P* = 0.0051, Fig. S8C). In summary, inhibition of ILK with Cpd22 in combination with ponatinib prolongs survival in BCR-ABL1^T315I+^ CML. The benefit of ILK inhibition may lie in the combination of increasing fibronectin levels in the BMM, as well as in the further increase of integrin β3 on leukemia cells.

### The fibronectin/integrin β3/ILK-axis in human BCR-ABL1^T315I+^ CML cells

Validating our results in the human setting, we demonstrated increased levels of integrin β3 (*P* = 0.025, Figs. 7c and S8D–E), increased migration (*P* = 0.0439, Fig. 7d) and adhesion of K562^T315I^ (*P* = 0.0288, Fig. 7e) compared with K562 cells [45]. Transplantation of K562 versus K562^T315I^ cells into NOD SCID interleukin-2 receptor γ (NSG) knockout mice led to increased percentages of human CD45^+^ leukocytes in peripheral blood (*P* = 0.0133, Fig. 7f) and BM (*P* = 0.0357, Fig. 7g) in recipients of K562^T315I^ cells. Similar, but less striking results were observed after transplantation of KCL-22^T315I^ compared with KCL-22 cells (Fig. S8F–G). Treatment of NSG mice transplanted with K562^T315I^ cells with Cpd22, ponatinib or their combination led to a trend towards reduced engraftment of human CD45^+^ leukocytes compared with vehicle (Figs. 7h and S8H). Furthermore, fibronectin levels were significantly reduced in bone sections (*P* = 0.043; Fig. 7i, j) and levels of ILK, ILK pS246 and integrin β3 were higher in leukemia cell samples from most patients with BCR-ABL1^T315I+^ compared with BCR-ABL1^+^ CML, though inter-patient variability was observed (Figs. 7k and S8I). Lastly, transplantation of human BCR-ABL1^+^ versus BCR-ABL1T315I^+^ CML cells into NSG mice treated in pairs with vehicle or fibronectin led to a nonsignificant reduction of the engraftment of human CD45^+^ leukocytes in the majority of treated versus untreated mouse pairs (Fig. 7l). Treatment with fibronectin also led to a reduction of oncogene transcript levels in 2/4 (50%) recipients of human BCR-ABL1^+^ and 4/5 (80%) recipients of BCR-ABL1^T315I+^ CML cells (Fig. 7m). In summary, our data with human material suggest that the described link between fibronectin/integrin β3/ILK may also be applicable to human BCR-ABL1^T315I+^ CML.

## Discussion

In continuation of previous studies [15, 16] we demonstrate here that the increased oncogenicity of the *BCR-ABL1*^*T315I*^ mutation is at least partly due to differences in the interaction with the BMM and its remodeling compared with native *BCR-ABL1*. BCR-ABL1^T315I+^ cells differ from BCR-ABL1^+^ cells with regards to the actin cytoskeleton, migratory properties, expression of integrin β3, the levels and phosphorylation of FAK and ILK, as well as ILK-dependent deposition of fibronectin in the BMM. Further increased expression of integrin β3 led to increased myeloid maturation and a deceleration of leukemic progression, while a decrease of fibronectin expression in BCR-ABL1^+^ cells accelerated the disease similar to BCR-ABL1^T315I+^ CML. Administration of fibronectin decelerated BCR-ABL1^T315I+^ disease. In summary, our data suggest that interactions with proteins of the ECM influence CML progression and that therapeutic manipulation of the levels of ECM proteins may be beneficial in (resistant) CML.

Our data extend the observation that integrin β3 is essential for leukemogenesis and influences outcome in AML [34] and suggest that integrin β3—possibly via its interaction with ECM proteins such as fibronectin—may be involved in myeloid maturation, as suggested for integrin β1 [46].

Our findings on the differential location of normal HSPC and BCR-ABL1^+^ or BCR-ABL1^T315I+^ LIC are consistent with a previous study on the distinct physical engagement of the BMM by MLL-AF9^+^ AML cells, which is dependent on the degree of leukemic progression [18]. Our results further suggest that niche location of malignant cells may influence survival, possibly also in response to TKI treatment.

The involvement of ILK in the pathophysiology of BCR-ABL1^T315I+^ (and BCR-ABL1^+^) CML, given its linkage to the cytoplasmic domains of integrin β1 and β3, its support of scaffolding proteins [40] and its known role in the deposition of fibronectin by epithelial cells [47]—though opposite from our results in hematopoietic cells—and other ECM proteins [48] was not surprising and may be context dependent. The expression and activity of ILK is known to be increased in several epithelial cancers [49], where it may also contribute to chemoresistance via regulation of adhesion to fibronectin [50]. In addition, ILK has been postulated to be a novel target in solid cancers [49] and leukemia [51], though a connection to the BMM was not established. In our work, treatment with Cpd22 and ponatinib significantly prolonged survival in BCR-ABL1^T315I+^ CML compared with ponatinib alone.

Fibronectin is overexpressed in certain cancers and contributes to the tumorigenic process [52] contrary to our findings in BCR-ABL1^T315I+^ CML, where decreased levels of fibronectin exacerbate leukemia progression. In hematopoiesis, fibronectin supports the growth of HSPC [53, 54], whereas fibronectin may inhibit the proliferation of B-ALL cells in vitro [55]. Our data suggest that administration of fibronectin—or an increase of fibronectin levels via inhibition of ILK—may be beneficial in BCR-ABL1^T315I+^ CML, for which limited therapies or only treatments with significant side effects exist. Although fibronectin did not lead to a significant survival prolongation in BCR-ABL1^+^ B-ALL or MLL-AF9^+^ AML, it is likely that this may have been due to suboptimal dosing and timing of the administration of fibronectin, whose high purchasing costs were prohibitive of further exploration in this study.

In conclusion, we have demonstrated that a point mutation in the kinase domain of *BCR-ABL1*, which leads to imatinib resistance, in our models has altered biological activity and is associated with more aggressive disease due to altered interactions with the BMM via the fibronectin-integrin β3-ILK pathway. We confirmed altered, though variable expression levels and targetability of these proteins in human BCR-ABL1^T315I+^ CML cells. This may also explain the accelerated phenotype in humans. These altered interactions and specifically decreased levels of fibronectin or ILK in BCR-ABL1^T315I+^ CML are valuable targets. It is to be hoped that modification of this pathway will lead to the development of novel therapies for imatinib-resistant CML and, hopefully, other leukemias.

### Supplementary information

Supplementary figures, tables, methods

Supplementary table 3
