# Supplementary material for: Specific, targetable interactions with the microenvironment influence imatinib-resistant chronic myeloid leukemia
Source: Leukemia. 2020 May 21;34(8):2087–101. doi: 10.1038/s41375-020-0866-1 (PMC7387317; doi:10.1038/s41375-020-0866-1)
Supplement: Supplementary file 1 — Supplementary figures, tables, methods [file 41375_2020_866_MOESM1_ESM.pdf]

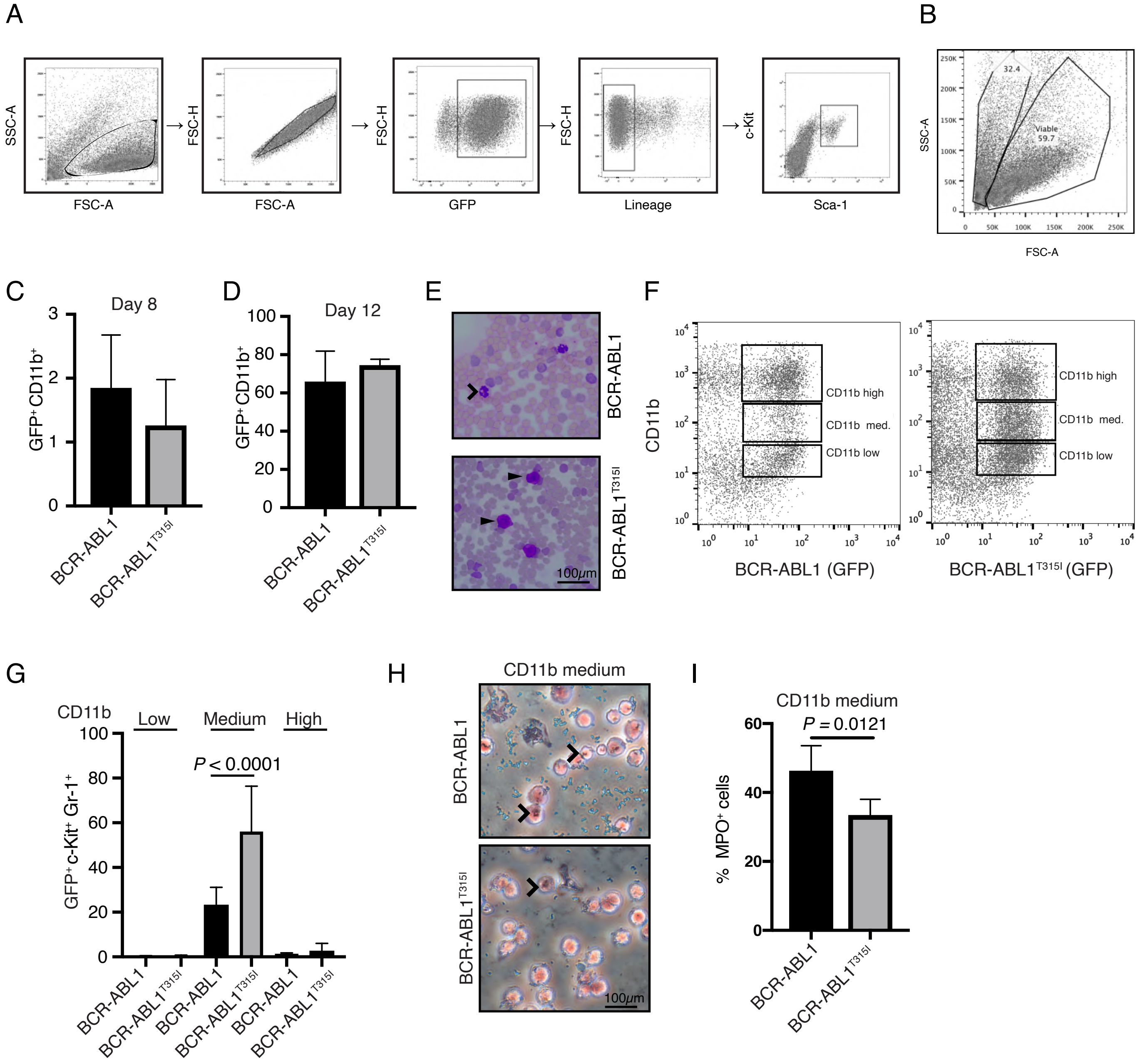

Supplementary figure 1: A) Gating strategy for the analysis of GFP+ (BCR-ABL1+ or BCR-ABL1T315I+) Lin- c-Kit+ Sca-1+ cells. B) Representative FACS plot of BCR-ABL1+ cells after imatinib treatment showing the percentage of viable cells. C-D) Percentage of GFP+ (BCR-ABL1+ or BCR-ABL1T315I+) CD11b+ cells in peripheral blood of mice with BCR-ABL1+ (black) or BCR-ABL1T315I+ (gray) CML on days 8 (C) or day 12 (D) after transplantation (n=4-6). E) Giemsa staining of peripheral blood from mice with BCR-ABL1+ (top) or BCR-ABL1T315I+ (bottom) CML on day 15 after transplantation. The open arrows point towards more mature myeloid cells, while the closed arrowheads point towards blasts. The slides are representative of 4-5 mice per group.The scalebar depicts 100 µm. F) Representative FACS plots of BCR-ABL1 (GFP)+ CD11b+ and BCR-ABL1T315I (GFP)+ CD11b+ cells gated on CD11b high+, CD11b medium+ and CD11b low+ cells in mice with BCR-ABL1+ (top) or BCR-ABL1T315I+ (bottom) CML on day 15 after transplantation. G) Percentage of BCR-ABL1 (GFP)+ c-Kit+ Gr-1+ (black) or BCR-ABL1T315I (GFP)+ c-Kit+ Gr-1+ (gray) cells gated on the CD11b low+, CD11b medium+ and CD11b high+ population ($P < 0.0001$; ANOVA, Tukey Test, n=4-5) from mice with BCR-ABL1+ or BCR-ABL1T315I+ CML on day 15 after transplantation. H-I) Representative image (H) and quantification (I) of sorted BCR-ABL1 (GFP)+ CD11b medium+ (top) or BCR-ABL1T315I (GFP)+ CD11b medium+ (bottom) cells stained for myeloperoxidase, characterized by black intracellular granules and open arrowheads, using a chromogen-based assay ($P = 0.0121$; t-test, n=4-5). The cells were taken from mice with BCR-ABL1+ or BCR-ABL1T315I+ CML on day 15 after transplantation.

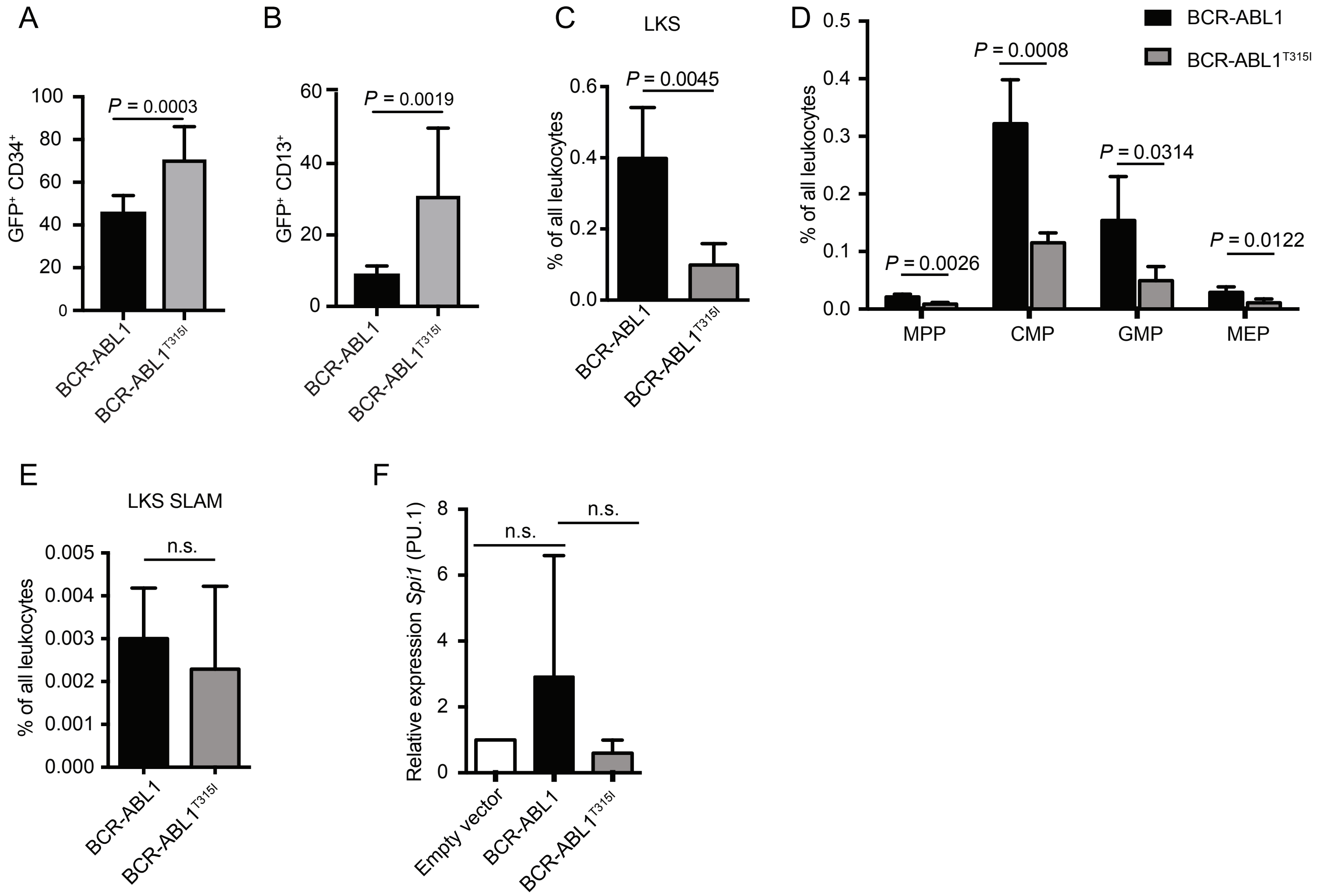

Supplementary figure 2: A-E) Percentage of BCR-ABL1 (GFP)+ or BCR-ABL1T315I (GFP)+ CD34+ (A), BCR-ABL1+ or BCR-ABL1T315I+ CD13+ (B), BCR-ABL1+ or BCR-ABL1T315I+ Lin- c-Kit+ Sca-1+ (LKS) cells (C), BCR-ABL1+ or BCR-ABL1T315I+ multipotent progenitors (MPP), common myeloid progenitors (CMP), granulocyte/macrophage progenitors (GMP) and megakaryocyte/erythroid progenitors (MEP) (D) and BCR-ABL1+ or BCR-ABL1T315I+ LKS CD150+ CD48-(SLAM) cells (E) in the bone marrow of BALB/c recipients of BCR-ABL1+ or BCR-ABL1T315I+ bone marrow on day 15 after transplantation (t-test, n=4-5). F) Relative expression of Spi1 (PU.1) in total bone marrow of murine recipients of empty vector (white)-, BCR-ABL1 (black)- or BCR-ABL1T315I (dark gray)- transduced donor bone marrow 15 days after transplantation (ANOVA, Tukey test) (n=5).

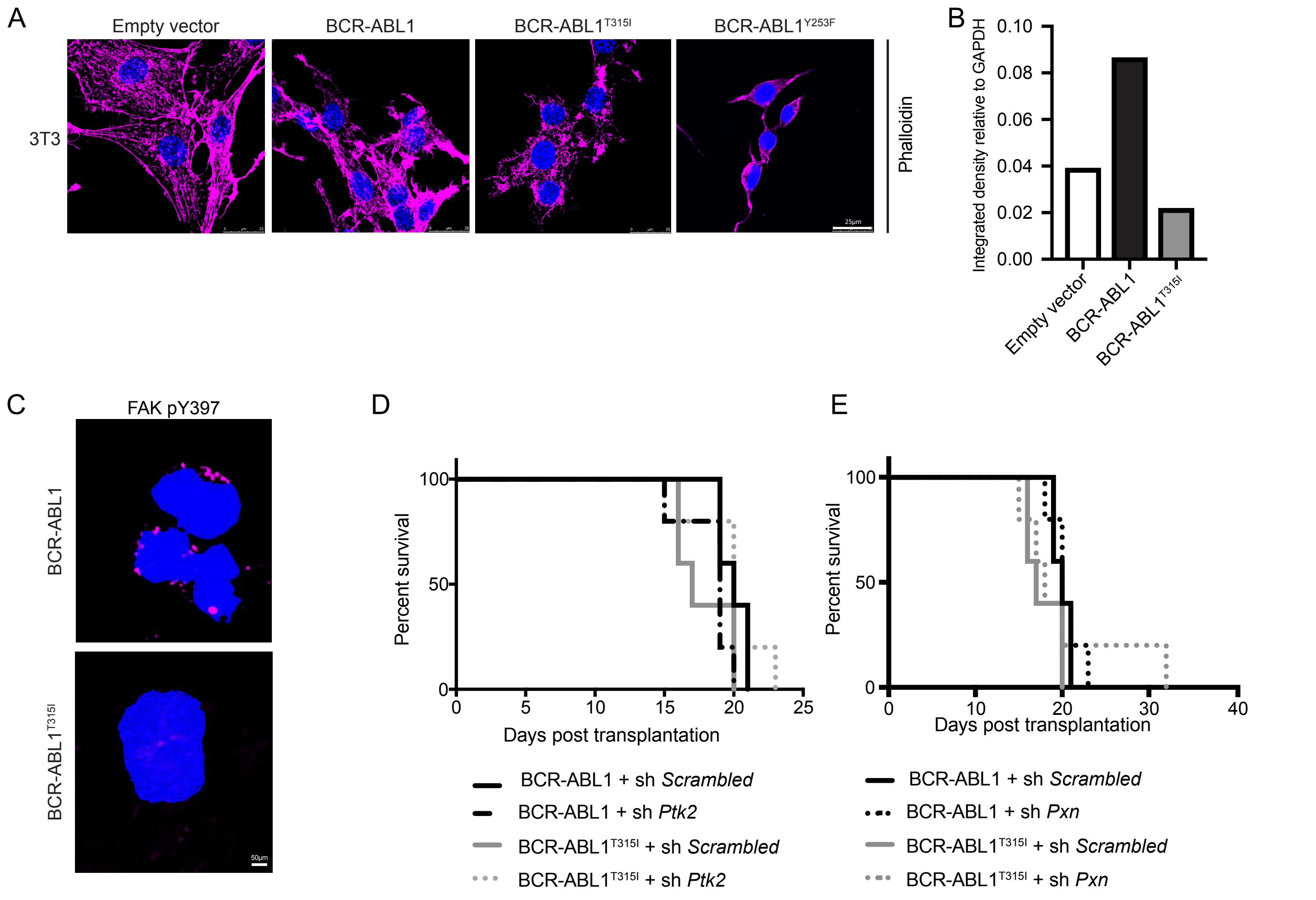

Supplementary figure 3: A) Immunofluorescence staining of 3T3 cells transduced with empty vector-, BCR-ABL1-, BCR-ABL1T315I- or BCR-ABL1Y253F- expressing retrovirus, stained with an antibody to phalloidin (pink). The nuclei are counterstained with DAPI. The images are representative of three independent experiments. The scalebar depicts 25 µm. B) Integrated relative density of the FAK pY397 band relative to GAPDH as loading control in figure 2D. C) Representative images of immunofluorescence studies of Lin- cells from the spleens of recipient mice transplanted with BCR-ABL1+ or BCR-ABL1T315I+ bone marrow, stained with an antibody to FAK pY397. The spleens were obtained on day 15 after transplantation (n=2). The scalebar depicts 50 µm. D-E) Kaplan-Meier-style survival curves of BALB/c recipient mice transplanted with bone marrow cotransduced with BCR-ABL1- or BCR-ABL1T315I (RFP)-expressing retrovirus and scrambled or Ptk2 (focal adhesion kinase; FAK) (n=5) (D) or Pxn (paxillin) (n = 5) (E) shRNA-expressing lentivirus.

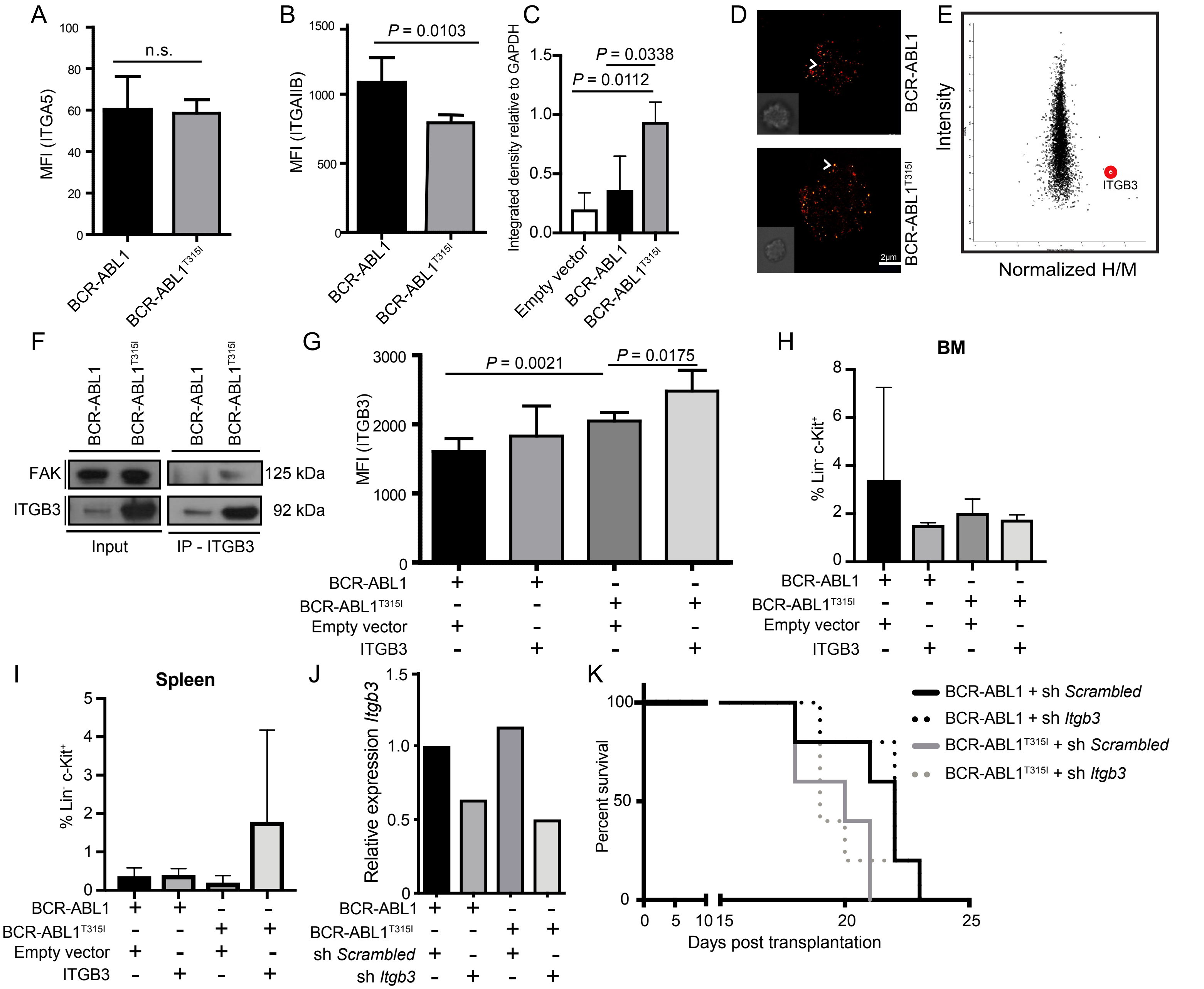

Supplementary figure 4: A-B) Mean fluorescence intensity (MFI) of integrin α5 (ITGA5) (A) or integrin α2b (ITGAIIB) (B) on BCR-ABL1 (GFP)+ (black) or BCR-ABL1T315I (GFP)+ (gray) CD11b+ cells from the bone marrow of mice with BCR-ABL1+ (black) or BCR-ABL1T315I+ (gray) CML on day 15 after transplantation (P = 0.0103 for ITGAIIB; t-test, n=5). C) Integrated relative density of the ITGB3 band relative to GAPDH as loading control in figure 3A (P = 0.0338; ANOVA, Tukey test, n=3). D) Super-resolution microscopy (dSTORM) images for ITGB3 on BA/F3 cells transduced with BCR-ABL1- (top) or BCR-ABL1T315I- (bottom) expressing retrovirus. Insets show the respective brightfield images. The white arrowheads point to ITGB3 epitopes labeled by the antibody. The data are representative of three experiments. E) Proteome comparison of BCR-ABL1+ (M; Labeled with medium SILAC amino acids) versus BCR-ABL1T315I+ (H; labeled with heavy SILAC amino acids) BA/F3 cells. Log10 transformed protein intensities are plotted against their log2 transformed normalized SILAC ratios (H/M). Integrin β3 (ITGB3) is circled in red (n=4). F) Co-immunoprecipitation of lysates of splenocytes transduced with BCR-ABL1 or BCR-ABL1T315I with an anti-ITGB3 antibody. The Western blot was performed with an antibody to ITGB3 (92 kDa) and FAK (125 kDa). G) Mean fluorescence intensity of ITGB3 on BCR-ABL1 or BCR-ABL1T315I (red fluorescent protein; RFP)+ CD11b+ myeloid cells from BALB/c recipient mice of donor bone marrow co-transduced with BCR-ABL1 or BCR-ABL1T315I (RFP+)- and empty vector- or integrin β3 (ITGB3)-overexpressing retrovirus on day 15 after transplantation (ANOVA; Tukey test, n=5). H-I) Percentage of BCR-ABL1+ or BCR-ABL1T315I+ (GFP+) Lin- c-Kit+ cells, which had homed to the bone marrow (H) or spleen (I) of recipient mice transplanted with BCR-ABL1+ or BCR-ABL1T315I+ bone marrow overexpressing empty vector or integrin β3 (ITGB3) eighteen hours post transplantation. A total of 2.5 x 10^6 cells had been transplanted (n=5). J) Relative expression of Itgb3 in BA/F3 cells after transduction with BCR-ABL1- or BCR-ABL1T315I-expressing retrovirus and sh scrambled- or sh Itgb3-expressing lentivirus showing the knockdown efficiency. K) Kaplan-Meier-style survival curve of BALB/c recipient mice transplanted with bone marrow cotransduced with BCR-ABL1- or BCR-ABL1T315I - expressing retrovirus and scrambled or Itgb3 shRNA-expressing lentivirus. A total of 2.5 x 10^5 cells had been transplanted (n=10).

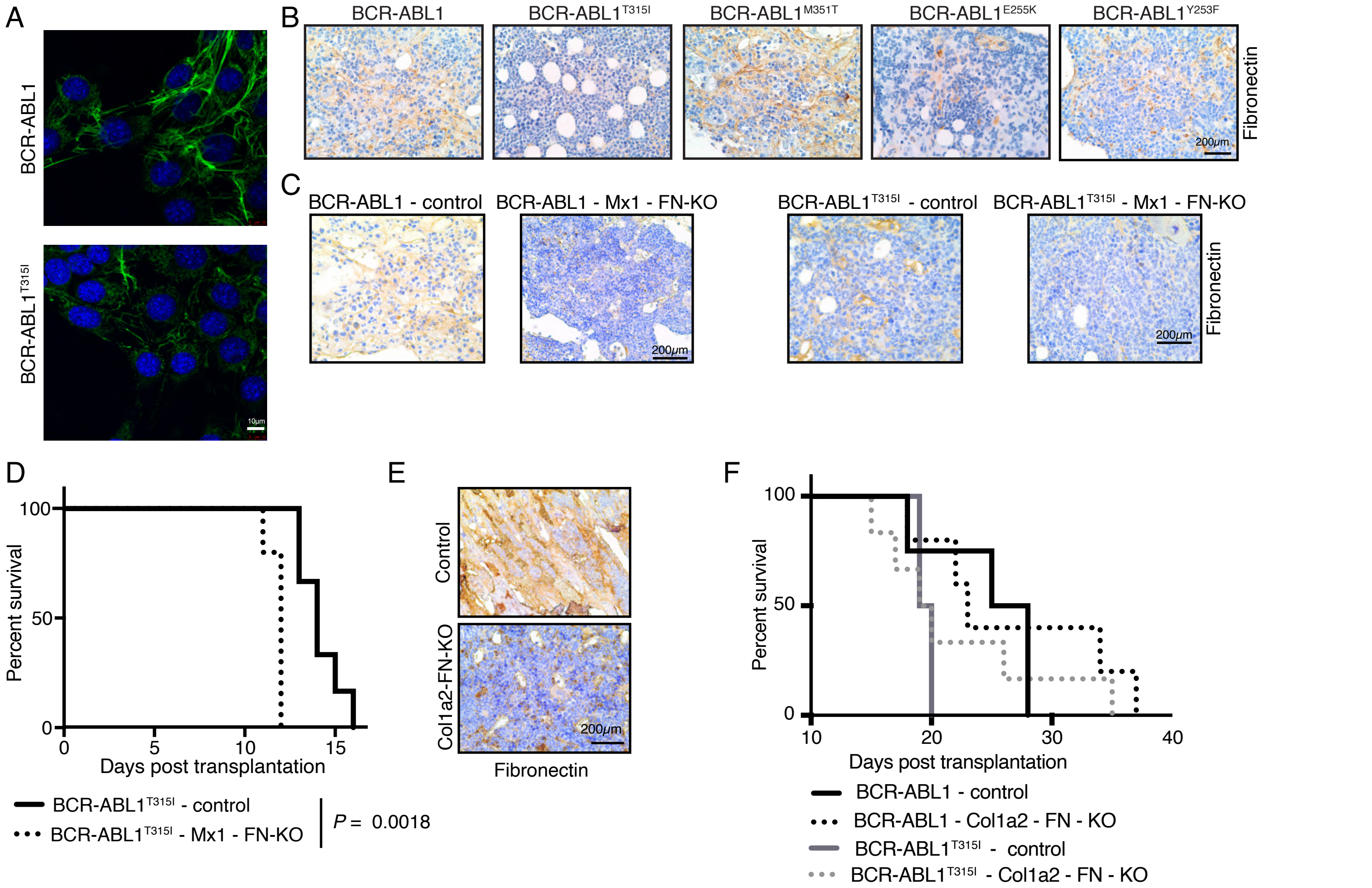

Supplementary figure 5: A) Immunofluorescence staining of 3T3 cells transduced with BCR-ABL1- or BCR-ABL1T315I-expressing retrovirus, stained with an antibody to fibronectin (green). The nuclei are counterstained with DAPI. The images are representative of three experiments. B) Immunohistochemistry of bone sections from BALB/c recipient mice transplanted with bone marrow transduced with BCR-ABL1-, BCR-ABL1T315I-, BCR-ABL1M351T-, BCR-ABL1E255K- or BCR-ABL1Y253F-expressing retrovirus stained with an antibody to fibronectin (detected by immunoperoxidaseusing yellow-brown horseradish-peroxidase chromogen).The images are representative of three mice per group. The scale bar represents 200 µm. C) Immunohistochemistry of bone sections from wildtype (C57Bl/6) recipient mice transplanted with control (Fibronectin (FN) flox/flox Mx1-Cre-negative) or FN flox/flox Mx1-Cre-positive bone marrow transduced with BCR-ABL1- or BCR-ABL1T315I-expressing retrovirus stained with an antibody to fibronectin (detected by immunoperoxidase using yellow-brown horseradish-peroxidase chromogen). The sections were from the time of death. Poly I:C (10mg/kg) to induce Cre was injected intraperitoneally on days 1, 2, 3 and 5. The images are representative of three mice per group. The scale bar represents 200 µm. D) Kaplan-Meier--style survival curve of C57Bl/6 recipient mice transplanted with control (FN flox/flox Mx1-Cre-negative) or FN flox/flox Mx1-Cre-positive bone marrow transduced with BCR-ABL1T315I-expressing retrovirus ($P = 0.0018$, Log-rank test). Poly I:C(10 mg/kg) to induce Cre was injected intraperitoneally on days 1, 2, 3 and 5.
E) Immunohistochemistry of bone sections from fibronectin (FN) flox/flox Col1a2-Cre-negative control or FN flox/flox Col1a2-Cre-positive recipient mice transplanted with wildtype littermate bone marrow transduced with BCR-ABL1- or BCR-ABL1T315I-expressing retrovirus, stained with an antibody to fibronectin (detected by immunoperoxidase using yellow-brown horseradish-peroxidase chromogen). The sections were taken at the time of death. Tamoxifen (2mg/dose) to induce Cre was injected intraperitoneally on days 1, 2, 3 and 5. The images are representative of 3 mice per group. The scale bar represents 200 µm. F) Kaplan-Meier-style survival curve of flox/flox Col1a2-Cre-negative control or FN flox/flox Col1a2-Cre-positive recipient mice transplanted with BCR-ABL1- or BCR-ABL1T315I- transduced bone marrow (n=5-6). Tamoxifen (2mg/dose) to induce Cre was injected intraperitoneally on days 1, 2, 3 and 5. The differences in survival are not significant (Log-rank test).

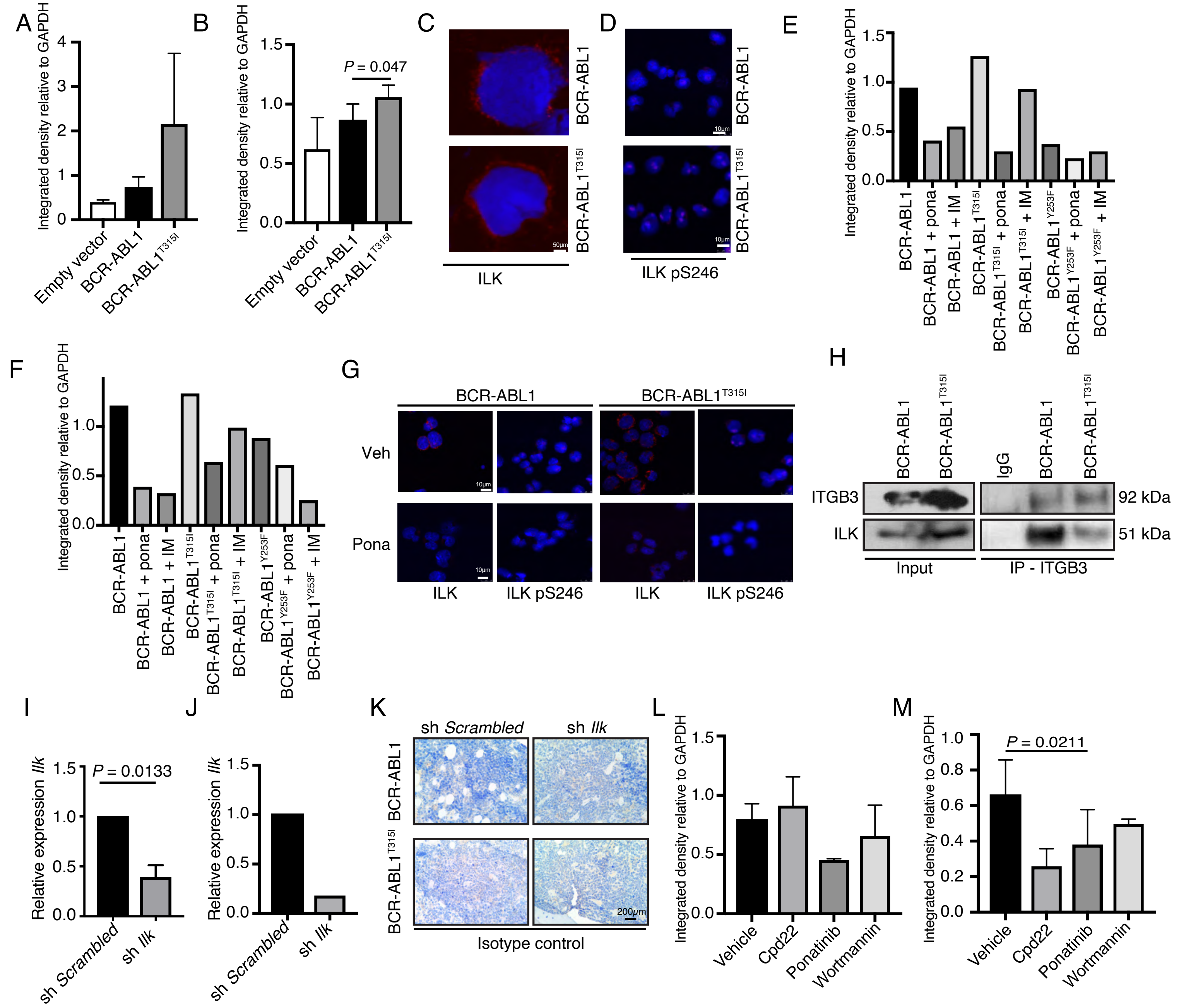

Supplementary figure 6: A-B) Integrated relative density of the ILK (A) and ILK pS246 (B) bands relative to the GAPDH loading control in figure 5A (P = 0.047; ANOVA, Tukey test, n=3). C) Immunofluorescence images of (lineage-depleted) Lin- bone marrow cells from mice with established BCR-ABL1+ or BCR-ABL1T315I+ CML, stained with an antibody to ILK (red) (C) or ILK pS246 (red) (D). The images are representative of three independent experiments. The nuclei are counterstained with DAPI. E-F) Integrated relative density of the ILK (E) and ILK pS246 (F) bands relative to the GAPDH loading control in figure 5B. G) Immunofluorescence images of BA/F3 cells transduced with BCR-ABL1- or BCR-ABL1T315I-expressing retrovirus, stained with an antibody to ILK or pS246ILK after treatment with vehicle or 60 nM ponatinib for 4-6 hours. The nuclei are counterstained with DAPI. The images are representative of three experiments. H) Coimmunoprecipitation of lysates of total splenocytes from mice with BCR-ABL1+ or BCR-ABL1T315I+ CML with an anti-integrin β3 antibody. The Western blot was performed with an antibody to ILK (51 kDa) or ITGB3 (92kDa). The data are representative of two experiments. I) Relative expression of Ilk in 3T3 cells after transduction with sh scrambled or sh Ilk-expressing lentivirus, as in the experiment in Figure 5F (P = 0.0133; t-test). The data are representative of three independent experiments. J) Relative expression of Ilk in 3T3 cells after transduction with inducible sh scrambled or sh Ilk-expressing lentivirus, as in the experiment in Figure 5G. 10 μg/ml doxycycline was added to the 3T3 culture for 4 days. K) Immunohistochemistry of bone sections from representative recipient BALB/c mice transplanted with bone marrow cotransduced with BCR-ABL1- or BCR-ABL1T315I-expressing retrovirus and sh scrambled- or sh Ilk-expressing lentivirus, stained with an isotype control IgG antibody (detected by immunoperoxidase using yellow-brown horseradish-peroxidase chromogen). The images are representative of three mice per group. The scale bar represents 200 μm. L-M) Integrated relative density of the ILK (L) and ILK pS246 (M) bands relative to the GAPDH loading control in figure 5J.

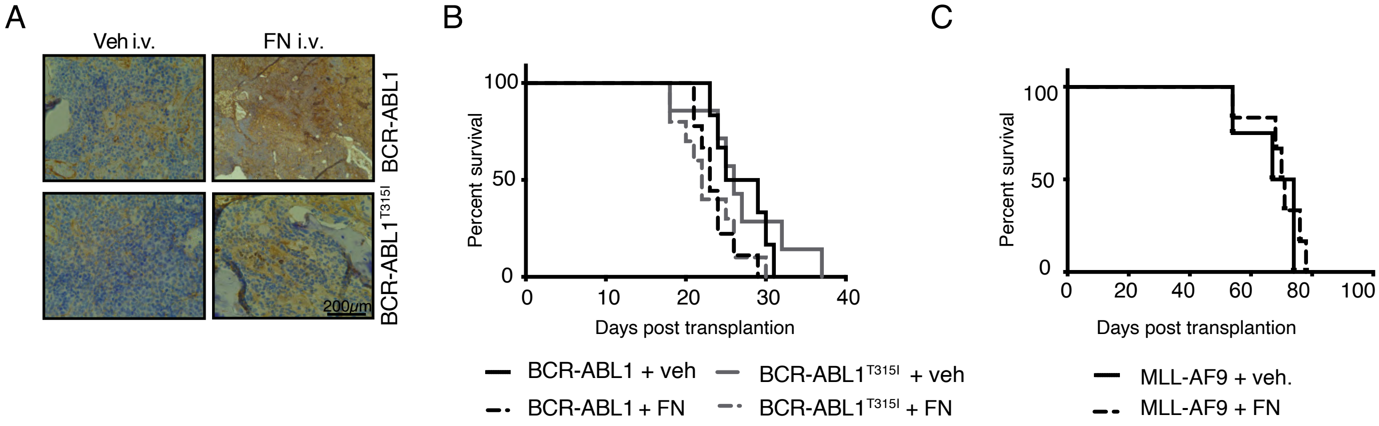

Supplementary figure 7: A) Immunohistochemistry of bone sections from representative recipient BALB/c mice transplanted with bone marrow transduced with BCR-ABL1- or BCR-ABL1T315I-expressing retrovirus and treated with intravenous doses of 200 µg/injection of fibronectin on days 9,10 and 12 after transplantation, stained with an antibody to fibronectin (detected by immunoperoxidase using yellow-brown horseradish-peroxidase chromogen). The images are representative of three mice per group. The scale bar represents 200 µm. B) Kaplan-Meier-style survival curve of BALB/c recipient mice transplanted with non-5-fluorouracil-pretreated bone marrow transduced with BCR-ABL1 (black)- or BCR-ABL1T315I (gray)– expressing retrovirus in the model of B-cell acute lymphoblastic leukemia. Recipient mice were treated with intravenous administrations of vehicle (solid line) or 200 µg fibronectin/injection (dashed line) on days 10, 11, 12, 14 and 15 after transplantation (n=6-10). C) Kaplan-Meier-style survival curve of BALB/c recipient mice transplanted with 5-fluorouracil-pretreated bone marrow transduced with MLL-AF9-expressing retrovirus in the model of acute myeloid leukemia. Recipient mice were treated with intravenous administrations of vehicle (solid line) or 200 µg fibronectin/injection (dashed line) on days 20, 22, 24, 26 and 28 after transplantation (n=4-6).

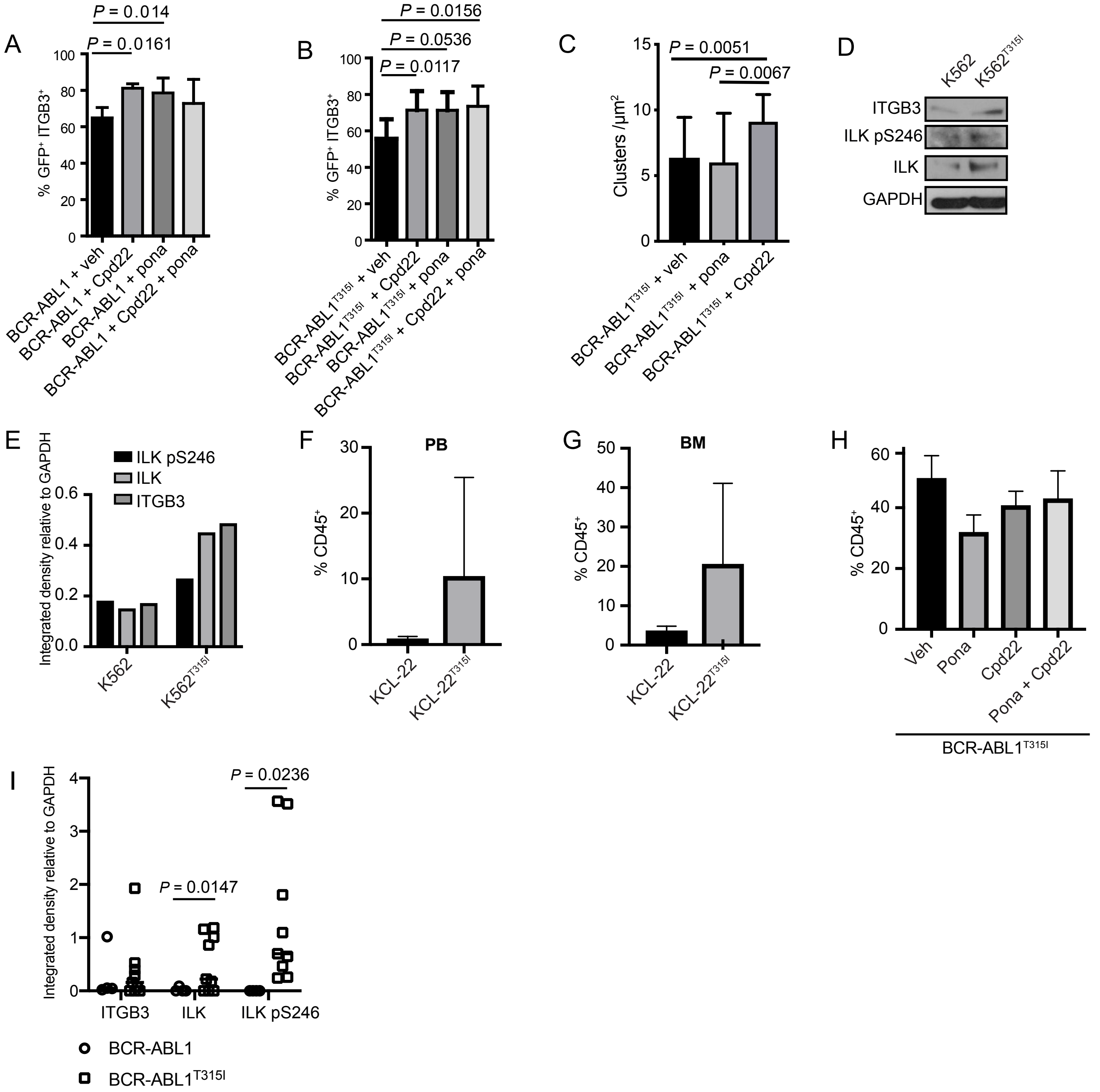

Supplementary figure 8: A-B) Percentage of GFP+ integrin β3+ (ITGB3+) BA/F3 cells transduced with BCR-ABL1 (A)- or BCR-ABL1T315I (B)-expressing retrovirus, treated with vehicle, the ILK-inhibitor Cpd22 (50 nM), ponatinib (60 nM) or Cpd22 plus ponatinib. The cells were treated for 4-8 hours. The data are representative of three independent experiments (ANOVA, Tukey Test). C) Clusters of integrin β3 per μm^2 on BA/F3 cells transduced with BCR-ABL1T315I and treated with vehicle, the ILK-inhibitor Cpd22 (50 nM), ponatinib (100 nM) or Cpd22 plus ponatinib for six hours. The data are representative of four independent experiments, as measured by super-resolution microscopy (dSTORM) (ANOVA, Tukey Test). D) Immunoblot showing the expression of ITGB3 (92kDa), ILKpS246 (65 kDa), ILK (51 kDa) or GAPDH (38 kDa) as loading control in lysates of K562 or K562T315I cells. The immunoblot is representative of three independent experiments. E) Integrated relative density of the ILK, ILK pS246 and ITGB3 bands relative to the GAPDH loading control in D. F-G) Percentage of human CD45+ cells in the peripheral blood (F) or bone marrow (G) of NSG mice injected with 3 x 10^6 KCL-22 or KCL-22T315I cells 45 days after transplantation (n=3-4). H) Percentage of human CD45+ cells in the peripheral blood of NSG mice injected with 3 x 10^6 K562T315I cells and treated with vehicle, ponatinib, Cpd22 and combination of ponatinib andCpd22. The peripheral blood was analyzed 25 days post stopping treatment for CD45+ cells (n=4-7). I) Integrated relative density of the ITGB3, ILK and ILK pS246 bands relative to the GAPDH loading control in Figure 7K.

Supplementary Table 1: Antibodies used in the study.

Antibody	Fluorochrome	Clone	Company	Catalogue number
Phalloidin	AlexaFluor 647		Thermo Fisher Scientific, Darmstadt, Germany	A22287
pCRKL		Y207	Cell Signaling Technology, Danvers, MA, USA	3181S
Annexin V	APC		Biolegend, San Diego, CA	640920
Streptavidin	APC-Cy7		Biolegend, San Diego, CA	405208
CD48	PB	HM48-1	Biolegend, San Diego, CA	103413
CD150	PE	Q38-480	BD Biosciences, San José, CA	562651
Sca-1	PE-Cy7	D7	Biolegend, San Diego, CA	108114
c-Kit	APC	2B8	BD Biosciences, San José, CA	553356
CD5-Biotin		53-73	BD Biosciences, San José, CA	553019
B220-Biotin		RA3-6B2	BD Biosciences, San José, CA	553086
F4/80-Biotin		BM8	eBiosciences, San Diego, CA	13-4801-82
Ly6G- and Ly6C-Biotin		RB6-8C5	BD Biosciences, San José, CA	553125
Ter119-Biotin			BD Biosciences, San José, CA	553672
FAK			Cell Signaling Technology, Danvers, MA, USA	3285S
FAKpY397			BD Biosciences, San José, CA	611722
FAKpY925			Cell Signaling Technology, Danvers, MA, USA	3284S
GAPDH			Abcam, Berlin, Germany	Ab2264
Gr-1	APC-Cy7	RB6-8C5	BD Biosciences, San José, CA	557661
CD11b	APC	M1/70	BD Biosciences, San José, CA	553312
Integrin β3 (CD61)	PE	2C9-G2	BD Biosciences, San José, CA	561910
Fibronectin			Abcam, Berlin, Germany	Ab2413
ILK			BD Biosciences, San José, CA	811802
ILK pS246			Merck Millipore, Burlington, MA, USA	AB1076
Integrin β3 (CD61)	Biotin	2C9-G2	Biolegend, San Diego, CA	104304
CD34	PE	MEC 14.7	Biolegend, San Diego, CA	119307
Paxillin			Abcam, Berlin, Germany	Ab2264
Integrin β3			Cell Signaling Technology, Danvers, MA, USA	4702S
CD13	AlexaFluor 647	R3-242	BD Biosciences, San José, CA	564352
Human CD45	PE	HI30	BD Biosciences, San José, CA	555483
Rabbit IgG			Cell Signaling Technology, Danvers, MA, USA	2729S

PE=Phycoerythrin; FITC= Fluorescein Isothiocyanate; BV=Brilliant Violet; APC= Allophycocyanin; Cy=Cyanine; PB=Pacific Blue

Supplementary table 2: Primers used in the study.

Genes	Forward primer	Reverse primer
CEBPα	CGATGAGCAGTCACCTCCAG	TATAGACGTCTCGTGCTCGC
SPI1 (PU.1)	CTCACCTACCAGTTCAGCGG	GGGCGACGGGTTAATGCTAT
ILK	AAG GTG CTG AAG GTT CGA GA	ATA CGG CAT CCA GTG TGT GA
FAK	CTTCTCAAAGTCACTGCTGCCT	TGGTGTGTGATTCAAGTTTGGG
Paxillin	TTCCAGATACGCTCACCAGC	GGGGAAGCTGTAGACGTGC
GAPDH	AGGTCGGTGTGAACGGATTTG	GGGGTCGTTGATGGCAACA
Itgβ3	TTACCACGGATGCCAAGACC	CCCCAGAGATGGGTAGTCCA
BCR-ABL1	GTGCAGAGTGGAGGGAGAAC	ATGCTACTGGCCGCTGAA
β-glucuronidase	GTCTGCGGCATTTTGTCGG	ACCTCCCGTTCGTACCACA

CEBPα = CCAAT/enhancer-binding protein alpha; Spi1 (PU.1) = Spi-1 Proto-oncogene;
ILK = integrin-linked kinase; FAK = focal adhesion kinase; GAPDH= Glyceraldehyde 3-phosphate dehydrogenase

Supplemental Materials and Methods

Data Sharing Statement

Additional data, including on mass spectrometry results, may be found in a data supplement available with the online version of this article or can be obtained by contacting krause@gsh.uni-frankfurt.de.

Mice

5 to 6 week old BALB/c mice were purchased from Charles River Laboratories. Fibronectin fl/fl, Col1a2-Cre and Mx1-Cre mice (all in a C57/Bl6 background) were kind gifts from Reinhard Faessler, Jörg Distler and Martin Zörnig, respectively. NOD SCID interleukin (IL)-2 receptor γ knockout (NSG) mice were bred in the animal facility of our institute. All animal studies were approved by the local German government (Regierungspräsidium Darmstadt) in Hessen, Germany.

Bone marrow transduction and transplantation

Generation of MSCV-IRES GFP BCR-ABL1 or MSCV-IRES GFP BCR-ABL1^{T315I} retrovirus and transplantation were performed as described [1]. Briefly, donor BM cells were harvested from 5-fluorouracil (5-FU) treated mice, transduced twice with cryopreserved retrovirus expressing MSCV-IRES GFP BCR-ABL1 or MSCV-IRES GFP BCR-ABL1^{T315I} (or the other mutants) and transplanted without sorting into sublethally irradiated recipient mice (750 cGy for Balb/c or 900 cGy for the fibronectin fl/fl-Col1a2-Cre- or -Mx1-Cre mice (C57BL/6 background)) at a dose of 2.5-3x10^5 cells i.v. All viral titers were matched.

For the knockdown experiments, 2.5-3×10^5 unsorted BM cells were transplanted after cotransduction of 5-FU–treated bone marrow with MSCV IRES RFP (red fluorescent protein) BCR-ABL1 or MSCV-IRES RFP BCR-ABL1^{T315I} retrovirus and sh *scrambled* control or sh *Ilk*-, sh *Itgb3*-, sh *Ptk2* (*Fak)*- or sh *Pxn*- expressing lentivirus. For the β3 integrin

overexpression experiments 5-FU–treated BM cells were cotransduced with MSCV IRES RFP BCR-ABL1 or MSCV-IRES RFP BCR-ABL1^{T315I} and MSCV IRES GFP empty vector or MSCV IRES GFP β3 integrin [2]-expressing retrovirus and transplanted into sublethally irradiated recipient BALB/c mice (750 cGy).

B-ALL was induced by retroviral transduction of non-5-FU-pretreated bone marrow with MSCV IRES GFP BCR-ABL1- or MSCV-IRES GFP BCR-ABL1^{T315I}-expressing retrovirus and transplantation (10^6 cells) into irradiated mice (750 cGy), while AML was induced by transplantation of 5 x 10^5 unsorted 5-FU-pretreated bone marrow cells transduced with MLL-AF9, as described [1].

In the homing assay 5-FU–treated bone marrow cells were cotransduced with MSCV IRES RFP BCR-ABL1- or MSCV-IRES RFP BCR-ABL1^{T315I}- and MSCV IRES GFP empty vector- or MSCV IRES GFP integrin β3-expressing retroviri. Then 2.5 × 10^6 cells were transplanted into sublethally irradiated recipient BALB/c mice (750 cGy). 18 hours post transplantation bone marrow and spleen of the recipient mice were analyzed for BCR-ABL1$^+$ (GFP$^+$) Lin$^-$ c-Kit$^+$ cells by flow cytometry on a BD Fortessa (Heidelberg, Germany).

Xenotransplantation experiments

NSG mice were transplanted intravenously with 3 x 10^6 KCL-22, KCL-22^{T315I}, K562 or K562^{T315I} cells. Peripheral blood or bone marrow (at the time of sacrifice) were analyzed for the presence of human CD45$^+$ cells by flow cytometry. Ponatinib (Selleckchem, Munich, Germany) was given by oral gavage at 20 mg/kg (three times a week)[3,4] and Cpd22 (Merck Millipore, Darmstadt, Germany) was given at 15 mg/kg intraperitoneally (twice a week)[5], starting on day 14 post transplantation of K562 or K562^{T315I} cells. Treatment was stopped after four weeks.

For transplantation of human CML samples (peripheral blood or bone marrow), NSG mice were irradiated with 325 cGy, and 7.5 x 10^5 to 3 x 10^6 total peripheral blood or bone marrow cells from patients with BCR-ABL1$^+$ or BCR-ABL1T315I$^+$ CML were transplanted into one or two

vehicle- and one to three fibronectin-treated recipient mice per patient sample. Fibronectin was administered intravenously at 200 μg/dose on days 9, 10, 11, 13 and 15 after transplantation. Peripheral blood and bone marrow were analyzed for human $CD45^{+}$ cells by flow cytometry. Cells from bone marrow aspirates, performed on day 28 after transplantation, were pelleted, and RNA was, subsequently, isolated using the Qiagen RNeasy Mini Kit/Qiagen RNeasy Micro Kit (Qiagen, Hilden, Germany). qPCR was performed for BCR-ABL1 and human β-glucuronidase transcript levels, as described [1].

Analysis of diseased mice and tumor burden

The leukemic burden in diseased animals was assessed by analysis of peripheral blood by a Scil Vet animal blood counter (Scil Animal Care Company, Viernheim, Germany) and by flow cytometry. Leukocytes from peripheral blood were analyzed with respect to GFP or RFP (BCR-ABL1 or BCR-ABL1^{T315I}) expression after staining with a phycoerythrin (PE)- or allophycocyanin (APC)-conjugated antibody to CD11b (clone M1/70, Biolegend, San Diego, CA) or Gr-1 (clone RB6-8C5, BD Biosciences, San José, CA) (myeloid cells). Samples were analyzed on a BD Fortessa (Heidelberg, Germany), while the data were analyzed with FlowJo. Other antibodies used are listed in Supplementary Table 1.

Cell lines and vectors

BA/F3 cells were transduced twice with MSCV empty vector-, MSCV IRES GFP BCR-ABL1- or MSCV IRES GFP BCR-ABL1^{T315I}- (or other BCR-ABL1 mutants-) expressing retrovirus, sorted and grown in RPMI, 10% fetal bovine serum, penicillin/streptomycin and L-glutamine. The medium for the MSCV empty vector-transduced cells was supplemented with 5 % (v/v) WEHI as a source of interleukin (IL)-3. 3T3 cells were transduced once.

The MSCV IRES GFP vectors coexpressing BCR-ABL1, BCR-ABL1^{T315I}, BCR-ABL1^{M351T}, BCR-ABL1^{Y253F} or BCR-ABL1^{E255K} and the MSCV BCR-ABL1 IRES RFP vector were kind

gifts from Rick Van Etten. The MSCV BCR-ABL1^{T315I} IRES RFP vector was cloned by overlap extension PCR using the external oligonucleotides 5'-GTATCCGCTGAGCAGCGGGATC-3' and 5'-TCACTGGCTCAGCTGCAGCAGG-3' and the internal oligonucleotides 5'-GAGGTTCCCGTAGGTCATGAAtTCAaTGATGATATAGAACGGGGGCTC-3' and 5'-GAGCCCCCGTTCTATATCATCAtTGAaTTCATGACCTACGGGAACCTC-3' (the T315I codon is underlined) and subsequent cloning of the amplified fragment into the wild type vector using the BlpI restriction enzyme. The integrin β3 construct was cloned, as described [2].

KCL-22 and KCL-22^{T315I} cells were a kind gift from Dr. Vignir Helgason. K562 and K562^{T315I} cells were generated as described [6]. All suspension cell lines were cultured in RPMI medium supplemented with 10% fetal calf serum (FCS), 1% penicillin/streptomycin and 1% L-Glutamine.

Drug treatment

In the CML model mice were treated with bovine fibronectin (Thermo Scientific, Grand Island, NY) on days 9, 10 and 12 (200 μg/dose) after transplantation for the intravenous treatments. This dose is equivalent to 10 mg/kg. For the intrafemoral experiment, the leukemia-initiating cells were resuspended in 40 µl of vehicle or fibronectin and intrafemorally injected. The mice were treated with two more intrafemoral doses of fibronectin on days 1 and 2 (50 μg/dose). In the AML model mice were treated with five doses of 200 μg/dose fibronectin on days 20, 22, 24, 26, 28, while in the B-ALL model the same dose of fibronectin was administered on days 10, 11, 12, 14 and 15. Ponatinib (Selleckchem, Munich, Germany), resuspended in citrate buffer [7], was given by oral gavage at 20 mg/kg (days 9-22 daily)[3,4], while Cpd22 (Merck Millipore, Darmstadt, Germany), resuspended in methylcellulose [8], was given intraperitoneally (i.p.) at 15 mg/kg i.p. (days 9-14 daily and days 14-22 every other day for Cpd22), respectively[5]. Poly I:C i.p. was administered to fibronectin fl/fl Mx1-Cre mice at a dose of 10 mg/kg i.p. [9] on transplantant days

1, 2, 3 and 5 or days 8, 9 and 10, and Col1a2-Cre mice were given 2 mg/mouse tamoxifen i.p. on transplantant days 1, 2, 3 and 5 (beginning two days after transplantation).

In vitro treatment of primary BCR-ABL1^{+} LKS cells occurred with 10 μM imatinib [10,11] for 4 hours prior to injection and *in vivo* microscopy analysis. For *in vitro* assays, BA/F3 cells expressing MSCV empty vector, BCR-ABL1, BCR-ABL1^{Y253F} or BCR-ABL1^{T315} were treated for 6 hours with different compounds that inhibit signaling pathways downstream of BCR-ABL1 at the following concentrations: Imatinib (Enzo Life Sciences, ALX-270-492, Farmingdale, NY) at 750 nM [12], wortmannin (PI3K inhibitor, Selleckchem, S2758) at 40 nM[13], ponatinib (Selleckchem, Munich, Germany) at 60 nM[14] and Cpd22 (Merck Millipore, Darmstadt, Germany) at 50 nM[15].

Southern blotting

Genomic DNA from spleen at the time of death was digested with the restriction enzyme BglII, run on an agarose gel and transferred onto a nylon membrane. The blot was then hybridized with a radioactively labeled probe targeting the GFP gene, in order to detect distinct proviral integration sites, as described [1].

In vivo microscopy

Unirradiated Col2.3kb-GFP reporter mice (GFP expression is under the control of the Col2.3kb promoter labeling osteoblastic cells) were injected with 2,000-10,000 sorted BCR-ABL1 or BCR-ABL1^{T315+} LKS or LKS SLAM cells labeled with DiD (Thermo Fisher Scientific, Grand Island, NY) for 15 minutes at 37° C, as described [16]. The mice were anesthesized by xylene/ketamine and the scalp removed [16]. The calvarium was visualized under a custom-built confocal two-photon hybrid in vivo microscope and images were analyzed with ImageJ software, as described [16].

Adhesion assay

Adhesion to fibronectin was performed according to the manufacturer's instructions (Cell Biolabs Inc., San Diego, CA). In brief, 1.5 x 10^5 $CD11b^+$ BCR-$ABL1^+$ or BCR-$ABL1^{T315I+}$ (GFP^+) myeloid cells per fibronectin-coated well of a 24-well plate were allowed to adhere for 72 hours. After vigorous washing with PBS cells were stained with the supplied staining solution and after washing with water extracted with the supplied extraction solution. Adhesion was measured as OD560 in a spectrophotometer. For the adhesion to MS-5 cells 1.5 x 10^5 $CD11b^+$ BCR-$ABL1^+$ or BCR-$ABL1^{T315I+}$ (GFP^+) myeloid cells or 2 x 10^5 K562 and $K562^{T315I}$ cells adhered to a confluent layer of MS-5 cells for 72 or 16 hours, respectively. Adhered cells were quantified by flow cytometry.

Transwell migration assay

10^5 BCR-$ABL1^+$ or BCR-$ABL1^{T315I+}$ BA/F3 cells were plated in the upper chamber of a transwell assay (Corning, Wiesbaden, Germany) in medium with 10 % fetal bovine serum and allowed to migrate through a membrane (5 μm pores for BA/F3 cells and 8 μm pores for K562 cells) for 8 hours towards a lower chamber, in which 3 x 10^4 MS-5 cells had previously been plated. Migrated cells were quantified by flow cytometry.

Immunofluorescence

Transduced 3T3 fibroblasts were allowed to adhere and grow on round 15 mm diameter coverslips for at least 24 hours. Non-adherent BA/F3 or bone marrow cells were cytospun on poly-L-lysine-coated slides (Thermo Fisher Scientific, Grand Island, New York, USA). Cells were fixed in 4% paraformaldehyde (Morphisto, Frankfurt, Germany) for 10 min at room temperature. Paraformaldehyde-fixed cells were permeabilized with 0.25% Triton in PBS for 5 minutes. Prior to incubation with primary antibodies cells were blocked in 2% BSA in PBS for 10 minutes at room temperature. Primary antibodies were generally diluted 1:100 in 2 %

BSA/PBS and cells were incubated for 1 h at room temperature. After two washing steps in PBS, the cells were incubated with fluorophore-labeled secondary antibodies (diluted 1:300) overnight. Slides were then washed with PBS and stained with a secondary antibody (Alexa Fluor^{TM} 488/555/647, Thermofisher, Waltham, Massachusetts, USA) for 2 hours followed by a counterstain for nuclei with 5 μg/ml 4′,6-Diamidin-2-phenylindol (DAPI) (Merck, Darmstadt, Germany). Cells were washed in PBS and briefly in water and mounted with FluorMount plus 50 μg/ml DABCO (Sigma-Aldrich, Munich, Germany). Specimens were analyzed using a confocal laser scanning microscope (Leica SP5, Wetzlar, Germany), and pictures were managed with ImageJ.

Mass spectrometry

BA/F3 cells expressing empty vector control, BCR-ABL1 or BCR-ABL1^{T315} were labeled with light (Lys +0/Arg +0), medium (Lys +4/Arg +6) and heavy (Lys +8/Arg +10) SILAC amino acids (Cambridge Isotopes, Tewksbury, USA), respectively. Cell lysis, protein purification and digestion as well as mass spectrometry were performed as previously described [17].

Quantitative Real-Time PCR

Quantitative PCR was performed using standard protocols and primers as listed in Supplementary Table 2.

Histopathology and staining procedures

Bones were decalcified with 0.5 M EDTA for 24 hours. The EDTA was changed the following day and bones were kept in EDTA for 5 days until mounting in paraffin. Bones were sectioned and stained with hematoxylin & eosin. Immunohistochemistry was performed according to standard protocols using an antibody to fibronectin (Abcam, Catalogue #ab2413, Berlin,

Germany) or an IgG isotype control (Cell Signaling Technology, Catalogue #2729S, Frankfurt am Main, Germany).

70,000 total bone marrow cells or 50,000 sorted GFP^+ $CD11b^+$ cells were cytospun for 300 rpm for 3 minutes on poly-L-lysine-coated slides. Cells were washed, air dried and stained with Giemsa according to standard protocols. Peripheral blood was smeared on a glass slide and, once dried, stained with Giemsa.

Staining with myeloperoxidase

10,000 total bone marrow cells or 10,000 sorted $CD11b^+$ cells were centrifuged in 96-well-plates at 500 rpm for 5 minutes. Cells were washed with PBS. Samples were processed according to the manufacturer's instructions and stained with myeloperoxidase (391A Peroxidase (Myeloperoxidase) Leukocyte Kit, Sigma Aldrich/Merck KGaA, Darmstadt, Germany).

Inducible knockdown of Ilk

For the inducible knockdown of *Ilk* lentiviri expressing the SMARTvector Inducible Lentiviral shRNA system and non-target control (Horizon Discovery/Dharmacon, Cambridge, UK) were prepared. For the *in vitro* induction of the shRNA 10 µg/ml doxycycline[18] was added to the culture of infected 3T3 cells and knockdown efficiency tested using qPCR. For *in vivo* experiments 50 mg/kg of doxycycline[19] was injected intraperitoneally into recipient mice on days 8, 9, 10 and 12 post transplantation.

Immunoblotting and coimmunoprecipitation

Cells were lysed in RIPA buffer (50 mM Tris HCl pH 7.4, 150 mM NaCl, 1% Triton X-100, 1% Na DOC, 0.1% SDS, 1 mM EDTA) supplemented with protease and phosphatase inhibitor cocktail (Sigma-Aldrich, Taufkirchen, Germany). Spleen cells were snap frozen, centrifuged and lysed in RIPA buffer as above. Lysates were kept on ice for 15 minutes and spun for 20 minutes

at 15,000 x g, quantified, denatured for 5 minutes at 94°C with SDS-Laemmli buffer and loaded on pre-cast 4-12% Bis-Tris polyacrylamide gels (Thermo Fisher Scientific, Darmstadt, Germany). Proteins were blotted on nitrocellulose membranes using the wet transfer method in presence of 10% methanol. Membranes were blocked in 5% milk in TBS-T and incubated with primary antibodies overnight at 4°C. After incubation with secondary antibodies, membranes were thoroughly washed with TBS-T and developed. Antibodies are listed in Supplementary Table 1. For the coimmunoprecipitation experiments proteins were isolated using RIPA buffer (150 mM sodium chloride, 1.0% Triton X-100, 0.5% sodium deoxycholate, 0.1% SDS, 50 mM Tris, pH 8.0) and magnetic beads (Dynabeads Protein G). Protein lysates were first incubated in 2 µg of antibody for 3 hours at 4°C and then incubated with magnetic beads overnight at 4 °C. After washing three times with ice cold lysis buffer immunoprecipitated proteins were mixed with 5x Laemmli elution buffer, heated to 95 °C for 4 minutes and eluted. The eluted proteins were separated using SDS-PAGE (4-12 % gradient) and blotted onto nitrocellulose membranes. Membranes were incubated overnight in the respective primary antibodies at 4 °C and then in secondary antibodies for 1 hour at room temperature.

Bioparticle phagocytosis assay

Bone marrow cells were collected after flushing of bones, followed by RBC lysis. 2 x 10^5 cells were cultured for 1 hour in RPMI. 1µg (2 mg/ml stock) of bioparticles (Thermo Fischer Scientific, Darmstadt, Germany) was added and incubated with the cells for 90 to 120 minutes. Consequently, the cells were washed with PBS and incubated with an anti-Gr-1 antibody before analysis by flow cytometry.

Single-molecule localization microscopy

Super-resolution imaging was performed using a custom-built wide-field microscope in total internal reflection fluorescence (TIRF) mode. A 638 nm laser (180 mW, LBX-638-180, Oxxius,

France) was combined with a 405 nm laser (50 mW, LBX-405-50-CSB-PP, Oxxius) by a dichroic mirror (LaserMUX 427-25, AHF Analysentechnik AG, Germany) and coupled into an inverted microscope (IX71, Olympus, Japan) equipped with a 100x oil immersion objective (PlanApo, NA 1.45, Olympus) and a nose piece to avoid drift. Fluorescence emission was collected by the objective and separated from excitation light with a dichroic mirror (HC Quad 410/504/582/669, AHF Analysentechnik AG) and a bandpass filter (700/75, AHF Analysentechnik AG). Fluorescence was detected with an EMCCD camera (iXon3, Andor).

For direct stochastic optical reconstruction microscopy (dSTORM) a special buffer was used for photoswitching of Alexa Fluor 647. The buffer consisted of 100 mM β-mercaptoethylamine and an oxygen scavenger system with 2.5 mM protocatechuic acid (cat. 03930590-50MG) and 10 nM protocatechuate-3,4-dioxygenase (cat. P8279-25UN) in Dulbecco's phosphate buffered saline (cat. D1408) (pH adjusted to 7.8 with 1 M NaOH) (all chemicals were purchased from Sigma-Aldrich, USA).

Super-resolution microscopy was performed in TIRF mode. Movies were recorded with 10,000-15,000 frame and a frame rate of 20 Hz under continuous 638 nm laser illumination (0.9 kW/cm^2). Fluorescence was recovered by the 405 nm laser (0-3 W/cm^2). Single-molecule localizations were obtained by fitting single-molecule point spread functions with two-dimensional Gaussian distributions using rapidSTORM [20]. The localization list was further analyzed with respect to clusters using the DBSCAN algorithm [21] in the localization microscopy analysis software LAMA [22]. Cell areas for calculation of cluster densities were determined using Fiji [23].

Patient CML cells

Cryopreserved unsorted cells from bone marrow or peripheral blood and bone sections from patients with BCR-ABL1$^+$ or BCR-ABL1T315I$^+$ CML were obtained from the University Center

for Tumoral Diseases (UCT) at the Goethe University Frankfurt, as approved of by the Ethics Committee (Approval number 319/15).

References

1. Krause DS, Fulzele K, Catic A, et al. Differential regulation of myeloid leukemias by the bone marrow microenvironment. *Nature Medicine*. 2013;19(11):1513-1517.
2. Verma D, Kumar R, Pereira R, et al. Vitamin K-antagonism impairs the bone marrow microenvironment and hematopoiesis *Blood*. 2019;134(3):227-238.
3. Ren M, Qin H, Ren R, Cowell JK. Ponatinib suppresses the development of myeloid and lymphoid malignancies associated with FGFR1 abnormalities. *Leukemia*. 2013;27(1):32-40.
4. Dubrovsky L, Pankov D, Brea EJ, et al. A TCR-mimic antibody to WT1 bypasses tyrosine kinase inhibitor resistance in human BCR-ABL+ leukemias. *Blood*. 2014;123(21):3296-3304.
5. Kudryashova TV, Goncharov DA, Pena A, et al. HIPPO-Integrin-linked Kinase Cross-Talk Controls Self-Sustaining Proliferation and Survival in Pulmonary Hypertension. *Am J Respir Crit Care Med*. 2016;194(7):866-877.
6. Tamai M, Inukai T, Kojika S, et al. T315I mutation of BCR-ABL1 into human Philadelphia chromosome-positive leukemia cell lines by homologous recombination using the CRISPR/Cas9 system. *Sci Rep*. 2018; 8(1):9966.
7. Paez-Mayorga J, Chen AL, Kotla S, et al. Ponatinib Activates an Inflammatory Response in Endothelial Cells via ERK5 SUMOylation. *Front Cardiovasc Med*. 2018;Sep 6(5):125.
8. Muise ES, Zhu Y, Verras A, et al. Identification and characterization of sebaceous gland atrophy-sparing DGAT1 inhibitors. *PLoS One*. 2014;Feb 18(9):2.
9. Mancini A, Niemann-Seyde SC, Pankow R, et al. THOC5/FMIP, an mRNA export TREX complex protein, is essential for hematopoietic primitive cell survival in vivo. *BMC Biol*. 2010;8(1).
10. Graham SM, Jorgensen HG, Allan E, et al. Primitive, quiescent, Philadelphia-positive stem cells from patients with chronic myeloid leukemia are insensitive to STI571 in vitro. *Blood*. 2002;99(1):319-325.
11. Chu S, Xu H, Shah NP, et al. Detection of BCR-ABL kinase mutations in CD34+ cells from chronic myelogenous leukemia patients in complete cytogenetic remission on imatinib mesylate treatment. *Blood*. 2005;105(5):2093-2098.
12. O'Hare T, Walters DK, Stoffregen EP, et al. In vitro activity of Bcr-Abl inhibitors AMN107 and BMS-354825 against clinically relevant imatinib-resistant Abl kinase domain mutants. *Cancer Res*. 2005;65(11):4500-4505.
13. Karnitz LM, Burns LA, Sutor SL, Blenis J, Abraham RT. Interleukin-2 triggers a novel phosphatidylinositol 3-kinase-dependent MEK activation pathway. *Mol Cell Biol*. 1995;15(6):3049-3057.
14. Gozgit JM, Chen TH, Song Y, et al. RET fusions observed in lung and colorectal cancers are sensitive to ponatinib. *Oncotarget*. 2018;9(51):29654-29664.
15. de la Puente P, Weisberg E, Muz B, et al. Identification of ILK as a novel therapeutic target for acute and chronic myeloid leukemia. *Leuk Res*. 2015;pii: S0145-2126(15):30377-30385.
16. Lo Celso C, Fleming HE, Wu JW, et al. Live-animal tracking of individual haematopoietic stem cells in their niche. *Nature*. 2009;457(7225):92-96.

17. Mohr S, Doebele C, Comoglio F, et al. Hoxa9 and Meis1 Cooperatively Induce Addiction to Syk Signaling by Suppressing miR-146a in Acute Myeloid Leukemia. *Cancer Cell*. Cancer Cell;31(4):549-562.
18. Luger AL, Sauer B, Lorenz NI, et al. Doxycycline Impairs Mitochondrial Function and Protects Human Glioma Cells from Hypoxia-Induced Cell Death: Implications of Using Tet-Inducible Systems. *Int J Mol Sci*. 2018;19(5):pii: E1504.
19. Walters BJ, Zuo J. A Sox10(rtTA/+) Mouse Line Allows for Inducible Gene Expression in the Auditory and Balance Organs of the Inner Ear. *J Assoc Res Otolaryngol*. 2015;16(3):331-345.
20. Wolter S, Schüttpelz M, Tscherepanow M, VAN DE Linde S, Heilemann M, Sauer M. Real-time computation of subdiffraction-resolution fluorescence images. *J Microsc*. 2010;237(1):12-22.
21. Ester M, Kriegel H-P, Sander J, X. X. A density-based algorithm for discovering clusters in large spatial databases with noise. *KDD'96 Proceedings of the Second International Conference on Knowledge Discovery and Data Mining*. 1996:226-231.
22. Malkusch S, Heilemann M. Extracting quantitative information from single-molecule super-resolution imaging data with LAMA - LocAlization Microscopy Analyzer. *Sci Rep*. 2016;Oct 5(6):34486.
23. Schindelin J, Arganda-Carreras I, Frise E, et al. Fiji: an open-source platform for biological-image analysis. *Nat Methods*. 2012;9(7):676-682.
