# Supplementary material for: Specific, targetable interactions with the microenvironment influence imatinib-resistant chronic myeloid leukemia
Source: Leukemia. 2020 May 21;34(8):2087–101. doi: 10.1038/s41375-020-0866-1 (PMC7387317; doi:10.1038/s41375-020-0866-1)
Supplement: Supplementary file 2 — Supplementary table 3 [file 41375_2020_866_MOESM2_ESM.pdf]

Supplementary Table 3: List of proteins and their normalized SILAC ratio (H/M); H=BCR-ABL1 T315I, M=BCR-ABL1

Protein names	Gene names	Normalized SILAC ratio (H/M)	Intensity
L-lactate dehydrogenase;L-lactate dehydrogenase B chain;L-lactate dehydrogenase A-like 6A	LDHB;LDHC;LDHAL6A	11,94186699	18142000
Protein SFI1 homolog	SFI1	9,146620324	16950000
Integrin beta;Integrin beta-3	ITGB3	5,156499768	103310000
ATP synthase mitochondrial F1 complex assembly factor 1	ATPAF1	4,048255202	134110000
NADH dehydrogenase [ubiquinone] 1 beta subcomplex subunit 10	NDUFB10	3,579866829	1250700000
Inositol hexakisphosphate and diphosphoinositol-pentakisphosphate kinase 1	PPIP5K1	2,517876926	11215000
Nuclear receptor subfamily 2 group C member 2	NR2C2	2,455675065	7670200
Zinc-alpha-2-glycoprotein	AZGP1	2,384301757	45410000
Coagulation factor V;Coagulation factor V heavy chain;Coagulation factor V light chain	F5	2,050987551	77196000
Protein-glutamine gamma-glutamyltransferase K	TGM1	2,042066571	27468000
Antigen peptide transporter 2	TAP2;TAP2-G	2,026424576	525610000
Pre-rRNA-processing protein TSR2 homolog	TSR2	1,986097319	68805000
Phosphoinositide 3-kinase adapter protein 1	PIK3AP1;FLJ00308	1,96858144	104440000
Pleckstrin	PLEK	1,955569462	1212200000
YY1-associated factor 2	YAF2	1,923816853	106620000
Ubiquitin thioesterase OTU1	YOD1	1,875152356	72977000
Bromodomain-containing protein 2	BRD2	1,860845941	81180000
Twisted gastrulation protein homolog 1	TWSG1	1,860119048	44041000
Ubiquitin-conjugating enzyme E2 J2	UBE2J2	1,852194851	29026000
Histone H1.2	HIST1H1C	1,849762306	35773000
Desmocollin-1	DSC1	1,837863667	49583000
Zinc finger SWIM domain-containing protein 8	ZSWIM8	1,812513594	168200000
Putative oxidoreductase GLYR1	GLYR1;N-PAC	1,793754148	35852000
E3 SUMO-protein ligase PIAS1	PIAS1	1,787086513	14509000
Sjoegren syndrome nuclear autoantigen 1	SSNA1	1,781546739	209040000
Adenylosuccinate synthetase isozyme 1	ADSSL1	1,775252974	120450000
BCL2/adenovirus E1B 19 kDa protein-interacting protein 2;Caytaxin	BNIP2;ATCAY	1,765443215	19942000
Src-like-adapter 2	SLA2	1,761897211	62108000
Syntaxin-16	STX16;STX16-NPEPL1	1,736442723	35278000
Histone H1.0;Histone H1.0, N-terminally processed	H1F0	1,731271966	3035000000
Protein Churchill	CHURC1;CHURC1-FNTB	1,71188907	87964000
RING1 and YY1-binding protein	RYBP	1,700477834	162680000
Selenoprotein H	C11orf31;SELH	1,69511637	38867000
Ubiquitin-conjugating enzyme E2 C	UBE2C	1,692906721	111530000
Uncharacterized protein C20orf24	C20orf24	1,682085786	29698000
Histone H3.1	HIST1H3A	1,678331068	1530600000
Engulfment and cell motility protein 2	ELMO2	1,673080141	305660000
E3 ubiquitin-protein ligase RNF115	RNF115	1,670704202	94228000
Golgi-associated plant pathogenesis-related protein 1	C9orf19;GLIPR2	1,663174001	284680000
Histone H1.4;Histone H1.3	HIST1H1E;HIST1H1D	1,654122901	47559000000
Protein FAM32A	FAM32A	1,652182533	161950000
Protein CBFA2T3;Protein CBFA2T2;Protein CBFA2T1	CBFA2T3;RUNX1T1;MTG8;CBFA2	1,645738361	22290000
Eukaryotic translation initiation factor 4E type 2	tmp_locus_9;EIF4E2	1,634334091	336960000
Ubiquitin-associated and SH3 domain-containing protein B	UBASH3B	1,63369329	135510000
Cytochrome c oxidase subunit 1	COX1;coxI;COI;CO1;cox1;MT-CO1	1,632892997	66901000
Histone H3;Histone H3.3;Histone H3.1t;Histone H3.3C	H3F3B;H3F3A;HIST3H3;H3F3C	1,621271077	695460000
ETS-related transcription factor Elf-1	DKFZp686H0575;ELF1	1,61736402	85879000
		1,608829255	20715000
Tax1-binding protein 3	TAX1BP3	1,606787069	71186000
Cytoplasmic tRNA 2-thiolation protein 1	CTU1	1,606322485	70039000
Protein dopey-1	DOPEY1	1,599309098	13349000
Protein S100-A8;Protein S100-A8, N-terminally processed	S100A8	1,599155646	202710000
Caspase-6;Caspase-6 subunit p18;Caspase-6 subunit p11	CASP6	1,59854213	620080000
Cytochrome c oxidase subunit NDUFA4	NDUFA4	1,595838054	1278800000
60S ribosomal protein L7-like 1	RPL7L1	1,594133588	77575000
Histone H1.5	HIST1H1B	1,587780442	11585000000
Integrin alpha-IIb;Integrin alpha-IIb heavy chain;Integrin alpha-IIb light chain, form 1;Integrin alpha-IIb light chain, form 2	ITGA2B	1,586520918	344370000
Putative uncharacterized protein C10orf115	C10orf115	1,583556351	6279700000
Eukaryotic translation initiation factor 1	SUI1;EIF1	1,58042798	6271200000
Nucleolar protein 9	DKFZp686E1893;NOP9	1,574976769	9894900
		1,568602845	75846000
Testis-expressed sequence 30 protein	TEX30	1,565704802	466970000
Mortality factor 4-like protein 1	MORF4L1	1,545523391	194770000
Poly(A) polymerase gamma	PAPOLG	1,544544668	41533000
Proline-serine-threonine phosphatase-interacting protein 2	PSTPIP2	1,539361473	53213000
Sestrin-2	SESN2	1,538508877	39995000
Ubiquitin-conjugating enzyme E2 B	UBE2B	1,534330648	171910000
Poly(rC)-binding protein 2	PCBP2	1,534212949	95194000
Glutaminase kidney isoform, mitochondrial	GLS	1,533601202	253420000
Mitochondrial folate transporter/carrier	SLC25A32	1,530713772	351410000
Transcription initiation factor TFIID subunit 8	TAF8	1,529168897	25587000
Transmembrane emp24 domain-containing protein 1	TMED1	1,528491074	29101000
ATP-binding cassette sub-family B member 6, mitochondrial	ABCB6	1,524088214	13252000
Leupaxin	LPXN	1,517312536	184210000
Copine-2	CPNE2	1,512859304	1246500000
Cyclin-dependent kinase 1	DKFZp686L20222;CDK1;CDC2	1,511281718	3209800000
Dyslexia-associated protein KIAA0319-like protein	KIAA0319L	1,510437121	12667000
		1,507567991	9557800
Transferrin receptor protein 1;Transferrin receptor protein 1, serum form	TFRC	1,506046778	353770000
Eukaryotic translation initiation factor 1b	GC20;EIF1B	1,502245858	15558000000
Chromatin accessibility complex protein 1	CHRAC1	1,498823424	45482000
LIM domain kinase 2	LIMK2	1,495550737	689410000
High mobility group nucleosome-binding domain-containing protein 3;Non-histone chromosomal protein HMG-17	HMGN3;HMGN2	1,495192955	614460000
	CLUH	1,494075989	236780000
DNA-directed RNA polymerase III subunit RPC6	POLR3F	1,493942065	168940000
E3 SUMO-protein ligase CBX4	CBX4	1,490846204	63119000
Spindlin-1	SPIN1	1,4803849	185740000
FGFR1 oncogene partner	FGFR1OP	1,478983642	61359000
Ubiquitin-conjugating enzyme E2 E1	UBE2E1	1,474165254	316490000
Transmembrane emp24 domain-containing protein 3	TMED3	1,471930289	231020000
Ras-related protein Rap-1b;Ras-related protein Rap-1b-like protein	RAP1B	1,470501735	45879000000
Enhancer of mRNA-decapping protein 3	EDC3	1,469486121	29145000
Heat shock-related 70 kDa protein 2	HSPA2	1,468428781	68905000
Importin subunit alpha;Importin subunit alpha-1	KPNA2;POU2F2	1,462480074	254790000
PDZ and LIM domain protein 7	PDLIM7	1,46226622	82309000
Dual specificity protein phosphatase 12	DUSP12	1,45902333	98399000
Ribosome biogenesis protein BRX1 homolog	BRIX1;BXDC2	1,456197577	94478000
Ubiquitin-conjugating enzyme E2 R2	UBE2R2	1,455921963	542370000
Large proline-rich protein BAG6	BAG6	1,455921963	3479100000
Target of rapamycin complex subunit LST8	MLST8	1,450852376	159560000
AT-rich interactive domain-containing protein 3A	ARID3A	1,450052927	84774000
Probable ribosome biogenesis protein RLP24	RSL24D1	1,44917035	114660000
Tetratricopeptide repeat protein 4	TTC4	1,445274674	93970000
Transcriptional repressor p66-beta	GATAD2B	1,444126737	525580000
G-protein coupled receptor 56;GPR56 N-terminal fragment;GPR56 C-terminal fragment	GPR56	1,442668359	100990000
UPF0547 protein C16orf87	C16orf87	1,438310848	23379000

Myotubularin-related protein 3	MTMR3	1,438269474	68575000
Rho guanine nucleotide exchange factor 40	FLJ10357;ARHGEF40	1,437814522	23455000
Muscleblind-like protein 1;Muscleblind-like protein 2	MBNL1;MBLL;MBNL2	1,435214421	381000000
Prostaglandin G/H synthase 1	PTGS1	1,433116455	775480000
Nuclear receptor-binding factor 2	NRBF2	1,432870039	41353000
NEDD4 family-interacting protein 1	NDFIP1	1,43006278	68606000
Transcriptional repressor p66-alpha	GATAD2A	1,429572129	104080000
Sorting nexin-20	SNX20	1,425252626	68198000
	MUC19	1,424887078	156350000
Bromodomain-containing protein 3	BRD3	1,42454201	51821000
Serine/threonine-protein kinase tousled-like 1	TLK1	1,421807686	9385900
	PCBP2	1,420575617	272460000
Golgi phosphoprotein 3-like	GOLPH3L	1,418238548	53122000
Ras-related protein Rab-6A	RAB6A	1,413927183	156850000
DNA-directed RNA polymerases I and III subunit RPAC2	POLR1D	1,413068053	86733000
Protein NATD1	NATD1	1,409204927	163320000
RNA-binding protein 5	RBM5	1,409105641	156540000
Caprin-1	CAPRIN1	1,40637086	1859300000
Putative ataxin-7-like protein 3B	ATXN7L3B	1,405204879	288740000
GA-binding protein subunit beta-2;GA-binding protein subunit beta-1	GABPB2;GABPB1	1,399129741	58774000
Ral GTPase-activating protein subunit alpha-1	RALGAPA1	1,399090591	37906000
Cytochrome c oxidase subunit 5B, mitochondrial	COX5B	1,397624039	1358400000
LanC-like protein 2	LANCL2	1,396823623	241580000
Mitogen-activated protein kinase;Serine/threonine-protein kinase NLK	NLK	1,395809779	91708000
Pseudopodium-enriched atypical kinase 1	PEAK1	1,393320422	812500000
Nucleolar GTP-binding protein 1	GTPBP4	1,392621889	533650000
Guanine nucleotide-binding protein G(I)/G(S)/G(O) subunit gamma-2;Guanine nucleotide-binding protein subunit gamma	GNG2	1,392486145	159870000
Nischarin	NISCH	1,391459223	477500000
PDZ and LIM domain protein 2	PDLIM2	1,390047262	85181000
CD2 antigen cytoplasmic tail-binding protein 2	CD2BP2	1,386827909	119500000
Nucleoporin NUP53	NUP35	1,386289596	1184800000
Selenide, water dikinase 2	SEPHS2	1,384734685	217930000
Tubulin-specific chaperone cofactor E-like protein	TBCEL	1,384542962	254920000
Huntingtin-interacting protein 1-related protein	HIP1R	1,384523793	70443000
Arachidonate 5-lipoxygenase-activating protein	ALOX5AP	1,382361073	1560700000
Bromodomain adjacent to zinc finger domain protein 1A	BAZ1A	1,3821318	46905000
Cysteine-rich PDZ-binding protein	CRIPT	1,380948435	29217000
Casein kinase I isoform delta	CSNK1D;CSNK1E	1,380452789	585220000
Ubiquitin-associated protein 2	UBAP2	1,374778317	312110000
TSC22 domain family protein 1	TSC22D1	1,374003847	24418000
Cytochrome c oxidase assembly protein COX15 homolog	COX15	1,372532872	191240000
Serine/threonine-protein kinase RIO1	RIOK1	1,371384687	29480000
Acetyl-coenzyme A synthetase;Acetyl-coenzyme A synthetase, cytoplasmic	ACSS2;DKFZp762G026	1,371046245	513300000
Protein DDI1 homolog 2	DDI2	1,370163323	1145900000
Ubiquitin-conjugating enzyme E2 R1	CDC34	1,370013152	208230000
Probable RNA-binding protein EIF1AD	EIF1AD	1,369112815	2078800000
Metallo-beta-lactamase domain-containing protein 1	MBLAC1	1,364312318	66715000
Protein scribble homolog	SCRIB	1,363735544	61626000
Ubiquitin-conjugating enzyme E2 Q1	GTAP;UBE2Q1	1,36293631	221170000
Origin recognition complex subunit 5	ORC5L;ORC5	1,361303584	116980000
Secretory carrier-associated membrane protein 2	SCAMP2	1,358105172	380460000
Protein kinase C-binding protein 1	ZMYND8	1,356557599	17418000
Ras GTPase-activating protein-binding protein 1	G3BP;DKFZp686L1159;G3BP1	1,355197181	2308700000
NEDD8-conjugating enzyme UBE2F	UBE2F	1,354628087	256300000
Nuclear pore complex protein Nup98-Nup96;Nuclear pore complex protein Nup98;Nuclear pore complex protein Nup96	NUP98	1,353216596	354770000
Pre-mRNA-splicing factor ISY1 homolog	ISY1	1,351972528	325040000
DNA/RNA-binding protein KIN17	KIN	1,351406138	324060000
Guanine nucleotide-binding protein-like 3	GNL3	1,350438893	123740000
Zinc finger and BTB domain-containing protein 7A	ZBTB7A	1,349910231	58028000
Nucleolar GTP-binding protein 2	GNL2	1,349746248	229580000
Ubiquitin-like protein 3	UBL3	1,346837625	34963000
Nuclear pore complex protein Nup153	NUP153	1,346021832	799620000
RNA-binding protein 38;RNA-binding protein 24	RBM38;RNPC1;RBM24	1,344158288	823740000
D-glucuronyl C5-epimerase	GLCE	1,343435972	72244000
Translation factor GUF1, mitochondrial	GUF1	1,342822613	20109000
Glutathione peroxidase;Phospholipid hydroperoxide glutathione peroxidase, mitochondrial	GPX4	1,341381623	1399300000
Rho GTPase-activating protein 6	ARHGAP6	1,341147754	227090000
Protein SDA1 homolog	SDAD1	1,337560023	244340000
Lamin-B1	LMNB1	1,337452688	3180600000
		1,336094595	21096000
Deoxycytidylate deaminase	DCTD	1,335898258	137040000
TBC1 domain family member 25	TBC1D25;MG81;OATL1	1,335291761	25389000
Sperm-associated antigen 7	SPAG7	1,334454275	462830000
WW domain-containing adapter protein with coiled-coil	WAC	1,333262226	60220000
Origin recognition complex subunit 2	ORC2	1,333120034	31104000
5-AMP-activated protein kinase subunit gamma-1	PRKAG1	1,332906803	495130000
Glucocorticoid receptor	NR3C1	1,332889037	74632000
Glutamate-rich WD repeat-containing protein 1	DKFZp564C172;GRWD1	1,331203408	134540000
rRNA-processing protein UTP23 homolog	UTP23	1,331114809	72047000
Glucose 1,6-bisphosphate synthase	PGM2L1	1,330849082	812390000
Casein kinase II subunit alpha	CSNK2A2	1,330618871	2012400000
Pre-mRNA-splicing regulator WTAP	WTAP;DKFZp686F20131	1,33010561	144510000
WAS protein family homolog 6;Putative WAS protein family homolog 3;WAS protein family homolog 2;WAS protein family hom	FLJ00075;WASH4P;DKFZp434K13	1,328921315	49395000
Stromal membrane-associated protein 2	SMAP2	1,328585853	315220000
Guanine nucleotide-binding protein-like 3-like protein	GNL3L	1,327457123	94813000
DNA excision repair protein ERCC-1	ERCC1	1,326488652	59541000
Ubiquitin-associated protein 2-like	UBAP2L	1,32596099	5265500000
Nuclear transcription factor Y subunit gamma	NFYC	1,325732467	1227500000
Selenoprotein S	VIMP;SELS	1,324924479	112400000
Tumor necrosis factor alpha-induced protein 8	TNFAIP8	1,324643671	244690000
Catenin alpha-1	CTNNA1	1,324538398	987690000
WD repeat-containing protein 48	WDR48	1,323661778	59127000
MOB kinase activator 1A	MOB1A	1,322436457	7590800000
Serine-protein kinase ATM	ATM;DKFZp781A0353	1,321999392	143780000
Neutrophil cytosol factor 4	NCF4	1,320829481	114810000
Prelamin-A/C;Lamin-A/C	LMNA	1,320445782	4040300000
Gigaxonin	GAN	1,320166869	28813000
Survival of motor neuron-related-splicing factor 30	SMNDC1	1,319278618	2573700000
Beta-arrestin-2	ARRB2	1,318774068	241170000
Vimentin	VIM	1,316915783	7523300000
Interferon regulatory factor 2-binding protein 2	IRF2BP2	1,316343724	232720000
Uridine phosphorylase 1	UPP1	1,316343724	249690000
Tax1-binding protein 1	TAX1BP1	1,315927992	411140000
Diphthine synthase	DPH5	1,315599058	328320000
Prolyl 3-hydroxylase 1	LEPRE1;DKFZp547C166	1,3151665	18465000
Exosome component 10	EXOSC10	1,313404607	505260000
Receptor-type tyrosine-protein phosphatase eta	PTPRJ	1,313283866	44824000
Putative beta-actin-like protein 3	POTEKP	1,313232127	774470000

Zinc finger protein ZFPM1	ZFPM1	1,312921776	203840000
Sulfatase-modifying factor 2	SUMF2;DKFZP566I1024	1,312577114	1574400000
Protein FAM195B	FAM195B	1,311957178	221250000
BUB3-interacting and GLEBS motif-containing protein ZNF207	ZNF207;DKFZp761N202	1,311819494	1901000000
Mothers against decapentaplegic homolog 2;Mothers against decapentaplegic homolog 3;Mothers against decapentaplegic h	SMAD2;SMAD3;SMAD9	1,310684702	178750000
Mini-chromosome maintenance complex-binding protein	MCMBP	1,310581636	613820000
Basic leucine zipper and W2 domain-containing protein 1	BZW1;hCG_2022736	1,309431838	8446400000
Regulation of nuclear pre-mRNA domain-containing protein 2	RPRD2	1,308592216	20849000
		1,307856293	69011000
Roundabout homolog 4	ROBO4	1,307668166	5076700000
High mobility group protein 20A	HMG20A	1,303594009	66500000
Low-density lipoprotein receptor-related protein 12	LRP12	1,303220257	46570000
F-box-like/WD repeat-containing protein TBL1XR1	TBL1XR1	1,301981616	63923000
Phospholipid scramblase 1	PLSCR1	1,301066875	1867300000
Caspase-14;Caspase-14 subunit p19;Caspase-14 subunit p10	CASP14	1,300965316	42239000
tRNA-specific adenosine deaminase 2	ADAT2	1,300170322	140470000
DNA topoisomerase 2-alpha	TOP2A	1,29947761	2875400000
Cytochrome c oxidase subunit 6A, mitochondrial		1,299173726	39265000
Eukaryotic translation initiation factor 4 gamma 1	EIF4G1	1,298667567	415230000
Geranylgeranyl pyrophosphate synthase	GGPS1	1,298650702	78819000
Chromobox protein homolog 5	CBX5	1,297706952	311720000
EH domain-containing protein 2	EHD2	1,297235591	87169000
CCR4-NOT transcription complex subunit 8	CNOT8	1,297117804	603510000
Cyclin-C	CCNC	1,296781389	84244000
Protein bicaudal D homolog 1	GLYAT;BICD1	1,296209882	15772000
Transmembrane and TPR repeat-containing protein 3	TMTC3	1,295756398	54422000
Alpha-tocopherol transfer protein-like	DKFZp686E0870;TTPAL	1,294381092	218790000
Uridine phosphorylase 2	UPP2	1,294330831	406630000
Ras GTPase-activating protein 2	RASA2	1,29406284	77774000
HEAT repeat-containing protein 6	HEATR6	1,291972972	91723000
rRNA/tRNA 2-O-methyltransferase fibrillarin-like protein 1	FBLL1	1,288493751	498230000
Catenin alpha-2	CTNNA2	1,288277959	80028000
Lethal(2) giant larvae protein homolog 1	LLGL1	1,288194981	53103000
	UNQ2523	1,288095422	423010000
TBC1 domain family member 5	TBC1D5	1,287929524	360570000
Plexin domain-containing protein 2	PLXDC2	1,286388721	244650000
Aladin	AAAS	1,284967169	38549000
mRNA turnover protein 4 homolog	MRTO4	1,284538016	4081900000
Copper homeostasis protein cutC homolog	CUTC	1,283812409	198980000
Niemann-Pick C1 protein	NPC1	1,283730006	41119000
SCY1-like protein 2	SCYL2	1,283614659	387500000
RNA-binding protein 42	RBM42	1,281049436	71630000
Ribonuclease H1	RNASEH1	1,280458916	32266000
Zyxin	ZYX	1,279918085	9448700
Calcium-regulated heat stable protein 1	CARHSP1	1,279787043	466170000
Conserved oligomeric Golgi complex subunit 2	COG2	1,278870502	99191000
Interferon regulatory factor 5	IRF5	1,276698967	16598000
Protein LTV1 homolog	LTV1	1,276275318	51192000
Leucine-rich repeat and calponin homology domain-containing protein 3	LRCH3	1,275363798	281970000
Regulator of chromosome condensation	RCC1	1,274242463	3346400000
Nck-associated protein 5-like	NCKAP5L	1,273739317	36277000
NAD-dependent protein deacetylase sirtuin-1;SirtT1 75 kDa fragment	SIRT1	1,273723093	125630000
Nitric oxide synthase-interacting protein	NOSIP	1,273674423	148590000
Nuclear respiratory factor 1	NRF1	1,273528438	39766000
Vitamin K epoxide reductase complex subunit 1	VKORC1	1,2724589	45424000
PERQ amino acid-rich with GYF domain-containing protein 1	GIGYF1	1,272086604	21779000
Ras-related protein Rab-9A	RAB9A	1,271779219	142310000
	EIF3G	1,271439651	242030000
Atrophin-1	ATN1	1,270664176	46679000
Protein IMPACT	IMPACT	1,270034799	82348000
Vesicle-associated membrane protein 8	VAMP8	1,269309369	1477900000
Crooked neck-like protein 1	crn;DKFZp762M013;CRNKL1	1,26890671	67026000
RanBP2-like and GRIP domain-containing protein 3;RANBP2-like and GRIP domain-containing protein 8	RGPD3;RGPD8	1,268649142	172590000
Disintegrin and metalloproteinase domain-containing protein 10	ADAM10	1,26860086	576400000
Nuclear transcription factor Y subunit beta	NFYB	1,268552582	284390000
Golgin subfamily A member 7	GOLGA7	1,268536489	396200000
Zinc finger protein 706	ZNF706	1,268391679	85531000
Pumilio homolog 1	PUM1	1,267346809	826320000
Histone H2A.V;Histone H2A.Z;Histone H2A	H2AFV;H2AFZ	1,26707382	7393400000
Exosome complex component CSL4	EXOSC1	1,266399878	505870000
Mitochondrial import inner membrane translocase subunit Tim22	TIMM22	1,265150173	569350000
Citron Rho-interacting kinase	CIT	1,265086152	56124000
Ubiquilin-4	UBQLN4	1,264830133	193000000
N(6)-adenine-specific DNA methyltransferase 2	N6AMT2	1,26459021	369940000
BMP-2-inducible protein kinase	BMP2K	1,264142595	44065000
Crk-like protein	CRKL	1,262833546	3770300000
DNA methyltransferase 1-associated protein 1	DMAP1	1,262737868	45225000
PERQ amino acid-rich with GYF domain-containing protein 2	GIGYF2	1,262275631	736740000
1-acylglycerol-3-phosphate O-acyltransferase ABHD5	ABHD5	1,261861498	145970000
Heterogeneous nuclear ribonucleoprotein D-like	HNRNPDL	1,26146355	941090000
Lanosterol 14-alpha demethylase	CYP51A1	1,261399902	36164000
AP-2 complex subunit alpha-2	AP2A2	1,260461833	33212000
Cytochrome c oxidase subunit 6B1	COX6B1	1,260398286	958790000
DnaJ homolog subfamily C member 5	DNAJC5;FLJ00095	1,260112402	860260000
MORC family CW-type zinc finger protein 3	MORC3	1,258304812	139690000
PDZ and LIM domain protein 5	PDLIM5	1,258130669	1367100000
Ribosome biogenesis protein WDR12	WDR12	1,258130669	1604300000
	ABCE1	1,25779835	109190000
Putative GTP cyclohydrolase 1 type 2;Putative GTP cyclohydrolase 1 type 2 NIF3L1	DKFZp762L015;NIF3L1	1,257339721	90402000
Polypeptide N-acetylgalactosaminyltransferase 6	GALNT6	1,257244874	102810000
		1,257197455	861030000
SRSF protein kinase 2;SRSF protein kinase 2 N-terminal;SRSF protein kinase 2 C-terminal	SRPK2	1,25710263	70458000
Elongation factor 1-alpha		1,257086827	25263000
Serine/threonine-protein kinase B-raf	BRAF	1,25629719	17886000
Ras-related protein Rab-11A	RAB11A	1,256028939	135250000
Polyadenylate-binding protein-interacting protein 2	PAIP2	1,255855426	119650000
Deoxyuridine 5-triphosphate nucleotidohydrolase, mitochondrial	DUT	1,255792342	4500200000
PAB-dependent poly(A)-specific ribonuclease subunit PAN2	PAN2	1,255697728	54439000
Set1/Ash2 histone methyltransferase complex subunit ASH2	ASH2L	1,255146099	184780000
Zinc transporter 1	SLC30A1	1,254594954	49329000
Bile salt export pump	ABCB11	1,253117129	19566000
Protein RRP5 homolog	PDCD11	1,252614833	21114000
Transcription initiation factor IIB	GTF2B	1,252520698	2457800000
Transcription initiation factor TFIID subunit 4	TAF4	1,251799462	120830000
RNA-binding protein EWS;Nuclear factor of activated T-cells, cytoplasmic 2	EWSR1/ZNF384 fusion;EWSR1;NF/	1,251235595	18373000
Replication protein A 14 kDa subunit	RPA3	1,24975005	271330000
Porphobilinogen deaminase	HMBS	1,248626511	528570000
Tyrosine-protein kinase Fes/Fps;Tyrosine-protein kinase	FES	1,248408279	53106000

Nipped-B-like protein	NIPBL	1,248314775	763330000
Tumor necrosis factor alpha-induced protein 8	TNFAIP8	1,247956471	4462100000
Ubiquitin-associated protein 2-like	UBAP2L	1,247894179	92751000
Angio-associated migratory cell protein	AAMP	1,247209369	565770000
La-related protein 4B	LARP4B	1,246976083	280800000
Neutrophil cytosol factor 1;Putative neutrophil cytosol factor 1B;Putative neutrophil cytosol factor 1C	NCF1;NCF1B;NCF1C	1,246913888	450970000
DNA excision repair protein ERCC-6-like	ERCC6L	1,246789517	21561000
Histone chaperone ASF1B	ASF1B	1,246742884	63657000
Interferon-related developmental regulator 1	IFRD1	1,24633888	338990000
SWI/SNF-related matrix-associated actin-dependent regulator of chromatin subfamily A member 5	SMARCA5	1,246276748	3616800000
Transcription and mRNA export factor ENY2	ENY2	1,245702327	458500000
Polycomb protein SUZ12	SUZ12	1,245454093	62001000
CUGBP Elav-like family member 1	CELF1	1,245050923	219240000
Protein IWS1 homolog	IWS1	1,244849435	275240000
Ubiquitin domain-containing protein UBFD1	UBFD1	1,244106048	447300000
AN1-type zinc finger protein 6	ZFAND6	1,243456311	128610000
Cytosolic phospholipase A2;Phospholipase A2;Lysophospholipase	PLA2G4A	1,243193516	7855800000
Guanine nucleotide-binding protein subunit gamma;Guanine nucleotide-binding protein G(I)/G(S)/G(O) subunit gamma-12	GNG12	1,243116244	206160000
Transmembrane protein 214	TMEM214;DKFZp761L1314	1,242853592	252920000
Eukaryotic translation initiation factor 2A;Eukaryotic translation initiation factor 2A, N-terminally processed	EIF2A	1,242482978	1119500000
Phospholipid-transporting ATPase IA	ATP8A1	1,242390359	28436000
2-oxoglutarate and iron-dependent oxygenase domain-containing protein 3	OGFOD3	1,241804093	76251000
Nuclear fragile X mental retardation-interacting protein 2	NUFIP2	1,241804093	167830000
Ubiquitin-like protein 5	UBL5	1,241033533	408430000
Bromodomain-containing protein 4	BRD4	1,240848741	533360000
Nuclear receptor coactivator 1	NCOA1	1,240433159	96191000
Metastasis-associated protein MTA2	MTA2	1,240217782	3509000000
DmX-like protein 2	DMXL2	1,240125501	101270000
Kinesin-like protein KIF15	KIF15	1,239587465	958340000
Cytochrome c oxidase subunit 7C, mitochondrial	COX7C	1,23954137	815800000
Protein HEXIM1	HEXIM1	1,237394048	322350000
	ACOT7	1,236690123	68242000
Peroxisomal biogenesis factor 19	PEX19	1,236628949	781350000
Ribonuclease H2 subunit B	RNASEH2B	1,236262038	927600000
C-terminal-binding protein 2	CTBP2	1,235391496	1017200000
Nucleoporin p58/p45	NUPL1	1,23496431	575870000
High affinity cationic amino acid transporter 1;Low affinity cationic amino acid transporter 2	SLC7A1;SLC7A2	1,234842311	711670000
RNA-binding protein 39	RBM39;DKFZp781C0423;DKFZp68	1,234308849	6419900000
Prolyl 3-hydroxylase OGFOD1	OGFOD1	1,233775848	141660000
Histone-binding protein RBBP7	RBBP7	1,233060827	6568200000
E1A-binding protein p400	EP400	1,233045623	70438000
Histone acetyltransferase p300;CREB-binding protein	EP300;CREBBP	1,232270705	746360000
Cold-inducible RNA-binding protein	CIRBP	1,231663608	123870000
F-box/LRR-repeat protein 15	FBXL15	1,230420927	136660000
	TARDBP	1,230042559	142930000
Methyl-CpG-binding domain protein 3	MBD3	1,229906404	673770000
Transcription factor BTF3	BTF3	1,229755156	21334000000
Importin subunit alpha-3	KPNA4	1,228727653	1336800000
BSD domain-containing protein 1	BSDC1	1,226602556	29095000
DNA-directed RNA polymerase II subunit RPB1	POLR2A	1,226467161	7202400000
BRO1 domain-containing protein BROX	BROX	1,225625375	1401700000
Serine/threonine-protein phosphatase 4 regulatory subunit 2	PPP4R2	1,225355047	95232000
Protein N-terminal asparagine amidohydrolase	NTAN1	1,224994794	46385000
Diphthine--ammonia ligase	DPH6	1,224619756	187320000
Protein dpy-30 homolog	DPY30;LOC84661	1,224140042	348070000
G patch domain-containing protein 8	GPATCH8	1,223795479	33370000
Ribonuclease;Ribonuclease H2 subunit A	RNASEH2A;JUNB	1,223166779	681040000
RNA-binding protein with multiple splicing 2	RBPMS2	1,222927444	215680000
Heterochromatin protein 1-binding protein 3	HP1BP3	1,222912488	995770000
ERO1-like protein beta	ERO1LB	1,222792859	116720000
ATP-dependent RNA helicase DDX18	DDX18	1,222404225	388080000
Nucleoporin p54	NUP54	1,222344457	1046800000
Cytochrome c oxidase subunit 7A-related protein, mitochondrial	COX7A2L	1,222254816	398540000
Cell division cycle protein 123 homolog	CDC123	1,221463558	300780000
Pumilio homolog 2	PUM2	1,220703125	248490000
Protein SCAF8	SCAF8;RBM16	1,220390281	690120000
E3 SUMO-protein ligase RanBP2	RANBP2	1,219794831	3220500000
Ras GTPase-activating protein 3	RASA3	1,219705563	24411000
F-box/LRR-repeat protein 20	FBXL20	1,219021613	97790000
Round spermatid basic protein 1-like protein	RSBN1L	1,218783897	124560000
Monocarboxylate transporter 1	SLC16A1	1,218769043	38344000
Ras-related protein Rab-32	RAB32	1,218427497	409870000
Serine/arginine-rich splicing factor 9	SRSF9	1,218130657	363000000
Microfibrillar-associated protein 1	MFAP1	1,218115818	154950000
Rho GTPase-activating protein 26	ARHGAP26	1,218041633	296760000
Probable ATP-dependent RNA helicase DHX37	DHX37	1,217982291	264040000
RAS protein activator like-3	FLJ00087;RASAL3	1,217166922	331600000
COBW domain-containing protein 5;COBW domain-containing protein 1;COBW domain-containing protein 3;COBW domain-c	CBWD3;CBWD5;CBWD2;CBWD6;C	1,217107665	156200000
PEST proteolytic signal-containing nuclear protein	PCNP	1,216811467	3685000000
Ubiquitin-conjugating enzyme E2 S	UBE2S	1,21618992	842830000
Thioredoxin reductase 2, mitochondrial	TXNRD2	1,215997665	27414000
Pumilio domain-containing protein KIAA0020	KIAA0020	1,215849818	31322000
Growth factor receptor-bound protein 7	GRB7	1,215820253	12801000
Nucleolar protein 58	NOP58	1,215687228	1532300000
NADH dehydrogenase [ubiquinone] 1 alpha subcomplex assembly factor 5	NDUFAF5	1,215170185	20444000
WD repeat and HMG-box DNA-binding protein 1	WDHD1	1,215170185	392740000
Estradiol 17-beta-dehydrogenase 11	HSD17B11	1,214624074	403100000
Cyclin-G-associated kinase	GAK	1,214063714	458050000
DNA topoisomerase 1	TOP1	1,213768995	4406800000
DNA-directed RNA polymerase II subunit RPB4	POLR2D	1,213680608	654940000
Endothelial differentiation-related factor 1	EDF1	1,213415522	3542700000
Histone H4	HIST1H4H;HIST1H4A	1,21297397	2,6944E+11
Coiled-coil domain-containing protein 12	CCDC12	1,212709192	212350000
Quinone oxidoreductase	CRYZ	1,212268154	1050100000
Tropomodulin-3	DKFZp686E1899;TMOD3	1,211783382	4031100000
RNA-binding motif, single-stranded-interacting protein 1;RNA-binding motif, single-stranded-interacting protein 3	RBMS1;RBMS3	1,211269653	275330000
Phosphorylated adapter RNA export protein	PHAX	1,210360687	658380000
DNA replication complex GINS protein PSF1	GINS1	1,210067764	212750000
E3 ubiquitin-protein ligase KCMF1	KCMF1	1,209467713	193500000
Guanine nucleotide-binding protein G(s) subunit alpha isoforms short;Guanine nucleotide-binding protein G(s) subunit alpha	GNAS;GSA	1,209438458	1213900000
Casein kinase II subunit beta	CSNK2B;CSNK2B-LY6G5B-1181;C	1,208999794	4704700000
Fibronectin type III domain-containing protein 3B	FNDC3B	1,208663701	15657000
PHD finger protein 6	PHF6	1,208269396	65856000
Rab5 GDP/GTP exchange factor	RABGEF1	1,20783158	53746000
Serine/threonine-protein kinase Nek6	NEK6	1,207627374	1454800000
Chromosome-associated kinesin KIF4A	KIF4A	1,207496136	679450000
Replication protein A 70 kDa DNA-binding subunit;Replication protein A 70 kDa DNA-binding subunit, N-terminally processed	RPA1	1,206913199	1180600000
Hydroxymethylglutaryl-CoA synthase, cytoplasmic	HMGCS1	1,206039848	319070000

Exosome complex component RRP43	EXOSC8;DKFZp564C0482	1,205865329	833840000
D-tyrosyl-tRNA(Tyr) deacylase;D-tyrosyl-tRNA(Tyr) deacylase 1	DTD1	1,205603646	1235300000
Transcription initiation factor TFIID subunit 1;Transcription initiation factor TFIID subunit 1-like	TAF1;TAF1L	1,205269438	71472000
Alpha-taxilin	TXLNA	1,204659623	370530000
Molybdopterin synthase sulfur carrier subunit	MOCS2	1,204616089	36603000
Retinal rod rhodopsin-sensitive cGMP 3,5-cyclic phosphodiesterase subunit delta	PDE6D	1,204456489	248670000
Secernin-3	SCRN3	1,204456489	210240000
	TARDBP	1,204224419	97406000
CCR4-NOT transcription complex subunit 2	CNOT2	1,204137416	171440000
Transcription factor E2F4	E2F4	1,204122917	15047000
1,4-alpha-glucan-branching enzyme	GBE1	1,203760548	18339000
WAS/WASL-interacting protein family member 1	WIPF1;WASPIP	1,203354954	689700000
Cell division control protein 42 homolog	CDC42	1,203152259	18243000
Cytochrome c oxidase subunit 5A, mitochondrial	COX5A	1,201720864	3349500000
Calcium-binding protein 39-like	CAB39L	1,201677542	469560000
Ankyrin repeat and KH domain-containing protein 1	ANKHD1;FLJ00246	1,201663102	611320000
Chromobox protein homolog 1	CBX1	1,201460977	328950000
Cold shock domain-containing protein E1	CSDE1;UNR	1,201129061	6071700000
Paxillin	PXN	1,201100208	87819000
DNA replication complex GINS protein SLD5;DNA replication complex GINS protein SLD5, N-terminally processed	GINS4	1,200912694	107920000
Glutaminase kidney isoform, mitochondrial	GLS	1,200696404	2601900000
Lysine-specific histone demethylase 1A	KDM1A	1,200624325	2564200000
Transcription initiation factor TFIID subunit 6	TAF6	1,1999904	79218000
Protein CDV3 homolog	CDV3	1,199673689	4919000000
UPF0609 protein C4orf27;Putative UPF0609 protein C4orf27-like	C4orf27	1,199558562	1326400000
Nuclear inhibitor of protein phosphatase 1;Activator of RNA decay	PPP1R8	1,199529784	2323100000
Cytoplasmic dynein 1 light intermediate chain 2	DYNC1LI2;DKFZp686J08252	1,199170174	65002000
E2/E3 hybrid ubiquitin-protein ligase UBE2O	UBE2O	1,199112657	1830900000
Protein farnesyltransferase subunit beta	FNTB	1,19881078	362380000
Sideroflexin-1	SFXN1	1,198509055	670680000
39S ribosomal protein L4, mitochondrial	MRPL4	1,197475721	607230000
Adenylosuccinate synthetase isozyme 2	ADSS	1,19734668	10477000000
Ribonucleoprotein PTB-binding 1	RAVER1	1,197246333	1355000000
High mobility group protein B3	HMGB3;DKFZp779G118	1,197060021	7314700000
N-alpha-acetyltransferase 40	NAA40	1,196845116	653620000
G-rich sequence factor 1	GRSF1	1,196730532	43109000
PDZ domain-containing protein GIPC1	GIPC1	1,196716211	1253600000
Dual specificity mitogen-activated protein kinase kinase 3	MAP2K3	1,196530063	166990000
Ras GTPase-activating protein-binding protein 2	G3BP2	1,196515746	133790000
Calcium signal-modulating cyclophilin ligand	CAMLG	1,195914755	73056000
Intersectin-2	ITSN2	1,195200076	959110000
Steroid receptor RNA activator 1	SRA1	1,19450052	404320000
DCN1-like protein;DCN1-like protein 5	DCUN1D5	1,194414916	464860000
Friend leukemia integration 1 transcription factor	FLI1	1,194386384	109560000
YTH domain-containing family protein 2	YTHDF2	1,193288943	746990000
Proteasome subunit beta type-5	PSMB5	1,193004223	8428100000
Clustered mitochondria protein homolog	CLUH	1,192790773	7042000000
Uncharacterized protein CXorf38	CXorf38	1,192776545	278000000
Nuclear pore glycoprotein p62	NUP62	1,192264587	1371700000
YTH domain-containing family protein 3	YTHDF3;DKFZp451J085;DKFZp45	1,192122455	992020000
FH1/FH2 domain-containing protein 1	FHOD1	1,191809882	108880000
Histone-binding protein RBBP4	RBBP4	1,191738866	3527200000
Syntaxin-binding protein 1	stxbp1;STXBP1	1,191724664	630860000
Chromobox protein homolog 3	CBX3	1,191483278	9762900000
Protein C8orf37	C8orf37	1,191043354	167610000
RNA-binding protein with multiple splicing	RBPMS;hCG_2043421	1,190773884	2270600000
Ubiquitin-conjugating enzyme E2 J1	UBE2J1	1,19023531	1421700000
Peptidyl-prolyl cis-trans isomerase D	PPID	1,19023531	13895000000
Palmitoyl-protein thioesterase 1	PPT1	1,189980365	598910000
Neutrophil cytosol factor 2	NCF2	1,189470804	73505000
Nuclear RNA export factor 1	NXF1;DKFZp667O0311	1,189329337	300910000
Exosome complex component RRP45	EXOSC9	1,189315192	441100000
DnaJ homolog subfamily B member 12	DNAJB12	1,189117199	442230000
mRNA export factor	RAE1	1,18908892	2938200000
WD repeat domain phosphoinositide-interacting protein 4	WDR45	1,188947544	158690000
Probable ATP-dependent RNA helicase DDX6	DDX6	1,188495365	7772800000
WD repeat-containing protein 5	WDR5	1,188368252	6267000000
Tyrosine-protein phosphatase non-receptor type 12	PTPN12	1,188340008	779600000
Nuclear pore complex protein Nup205	NUP205	1,188311765	5219600000
		1,187916513	54556000000
NEDD8 ultimate buster 1	NUB1	1,187733093	298270000
Anaphase-promoting complex subunit 7	ANAPC7	1,187394619	329190000
DNA-directed RNA polymerases I and III subunit RPAC1	POLR1C	1,187338225	834070000
Atlastin-2	ATL2	1,187324128	170410000
Gelsolin	GSN	1,186887269	683340000
Solute carrier family 25 member 36	SLC25A36	1,186633757	355000000
Methionine-R-sulfoxide reductase B2, mitochondrial	MSRB2	1,186450733	85834000
Ran-binding protein 9	RANBP9	1,186450733	663540000
Putative RNA-binding protein Luc7-like 1	LUC7L	1,186436656	964820000
Homeobox protein cut-like 1	CUX1	1,186366279	480620000
DNA polymerase alpha subunit B	POLA2	1,186324056	219160000
Cytosolic Fe-S cluster assembly factor NUBP1	NUBP1	1,186183336	50216000
Mevalonate kinase	MVK	1,185916061	33284000
Spectrin beta chain, non-erythrocytic 1	SPTBN1	1,185733257	100080000
Zinc finger protein 593	ZNF593	1,185311618	105230000
Serine-threonine kinase receptor-associated protein	STRAP	1,184918359	23076000000
Protein cornichon homolog 1	CNIH1;CNIH	1,184707792	169060000
E3 ubiquitin-protein ligase;E3 ubiquitin-protein ligase Itchy homolog	ITCH	1,184413123	186160000
Digestive organ expansion factor homolog	DIEXF	1,184090559	170150000
Neurochondrin	NCDN	1,183922335	805360000
T-complex protein 1 subunit zeta-2	CCT6B	1,183754158	823480000
ATP-dependent RNA helicase DDX55	DDX55	1,18357202	147570000
Cullin-4B	CUL4B	1,183544004	1610700000
RRP12-like protein	RRP12	1,183249914	2454400000
Dual adapter for phosphotyrosine and 3-phosphotyrosine and 3-phosphoinositide	DAPP1	1,183137918	215730000
Protein NEDD1	NEDD1	1,182858022	25155000
Mitogen-activated protein kinase 8	MAPK8	1,182228738	425820000
Protein FAM188A	FAM188A	1,181851489	389620000
Protein arginine N-methyltransferase 3	PRMT3	1,181809587	596950000
Nuclear receptor-binding protein	NRBP1	1,181460521	661310000
LIM and SH3 domain protein 1	LASP1	1,181083762	4845200000
Protein zyg-11 homolog B	ZYG11B	1,181041915	170770000
Uncharacterized protein C9orf78	C9orf78	1,180679363	61910000
S1 RNA-binding domain-containing protein 1	SRBD1	1,180219521	122710000
Synaptic vesicular amine transporter	SLC18A2	1,180094172	1331200000
Nascent polypeptide-associated complex subunit alpha;Nascent polypeptide-associated complex subunit alpha, muscle-spe	NACA	1,179927081	40033000000
Sorting nexin-1	SNX1	1,179843553	1035600000
TBC1 domain family member 17	TBC1D17	1,179815713	42582000

Maspardin	SPG21	1,179787874	430130000
Importin subunit alpha-4;Importin subunit alpha	KPNA3	1,179773955	3914700000
YTH domain-containing family protein 1	YTHDF1	1,178897731	29144000
Histone H2A;Histone H2A type 1-C;Histone H2A type 3;Histone H2A type 1-B/E	HIST1H2AB;HIST1H2AC;HIST3H2/	1,178772662	2,3305E+11
Protein-tyrosine kinase 2-beta	PTK2B	1,178592054	894920000
Thymidylate synthase	TYMS	1,178592054	201670000
Pleckstrin homology domain-containing family F member 2	PLEKHF2	1,178453162	334710000
Histone acetyltransferase type B catalytic subunit	HAT1	1,178244886	1075600000
Geranylgeranyl transferase type-2 subunit beta	RABGGTB	1,177648236	696750000
E3 ubiquitin-protein ligase HECTD1	HECTD1	1,177634368	2354300000
Protein ENL	MLLT1	1,177578898	68867000
		1,177578898	252340000
Ataxin-2-like protein	ATXN2L	1,177426381	5720000000
Microtubule-associated protein RP/EB family member 1	MAPRE1	1,177121467	8831300000
Cell division cycle protein 27 homolog	CDC27	1,177038336	65047000
DNA-directed RNA polymerase II subunit RPB9	POLR2I	1,176982922	204290000
Adaptin ear-binding coat-associated protein 2	NECAP2	1,176733623	820590000
POTE ankyrin domain family member E;POTE ankyrin domain family member F	POTEE;POTEF	1,176581325	37090000000
REST corepressor 1	RCOR1	1,176304522	152090000
E3 ubiquitin-protein ligase TRIM33	TRIM33	1,175986359	2321100000
CWF19-like protein 1	CWF19L1	1,17568219	668010000
Splicing factor 1	SF1	1,175391993	6085400000
RNA-binding protein 28	RBM28	1,175226231	73802000
UPF0469 protein KIAA0907	KIAA0907	1,174977675	20605000
Mitotic checkpoint protein BUB3	BUB3	1,173915595	14602000000
Ribonuclease P/MRP protein subunit POP5	POP5	1,173832917	48725000
Phosducin-like protein 3	PDCL3	1,173653819	901170000
Protein canopy homolog 3	CNPY3	1,173405928	828040000
Thioredoxin-like protein 4A	TXNL4A	1,173158142	198570000
E3 ubiquitin-protein ligase CBL	CBL	1,17286919	561920000
Ubiquitin carboxyl-terminal hydrolase 15	USP15	1,172827923	2564100000
NHP2-like protein 1;NHP2-like protein 1, N-terminally processed	NHP2L1	1,172704138	2656600000
E3 ubiquitin-protein ligase RING2	RNF2	1,172291713	1350500000
ATP-dependent RNA helicase DDX25	DDX25	1,172223004	396290000
Chromodomain-helicase-DNA-binding protein 8	CHD8	1,172085609	685030000
cAMP-regulated phosphoprotein 19	ARPP19	1,17183838	156270000
Hepatoma-derived growth factor	HDGF	1,17183838	16457000000
rRNA 2-O-methyltransferase fibrillarin	FBL	1,171701076	6392900000
Annexin;Annexin A1	ANXA1	1,171659891	10845000000
Myomegalin	PDE4DIP	1,171495179	921840000
Exportin-5	XPO5	1,171495179	2431900000
Poly [ADP-ribose] polymerase 2	PARP2	1,171481455	19908000
Ubiquitin-conjugating enzyme E2 D3;Ubiquitin-conjugating enzyme E2 D2	UBE2D3;UBE2D2	1,171303075	10807000000
Cyclin-H	CCNH	1,170507883	285380000
Endoplasmic reticulum resident protein 29	ERP29	1,170480482	5936100000
Vacuolar protein sorting-associated protein 37B	VPS37B	1,170165461	54594000
Nucleolysin TIAR	TIAL1	1,169891668	3265900000
Proteinase-activated receptor 1	thrombin receptor;F2R	1,169727454	509850000
Recombining binding protein suppressor of hairless	RBPJ	1,169508572	4019100000
Replication factor C subunit 4	RFC4	1,169399163	2889500000
Cation-dependent mannose-6-phosphate receptor	M6PR	1,169371813	726700000
Poly(A) polymerase alpha	PAPOLA	1,169371813	1480400000
RNA-binding protein 27	RBM27;POU4F3	1,169276101	949320000
Striatin-4	STRN4	1,169071056	207600000
GDP-fucose protein O-fucosyltransferase 1	POFUT1	1,168647524	928330000
Carnitine O-acetyltransferase	CRAT	1,168156066	141200000
Ribosomal RNA small subunit methyltransferase NEP1	EMG1	1,168074196	1075100000
DNA-directed RNA polymerase;DNA-directed RNA polymerase I subunit RPA1	POLR1A	1,167965054	799100000
Protein farnesyltransferase/geranylgeranyltransferase type-1 subunit alpha	FNTA	1,167583219	1135000000
Putative RNA-binding protein 3	RBM3	1,16746054	2438700000
SAP30-binding protein	SAP30BP	1,167419652	296590000
Mortality factor 4-like protein 2	MORF4L2	1,167024554	569010000
Exosome complex component RRP4	EXOSC2	1,166194358	2052200000
Probable ATP-dependent RNA helicase DDX47	DDX47;E4-DBP	1,16603118	919810000
Serine/threonine-protein phosphatase 2A 65 kDa regulatory subunit A beta isoform	PPP2R1B	1,16603118	1301500000
Dual specificity protein phosphatase 3	DUSP3	1,165990392	1771000000
Mediator of RNA polymerase II transcription subunit 11	MED11	1,165963202	85389000
SEC14-like protein 2	SEC14L2	1,165650608	548880000
Minor histocompatibility protein HA-1;Minor histocompatibility antigen HA-1	HMHA1	1,16493092	1204800000
Toll-interacting protein	TOLLIP	1,164768095	1451600000
Girdin	KIAA1212;CCDC88A	1,164008846	90912000
Small ubiquitin-related modifier 3	SUMO3	1,163521281	5546700000
Serine/threonine-protein kinase Nek7	NEK7	1,163345316	317970000
Phosphatidylinositol 4,5-bisphosphate 3-kinase catalytic subunit beta isoform	PIK3CB;DKFZp779K1237	1,163264119	32484000
Y-box-binding protein 3	YBX3	1,163209994	961740000
SAM domain-containing protein SAMSN-1	SAMSN1	1,162993545	1710700000
Calcium-binding protein 39	CAB39	1,162885351	19660000000
Exportin-6	XPO6	1,162547374	74650000
Arf-GAP domain and FG repeat-containing protein 1	AGFG1	1,162236608	245320000
Ubiquitin carboxyl-terminal hydrolase 46;Ubiquitin carboxyl-terminal hydrolase 12	USP46;USP12	1,16126485	101330000
AP2-associated protein kinase 1	AAK1	1,161089566	1314800000
Beta-actin-like protein 2	ACTBL2	1,160954769	2137500000
Torsin-1B	TOR1B	1,160766106	447810000
Nucleoporin Nup37	NUP37	1,160671797	499120000
Peptidyl-prolyl cis-trans isomerase E;Peptidyl-prolyl cis-trans isomerase	PPIE	1,160671797	1773400000
Transcription initiation factor TFIID subunit 9;Transcription initiation factor TFIID subunit 9B	AK6;TAF9;TAF9B	1,160456291	496370000
Probable dimethyladenosine transferase	DIMT1	1,159541285	853560000
Ornithine aminotransferase, mitochondrial;Ornithine aminotransferase, hepatic form;Ornithine aminotransferase, renal form	OAT	1,159433733	4057200000
Small ubiquitin-related modifier 1	SUMO1	1,158667995	583450000
Transcription factor jun-D	JUND	1,158600874	165890000
SURP and G-patch domain-containing protein 1	SF4;SUGP1	1,15858745	20458000
Phosphomannomutase 1	PMM1	1,15858745	42379000
Hematological and neurological expressed 1-like protein	HN1L	1,158560604	837660000
tRNA (guanine-N(7)-)-methyltransferase	METTL1	1,158399555	687980000
DENN domain-containing protein 4B	DENND4B	1,158225136	169470000
DNA helicase B	HELB	1,15776921	139270000
Putative Polycomb group protein ASXL1	ASXL1;ASXH1	1,157648584	32054000
Integrator complex subunit 12	INTS12	1,157541382	40990000
Serine/threonine-protein phosphatase 4 catalytic subunit;Serine/threonine-protein phosphatase	PPP4C	1,157085995	3107800000
Torsin-1A	TOR1A	1,156430331	111770000
DNA replication complex GINS protein PSF2	GINS2	1,15593573	221510000
Replication factor C subunit 3	RFC3	1,155895646	2729500000
DNA-directed RNA polymerase;DNA-directed RNA polymerase I subunit RPA2	POLR1B	1,155882285	415660000
DnaJ homolog subfamily B member 6	DNAJB6	1,155615134	456830000
H/ACA ribonucleoprotein complex subunit 1	GAR1	1,155508308	1232700000
N-alpha-acetyltransferase 20	NAA20	1,155294716	1052500000
Splicing regulatory glutamine/lysine-rich protein 1	SREK1	1,15506786	787420000
Lactoylglutathione lyase	GLO1	1,15478775	20467000000

Neuropathy target esterase	PNPLA6	1,154774415	64346000
Ubiquitin-conjugating enzyme E2 A	UBE2A	1,154361177	458950000
Cyclin-dependent kinase 17	CDK17	1,154134688	32043000
ADP-ribosylation factor GTPase-activating protein 2	ARFGAP2	1,153788464	569450000
Pleckstrin homology domain-containing family A member 2	PLEKHA2	1,153748529	63104000
Protein NDRG3	NDRG3	1,153708596	778760000
Twinfilin-2	TWF2	1,152817486	5084500000
Polypyrimidine tract-binding protein 3	PTBP3	1,152764329	361960000
Calcyclin-binding protein	CACYBP	1,152658029	13845000000
Gamma-adducin	ADD3	1,152166649	577990000
RNA-binding protein FUS	FUS	1,151980831	4415100000
Probable ATP-dependent RNA helicase DDX5	DDX5;DKFZp686J01190	1,151887944	19032000000
M-phase phosphoprotein 6	MPHOSPH6	1,151635899	168610000
Protein FAM83H	FAM83H	1,151277918	58585000
Selenoprotein T	SELT	1,151277918	654460000
Putative pre-mRNA-splicing factor ATP-dependent RNA helicase DHX15	DHX15	1,151118888	21865000000
Ubiquitin-conjugating enzyme E2 K	UBE2K;HIP2	1,150734744	14626000000
Far upstream element-binding protein 3	FUBP3	1,150443496	1541800000
Tubulin beta-3 chain	TUBB3	1,150390558	286990000
Alpha-mannosidase 2x	MAN2A2	1,150152395	811170000
Proteasome inhibitor PI31 subunit	PSMF1	1,149729239	514040000
tRNA (adenine(58)-N(1))-methyltransferase catalytic subunit TRMT61A	TRMT61A	1,149663149	1478700000
Suppressor of IKBKE 1	SIKE1	1,149002666	80427000
Nuclear pore complex protein Nup85	NUP85	1,148791471	109220000
ATP-dependent RNA helicase DDX3X;ATP-dependent RNA helicase DDX3Y	DDX3X;DDX3Y	1,148567162	20990000000
GPN-loop GTPase 1	GPN1;MBDin	1,148448446	55898000
Golgi to ER traffic protein 4 homolog	GET4	1,148382503	449030000
Protein tyrosine phosphatase type IVA 2	PTP4A2	1,148382503	524090000
Phosphofurin acidic cluster sorting protein 2	PACS2	1,147855232	19997000
Ubiquitin carboxyl-terminal hydrolase 48	USP48	1,147381103	219070000
Serine/threonine-protein kinase 38	STK38	1,147328446	423160000
BAG family molecular chaperone regulator 5	BAG5	1,147209985	83735000
	RPL10	1,147052076	2136700000
High mobility group protein HMG-I/HMG-Y	HMGA1	1,147012606	1511000000
Mitotic spindle assembly checkpoint protein MAD2A	MAD2L1	1,146828446	2429500000
Kinesin-associated protein 3	KIFAP3	1,146552317	14776000
Cytohesin-1;Cytohesin-2;Cytohesin-3	CYTH3;PSCD2L;CYTH1;CYTH2	1,146499736	78084000
Exosome complex component RRP46	EXOSC5	1,146394589	740020000
		1,146263182	63311000
Alpha-actinin-1	ACTN1	1,146210628	27570000000
Eukaryotic initiation factor 4A-I	EIF4A1	1,145803495	1,9271E+11
Vinculin	VCL	1,145750982	26510000000
Splicing factor, suppressor of white-apricot homolog	SFSWAP	1,145475372	167530000
Erythroid differentiation-related factor 1	EDRF1	1,145449131	25129000
Serine/threonine-protein phosphatase 6 regulatory ankyrin repeat subunit C	ANKRD52	1,145331058	42812000
Cyclin-dependent kinase 9	CDK9	1,145094986	1946100000
	AARSD1	1,144990096	200280000
Mediator of RNA polymerase II transcription subunit 19	MED19;MED19AS	1,144885225	90744000
Histone H2B;Histone H2B type 1-C/E/F/G/I;Histone H2B type F-S;Histone H2B type 1-K;Histone H2B type 1-L;Histone H2B	HIST1H2BC;H2BFS;HIST1H2BK;HI	1,144714852	2,5745E+11
Integrin-linked protein kinase	ILK	1,144295686	1029400000
Zinc finger RNA-binding protein	ZFR	1,143811408	944720000
Tubulin-folding cofactor B	TBCB	1,143759079	519710000
CDP-diacylglycerol--glycerol-3-phosphate 3-phosphatidyltransferase, mitochondrial	PGS1	1,14358904	74866000
Glutathione synthetase	GSS	1,143523654	578200000
Switch-associated protein 70	SWAP70	1,143484426	667460000
Protein syndesmos	NUDT16L1	1,143118427	778320000
Phosphatidylinositol 5-phosphate 4-kinase type-2 alpha	PIP4K2A	1,142883266	677150000
Methyltransferase-like protein 17, mitochondrial	METTL17	1,142517652	1199900000
Nucleoporin Nup43	NUP43	1,141682841	473070000
Eukaryotic translation initiation factor 5	EIF5	1,141513418	5552300000
DNA-directed RNA polymerase II subunit RPB11-b2;DNA-directed RNA polymerase II subunit RPB11-b1;DNA-directed RNA	POLR2J1;POLR2J3;POLR2J2;POL	1,141396156	204570000
General transcription factor IIH subunit 2-like protein;General transcription factor IIH subunit 2	GTF2H2C;GTF2H2	1,141383128	226310000
Pericentriolar material 1 protein	PCM1	1,141278917	456270000
17-beta-hydroxysteroid dehydrogenase 13	HSD17B13	1,141213795	1560300000
Protein unc-119 homolog B	UNC119B	1,141187748	109950000
Nucleosome assembly protein 1-like 1	NAP1L1	1,140836233	12593000000
Cathepsin B;Cathepsin B light chain;Cathepsin B heavy chain	CTSB	1,140797189	307620000
General transcription factor 3C polypeptide 4	GTF3C4	1,140745135	1917000000
PH and SEC7 domain-containing protein 4	PSD4	1,140693085	100430000
Alpha/beta hydrolase domain-containing protein 14B	ABHD14B	1,140654051	8001500000
Anaphase-promoting complex subunit 2	ANAPC2	1,140497941	78836000
Active regulator of SIRT1	RPS19BP1	1,140497941	145390000
WD40 repeat-containing protein SMU1;WD40 repeat-containing protein SMU1, N-terminally processed	SMU1	1,140289862	2549600000
Polymerase delta-interacting protein 3	POLDIP3	1,140003876	255140000
Ubiquitin carboxyl-terminal hydrolase 10	USP10	1,139912911	347350000
Importin subunit alpha;Importin subunit alpha-5;Importin subunit alpha-5, N-terminally processed	KPNA1	1,139860937	1054900000
Probable helicase with zinc finger domain	HELZ	1,139847944	127520000
Cleavage and polyadenylation specificity factor subunit 2	CPSF2	1,139354442	1451800000
Transgelin-2	TAGLN2	1,139068925	1,1039E+11
Ras-related protein Rab-27B	RAB27B	1,138952164	837930000
Protein PBDC1	PBDC1	1,138913249	2152800000
Protein yippee-like 5	YPEL5	1,138614989	400850000
Cytoplasmic protein NCK1	NCK1	1,138278013	462890000
Gamma-aminobutyric acid receptor-associated protein-like 2	GABARAPL2	1,1381096	175570000
Protein Dr1	DKFZp666G145;DR1	1,138096647	1343400000
Pre-mRNA-processing factor 19	PRPF19	1,137980085	5476000000
DNA polymerase epsilon subunit 4	POLE4	1,137837653	405230000
Syntaxin-8	STX8	1,137630543	733880000
Phosphatidylinositol 4-phosphate 5-kinase type-1 alpha	PIP5K1A	1,13760466	22242000
Nucleoporin NUP188 homolog	NUP188	1,13747526	1526300000
E3 ubiquitin-protein ligase MARCH5	05-Mar	1,137100167	878240000
Armadillo repeat-containing protein 8	ARMC8	1,13677701	306740000
Signal recognition particle receptor subunit alpha	SRPR	1,136647798	480260000
Tetratricopeptide repeat protein 27	TTC27	1,136531533	497930000
RNA-binding protein 33	RBM33	1,136466952	233870000
Protein LSM12 homolog	LSM12	1,136389463	3405800000
Lipoyl synthase, mitochondrial	LIAS	1,13626034	94673000
DCN1-like protein 1;DCN1-like protein	DCUN1D1	1,135576475	3360100000
Isocitrate dehydrogenase [NADP];Isocitrate dehydrogenase [NADP] cytoplasmic	IDH1	1,135267072	1340000000
Nucleolysin TIA-1 isoform p40	TIA1	1,135228408	110290000
DNA primase large subunit	PRIM2	1,135060896	91963000
Negative elongation factor A	NELFA;WHSC2	1,135035129	179690000
Replication factor C subunit 2	RFC2	1,13481616	2363200000
Sodium-coupled neutral amino acid transporter 2	SLC38A2	1,134610148	937220000
Nuclear pore complex protein Nup93	NUP93	1,134262673	4500600000
Chromatin complexes subunit BAP18	C17orf49;BAP18;RNASEK-C17orf4	1,134198349	141530000
Notchless protein homolog 1	NLE1	1,134146895	1467500000
Geranylgeranyl transferase type-1 subunit beta	PGGT1B	1,133863982	1862700000

Alpha-adducin	ADD1;LA04NC01-25A3.2-001	1,133761139	801280000
Histone H3.2	HIST2H3A	1,13368402	1,289E+11
Protein timeless homolog	TIMELESS	1,13360691	188050000
Transforming growth factor beta-1;Latency-associated peptide	TGFB1	1,133144476	1595000000
Coiled-coil domain-containing protein 50	CCDC50	1,132785065	195580000
Fragile X mental retardation syndrome-related protein 1	FXR1	1,132785065	1554000000
Ubiquitin-fold modifier 1	UFM1	1,132708078	2390700000
DnaJ homolog subfamily C member 8	DNAJC8	1,132669589	5966300000
Protein 4.1	EPB41	1,132477181	2462200000
Sex hormone-binding globulin	SHBG	1,132425883	307350000
Proteasomal ubiquitin receptor ADRM1	ADRM1;DKFZp686G2045	1,132400236	1755300000
Dipeptidyl peptidase 8	DPP8	1,13155453	123790000
Translationally-controlled tumor protein	TPT1	1,13155453	8973500000
UBX domain-containing protein 4	UBXN4	1,131080974	255630000
Protein RTF2 homolog	RTFDC1	1,130889105	158690000
cAMP-dependent protein kinase type II-beta regulatory subunit	PRKAR2B	1,130825163	2140500000
Zinc finger protein 622	ZNF622	1,130812376	180470000
AP-1 complex subunit sigma-2	DKFZp779P0659;AP1S2	1,130569468	121600000
Nucleophosmin	NPM1	1,130186142	1,7152E+11
Exosome complex component MTR3	EXOSC6	1,130147823	520400000
Arginine/serine-rich coiled-coil protein 2	RSRC2;FLJ11021	1,129994576	49134000
Fermitin family homolog 3	FERMT3	1,129969039	27174000000
Exosome complex component RRP42	EXOSC7	1,129752019	2347800000
Protein SET	SET	1,129394757	1636500000
N6-adenosine-methyltransferase subunit METTL14	METTL14	1,129139708	159470000
Charged multivesicular body protein 7	CHMP7	1,128961243	134710000
Protein PRRC2C	PRRC2C	1,128540797	1919000000
Zinc finger MIZ domain-containing protein 1	ZMIZ1	1,128413451	181740000
Neutral amino acid transporter B(0)	SLC1A5	1,128247944	1782300000
Transcriptional repressor protein YY1	YY1	1,128235214	503230000
Mediator of RNA polymerase II transcription subunit 22	MED22	1,128209757	147550000
Importin subunit alpha;Importin subunit alpha-7;Importin subunit alpha-6	KPNA6;KPNA5	1,128197028	11396000
Chromodomain-helicase-DNA-binding protein 4	CHD4	1,128197028	17585000000
Ubiquitin-like protein 7	UBL7	1,128057035	734720000
Nuclear factor interleukin-3-regulated protein	NFIL3	1,127917076	83246000
Protein Red	CSA2;IK;DKFZp564B112	1,127459271	401460000
	EIF4ENIF1	1,127395716	247050000
Nuclear pore complex protein Nup214	NUP214;DKFZp686J0330	1,127332168	1225700000
Integrator complex subunit 11	CPSF3L	1,126760563	364620000
	DDX19L	1,126735172	59563000
Protein DEK	DEK	1,126709782	4150200000
Inorganic pyrophosphatase	PPA1	1,126659005	18530000000
Probable ATP-dependent RNA helicase DDX17	DDX17	1,126620926	5858900000
Glycogen phosphorylase, brain form;Phosphorylase	PYGB	1,126252956	3101100000
Putative deoxyribose-phosphate aldolase	DERA	1,126214904	1336100000
UDP-N-acetylglucosamine--peptide N-acetylglucosaminyltransferase 110 kDa subunit	OGT	1,126088083	1509700000
Glycerophosphodiester phosphodiesterase domain-containing protein 1	GDPD1	1,126012003	37560000
Pre-mRNA-splicing factor SPF27	BCAS2	1,12591058	2769800000
Cysteine-rich and transmembrane domain-containing protein 1	CYSTM1	1,125631761	139100000
Protein VAC14 homolog	VAC14	1,125568412	770560000
Tropomyosin alpha-1 chain	TPM1	1,124834087	4001500000
Inverted formin-2	INF2	1,124796131	496770000
2-methoxy-6-polyprenyl-1,4-benzoquinol methylase, mitochondrial	COQ5	1,124416709	266540000
MICAL-like protein 1;MICAL-like protein 2	MICALL1;DKFZp686M2226;MICAL-	1,124366139	29604000
Inositol polyphosphate 5-phosphatase K	INPP5K	1,124353497	12905000
Quinone oxidoreductase-like protein 1	CRYZL1	1,124340855	1252900000
Exosome complex component RRP40	EXOSC3	1,124176541	502170000
RNA polymerase II subunit A C-terminal domain phosphatase SSU72	SSU72	1,124037543	2443700000
Lysine-specific demethylase 3B	KDM3B;JMJD1B	1,124012274	340410000
SNW domain-containing protein 1	SNW1	1,123709139	502330000
Serine/threonine-protein phosphatase 2A 56 kDa regulatory subunit epsilon isoform	PPP2R5E	1,123519763	454110000
60S ribosomal protein L13	RPL13	1,123393547	47768000000
Peptidyl-prolyl cis-trans isomerase-like 3;Peptidyl-prolyl cis-trans isomerase	PPIL3	1,123292595	368470000
Histone-lysine N-methyltransferase SETD1A	SETD1A	1,123279978	326380000
Delta-aminolevulinic acid dehydratase	ALAD	1,122952016	4495200000
Ribose-5-phosphate isomerase	RPIA	1,122901578	4676000000
IST1 homolog	IST1	1,122762895	2131700000
Protein transport protein Sec24B	SEC24B	1,1222211	1152100000
Riboflavin kinase	RFK	1,122170727	80482000
Eukaryotic translation initiation factor 4B	EIF4B	1,12191893	5955800000
Exosome complex component RRP41	EXOSC4	1,121491135	2138600000
DNA damage-binding protein 1	DDB1	1,121264787	22796000000
Histone-lysine N-methyltransferase setd3	SETD3	1,121239643	308380000
Far upstream element-binding protein 2	KHSRP	1,121239643	13019000000
THO complex subunit 3	THOC3	1,121151647	908800000
DNA topoisomerase 2;DNA topoisomerase 2-beta	TOP2B	1,121063665	799690000
Arf-GAP with GTPase, ANK repeat and PH domain-containing protein 3	AGAP3;CENTG3	1,120548621	51018000
Fanconi anemia group I protein	FANCI	1,120347756	65017000
Integral membrane protein 2B;BRI2, membrane form;BRI2 intracellular domain;BRI2C, soluble form;Bri23 peptide	ITM2B	1,120297551	343570000
Uncharacterized protein C18orf8	C18orf8	1,119845909	1220400000
UDP-N-acetylglucosamine--peptide N-acetylglucosaminyltransferase 110 kDa subunit	OGT	1,119645296	30314000
60S acidic ribosomal protein P2	RPLP2	1,119645296	6506900000
Heterogeneous nuclear ribonucleoprotein A1;Heterogeneous nuclear ribonucleoprotein A1, N-terminally processed;Heteroge	HNRNPA1;HNRPA1;HNRNPA1L2	1,119595154	42822000000
Glutaredoxin-1	GLRX;hCG_1979388	1,119532483	621600000
Protein FAM45A;Protein FAM45B	FAM45A;FAM45B	1,11951995	605710000
Polycomb protein EED	EED	1,119469819	204940000
Syntaxin-5	STX5A;STX5	1,119382101	740770000
Prenylcysteine oxidase-like	PCYOX1L	1,11919418	2094900000
U6 snRNA-associated Sm-like protein LSm1	LSM1	1,118668337	1163800000
Serine/threonine-protein kinase MARK2	MARK2;Par1b	1,118092981	278170000
Actin-related protein 10	ACTR10	1,118042978	665740000
Casein kinase I isoform alpha;Casein kinase I isoform alpha-like	CSNK1A1;CSNK1A1L	1,117967981	2463100000
Ubiquilin-2	UBQLN2	1,117892995	1090200000
Induced myeloid leukemia cell differentiation protein Mcl-1	MCL1	1,117868002	145480000
WD repeat-containing protein 70	WDR70	1,117305952	46755000
Protein FAM192A	FAM192A;NIP30	1,117181129	599270000
Glycogen synthase kinase-3 beta	GSK3B	1,117156167	1718600000
Adenine phosphoribosyltransferase	APRT	1,117106248	5338300000
Nuclear transport factor 2	NUTF2	1,116956517	5153500000
Protein BUD31 homolog	BUD31	1,116657175	1307100000
Eukaryotic translation initiation factor 4 gamma 2	EIF4G2;AAG1	1,116632237	7684900000
Mitogen-activated protein kinase;Mitogen-activated protein kinase 1	MAPK1	1,116544963	19879000000
60S ribosomal export protein NMD3	NMD3	1,116482633	176220000
DNA replication licensing factor MCM5	MCM5	1,116457703	9189200000
Protein kinase C delta type;Protein kinase C delta type regulatory subunit;Protein kinase C delta type catalytic subunit;Protei	PRKCD	1,116445238	1042600000
Reticulocalbin-1	RCN1	1,116345531	1876400000
Elongation factor 1-alpha;Elongation factor 1-alpha 1;Putative elongation factor 1-alpha-like 3	EEF1A1;EEF1A1P5;EEF1A1L14;PT	1,116308146	8,0069E+11
Protein phosphatase 1G	PPM1G	1,116245842	2107200000

Poly(rC)-binding protein 1	PCBP1	1,116220922	1,0892E+11
Caspase-7;Caspase-7 subunit p20;Caspase-7 subunit p11	CASP7	1,115847263	449400000
Protein S100-A10	S100A10	1,115623187	332340000
Malic enzyme;NADP-dependent malic enzyme	ME1	1,115610741	66345000
Thioredoxin-related transmembrane protein 2	TMX2	1,115200178	1928000000
Dual specificity protein kinase CLK3	CLK3	1,115150434	30490000
Charged multivesicular body protein 5	CHMP5	1,115001227	1125400000
Ubiquitin fusion degradation protein 1 homolog	ufd1;UFD1L	1,1149515	2225300000
Peflin	PEF1	1,114814774	1337500000
Poly(ADP-ribose) glycohydrolase ARH3	ADPRHL2	1,114417216	1365300000
Calcineurin B homologous protein 1	CHP1	1,114404796	1266400000
Tubulin--tyrosine ligase-like protein 12	TTLL12	1,114379959	1042000000
RNMT-activating mini protein	FAM103A1	1,114193714	181430000
Protein VPRBP	VPRBP	1,114081996	604870000
ADP-ribosylation factor 5	ARF5	1,114019941	3186200000
N6-adenosine-methyltransferase 70 kDa subunit	METTL3	1,113635351	307720000
Zinc finger MYM-type protein 2	ZMYM2	1,113548545	58028000
Na(+)/H(+) exchange regulatory cofactor NHE-RF1	SLC9A3R1	1,113362578	1926200000
Ras-related protein Rab-8B	RAB8B	1,113312997	1996600000
	PQBP1	1,113176672	4356700000
Retinoblastoma-like protein 1	RBL1	1,113065159	455670000
Parafibromin	CDC73	1,11305277	313910000
Small ubiquitin-related modifier 2;Small ubiquitin-related modifier 4	SUMO2;SUMO4	1,112656467	2218800000
DNA replication licensing factor MCM4	MCM4	1,112656467	11023000000
Protein QIL1	C19orf70;QIL1	1,112606949	189660000
COP9 signalosome complex subunit 8	COPS8	1,112309934	1679000000
26S proteasome non-ATPase regulatory subunit 10	PSMD10	1,112272818	640920000
CLIP-associating protein 1	CLASP1	1,112136748	1267500000
Histone deacetylase;Histone deacetylase 1	HDAC1	1,112112012	2973300000
Ubiquitin-conjugating enzyme E2 E3	UBE2E3	1,112013077	2517600000
Transcriptional adapter 1	TADA1	1,111827622	176760000
Ankyrin repeat domain-containing protein 13A	ANKRD13;ANKRD13A	1,111580445	86043000
Coiled-coil and C2 domain-containing protein 1B	CC2D1B	1,111308677	309290000
Breakpoint cluster region protein	BCR;BCR/ABL fusion	1,111074075	1036400000
DnaJ homolog subfamily C member 7	DNAJC7	1,110962983	1612900000
Renin receptor	ATP6AP2	1,110888933	233230000
Filamin-B	FLNB;DKFZp686A1668	1,11071619	28486000000
Exosome complex exonuclease RRP44	DIS3	1,110420183	2104200000
Sorting nexin-4	SNX4	1,110346206	91230000
Alpha/beta hydrolase domain-containing protein 17B	ABHD17B	1,110148982	702660000
BRCA1-A complex subunit BRE	BRE	1,110087364	2185000000
E3 ubiquitin-protein ligase RNF185	RNF185	1,109890232	98745000
Neuron-specific calcium-binding protein hippocalcin	HPCA	1,109828642	198850000
Eukaryotic translation initiation factor 3 subunit J	EIF3J	1,109816325	9196300000
Ran-binding protein 3	ZNF8;RANBP3	1,109779376	527540000
Thyroid receptor-interacting protein 11	TRIP11;DKFZp686C06243	1,109213124	174860000
Inositol polyphosphate 1-phosphatase	INPP1	1,109163912	230210000
Telomerase-binding protein EST1A	SMG6	1,108967108	206860000
RING finger protein 214	RNF214	1,10875808	55141000
TBC1 domain family member 15	TBC1D15	1,10875808	1583900000
Histone deacetylase complex subunit SAP130	SAP130	1,108745787	125810000
Short coiled-coil protein	SCOC	1,108696616	32262000
CDK-activating kinase assembly factor MAT1	MNAT1	1,108573709	566730000
	RPLP1	1,108438543	3893600000
Ankyrin repeat and LEM domain-containing protein 2	ANKLE2	1,108278843	31970000
DNA replication licensing factor MCM6	MCM6	1,107885932	11062000000
Partner of Y14 and mago	WIBG	1,107542363	152460000
Splicing factor 3B subunit 1	SF3B1	1,107382922	35746000000
MHC class II regulatory factor RFX1	RFX1	1,107199008	55009000
Ankyrin repeat and SAM domain-containing protein 1A	ANKS1A	1,107186749	55309000
DNA-directed RNA polymerases I, II, and III subunit RPABC1	POLR2E	1,106880368	2052900000
Protein furry homolog-like	FRYL	1,106843614	1496000000
Cyclin-dependent kinase 2	CDK2	1,106831363	2960000000
DnaJ homolog subfamily C member 9	DNAJC9	1,106770113	797830000
Zinc finger CCCH domain-containing protein 7B	ZC3H7B	1,106476205	16273000
DNA replication licensing factor MCM7	MCM7	1,10645172	8608100000
Kidney mitochondrial carrier protein 1;Brain mitochondrial carrier protein 1	SLC25A30;SLC25A14	1,105998938	152390000
Bifunctional protein NCOAT;Protein O-GlcNAcase;Histone acetyltransferase	MGEA5	1,105974474	4410200000
Heat shock 70 kDa protein 4L	HSPA4L	1,105950011	1516300000
Histone H3	HIST2H3PS2	1,105680989	10316000000
Signal recognition particle receptor subunit beta	SRPRB	1,105644314	2750300000
Serine/threonine-protein kinase PAK 3	PAK3	1,105399878	398010000
Nuclear factor NF-kappa-B p100 subunit;Nuclear factor NF-kappa-B p52 subunit	NFKB2	1,105277701	887490000
TRMT1-like protein	TRMT1L	1,105155551	70395000
FAD synthase;Molybdenum cofactor biosynthesis protein-like region;FAD synthase region	FLAD1	1,105045638	22553000
Flavin reductase (NADPH)	BLVRB	1,105033427	1890800000
Golgi phosphoprotein 3	GOLPH3	1,104886915	657100000
Small integral membrane protein 11	SMIM11	1,104752646	96030000
14-3-3 protein theta	YWHAQ	1,104606208	30670000000
Formin-binding protein 4	FNBP4	1,104228089	477960000
GPN-loop GTPase 3	GPN3	1,104167127	153570000
Immunoglobulin-binding protein 1	IGBP1	1,103838045	180100000
Chromatin target of PRMT1 protein	CHTOP	1,103630946	887520000
F-actin-capping protein subunit alpha-1	CAPZA1	1,103606586	11130000000
Charged multivesicular body protein 2b	CHMP2B	1,103582228	402330000
Tumor necrosis factor receptor type 1-associated DEATH domain protein	TRADD	1,103387399	436740000
Serine/threonine-protein phosphatase;Serine/threonine-protein phosphatase 2A catalytic subunit alpha isoform	PPP2CA	1,103363051	15593000000
14-3-3 protein eta	YWHAH	1,103326529	29816000000
Cysteine and glycine-rich protein 1	CSRP1;DKFZp686M148	1,103314356	5209900000
Enolase-phosphatase E1	ENOPH1	1,103131791	1239200000
Histone-lysine N-methyltransferase EZH2	EZH2	1,103119622	91767000
Protein strawberry notch homolog 1	SBNO1	1,103107454	2395700000
Exocyst complex component 5	EXOC5;DKFZp666H126	1,103083117	203150000
Cleavage stimulation factor subunit 2	CSTF2	1,102864138	1364000000
CUGBP Elav-like family member 2	CELF2	1,102803326	884030000
Ubiquilin-1	UBQLN1	1,102572301	2073300000
SWI/SNF-related matrix-associated actin-dependent regulator of chromatin subfamily B member 1	SMARCB1	1,102450748	1059500000
14-3-3 protein beta/alpha;14-3-3 protein beta/alpha, N-terminally processed	YWHAB	1,102438594	25672000000
Integrin alpha-5;Integrin alpha-5 heavy chain;Integrin alpha-5 light chain	ITGA5	1,102426441	486650000
Bifunctional ATP-dependent dihydroxyacetone kinase/FAD-AMP lyase (cyclizing);ATP-dependent dihydroxyacetone kinase;	DAK	1,102365677	31265000
V-type proton ATPase subunit d 1	ATP6V0D1	1,102329222	3521900000
Trafficking protein particle complex subunit 5	TRAPPC5	1,10223202	1368900000
ORM1-like protein 3	ORMDL3	1,102219871	22173000
DnaJ homolog subfamily C member 21	DNAJC21	1,102049813	67449000
Integrin alpha-V;Integrin alpha-V heavy chain;Integrin alpha-V light chain	ITGAV	1,10198909	150480000
Poly(U)-binding-splicing factor PUF60	PUF60	1,101964803	4826400000
Cyclin-T1;Cyclin-T2	CCNT1;CCNT2	1,101891948	72477000
Guanine nucleotide-binding protein subunit alpha-13	GNA13	1,101879807	774630000

Ribosome maturation protein SBDS	SBDS	1,101685579	8971100000
Tumor suppressor p53-binding protein 1	TP53BP1	1,10144289	876830000
UBX domain-containing protein 7	UBXN7	1,10113968	749510000
Clathrin interactor 1	CLINT1	1,10113968	2331000000
SOSS complex subunit B1	NABP2	1,101103306	74380000
Uridine 5-monophosphate synthase;Orotate phosphoribosyltransferase;Orotidine 5-phosphate decarboxylase;Orotidine 5-pl	UMPS	1,100872992	5762400000
Ubiquitin carboxyl-terminal hydrolase;Ubiquitin carboxyl-terminal hydrolase 4	USP4	1,100703349	991490000
E3 ubiquitin-protein ligase RNF126	RNF126	1,100400546	422310000
Factor VIII intron 22 protein	F8A1	1,100340005	65040000
Ribosome-binding protein 1	RRBP1	1,100110011	2590800000
DNA polymerase epsilon subunit 3	POLE3	1,099964801	759060000
Replication factor C subunit 1	RFC1	1,099964801	713290000
Protein tyrosine phosphatase type IVA 1	PTP4A1	1,099843822	1794300000
Niban-like protein 1	FAM129B	1,099795438	379150000
Trafficking protein particle complex subunit 4	TRAPPC4	1,099239326	1960500000
Apoptosis inhibitor 5	API5	1,099203078	7940000000
Heterogeneous nuclear ribonucleoprotein A0	HNRNPA0	1,099178913	5871400000
Nuclear pore complex protein Nup50	NUP50	1,09915475	417530000
Protein MEMO1	MEMO1	1,099021871	6116700000
Rho guanine nucleotide exchange factor 6	ARHGEF6	1,098997714	363080000
Annexin;Annexin A3	ANXA3	1,098683777	711670000
Lysophospholipid acyltransferase 5	LPCAT3	1,098599286	1043700000
Heterogeneous nuclear ribonucleoprotein K	HNRPK;HNRNPK	1,098563079	66558000000
TATA-box-binding protein;TATA box-binding protein-like protein 2	TBP;TBPL2	1,098454475	431150000
Cyclin-dependent kinase 7	CDK7	1,098406213	1160500000
Craniofacial development protein 1	CFDP1	1,098273514	782690000
Exportin-T	XPOT	1,097996157	6014300000
Spastin	SPAST	1,097899718	42975000
Probable ATP-dependent RNA helicase DHX35	DHX35	1,097851505	41598000
Scaffold attachment factor B1;Scaffold attachment factor B2	SAFB;SAFB2	1,097658694	91555000
Replication factor C subunit 5	RFC5	1,09751413	4160500000
PHD finger-like domain-containing protein 5A	PHF5A	1,097453907	1403400000
Heterogeneous nuclear ribonucleoprotein K	HNRNPK	1,097417776	74039000
Protein HGH1 homolog	C8orf30A;HGH1	1,097032527	335140000
THUMP domain-containing protein 1	DKFZp686C1054;THUMPD1;44M2.	1,09690016	5596200000
EMILIN-2	EMILIN2	1,09658742	355730000
Arfaptin-1	ARFIP1	1,096551346	1369500000
TBC1 domain family member 4	TBC1D4	1,09645516	52558000
Probable ATP-dependent RNA helicase DDX27	DDX27	1,096443138	67046000
U4/U6 small nuclear ribonucleoprotein Prp4	PRPF4	1,096190737	841480000
RNA polymerase II-associated factor 1 homolog	PAF1	1,09575832	431120000
Mannosyl-oligosaccharide 1,2-alpha-mannosidase IA	MAN1A1	1,095698289	68738000
m7GpppX diphosphatase	DCPS	1,095638264	6418700000
Oxysterol-binding protein;Oxysterol-binding protein-related protein 9	OSBPL9	1,09562626	306020000
ATP-binding cassette sub-family E member 1	ABCE1	1,095470231	21089000000
Myotubularin-related protein 12	MTMR12	1,09543423	89934000
Splicing factor 3B subunit 2	SF3B2;DKFZp781L0540	1,095386233	9205800000
Phosphatidylinositol 5-phosphate 4-kinase type-2 gamma	PIP4K2C	1,09533824	311570000
Ubiquitin carboxyl-terminal hydrolase isozyme L5	UCHL5	1,095230272	10103000000
MAP kinase-activated protein kinase 2	MAPKAPK2	1,095122325	1126500000
Splicing factor 3B subunit 4	SF3B4	1,094990419	1384000000
Splicing factor 3B subunit 6	SF3B6	1,09495445	2533700000
MOB kinase activator 2	MOB2	1,094534987	286730000
ELKS/Rab6-interacting/CAST family member 1	ERC1	1,094511027	570590000
F-box-like/WD repeat-containing protein TBL1X	TBL1X	1,094451133	1155600000
Zinc finger CCHC domain-containing protein 8	ZCCHC8	1,094415199	169390000
Synaptojanin-1	SYNJ1	1,094367292	567990000
Phosphatidylinositol-binding clathrin assembly protein	PICALM	1,094355315	2213900000
Eyes absent homolog 3	EYA3	1,094032055	137190000
ORM1-like protein 1;ORM1-like protein 2	ORMDL1;ORMDL2;ORMDL3	1,093984181	301840000
Tubulin-specific chaperone A	TBCA	1,093984181	1454800000
PAX3- and PAX7-binding protein 1	PAXBP1	1,093864514	412080000
Tubulin alpha-4A chain	TUBA4A	1,093756836	7923600000
ATP-dependent RNA helicase DDX19A	DDX19A;DDX19B;DKFZp762C1313	1,093589379	3140600000
WW domain-binding protein 11	WBP11	1,093457842	757230000
Protein-L-isoaspartate O-methyltransferase;Protein-L-isoaspartate(D-aspartate) O-methyltransferase	PCMT1	1,093445885	4908200000
TATA-binding protein-associated factor 172	BTAF1	1,093290476	8573900000
Zinc finger protein ubi-d4	DPF2	1,093278524	1667200000
	PIK3CG	1,093182911	434340000
Myosin light polypeptide 6	MYL6;PDE6H	1,093170961	29468000000
Protein phosphatase inhibitor 2-like protein 3;Protein phosphatase inhibitor 2	PPP1R2;PPP1R2P3	1,093170961	2122200000
Protein Hook homolog 3	HOOK3	1,093123162	426830000
Polyribonucleotide 5-hydroxyl-kinase Clp1	CLP1	1,093027577	417380000
60S ribosomal protein L30	RPL30	1,092872287	43376000000
Glutamate--cysteine ligase regulatory subunit	GCLM	1,092800629	5231400000
Inhibitor of nuclear factor kappa-B kinase subunit beta	IKBKB	1,092788687	224960000
	HSP90Af	1,092705101	44432000
Programmed cell death protein 6	PDCD6	1,092633465	4750000000
Lymphoid-specific helicase	HELLS	1,092609589	116210000
Nucleoporin SEH1	SEH1L	1,092537966	730670000
Inositol-3-phosphate synthase 1	ISYNA1	1,092358949	3027500000
Transcription factor p65	RELA	1,092335085	587970000
Cellular nucleic acid-binding protein	CNBP	1,092072645	23029000000
PDZ domain-containing protein 11	PDZD11	1,092060719	377340000
Kinesin-like protein KIF2A	KIF2A	1,092060719	1784800000
Trafficking protein particle complex subunit 2-like protein	TRAPPC2L	1,092013017	81442000
La-related protein 7	LARP7	1,091631553	474900000
Protein PAT1 homolog 1	PATL1	1,091429008	193560000
Guanine nucleotide exchange factor MSS4	RABIF	1,091345629	118150000
Lysosomal alpha-mannosidase;Lysosomal alpha-mannosidase A peptide;Lysosomal alpha-mannosidase B peptide;Lysoson	MANB;MAN2B1	1,091309899	86773000
Tyrosine-protein kinase;Tyrosine-protein kinase SYK	SYK	1,091119379	791160000
Protein SON	SON	1,090857523	230020000
Gem-associated protein 2	GEMIN2	1,090655266	423270000
Heat shock protein 105 kDa	HSPH1	1,090369853	13212000000
Zinc finger CCCH domain-containing protein 7A	ZC3H7A	1,090191547	64093000
6-phosphogluconate dehydrogenase, decarboxylating		1,090191547	11874000
Protein PRRC2B	BAT2L;PRRC2B	1,090108357	24333000
FAS-associated factor 1	FAF1	1,090037061	701600000
Protein FAM76B	FAM76B	1,08988262	397330000
tRNA (guanine(37)-N1)-methyltransferase	TRMT5	1,089823231	130010000
Serine/threonine-protein phosphatase 2B catalytic subunit beta isoform;Serine/threonine-protein phosphatase	PPP3CB	1,08966885	123870000
Xaa-Pro aminopeptidase 1	XPNPEP1	1,089656976	7913400000
Phosphatidylinositol 3-kinase;Phosphatidylinositol 3-kinase catalytic subunit type 3	PIK3C3	1,089514512	43419000
Protein arginine N-methyltransferase 7	PRMT7	1,089383953	144570000
TIP41-like protein	TIPRL	1,089229697	4520000000
AH receptor-interacting protein;Peptidyl-prolyl cis-trans isomerase	AIP	1,089158516	3042400000
Bifunctional purine biosynthesis protein PURH;Phosphoribosylaminoimidazolecarboxamide formyltransferase;IMP cyclohydr	ATIC	1,089063623	9150300000
SEC23-interacting protein	SEC23IP	1,08894503	2493800000

Mediator of RNA polymerase II transcription subunit 17	MED17	1,088933172	262880000
Cytoplasmic FMR1-interacting protein 1	CYFIP1	1,088838318	1664200000
Abhydrolase domain-containing protein 4	ABHD4	1,088802752	261870000
Regulator of nonsense transcripts 1	UPF1	1,088802752	20433000000
U2 small nuclear ribonucleoprotein B	SNRPB2	1,088779043	4759100000
Probable E3 ubiquitin-protein ligase HERC1	HERC1	1,088755335	619970000
Calreticulin	CALR	1,088613107	69969000000
Methyltransferase-like protein 16	METTL16	1,088542007	608970000
Transcription initiation factor TFIID subunit 7	TAF7	1,088435374	32732000
40S ribosomal protein S27	RPS27	1,088352452	11328000000
3-ketodihydrosphingosine reductase	KDSR	1,088127441	762030000
	C16orf45	1,088032728	519370000
Pre-mRNA-splicing factor SYF1	XAB2;DKFZp762C1015	1,087985377	417450000
Cathepsin D;Cathepsin D light chain;Cathepsin D heavy chain	CTSD	1,087949867	2144900000
Protein zwilch homolog	ZWILCH	1,087831517	92900000
Serine/threonine-protein phosphatase 2A 56 kDa regulatory subunit delta isoform	PPP2R5D	1,08780785	930790000
Protein FAM98A	FAM98A	1,087748687	293860000
Guanine nucleotide-binding protein G(k) subunit alpha	GNAI3	1,087701361	1902000000
Transgelin-3	TAGLN3	1,08768953	20462000000
Ubiquitin-conjugating enzyme E2 Z	UBE2Z	1,08761855	3640100000
Transcription initiation factor IIA subunit 1;Transcription initiation factor IIA alpha chain;Transcription initiation factor IIA beta c	HIST1H4F;GTF2A1	1,087559408	522470000
Cellular nucleic acid-binding protein	CNBP	1,087452968	515340000
Signal transducing adapter molecule 1	STAM	1,087452968	277490000
Elongation factor 1-beta	LOC392793;EEF1B2	1,087417492	12601000000
Protocadherin Fat 4	FAT4	1,08731108	258210000
52 kDa repressor of the inhibitor of the protein kinase	PRKRIR	1,087287435	72124000
Guanine nucleotide-binding protein G(I)/G(S)/G(T) subunit beta-2	GNB2	1,087216508	5077500000
Cyclin-K	CCNK	1,08715741	246830000
Pleckstrin homology domain-containing family A member 5	PLEKHA5	1,08715741	343400000
Plasminogen activator inhibitor 1 RNA-binding protein	SERBP1;DKFZp686P17171	1,08715741	16961000000
mRNA-decapping enzyme 1A	DCP1A;Nbla00360	1,087098317	87614000
	RPS10-NUDT3	1,087027415	35604000000
UV excision repair protein RAD23 homolog B	RAD23B	1,086980152	6206800000
Transcription factor BTF3 homolog 4	BTF3L4	1,086921079	3014100000
Glucosamine-6-phosphate isomerase 2	GNPDA2	1,086897451	501390000
GMP synthase [glutamine-hydrolyzing]	GMPS	1,086814763	8435600000
Acyl-protein thioesterase 1	LYPLA1	1,086743898	1925000000
Nuclear receptor coactivator 2	NCOA2	1,086531357	60758000
WD repeat-containing protein 61;WD repeat-containing protein 61, N-terminally processed	WDR61	1,086425118	1707900000
Beta-catenin-like protein 1	CTNNBL1	1,086413315	1404600000
Regulator of G-protein signaling 14	RGS14	1,08638971	12087000
Exportin-2	CSE1L	1,086366105	26354000000
High mobility group protein HMG-I/HMG-Y	HMGA1	1,085941403	491370000
Thioredoxin	TXN	1,085835279	10891000000
Methionine aminopeptidase 2;Methionine aminopeptidase	METAP2	1,085646665	3762900000
Prostaglandin reductase 2	PTGR2	1,085623093	1043300000
Filamin-A	FLNA;FLJ00119	1,085564168	1,9472E+11
Membrane magnesium transporter 1	MMGT1	1,08539921	64559000
Neuroblast differentiation-associated protein AHNAK	AHNAK	1,085257857	26193000
dCTP pyrophosphatase 1	DCTPP1	1,085151867	115830000
Pre-mRNA cleavage complex 2 protein Pcf11	PCF11	1,085081218	349440000
Golgi SNAP receptor complex member 2	GOSR2	1,085069444	368960000
RNA-binding protein 4	RBM4	1,084998807	269740000
Nuclear pore complex protein Nup155	NUP155	1,084822252	7939600000
Transaldolase	TALDO1	1,084669284	44202000000
Cysteine and histidine-rich domain-containing protein 1	CHORDC1	1,084645755	8310200000
U4/U6.U5 tri-snRNP-associated protein 2	USP39	1,084575172	1466400000
Transporter;Sodium- and chloride-dependent glycine transporter 1	SLC6A9	1,08451636	76378000
Peroxiredoxin-6	PRDX6	1,084481076	38736000000
Ras association domain-containing protein 5	RASSF5;FLJ00186	1,084422274	621770000
Transcription intermediary factor 1-beta	TRIM28	1,084140114	16652000000
39S ribosomal protein L47, mitochondrial	MRPL47	1,084128361	152480000
Transportin-1	TNPO1	1,083670174	10778000000
PC4 and SFRS1-interacting protein	PSIP1	1,083071591	3416200000
	LLDBP	1,083048131	262490000
NAD kinase 2, mitochondrial	NADK2	1,083012942	197100000
Mediator of RNA polymerase II transcription subunit 10	MED10	1,082860484	152440000
Hepatocyte growth factor-regulated tyrosine kinase substrate	HGS	1,082801858	570110000
Nuclear pore complex protein Nup107	NUP107	1,082485386	507960000
ER membrane protein complex subunit 7	EMC7	1,082450234	1815400000
60S acidic ribosomal protein P1	RPLP1	1,082450234	2544100000
Forkhead box protein K1	FOXK1;KIAA0415	1,082415085	265300000
ADP-ribosylation factor-related protein 1	ARFRP1	1,082215945	640020000
Tyrosine-protein kinase Lyn	LYN	1,082215945	1416200000
Protein transport protein Sec61 subunit beta	SEC61B	1,0821691	1505700000
General transcription factor 3C polypeptide 1	DKFZp686A111;GTF3C1;DKFZp68	1,082122258	552690000
Centrin-3	CETN3	1,082110548	52348000
N-acylneuraminate-9-phosphatase	NANP	1,082063712	581310000
60S ribosomal protein L10	RPL10	1,082028587	47526000000
Leucine-rich repeat and calponin homology domain-containing protein 1	LRCH1	1,081923227	56052000
Helicase-like transcription factor	HLTF	1,081899816	36042000
Selenoprotein K	SELK	1,081876406	194640000
Peroxisomal targeting signal 1 receptor	PEX5	1,081864702	86903000
Phosphatidylinositol 3-kinase regulatory subunit alpha	PIK3R1	1,081841294	930560000
DNA replication licensing factor MCM2	MCM2	1,081642366	17962000000
Glucose-induced degradation protein 8 homolog	GID8	1,081548778	1434700000
[Protein ADP-ribosylarginine] hydrolase	ADPRH	1,081221348	333740000
E3 ubiquitin-protein ligase listerin	LTN1	1,081116144	440660000
Heat shock 70 kDa protein 1-like	HSPA1L	1,081104456	415240000
Myosin-10	MYH10	1,081069394	11632000000
Golgi SNAP receptor complex member 1	GOSR1	1,08096422	1069000000
Glucosamine-6-phosphate isomerase 1	GNPDA1	1,080940851	1495100000
ADP-ribose pyrophosphatase, mitochondrial	NUDT9	1,080905799	213600000
40S ribosomal protein S20	RPS20	1,080894116	33651000000
60S acidic ribosomal protein P0;60S acidic ribosomal protein P0-like	RPLP0;RPLP0P6	1,080882432	1,1447E+11
ER membrane protein complex subunit 8	COX4NB;EMC8	1,080859067	1882100000
Splicing factor, arginine/serine-rich 15	SFRS15;SCAF4	1,080812339	214620000
Cullin-3	CUL3	1,0806605	2000200000
CCR4-NOT transcription complex subunit 11	CNOT11	1,080613789	810690000
60S ribosomal protein L22-like 1	RPL22L1	1,080462006	17693000000
Vacuolar protein sorting-associated protein VTA1 homolog	VTA1	1,080415312	2798400000
DNA repair endonuclease XPF	ERCC4	1,08035695	89356000
Ribonuclease P protein subunit p14	RPP14	1,080135233	49291000
		1,080135233	11388000000
Group XIIA secretory phospholipase A2	PLA2G12A	1,0801119	48850000
	CIC	1,080053571	89226000
RNA-binding protein Musashi homolog 2	IGSF9B;MSI2	1,079855299	2028800000
Programmed cell death protein 5	PDCD5	1,07976202	2287400000

Minor histocompatibility antigen H13	HM13	1,079563856	4118400000
Disks large-associated protein 4	DLGAP4	1,079552202	506570000
Endophilin-B1	SH3GLB1	1,079493933	1454300000
AT-rich interactive domain-containing protein 1A	ARID1A	1,079412368	2011400000
U5 small nuclear ribonucleoprotein 40 kDa protein	WDR57;SNRNP40	1,079365765	2269000000
Annexin;Annexin A7	ANXA7	1,079365765	10442000000
Rho-associated protein kinase 2	ROCK2	1,079354114	3376500000
ATPase ASNA1	ASNA1	1,079330815	7822500000
Ubiquitin carboxyl-terminal hydrolase 47	USP47	1,079214332	2732500000
F-actin-capping protein subunit alpha-2	CAPZA2	1,079074586	28521000000
Heat shock 70 kDa protein 13	HSPA13	1,078737015	588900000
Methylsterol monooxygenase 1	MSMO1	1,078643929	82391000
Src kinase-associated phosphoprotein 2	SKAP2;SCAP2	1,078609026	480890000
Myosin regulatory light chain 12A;Myosin regulatory light chain 12B;Myosin regulatory light polypeptide 9	MYL12A;MYL12B;MYL9	1,078550859	21164000000
Myb-binding protein 1A	MYBBP1A	1,078539226	4485500000
DNA replication licensing factor MCM3	HCC5;MCM3	1,078353139	13592000000
Proteasome activator complex subunit 3	PSME3	1,078260119	10789000000
Nuclear distribution protein nudE homolog 1;Nuclear distribution protein nudE-like 1	NDE1;NDEL1	1,07812062	80229000
E3 ubiquitin-protein ligase ARIH2	ARIH2	1,078039262	233660000
Glycylpeptide N-tetradecanoyltransferase;Glycylpeptide N-tetradecanoyltransferase 1	NMT1	1,077946297	3329000000
Queuine tRNA-ribosyltransferase subunit QTRTD1	QTRTD1	1,077806879	325910000
Vesicle transport protein USE1	USE1	1,077806879	124200000
RNA-binding protein 6	RBM6	1,077783646	195270000
Adenylyl cyclase-associated protein	CAP1	1,077737183	29149000000
Tyrosine-protein phosphatase non-receptor type 11	PTPN11	1,077667496	1316400000
Inosine-5-monophosphate dehydrogenase 2	IMPDH2	1,077191546	11244000000
Serine/threonine-protein kinase tousled-like 2	TLK2	1,077029123	340260000
GTPase-activating protein and VPS9 domain-containing protein 1	GAPVD1	1,07683196	3086200000
Putative heat shock protein HSP 90-alpha A5	HSP90AA5P	1,076820365	530030000
Glycylpeptide N-tetradecanoyltransferase;Glycylpeptide N-tetradecanoyltransferase 2	NMT2	1,076681238	238720000
Cytosolic purine 5-nucleotidase	NT5C2	1,076658053	3142400000
60S ribosomal protein L10a;Ribosomal protein	RPL10A	1,076403091	1,0935E+11
Nucleolin	NCL	1,076345162	77048000000
RRP15-like protein	RRP15	1,07614825	144310000
Heterogeneous nuclear ribonucleoprotein H2;Heterogeneous nuclear ribonucleoprotein H2, N-terminally processed	HNRNPH2	1,076090349	95302000
60S ribosomal protein L27a	L27a;RPL27A	1,076020875	8513900000
Ubiquitin-associated domain-containing protein 1	UBAC1	1,07595141	179430000
Nuclear receptor corepressor 1	NCOR1	1,075858804	344900000
RNA-binding protein 26	RBM26	1,075754642	25094000
Ran GTPase-activating protein 1	RANGAP1	1,075673641	1690800000
Mannose-P-dolichol utilization defect 1 protein	MPDU1;HBEBP2BPA	1,075604221	477380000
Protein unc-119 homolog A	UNC119;DKFZp686E1393	1,07553481	918430000
Histone-lysine N-methyltransferase EHMT2	EHMT2	1,075500108	189700000
Muscleblind-like protein 1	MBNL1	1,075476974	8612600000
Selenocysteine-specific elongation factor	EEFSEC	1,075106973	1199900000
		1,074887406	966830000
26S proteasome non-ATPase regulatory subunit 5	PSMD5	1,074864298	2428200000
Vacuolar fusion protein CCZ1 homolog;Vacuolar fusion protein CCZ1 homolog B	DKFZp586I1023;CCZ1;CCZ1B	1,074783431	443480000
CXXC-type zinc finger protein 1	CXXC1	1,074679477	65939000
Peptidyl-prolyl cis-trans isomerase-like 4	PPIL4	1,07439082	951630000
N-alpha-acetyltransferase 35, NatC auxiliary subunit	NAA35	1,074136931	526240000
RNA polymerase-associated protein LEO1	LEO1	1,074113856	69573000
MOB kinase activator 3A	MOB3A;DKFZp686N2312	1,073917759	207520000
Serine/threonine-protein kinase ATR	ATR	1,073837034	290950000
40S ribosomal protein SA	RPSA;RPSAP58	1,073802442	1,4181E+11
60S ribosomal protein L17	RPL17	1,073802442	51258000000
Myosin light chain 1/3, skeletal muscle isoform;Myosin light chain 3	MYL1;MYL3	1,073617985	630270000
Midasin	MDN1	1,073617985	3645300000
Elongation factor 2	EEF2	1,073306858	1,691E+11
GPN-loop GTPase 2	GPN2	1,073295339	67934000
ADP-ribosylation factor GTPase-activating protein 1	ARFGAP1	1,073283819	590730000
60S ribosomal protein L12	RPL12;hCG_21173	1,073237744	66063000000
Proline synthase co-transcribed bacterial homolog protein	PROSC	1,073226225	3817100000
Sjoegren syndrome/scleroderma autoantigen 1	SSSCA1	1,072731174	683890000
OTU domain-containing protein 6B	OTUD6B	1,072616111	1536900000
Tether containing UBX domain for GLUT4	ASPSCR1	1,072478068	209960000
Ubiquitin thioesterase OTUB2	OTUB2	1,072443563	429630000
SWI/SNF complex subunit SMARCC1	SMARCC1	1,072190593	7328400000
Pre-mRNA-splicing factor SLU7	SLU7	1,072121621	46103000
Serine/arginine-rich splicing factor 11	SFRS11;SRSF11;DKFZp686D0211	1,071983706	1570200000
Reactive oxygen species modulator 1	ROMO1	1,071845826	157710000
CTP synthase 1;CTP synthase	CTPS1	1,071845826	4100900000
Heterogeneous nuclear ribonucleoprotein L-like	HNRPLL;HNRNPLL	1,071730953	2035500000
SWI/SNF-related matrix-associated actin-dependent regulator of chromatin subfamily D member 1	SMARCD1	1,071375003	90228000
Regulation of nuclear pre-mRNA domain-containing protein 1B	RPRD1B	1,07129466	4481400000
Importin subunit beta-1	KPNB1	1,071248755	50864000000
Serine/threonine-protein phosphatase 4 regulatory subunit 1	PPP4R1	1,070893125	195860000
Spermine synthase	SMS	1,070881657	3723300000
Importin-8	IPO8	1,070847254	531240000
Probable 28S rRNA (cytosine-C(5))-methyltransferase	NSUN5	1,070778456	22761000
Zinc finger E-box-binding homeobox 1	ZEB1	1,07074406	76577000
Putative tRNA pseudouridine synthase Pus10	PUS10	1,070652348	271510000
Transmembrane protein 87A	TMEM87A	1,070572114	291720000
U6 snRNA-associated Sm-like protein LSm5	LSM5	1,07041168	482610000
Islet cell autoantigen 1	ICA1	1,070297114	60264000
Pre-mRNA-splicing factor 38B	PRPF38B	1,070239841	160950000
Protein SET	SET	1,070194026	1,184E+11
Sialic acid synthase	NANS	1,070125312	5574100000
Splicing factor 3A subunit 1	SF3A1	1,070033706	7692800000
DNA-directed RNA polymerase III subunit RPC3	POLR3C	1,069987909	40422000
Protein CASC3	CASC3	1,069781871	32933000
Phosphatidylinositol transfer protein alpha isoform	PITPNA	1,069655999	6750600000
Protein quaking	QKI	1,069575913	1305100000
Protein transport protein Sec23A	SEC23A	1,069553034	2207000000
C-Myc-binding protein	MYCBP	1,06949584	790270000
Tyrosine-protein kinase receptor;Protein TFG	TFG;TFG/ALK fusion	1,069072793	1552900000
Nucleoporin NDC1	NDC1	1,068924235	301630000
Endoplasmic reticulum lectin 1	ERLEC1	1,068878533	139570000
Double-strand break repair protein MRE11A	MRE11A	1,068867108	517740000
DnaJ homolog subfamily B member 1	DNAJB1	1,068672922	980910000
Selenium-binding protein 1	SELENBP1	1,068581565	787860000
Haloacid dehalogenase-like hydrolase domain-containing protein 2	HDHD2	1,068387483	2434400000
RNA-binding protein 14	RBM14	1,068319	1865400000
Actin-like protein 6A	BAF53A;ACTL6A	1,068284762	4183900000
KDEL motif-containing protein 1	KDELC1	1,068125013	159270000
Vacuolar protein sorting-associated protein 4A	VPS4A;DKFZp434E0418	1,068125013	51677000
Helicase SKI2W	SKIV2L;SKI2W	1,068090788	1339500000
Acidic leucine-rich nuclear phosphoprotein 32 family member A;Acidic leucine-rich nuclear phosphoprotein 32 family member	ANP32A;ANP32D	1,067988124	7396300000

SH3 domain-binding glutamic acid-rich-like protein 3	SH3BGRL3;HEL-S-297	1,067919692	3948000000
Tyrosyl-DNA phosphodiesterase 1	TDP1	1,067839867	126520000
40S ribosomal protein S14	RPS14	1,067828464	47098000000
Methionine adenosyltransferase 2 subunit beta	MAT2B	1,067794257	1994200000
Thioredoxin-like protein 1	TXNL1	1,06771445	18459000000
WD repeat domain phosphoinositide-interacting protein 1	WIPI1	1,067429523	23574000
Mediator of RNA polymerase II transcription subunit 8	MED8	1,067418129	354950000
Eukaryotic translation initiation factor 2 subunit 2	EIF2S2	1,067395342	17555000000
Torsin-1A-interacting protein 1	TOR1AIP1	1,06721308	39059000
Histidine triad nucleotide-binding protein 1	HINT1	1,066962571	8867900000
Probable tRNA N6-adenosine threonylcarbamoyltransferase	OSGEP	1,066700801	811680000
Peptidylprolyl isomerase domain and WD repeat-containing protein 1	PPWD1	1,066700801	340580000
Reticulon;Reticulon-3	RTN3	1,066564276	12089000000
Proto-oncogene c-Rel	REL	1,066461906	14741000
Putative heat shock protein HSP 90-beta 2	HSP90AB2P	1,066336813	13386000000
SWI/SNF-related matrix-associated actin-dependent regulator of chromatin subfamily E member 1	SMARCE1	1,066336813	579640000
Transcription initiation factor IIE subunit beta	GTF2E2	1,066268593	1257400000
Adapter molecule crk	CRK	1,066109447	1639700000
DnaJ homolog subfamily C member 3	DNAJC3	1,06605262	2115600000
Lys-63-specific deubiquitinase BRCC36	BRCC3	1,065825375	1486500000
60S ribosomal protein L31	RPL31	1,065711789	29740000000
A-kinase anchor protein 13	AKAP13	1,065689075	175090000
Trafficking protein particle complex subunit 13	C5orf44;TRAPPC13	1,065439281	248360000
Bifunctional methylenetetrahydrofolate dehydrogenase/cyclohydrolase, mitochondrial;NAD-dependent methylenetetrahydrofo	MTHFD2	1,065427929	4657500000
Polypyrimidine tract-binding protein 1	PTBP1	1,065257686	15822000000
Ubiquitin carboxyl-terminal hydrolase 25	USP25	1,065155566	780180000
40S ribosomal protein S12	RPS12	1,065030779	50388000000
Dynactin subunit 6	DCTN6	1,065019437	438630000
Leucine zipper transcription factor-like protein 1	LZTFL1	1,064974068	492130000
Serine/threonine-protein phosphatase 2A 55 kDa regulatory subunit B alpha isoform;Serine/threonine-protein phosphatase 2A	PPP2R2A	1,064894682	5968500000
S-formylglutathione hydrolase	ESD	1,064633926	7892000000
S-adenosylmethionine synthase isoform type-2;S-adenosylmethionine synthase	MAT2A	1,064475267	14321000000
DNA repair protein RAD50	RAD50	1,064418615	4420900000
RANBP2-like and GRIP domain-containing protein 5/6	RGPD5	1,064067504	510190000
Synaptobrevin homolog YKT6	YKT6	1,063988254	4671800000
U3 small nucleolar RNA-interacting protein 2	RRP9	1,063841105	442340000
		1,063705311	692760000
60S ribosomal protein L9	RPL9	1,063682682	62350000000
E3 ubiquitin-protein ligase TRIM56	TRIM56	1,063512996	74995000
LIM domain-containing protein 2	LIMD2	1,063456446	718520000
Eukaryotic translation initiation factor 4H	EIF4H;WBSCR1;LOC392647	1,063264221	11413000000
40S ribosomal protein S7	RPS7	1,063230306	1,0512E+11
GA-binding protein alpha chain	GABPA;GABPAP	1,062936468	573570000
Protein kinase C;Protein kinase C alpha type	PRKCA	1,062902574	482750000
SRSF protein kinase 1	SRPK1	1,062857386	2712800000
Myosin-11	MYH11	1,062834793	514040000
High mobility group protein B2	HMGB2	1,062778315	30453000000
Mediator of RNA polymerase II transcription subunit 20	TRFP;MED20	1,06271055	455420000
Activator of 90 kDa heat shock protein ATPase homolog 1	AHSA1	1,062518594	14134000000
Spermatogenesis-associated protein 5	SPATA5	1,062473438	84129000
Selenide, water dikinase 1	SEPHS1	1,062405711	3176300000
60S ribosomal protein L5	RPL5	1,062146173	77997000000
Brefeldin A-inhibited guanine nucleotide-exchange protein 2	ARFGEF2	1,062134891	885050000
Protein PRRC2A	PRRC2A	1,062089768	2570700000
60S ribosomal protein L7a	RPL7A;RP-L7a	1,062033369	87567000000
Evolutionarily conserved signaling intermediate in Toll pathway, mitochondrial	ECSIT	1,062010811	66401000
Pre-mRNA-splicing factor RBM22	RBM22	1,061875484	445160000
NEDD8-activating enzyme E1 catalytic subunit	UBE1C;UBA3	1,061864209	5283600000
Exocyst complex component 7	DKFZp686P1551;EXOC7	1,061830383	390570000
Importin-5	IPO5	1,061819108	46697000000
COP9 signalosome complex subunit 2	COPS2	1,061706374	9401400000
T-complex protein 1 subunit beta	CCT2	1,061582395	52834000000
Importin-11	IPO11	1,061503514	1251300000
Biorientation of chromosomes in cell division protein 1-like 1	BOD1L1	1,061503514	36558000
Aflatoxin B1 aldehyde reductase member 2	AKR7A2	1,061413378	969040000
Spectrin alpha chain, erythrocytic 1	SPTA1	1,061413378	299310000
Interferon regulatory factor 2-binding protein 1	IRF2BP1	1,061334522	23387000
Serine/threonine-protein phosphatase 2A 65 kDa regulatory subunit A alpha isoform	PPP2R1A	1,061210629	18401000000
ER membrane protein complex subunit 6	EMC6	1,061165584	502080000
CTD nuclear envelope phosphatase 1	CTDNEP1;hCG_1987397	1,061109284	75331000
Coiled-coil domain-containing protein 93	CCDC93	1,060884141	375640000
Large subunit GTPase 1 homolog	LSG1	1,060861632	245270000
Synaptic vesicle membrane protein VAT-1 homolog	VAT1	1,060839124	9936500000
Protein unc-45 homolog A	UNC45A	1,060771605	1141000000
Gamma-tubulin complex component 4	TUBGCP4	1,060704095	11312000
Nucleoplasmin-3	NPM3	1,060659094	3462600000
Ragulator complex protein LAMTOR3	LAMTOR3	1,060625345	817650000
Flap endonuclease 1	FEN1	1,060535358	6772100000
DnaJ homolog subfamily A member 2	DNAJA2	1,060512864	11003000000
60S ribosomal protein L4	RPL4	1,060186805	78327000000
E3 ubiquitin-protein ligase RBX1;E3 ubiquitin-protein ligase RBX1, N-terminally processed	RBX1	1,060085655	1410500000
Transcription elongation regulator 1	TCERG1	1,059838481	11041000000
Host cell factor 1;HCF N-terminal chain 1;HCF N-terminal chain 2;HCF N-terminal chain 3;HCF N-terminal chain 4;HCF N-te	HCFC1	1,059827248	8381000000
SUMO-conjugating enzyme UBC9	UBE2I	1,059816016	7568400000
Serine/threonine-protein phosphatase 4 regulatory subunit 3A	SMEK1	1,059804784	883610000
Protein kinase C iota type	PRKCI	1,059748628	37515000
Ribosomal protein L19;60S ribosomal protein L19	RPL19	1,059737397	28972000000
	LOC202789	1,059703707	92403000
Radixin	RDX	1,059670019	6958700000
Interleukin enhancer-binding factor 2	ILF2	1,059647561	6766200000
WD repeat domain phosphoinositide-interacting protein 2	WIPI2	1,059344478	583150000
Regulator of G-protein signaling 18	RGS18	1,059333256	1070500000
Probable D-tyrosyl-tRNA(Tyr) deacylase 2	DTD2	1,058963063	278490000
Zinc finger protein ZPR1	ZPR1	1,058895783	630720000
Oxysterol-binding protein;Oxysterol-binding protein-related protein 2	OSBPL2	1,058884571	42661000
LIM and senescent cell antigen-like-containing domain protein 1;LIM and senescent cell antigen-like-containing domain protei	LIMS1;LIMS2	1,058671579	1368700000
Heterogeneous nuclear ribonucleoprotein Q	SYNCRIP	1,058581924	20427000000
Transcription elongation factor SPT4	SUPT4H1	1,058548307	242620000
60S ribosomal protein L13a;Putative 60S ribosomal protein L13a protein RPL13AP3	RPL13A;RPL13a;RPL13AP3	1,058413861	28392000000
Sodium/potassium-transporting ATPase subunit alpha-2	ATP1A2	1,058290649	12931000000
Ethylmalonyl-CoA decarboxylase	ECHDC1	1,058223454	703110000
Lysophospholipid acyltransferase 7	MBOAT7	1,058212256	1154900000
Polyadenylate-binding protein 1-like	PABPC1L	1,058178663	49124000
Stromal membrane-associated protein 1	SMAP1	1,058156269	493730000
Bis(5-adenosyl)-triphosphatase ENPP4;Ectonucleotide pyrophosphatase/phosphodiesterase family member 7	ENPP4;ENPP7	1,058033116	226210000
Cell cycle checkpoint protein RAD1	RAD1	1,058033116	2249200000
Eukaryotic peptide chain release factor subunit 1	ETF1	1,057999534	23947000000
T-complex protein 1 subunit delta	CCT4	1,057865228	58448000000

Serine/arginine-rich splicing factor 3	SFRS3;SRSF3	1,057697393	11672000000
ATP-dependent RNA helicase DHX36	DHX36	1,057585532	1391200000
Mediator of RNA polymerase II transcription subunit 23	MED23	1,057451331	2076000000
Elongation factor 1-gamma	EEF1G	1,057350702	1,5735E+11
Splicing factor 3A subunit 2	SF3A2	1,057339522	1367500000
ATP-citrate synthase	ACLY variant protein;ACLY	1,057305984	24893000000
Hydroxypyruvate isomerase;Putative hydroxypyruvate isomerase	HYI	1,057250093	60687000
60S ribosomal protein L23a	RPL23A	1,057171854	66171000000
Adenosine kinase	ADK	1,057138327	2094700000
Lariat debranching enzyme	DBR1	1,057093627	172740000
Proliferating cell nuclear antigen	PCNA	1,056948379	62360000000
Tyrosine-protein kinase CSK	CSK	1,056724998	2017800000
Ubiquitin carboxyl-terminal hydrolase;Ubiquitin carboxyl-terminal hydrolase 28	USP28	1,056658002	47949000
Obg-like ATPase 1	OLA1;PTD004	1,056624507	26524000000
Hsp90 co-chaperone Cdc37;Hsp90 co-chaperone Cdc37, N-terminally processed	CDC37;MBD5	1,056367785	9980000000
40S ribosomal protein S28	RPS28	1,056356626	15839000000
Superkiller viralicidic activity 2-like 2	SKIV2L2	1,056311992	5517300000
SET and MYND domain-containing protein 5	SMYD5	1,056311992	572650000
Prefoldin subunit 1	PFDN1	1,056233892	1452700000
Exocyst complex component 4	EXOC4	1,056200425	1622100000
Ubiquitin thioesterase otulin	OTULIN	1,056155804	92534000
Protein-glutamine gamma-glutamyltransferase 2	TGM2	1,056066574	1707800000
DNA-(apurinic or apyrimidinic site) lyase;DNA-(apurinic or apyrimidinic site) lyase, mitochondrial	APEX1	1,05588816	20257000000
Transmembrane and coiled-coil domain-containing protein 1	TMCO1	1,055854714	4730600000
DnaJ homolog subfamily A member 1	DNAJA1;HDJ2	1,055843566	9933600000
Transcription elongation factor SPT6	SUPT6H	1,055843566	1036100000
CLIP-associating protein 2	CLASP2	1,055776682	2894600000
SPRY domain-containing protein 7	SPRYD7	1,055754389	494920000
Casein kinase II subunit alpha;Casein kinase II subunit alpha 3	CSNK2A1;CSNK2A3	1,055687516	18992000000
RNA-binding protein NOB1	NOB1	1,055609509	450900000
40S ribosomal protein S5;40S ribosomal protein S5, N-terminally processed	RPS5	1,055531513	99168000000
39S ribosomal protein L1, mitochondrial	MRPL1	1,05549809	133640000
Spectrin beta chain, non-erythrocytic 1	SPTBN1	1,055475808	41611000000
Arylamine N-acetyltransferase 2	NAT2	1,055397832	65361000
Nucleolar RNA helicase 2	DDX21	1,055130572	2703400000
B-cell lymphoma/leukemia 10	BCL10	1,05507491	159540000
TGF-beta-activated kinase 1 and MAP3K7-binding protein 1	MAP3K7IP1;TAB1	1,054985863	298590000
TAR DNA-binding protein 43	TARDBP	1,054930216	11966000000
Leucine-rich repeat-containing protein 47	LRRC47	1,054885703	1368200000
Anaphase-promoting complex subunit 4	ANAPC4	1,054852321	277230000
Putative histone H2B type 2-D;Putative histone H2B type 2-C	HIST2H2BD;HIST2H2BC	1,054830067	1513800000
		1,054796688	8380400
Multidrug resistance protein 1	ABCB1	1,054785562	284300000
Filamin A-interacting protein 1-like	FILIP1L	1,054741061	39645000
WD repeat-containing protein 26	WDR26	1,054729936	1205800000
Ubiquitin carboxyl-terminal hydrolase;Ubiquitin carboxyl-terminal hydrolase 19	USP19	1,054718812	929390000
60S ribosomal protein L6	RPL6	1,054607581	59083000000
DNA-directed RNA polymerases I, II, and III subunit RPABC3	POLR2H	1,054329605	2389500000
Eukaryotic translation initiation factor 5B	EIF5B	1,054318489	16135000000
Transcription elongation factor SPT5	SUPT5H	1,054174002	4405600000
Histone-lysine N-methyltransferase EHMT1	EHMT1	1,054062885	51346000
Rab GTPase-binding effector protein 2	RABEP2	1,053974009	257240000
Peptidyl-prolyl cis-trans isomerase FKBP4;Peptidyl-prolyl cis-trans isomerase FKBP4, N-terminally processed	FKBP4	1,053940684	8118000000
T-complex protein 1 subunit epsilon	CCT5	1,053885148	26112000000
Exportin-4	XPO4	1,053785196	3079700000
Tyrosine-protein phosphatase non-receptor type;Tyrosine-protein phosphatase non-receptor type 2	PTPN2	1,053674162	1013400000
Putative uncharacterized protein C11orf40	C11orf40	1,053552051	2135000000
POTE ankyrin domain family member I	POTEI	1,053529852	371940000
Zinc finger CCCH domain-containing protein 15	ZC3H15	1,053385581	1681200000
Core histone macro-H2A.1;Histone H2A	H2AFY	1,053319008	5477400000
Guanine nucleotide-binding protein subunit beta-2-like 1;Guanine nucleotide-binding protein subunit beta-2-like 1, N-terminally	GNB2L1	1,053119339	1,459E+11
Uncharacterized protein C1orf50	C1orf50	1,052919746	208390000
Interferon-related developmental regulator 2	IFRD2	1,052897574	243400000
Cell division cycle 5-like protein	CDC5L	1,052853232	3076600000
Derlin-1	DERL1	1,052842147	1931500000
Uncharacterized protein C19orf43	C19orf43	1,052842147	1101800000
60S ribosomal protein L11	RPL11	1,052842147	61419000000
Nuclease-sensitive element-binding protein 1	YBX1	1,052753477	19922000000
Osteoclast-stimulating factor 1	OSTF1	1,052698065	13329000000
Zinc finger protein 148	ZNF148;tb protein	1,052675902	87596000
E3 ubiquitin-protein ligase BRE1B	RNF40	1,05265374	1002500000
Peptidyl-prolyl cis-trans isomerase NIMA-interacting 1	PIN1	1,052310347	2430600000
Exocyst complex component 6	EXOC6;DKFZp761I2124;SEC15L3	1,052254982	202760000
Presenilin;Presenilin-1;Presenilin-1 NTF subunit;Presenilin-1 CTF subunit;Presenilin-1 CTF12;Presenilin-2;Presenilin-2 NTF :	PSEN2;PSEN1	1,052232838	16658000
Eukaryotic translation initiation factor 4 gamma 3	EIF4G3	1,05214427	762560000
Ras-related protein Rab-38	RAB38	1,052088923	976960000
Uncharacterized aarF domain-containing protein kinase 5	ADCK5	1,052022513	38577000
Ubiquitin carboxyl-terminal hydrolase 14;Ubiquitin carboxyl-terminal hydrolase	USP14	1,051867591	6051000000
Bifunctional coenzyme A synthase;Phosphopantetheine adenylyltransferase;Dephospho-CoA kinase	COASY	1,051856527	171330000
tRNA pseudouridine synthase;tRNA pseudouridine synthase A, mitochondrial	PUS1	1,051856527	663620000
Splicing factor U2AF 65 kDa subunit	U2AF2	1,05175696	6280000000
ATP-binding cassette sub-family F member 3	ABCF3	1,051734837	462970000
Structural maintenance of chromosomes protein 3	SMC3	1,051712714	21217000000
Synergin gamma	SYNRG	1,051690593	184880000
Protein transport protein Sec24A	SEC24A	1,051469429	1213400000
Protein kish-A	TMEM167A	1,051358881	1409300000
WD repeat-containing protein 44	WDR44;DKFZp761M142	1,051314669	360490000
60S ribosomal protein L8	RPL8	1,051292564	86226000000
60S ribosomal protein L35	RPL35;LOC154880	1,051259409	21952000000
NHL repeat-containing protein 2	DKFZp779F115;NHLRC2	1,051049473	87371000
Codanin-1	CDAN1	1,050939014	10501000
NudC domain-containing protein 3	NUDCD3;KIAA1068	1,050828578	501130000
Translational activator GCN1	PRIC295;GCN1L1	1,050751287	28800000000
NAD(P)H-hydrate epimerase	APOA1BP	1,050729206	140090000
Integrator complex subunit 4	INTS4	1,050674007	462030000
Peptidyl-prolyl cis-trans isomerase FKBP8	FKBP8	1,050662968	985540000
Rho GTPase-activating protein 28	ARHGAP28	1,050365002	81786000
Tyrosine-protein phosphatase non-receptor type 23	PTPN23	1,050320873	1456900000
Omega-amidase NIT2	NIT2	1,050265717	1705800000
Cohesin subunit SA-2	STAG2;DKFZp686P16143;DKFZp6	1,050221597	3100100000
Heterogeneous nuclear ribonucleoprotein H;Heterogeneous nuclear ribonucleoprotein H, N-terminally processed	HNRNPH1	1,050133367	19426000000
Oligosaccharyltransferase complex subunit OSTC	OSTC	1,050111312	2880700000
RUN and FYVE domain-containing protein 1	RUFY1	1,050012075	219700000
		1,049593283	130860000
Sorting nexin-5	SNX5	1,049516173	4460200000
60S ribosomal protein L15;Ribosomal protein L15	RPL15	1,049439075	44537000000
Protein arginine N-methyltransferase 6	PRMT6	1,049406036	200370000
Transcriptional activator protein Pur-alpha	PURA	1,049373	5864100000

15 kDa selenoprotein		15-Sep	1,049328954	1013400000
Microtubule-associated protein;Microtubule-associated protein 4	MAP4;DKFZp547H1810		1,049306933	1768100000
40S ribosomal protein S26;Putative 40S ribosomal protein S26-like 1	RPS26;RPS26P11		1,049042748	24052000000
60S ribosomal protein L24	RPL24		1,048998731	42771000000
Prefoldin subunit 5	PFDN5		1,048921708	2454000000
Huntingtin-interacting protein K	HYPK		1,048822697	957940000
Mediator of RNA polymerase II transcription subunit 15	DKFZp762B1216;DKFZp686A2214;		1,048811696	56984000
Heat shock cognate 71 kDa protein	HSPA8		1,048723703	3,7669E+11
	WUGSC:H_RG054D04.1		1,048701707	16871000000
60S ribosomal protein L27	RPL27		1,048701707	55926000000
NudC domain-containing protein 1	NUDCD1		1,048668715	369910000
Splicing factor 3B subunit 3	SF3B3		1,048580746	34624000000
Acidic leucine-rich nuclear phosphoprotein 32 family member B	ANP32B		1,048525773	32120000000
Serine/threonine-protein kinase RIO3	RIOK3		1,048525773	549860000
Glucose-6-phosphate isomerase	GPI		1,048514779	1756900000
Protein arginine N-methyltransferase 5;Protein arginine N-methyltransferase 5;Protein arginine N-methyltransferase 5, N-terı	PRMT5		1,048415844	3920500000
Beta-arrestin-1	ARRB1		1,048360888	111700000
Dual specificity mitogen-activated protein kinase kinase 4	MAP2K4		1,048294948	1214800000
TBC1 domain family member 9B	TBC1D9B		1,048250993	1547900000
N-alpha-acetyltransferase 50	NAA50		1,048207042	7625100000
Luc7-like protein 3	CROP;LUC7L3		1,048130136	1991100000
Gem-associated protein 5	GEMIN5		1,04811915	3043100000
Kinesin light chain 1	KNS2;KLC1		1,048042257	185340000
Peptidyl-prolyl cis-trans isomerase;Peptidyl-prolyl cis-trans isomerase H	PPIH		1,047932429	2586500000
Transcriptional repressor CTCF	CTCF		1,047910467	412150000
Spliceosome-associated protein CWC15 homolog	CWC15		1,047899485	935440000
Exocyst complex component 2	EXOC2		1,047877524	545590000
U6 snRNA-associated Sm-like protein LSm8	LSM8		1,047855564	2049600000
60S ribosomal protein L38	RPL38		1,047767731	23757000000
Eukaryotic translation initiation factor 4 gamma 1	EIF4G1;EIF4G1 variant protein		1,047701866	22681000000
Serine/threonine-protein phosphatase 6 regulatory subunit 1	PPP6R1		1,047657961	261260000
U4/U6 small nuclear ribonucleoprotein Prp3	PRPF3		1,047570161	833500000
Ribonuclease P protein subunit p30	RPP30		1,047482376	1406200000
Probable ATP-dependent RNA helicase YTHDC2	YTHDC2		1,047449461	829930000
3-mercaptopyruvate sulfurtransferase	MPST		1,047416547	387560000
Mannose-6-phosphate isomerase	MPI		1,047087526	707630000
BRISC complex subunit Abro1	FAM175B		1,046901173	344300000
Death-inducer obliterator 1	DIDO1		1,046846375	187410000
Alpha-soluble NSF attachment protein	NAPA		1,046835417	8624100000
40S ribosomal protein S6	RPS6		1,0468135	53484000000
Probable ATP-dependent RNA helicase DDX31	DDX31		1,0468135	75375000
Protein FAM3C	FAM3C		1,046791584	2056700000
Double-strand-break repair protein rad21 homolog	RAD21		1,046791584	945840000
T-complex protein 1 subunit eta	CCT7		1,046747755	41578000000
Cytosine-specific methyltransferase;DNA (cytosine-5)-methyltransferase 1	DNMT1		1,046211146	2023300000
Cleavage stimulation factor subunit 2 tau variant	CSTF2T		1,045948518	69973000
Leucine-rich repeat-containing protein 57	LRRC57		1,045839129	363560000
40S ribosomal protein S13	RPS13		1,04576257	69431000000
Ragulator complex protein LAMTOR5	LAMTOR5		1,04565322	240280000
Ubiquitin-like protein 4A	UBL4A		1,04565322	683770000
40S ribosomal protein S25	RPS25		1,045642286	56189000000
Vesicle-associated membrane protein 3;Vesicle-associated membrane protein 2	VAMP3;VAMP2		1,045609486	940150000
GTPase KRas;GTPase KRas, N-terminally processed	KRAS		1,045543892	482020000
Importin-7	IPO7		1,045369015	35783000000
Charged multivesicular body protein 4b	CHMP4B		1,04510681	6659500000
Glucose-induced degradation protein 4 homolog	GID4		1,045063122	69394000
Probable ubiquitin carboxyl-terminal hydrolase FAF-X;Ubiquitin carboxyl-terminal hydrolase;Probable ubiquitin carboxyl-term	USP9X;USP9Y		1,0450522	12347000000
Ubiquitin-like modifier-activating enzyme 6	UBA6		1,045019437	2401400000
Regulator of nonsense transcripts 2	UPF2		1,044877488	2076400000
Eukaryotic translation initiation factor 3 subunit F	EIF3F		1,044779238	22661000000
Trifunctional purine biosynthetic protein adenosine-3;Phosphoribosylamine--glycine ligase;Phosphoribosylformylglycinamidin	GART		1,044746492	8424200000
40S ribosomal protein S17-like;40S ribosomal protein S17	RPS17L;RPS17		1,044724663	49606000000
Probable methyltransferase TARBP1	TARBP1		1,044713748	806340000
COP9 signalosome complex subunit 7a	COPS7A		1,044615529	4681200000
60S ribosomal protein L23	RPL23		1,044615529	47752000000
Serine/threonine-protein phosphatase 2A activator	PPP2R4;DKFZp781M17165		1,044451872	5641200000
THO complex subunit 6 homolog	THOC6		1,044408238	1273800000
Sorting nexin-27	SNX27		1,044408238	329220000
Sphingomyelin phosphodiesterase 4	SMPD4		1,044266455	417850000
Myosin-9	MYH9		1,044244646	3,3168E+11
Peptidyl-prolyl cis-trans isomerase;Peptidyl-prolyl cis-trans isomerase A;Peptidyl-prolyl cis-trans isomerase A, N-terminally ɾ	PPIA		1,044244646	2,6751E+11
Phosphomannomutase;Phosphomannomutase 2	PMM2		1,044157417	4244700000
Box C/D snoRNA protein 1	ZNHIT6		1,044135612	32885000
Protein cornichon homolog 4	CNIH4		1,043950308	544070000
60S ribosomal protein L14	RPL14		1,043841336	20048000000
40S ribosomal protein S3a	RPS3A		1,043808649	1,0522E+11
Platelet-activating factor acetylhydrolase IB subunit alpha	PAFAH1B1		1,043797754	17336000000
ELAV-like protein 1	ELAVL1		1,043667028	3048600000
Signal transducer and activator of transcription;Signal transducer and activator of transcription 5B	STAT5B		1,043634352	2496400000
Baculoviral IAP repeat-containing protein 6	BIRC6		1,043590787	4751700000
Symplekin	SYMPK;SYMPK variant protein		1,043579896	11499000000
Eukaryotic translation initiation factor 3 subunit E	EIF3S6;EIF3E		1,043547226	32776000000
Glutathione reductase, mitochondrial	GSR		1,04342745	3192100000
E3 ubiquitin-protein ligase TRIP12	TRIP12		1,043351244	2405000000
			1,043144455	37327000
Deubiquitinating protein VCIP135	VCPIP1		1,043122692	2925500000
COP9 signalosome complex subunit 4	COPS4		1,043024772	7837100000
Uroporphyrinogen decarboxylase	UROD		1,042981258	671960000
Protein SMG7	SMG7;SGA56M		1,042937747	31445000
Putative prolyl-tRNA synthetase associated domain-containing protein 1	PRORSD1P		1,042861612	630530000
60S ribosomal protein L26;60S ribosomal protein L26-like 1	RPL26;KRBA2;RPL26L1		1,042839862	59493000000
General transcription factor IIF subunit 2	GTF2F2		1,042785489	5364600000
Dipeptidyl peptidase 3	DKFZp686O1117;DPP3		1,042785489	5775200000
Lysosome membrane protein 2	SCARB2		1,04267676	241280000
Eukaryotic translation initiation factor 3 subunit A	EIF3A;eIF3a		1,042546315	95766000000
Serine/threonine-protein phosphatase 2A 56 kDa regulatory subunit alpha isoform	PPP2R5A		1,04245937	326400000
T-complex protein 1 subunit theta	CCT8		1,04232898	46083000000
DNA-directed RNA polymerase II subunit RPB7	POLR2G		1,042166038	723330000
Integrator complex subunit 9	INTS9		1,042090016	88367000
ATP-binding cassette sub-family F member 2	ABCF2		1,042024863	4404600000
Eukaryotic translation initiation factor 3 subunit M	EIF3M		1,041775185	22571000000
Ubiquitin carboxyl-terminal hydrolase isozyme L3	UCHL3		1,041764332	11188000000
Nardilysin	NRD1		1,041731775	4185500000
T-complex protein 1 subunit alpha	TCP1		1,041688369	49895000000
Filamin-C	FLNC		1,041623266	4923000000
Bifunctional UDP-N-acetylglucosamine 2-epimerase/N-acetylmannosamine kinase;UDP-N-acetylglucosamine 2-epimerase (	GNE		1,041536475	637270000
Polyadenylate-binding protein 1	PABPC1		1,041471391	44133000000
Phosphoserine phosphatase	PSPH		1,04131956	993280000

Ubiquitin-conjugating enzyme E2 G2	DKFZp586L2318;UBE2G2	1,041297874	258970000
Biliverdin reductase A	BLVRA	1,041254503	2711600000
HEAT repeat-containing protein 5A	HEATR5A	1,041200296	628330000
Eukaryotic translation initiation factor 3 subunit G	EIF3G;EIF3S4	1,041167774	8548100000
Elongation factor 1-alpha 2	EEF1A2	1,041026869	3904200000
Cleavage and polyadenylation specificity factor subunit 6	CPSF6	1,040810167	4294100000
Apoptotic protease-activating factor 1	APAF1	1,040777669	1026100000
Ras-related protein Rap-1A	RAP1A	1,040604383	2728400000
Gamma-aminobutyric acid receptor-associated protein	GABARAP	1,040485282	358980000
Pleiotropic regulator 1	PLRG1	1,040474456	664780000
WW domain-binding protein 2	WBP2	1,040452805	232810000
Heterogeneous nuclear ribonucleoproteins C1/C2	HNRNPC;hCG_1641229	1,040452805	9303500000
1-phosphatidylinositol 3-phosphate 5-kinase	PIKFYVE	1,040344562	212800000
Tight junction protein ZO-2	TJP2	1,040333739	4150100000
Translation initiation factor eIF-2B subunit beta	EIF2B2	1,040322916	1102100000
YEATS domain-containing protein 2	YEATS2	1,040301271	42949000
Ras-related protein Rab-8A	RAB8A	1,040149782	5246100000
Ankyrin repeat domain-containing protein 17	ANKRD17	1,040149782	3960000000
Espin	ESPN	1,040138963	835690000
Nitrilase homolog 1	NIT1	1,040117325	331700000
60S ribosomal protein L21	RPL21	1,040117325	8296300000
Guanine nucleotide-binding protein G(i) subunit alpha-2;Guanine nucleotide-binding protein G(i) subunit alpha-1	GNAI2;WUGSC:H_LUCA16.1;GNAI	1,040095689	21571000000
Liprin-alpha-2	PPFIA2;PPFIA2 variant protein	1,040009152	117730000
Conserved oligomeric Golgi complex subunit 8	COG8	1,039846935	220070000
DNA-directed RNA polymerases I, II, and III subunit RPABC5	POLR2L	1,039814497	438440000
Conserved oligomeric Golgi complex subunit 4	COG4	1,039717197	195050000
Adapter molecule crk	CRK	1,039609107	196580000
Prefoldin subunit 3	VBP1	1,03943621	6423900000
Cleavage and polyadenylation specificity factor subunit 3	CPSF3	1,039306575	1664500000
Rho guanine nucleotide exchange factor 2	ARHGEF2	1,039295773	3352500000
Transcription activator BRG1	SMARCA4	1,03920937	6297000000
Cytoskeleton-associated protein 5	CKAP5	1,039166173	5852200000
Transmembrane 9 superfamily member 3	SMBP;TM9SF3	1,038993423	4268100000
40S ribosomal protein S21	RPS21	1,038809939	8398300000
6-phosphofructo-2-kinase/fructose-2,6-bisphosphatase 3;6-phosphofructo-2-kinase;Fructose-2,6-bisphosphatase	PFKFB3	1,038809939	95285000
U2 small nuclear ribonucleoprotein A	SNRPA1	1,038777567	18212000000
Eukaryotic translation initiation factor 3 subunit H	EIF3S3;EIF3H	1,03862652	16492000000
Eukaryotic translation initiation factor 1A, X-chromosomal;Eukaryotic translation initiation factor 1A, Y-chromosomal	EIF1AX;EIF1AY	1,038594159	10452000000
GTP-binding protein 1	GTPBP1	1,038389251	1650500000
Peptidyl-prolyl cis-trans isomerase;Peptidyl-prolyl cis-trans isomerase F, mitochondrial	PPIF	1,038346122	2809100000
General transcription factor IIH subunit 4	GTF2H4	1,038227538	521590000
ATP-dependent RNA helicase DHX29	DHX29	1,03820598	1652600000
Rotatin	RTTN	1,038130535	7525400000
Striatin	STRN	1,038130535	433840000
PDZ and LIM domain protein 1	PDLIM1	1,038119757	2107300000
60S ribosomal protein L18	RPL18	1,037915036	26159000000
Uncharacterized protein C7orf26	C7orf26	1,037871947	295180000
Protein TSSC1	TSSC1	1,037861176	451050000
Protein MON2 homolog	MON2	1,037818091	1161900000
Sister chromatid cohesion protein PDS5 homolog B	PDS5B	1,037742702	5920800000
Cofilin-2	CFL2	1,037656556	42816000000
Heterogeneous nuclear ribonucleoprotein A/B	HNRNPAB	1,037462781	19176000000
HEAT repeat-containing protein 1;HEAT repeat-containing protein 1, N-terminally processed	HEATR1	1,037441255	429940000
COP9 signalosome complex subunit 1	GPS1	1,037355159	1813500000
Protein-lysine methyltransferase METTL21D	VCPKMT	1,037344398	268030000
Rho-related GTP-binding protein RhoC	RHOC	1,037312117	259730000
Nucleoprotein TPR	TPR	1,037301357	20558000000
Talin-1	TLN1	1,03724756	5,3203E+11
TSC22 domain family protein 2	TSC22D2	1,037226043	146020000
Serine/threonine-protein phosphatase PP1-beta catalytic subunit;Serine/threonine-protein phosphatase	PPP1CB	1,037204526	4169300000
Aminoacyl tRNA synthase complex-interacting multifunctional protein 1;Endothelial monocyte-activating polypeptide 2	AIMP1	1,037183011	6489800000
Nuclear factor NF-kappa-B p105 subunit;Nuclear factor NF-kappa-B p50 subunit	NFKB1	1,037172254	369030000
Methionine aminopeptidase 1	METAP1;DKFZp781C0419	1,037053937	1232100000
Splicing factor 3A subunit 3	SF3A3	1,037000166	3511500000
Translocation protein SEC63 homolog	SEC63	1,036978659	2885700000
CCA tRNA nucleotidyltransferase 1, mitochondrial	TRNT1	1,0369464	3478600000
Abl interactor 1	ABI1	1,036903392	191760000
60S ribosomal protein L35a	RPL35A	1,036838886	20232000000
	SEC22B	1,036709897	860910000
Methyltransferase-like protein 13	feat;METTL13	1,036613177	274490000
40S ribosomal protein S3	RPS3	1,036602432	1,9034E+11
Dynactin subunit 4	DCTN4	1,036559452	531910000
Ras-related GTP-binding protein A;Ras-related GTP-binding protein B	RRAGA;RRAGB	1,036280169	2379400000
Aldose reductase	AKR1B1	1,036258692	20402000000
GRIP and coiled-coil domain-containing protein 2	GCC2	1,036258692	81135000
Putative pre-mRNA-splicing factor ATP-dependent RNA helicase DHX16	DBP2;DHX16	1,036162056	357900000
E3 ubiquitin-protein ligase BRE1A	RNF20	1,036162056	1938900000
Probable ATP-dependent RNA helicase DDX23	DDX23	1,036001036	807960000
GPALPP motifs-containing protein 1	GPALPP1	1,035990303	56982000
SH3 domain-binding protein 1	SH3BP1	1,035958106	340970000
Cullin-4A	CUL4A	1,035915179	574660000
Histone deacetylase 2;Histone deacetylase	HDAC2	1,035915179	515210000
40S ribosomal protein S2	RPS2;rps2	1,035904448	1,1282E+11
		1,035872256	48003000
Ragulator complex protein LAMTOR4;Ragulator complex protein LAMTOR4, N-terminally processed	LAMTOR4	1,035797149	242290000
Chloride intracellular channel protein 1	CLIC1	1,035539723	52307000000
Ubiquitin thioesterase OTUB1	OTUB1;DKFZp564E242	1,035529	20298000000
Ubiquitin carboxyl-terminal hydrolase 16	USP16	1,035368177	103610000
Serine/arginine-rich splicing factor 5	SRSF5	1,035282425	4425800000
Kinectin	KTN1	1,035228837	2372600000
28 kDa heat- and acid-stable phosphoprotein	PDAP1	1,035207404	2497300000
Heterogeneous nuclear ribonucleoproteins A2/B1	HNRNPA2B1	1,035164539	48885000000
Rab-like protein 3	RABL3	1,035132393	332700000
40S ribosomal protein S4;40S ribosomal protein S4, X isoform	RPS4X	1,035014542	1,4619E+11
E3 ubiquitin-protein ligase AMFR	AMFR	1,034993117	85804000
60S ribosomal protein L7	RPL7	1,034971694	55210000000
Type 2 phosphatidylinositol 4,5-bisphosphate 4-phosphatase	TMEM55A	1,034896717	367430000
Mitochondrial import inner membrane translocase subunit Tim8 A	TIMM8A	1,034864588	1372400000
Exportin-1	XPO1	1,034832461	23414000000
Elongation factor Tu GTP-binding domain-containing protein 1	EFTUD1	1,034811044	1011300000
Heterogeneous nuclear ribonucleoprotein D0	HNRNPD	1,034768212	25757000000
5-3 exoribonuclease 1	XRN1	1,034736091	720550000
Amidophosphoribosyltransferase	PPAT	1,034725384	4300100000
Prenylcysteine oxidase 1	PCYOX1	1,034703971	146350000
Rab GDP dissociation inhibitor alpha	GDI1	1,034382887	1190600000
Transformation/transcription domain-associated protein	TRRAP	1,034340091	7215300000
Dipeptidyl peptidase 9	DPP9	1,034265206	436080000
Chromosome transmission fidelity protein 18 homolog	RUVBL;CHTF18	1,034233116	115470000

Cullin-1	CUL1	1,034019233	7713900000
Metaxin-2	MTX2	1,033997849	1783600000
Hippocalcin-like protein 1	HPCAL1;DKFZp781K1922	1,033976467	831800000
Required for meiotic nuclear division protein 1 homolog	RMND1	1,033944394	441920000
Arf-GAP with SH3 domain, ANK repeat and PH domain-containing protein 1	ASAP1	1,033880256	70390000
Myosin-14	MYH14	1,033837501	8856000000
F-actin-capping protein subunit beta	CAPZB	1,033837501	49597000000
Nicastrin	NCSTN	1,033741317	1024500000
Protein phosphatase methylesterase 1	PPME1	1,033741317	2224400000
Eukaryotic translation initiation factor 3 subunit L	EIF3L	1,033719945	19747000000
RNA polymerase II subunit A C-terminal domain phosphatase	CTDP1	1,03347423	685390000
ER membrane protein complex subunit 3	EMC3	1,033431509	2874600000
Ras-related protein Rab-31	RAB31	1,033367434	273930000
ADP-ribosylation factor-binding protein GGA1	GGA1	1,033303368	147860000
Glomulin	GLMN	1,033196607	158980000
N-acetylgalactosaminyltransferase 7	GALNT7	1,033185932	232490000
Prefoldin subunit 6	PFDN6;HKE2	1,033121888	2110200000
Thioredoxin, mitochondrial	TXN2	1,033004494	1238700000
Eukaryotic translation initiation factor 3 subunit I	EIF3S2;EIF3I	1,032961811	27906000000
Protein LSM14 homolog A	LSM14A	1,032919133	544680000
Nurim	NRM	1,032130214	362670000
T-complex protein 1 subunit zeta	CCT6A	1,032119561	41148000000
U6 snRNA-associated Sm-like protein LSm3	LSM3	1,032044997	1710900000
Protein disulfide-isomerase;Protein disulfide-isomerase A3	PDIA3	1,032013045	39349000000
Katanin p60 ATPase-containing subunit A-like 2	KATNAL2	1,031981094	4484300000
DNA-directed RNA polymerase II subunit RPB3	POLR2C	1,031917199	1214900000
40S ribosomal protein S8	RPS8	1,031853312	1,1193E+11
Probable 18S rRNA (guanine-N(7))-methyltransferase	WBSCR22	1,031842665	315110000
Ubiquitin-like modifier-activating enzyme 1	UBA1	1,031672341	77403000000
Serine/threonine-protein kinase PAK 2;PAK-2p27;PAK-2p34	PAK2	1,031651054	5909100000
Guanine nucleotide-binding protein G(I)/G(S)/G(T) subunit beta-1	GNB1	1,031608484	11233000000
	FP3184	1,031565917	43186000
ARF GTPase-activating protein GIT2	GIT2	1,031512713	493000000
WD repeat-containing protein 82	WDR82	1,031416961	3856300000
Zinc phosphodiesterase ELAC protein 2	ELAC2	1,031353135	688080000
CGG triplet repeat-binding protein 1	CGGBP1	1,031278682	1208300000
Eukaryotic translation initiation factor 2 subunit 1	EIF2S1	1,031182973	47553000000
Cleavage and polyadenylation specificity factor subunit 7	CPSF7	1,031161707	766320000
	FBXO31	1,03107665	113800000
COMM domain-containing protein 9	COMMD9	1,030895952	708160000
Mitochondrial import inner membrane translocase subunit Tim10 B	FXC1;TIMM10B	1,030832191	199970000
60S ribosomal protein L36a;60S ribosomal protein L36a-like	RPL36A;RPL36A-HNRNPH2;RPL3(	1,030821565	11539000000
Prolyl endopeptidase	PREP	1,030694069	13027000000
Heat shock 70 kDa protein 4	HSPA4;HS24/p52	1,030683446	44818000000
Inhibitor of nuclear factor kappa-B kinase subunit alpha	CHUK	1,030672823	613090000
E3 ubiquitin-protein ligase ARIH1	ARIH1	1,030577226	862650000
Mitochondrial import inner membrane translocase subunit Tim17-A	TIMM17A;TIM17A	1,030439173	171900000
Elongation factor G, mitochondrial	GFM1	1,03040732	683670000
Adenylate kinase 2, mitochondrial;Adenylate kinase 2, mitochondrial;Adenylate kinase 2, mitochondrial, N-terminally process	AK2	1,030269312	14268000000
Chromodomain-helicase-DNA-binding protein 3	CHD3	1,030195016	329060000
CTP synthase 2	CTPS2	1,03017379	236350000
Wiskott-Aldrich syndrome protein family member 2	WASF2	1,030067675	240300000
Choline/ethanolamine kinase	CHKB	1,030014626	179360000
Eukaryotic initiation factor 4A-III;Eukaryotic initiation factor 4A-III, N-terminally processed	EIF4A3	1,029950974	11293000000
WD repeat domain phosphoinositide-interacting protein 3	WDR45L;WDR45B	1,029940366	389800000
GSK3-beta interaction protein	GSKIP	1,029929759	68075000
Charged multivesicular body protein 1a	CHMP1A	1,029919151	875220000
Proliferation-associated protein 2G4	PA2G4	1,02988733	1,0327E+11
GTP-binding nuclear protein Ran	RAN	1,029855511	1,7594E+11
Conserved oligomeric Golgi complex subunit 3	COG3	1,029717651	193740000
Spectrin alpha chain, non-erythrocytic 1	SPTAN1	1,029707048	56179000000
DNA primase;DNA primase small subunit	PRIM1	1,02967524	173580000
Zinc finger CCCH domain-containing protein 4	ZC3H4	1,029643434	422860000
DNA-directed RNA polymerase;DNA-directed RNA polymerase III subunit RPC2	DKFZp686D10173;POLR3B	1,029484434	125900000
A-kinase anchor protein 10, mitochondrial	AKAP10	1,029484434	23463000
Pantothenate kinase 4	PANK4	1,029463238	149680000
Actin-related protein 2/3 complex subunit 1A	ARPC1A	1,02945264	331640000
5-nucleotidase domain-containing protein 3	NT5DC3	1,02920895	116040000
Mannosyl-oligosaccharide glucosidase	GCS1;MOGS	1,02920895	2286400000
Ataxin-2	ATXN2	1,02920895	265100000
6-phosphogluconate dehydrogenase, decarboxylating	PGD	1,029198357	22931000000
F-box only protein 22	FBXO22	1,029177173	486920000
Mitogen-activated protein kinase 14	MAPK14	1,028848924	6286400000
Saccharopine dehydrogenase-like oxidoreductase	SCCPDH	1,028774832	208600000
Zinc finger CCCH domain-containing protein 18	ZC3H18	1,028721916	186320000
COP9 signalosome complex subunit 5	COPS5	1,028658424	8544400000
WD repeat-containing protein 1	WDR1	1,028658424	8678500000
26S protease regulatory subunit 10B	PSMC6	1,028594939	25143000000
E3 ubiquitin-protein ligase UBR5	UBR5	1,028584359	3786400000
Heterogeneous nuclear ribonucleoprotein A3	HNRPA3;HNRNPA3;D10S102	1,028552621	3321400000
Terminal uridylyltransferase 7	ZCCHC6	1,028531463	239180000
Coiled-coil domain-containing protein 47	CCDC47	1,028499727	2047900000
Kinesin-like protein KIF7	KIF7	1,028499727	277620000
Annexin;Annexin A11	ANXA11	1,028489149	1990600000
Dr1-associated corepressor	DRAP1	1,028415109	4019400000
Peptidyl-prolyl cis-trans isomerase FKBP5	FKBP5	1,028288209	549100000
Ubiquitin-like modifier-activating enzyme 5	UBA5	1,02822477	87834000
Bridging integrator 3	BIN3	1,028108486	79547000
Ras-related protein R-Ras2	RRAS2	1,027685857	1585300000
CDKN2A-interacting protein	CDKN2AIP	1,027590813	198690000
Rho GTPase-activating protein 17	ARHGAP17	1,027495787	498600000
Ubiquinone biosynthesis protein COQ9, mitochondrial	COQ9	1,02742189	1228900000
Cytokine receptor-like factor 3	CRLF3	1,027284681	754160000
Ras-related protein Rap-2c	RAP2C	1,027063113	936690000
Ribonucleases P/MRP protein subunit POP1	POP1	1,026989278	233820000
Catechol O-methyltransferase domain-containing protein 1	COMTD1	1,026947092	64017000
Copine-1	CPNE1	1,026926	2646400000
Rho-related GTP-binding protein RhoG	ARHG;RHOG	1,026831097	23016000000
C-Jun-amino-terminal kinase-interacting protein 4	SPAG9	1,026799466	2570800000
Twinfilin-1	TWF1	1,026767838	3849600000
Actin, cytoplasmic 2;Actin, cytoplasmic 2, N-terminally processed	ACTG1	1,026757295	1,7989E+12
	OK/KNS-cl.6	1,026715128	412660000
Trafficking protein particle complex subunit 9	TRAPPC9	1,026620263	60727000
Condensin complex subunit 3	NCAPG	1,026578107	2215300000
AP-3 complex subunit mu-1	AP3M1;DKFZp586G1518	1,026535954	2362500000
Alpha-1,6-mannosyl-glycoprotein 2-beta-N-acetylglucosaminyltransferase	MGAT2	1,026483268	40658000
Elongation factor 1-delta	EEF1D	1,026451659	57400000000
Tetratricopeptide repeat protein 37	TTC37	1,026409517	537970000

Serine/threonine-protein kinase VRK1	VRK1	1,026325242	1853800000
Caspase recruitment domain-containing protein 9	CARD9	1,02628311	234590000
DENN domain-containing protein 4C	DENND4C	1,026156735	252500000
Integrator complex subunit 7	INTS7	1,026114617	495000000
Alpha-actinin-4	ACTN4	1,026061974	8009900000
Survival motor neuron protein	SMN1;SMN2	1,026040919	300620000
Phospholipid-transporting ATPase IG	ATP11C	1,025946179	31099000
Pyruvate kinase PKM;Pyruvate kinase	PKM;PKM2	1,025809364	218090000
DNA ligase;DNA ligase 1	LIG1	1,025767274	3743000000
S-phase kinase-associated protein 1	SKP1	1,025651545	4548500000
Transmembrane protein 68	TMEM68	1,025577913	339160000
Ubiquitin conjugation factor E4 B	UBE4B	1,025462227	2700700000
60S ribosomal protein L37a	RPL37A	1,025104817	12602000000
Splicing factor 3B subunit 5	SF3B5	1,024947215	1340800000
Ragulator complex protein LAMTOR1	LAMTOR1	1,024810666	1653900000
60S ribosomal protein L28	RPL28	1,024695153	25066000000
26S protease regulatory subunit 8	PSMC5	1,024527181	24232000000
Malectin	MLEC	1,024485196	8401600000
U3 small nucleolar ribonucleoprotein protein IMP3	IMP3	1,024443215	89066000
Hsp70-binding protein 1	HSPBP1	1,024443215	5657100000
Microtubule-actin cross-linking factor 1, isoforms 1/2/3/5	MACF1	1,024443215	1165900000
Clathrin light chain B	CLTB	1,024443215	1413400000
Mediator of RNA polymerase II transcription subunit 27	MED27	1,024390744	243380000
Eukaryotic translation initiation factor 3 subunit D	EIF3D	1,02434877	10670000000
Dynactin subunit 2	DCTN2	1,024201891	5726900000
Serine/threonine-protein phosphatase;Serine/threonine-protein phosphatase 5	PPP5C	1,024170422	1100500000
Developmentally-regulated GTP-binding protein 1	DRG1	1,023929226	16602000000
Ribosome-releasing factor 2, mitochondrial	GFM2	1,023855841	87421000
Charged multivesicular body protein 3	CHMP3	1,023730063	1567700000
Structural maintenance of chromosomes protein 1A	SMC1A;DKFZp686L19178	1,023646228	23115000000
Leucine-rich repeat serine/threonine-protein kinase 1	LRRK1	1,023583361	80831000
		1,023562407	227430000
PCI domain-containing protein 2	PCID2	1,023541453	904410000
Bleomycin hydrolase	BLMH	1,023530977	3064300000
UPF0690 protein C1orf52	C1orf52	1,023510025	173490000
UBX domain-containing protein 1	UBXN1	1,023290082	2769100000
cAMP-dependent protein kinase type II-alpha regulatory subunit	PRKAR2A	1,023059767	323410000
Heat shock protein HSP 90-beta	HSP90AB1	1,023049301	5,2735E+11
60S ribosomal protein L34	RPL34	1,023017903	14749000000
tRNA (adenine(58)-N(1))-methyltransferase non-catalytic subunit TRM6	TRMT6	1,02285048	647750000
U6 snRNA-associated Sm-like protein LSm2	LSM2	1,022756328	2782000000
40S ribosomal protein S10;Putative 40S ribosomal protein S10-like	RPS10;RPS10P5	1,022672653	10041000000
Negative elongation factor B	NELFB	1,022672653	907640000
Serine/threonine-protein kinase TBK1	TBK1	1,022317184	234810000
14-3-3 protein sigma	SFN	1,022296282	29886000000
Phosphoglycolate phosphatase	PGP	1,02224403	1517500000
Dynactin subunit 5	DCTN5	1,022212682	527760000
Catenin alpha-3	CTNNA3	1,022003741	616520000
Eukaryotic translation initiation factor 3 subunit K	EIF3K	1,021909745	13170000000
Dihydrofolate reductase, mitochondrial	DHFRL1	1,021878417	1420800000
Peroxiredoxin-4	PRDX4	1,021742684	19670000000
Eukaryotic translation initiation factor 2 subunit 3;Putative eukaryotic translation initiation factor 2 subunit 3-like protein	EIF2S3;EIF2S3L	1,021690489	20127000000
UDP-N-acetylhexosamine pyrophosphorylase;UDP-N-acetylgalactosamine pyrophosphorylase;UDP-N-acetylglucosamine p	UAP1	1,021533935	734180000
Zinc finger protein 830	ZNF830	1,021460893	18404000
RNA-binding protein EWS	EWSR1	1,021293979	4554300000
Basic leucine zipper and W2 domain-containing protein 2	BZW2	1,02109584	11668000000
Immediate early response 3-interacting protein 1	IER3IP1	1,021085414	1846900000
ER membrane protein complex subunit 4	EMC4	1,021054136	1095100000
Iron-responsive element-binding protein 2	IREB2	1,020887355	3532000000
Mitochondrial fission 1 protein	FIS1	1,020845669	2153300000
Serine/threonine-protein kinase 10	STK10	1,020772725	805690000
Dynamin-2	DNM2	1,02059562	9395800
Uncharacterized protein C19orf18	C19orf18	1,020574788	208080000
Nuclear protein localization protein 4 homolog	NPLOC4	1,020574788	1286100000
Sphingosine-1-phosphate lyase 1	SGPL1	1,020553957	54945000
RalBP1-associated Eps domain-containing protein 1	REPS1	1,020449814	489940000
Importin-9	IPO9	1,020387339	6103600000
	MDN1	1,020356104	124420000
Histone H2A type 2-C;Histone H2A type 2-A	HIST2H2AC;HIST2H2AA3	1,020252002	678250000
Protein argonaute-2;Protein argonaute	AGO2;EIF2C2	1,020220776	1345800000
Caspase-3;Caspase-3 subunit p17;Caspase-3 subunit p12	CASP3	1,020220776	6975600000
Protein EVI2B	EVI2B	1,020168736	191630000
Cytochrome b-c1 complex subunit 10	UQCR11	1,020106295	269280000
ARF GTPase-activating protein GIT1	GIT1	1,020023053	444270000
Serine/threonine-protein phosphatase 6 catalytic subunit;Serine/threonine-protein phosphatase 6 catalytic subunit, N-termina	PPP6C	1,019950226	6362600000
26S protease regulatory subunit 7	PSMC2	1,019929421	28341000000
Aspartate--tRNA ligase, cytoplasmic	DARS	1,019835806	24289000000
Solute carrier family 2, facilitated glucose transporter member 14;Solute carrier family 2, facilitated glucose transporter memb	SLC2A14;SLC2A3	1,019763007	4004900000
7SK snRNA methylphosphate capping enzyme	MEPCE	1,019742209	78855000
Pyrroline-5-carboxylate reductase;Pyrroline-5-carboxylate reductase 3	PYCRL	1,019669423	1922700000
RWD domain-containing protein 4	RWDD4	1,019627836	424650000
Probable ATP-dependent RNA helicase DHX40	DHX40	1,019357601	82141000
V-type proton ATPase subunit d 2	ATP6V0D2	1,019316039	374910000
3-hydroxyisobutyryl-CoA hydrolase, mitochondrial	HIBCH	1,019305649	1241800000
Methylosome subunit pICln	CLNS1A	1,019170599	1539900000
DnaJ homolog subfamily B member 4	DNAJB4	1,019139439	172440000
Peroxisomal membrane protein PEX13	PEX13	1,019118666	39528000
Thioredoxin-interacting protein	TXNIP	1,019097895	1203600000
Nuclear pore complex protein Nup88	NUP88	1,019087509	990840000
Formin-like protein 1	FMNL1	1,019045969	3368800000
E3 ubiquitin-protein ligase UBR4	UBR4	1,019014816	22376000000
Adenosylhomocysteinase;Putative adenosylhomocysteinase 2	AHCYL1	1,0189629	1785400000
TOX high mobility group box family member 4;Thymocyte selection-associated high mobility group box protein TOX;TOX hig	TOX4;Bbtx1;TOX;TOX3	1,018900606	122320000
Polycomb group RING finger protein 6	PCGF6	1,018838321	31215000
Polyadenylate-binding protein 4	PABPC4	1,018786422	4127600000
Serine/threonine-protein phosphatase 6 regulatory subunit 3	PPP6R3	1,018620381	7860900000
Protein FRG1;Protein FRG1B	FRG1;FRG1B	1,018589254	1574300000
Pre-mRNA-splicing factor 38A	PRPF38A	1,018392162	422680000
U4/U6 small nuclear ribonucleoprotein Prp31	PRPF31	1,018340309	1819100000
ADP-ribosylation factor-like protein 6	ARL6	1,01829883	206690000
Ubiquitin-like modifier-activating enzyme ATG7	ATG7	1,018008572	825260000
High mobility group protein B1;Putative high mobility group protein B1-like 1	HMGB1;HMGB1P1	1,017925671	89320000000
Probable ATP-dependent RNA helicase DDX46	DDX46	1,017925671	14165000000
Ras-related protein Rap-2a	RAP2A	1,017915309	257200000
Vacuolar protein sorting-associated protein 33A	VPS33A	1,017842784	261380000
Plectin	PLEC	1,017770269	26177000000
Endophilin-B2	SH3GLB2	1,017708121	290190000
Ubiquitin-fold modifier-conjugating enzyme 1	UFC1	1,017687407	4559200000

ATP-dependent RNA helicase DDX42	DDX42	1,017604559	4882900000
Insulin-degrading enzyme	IDE	1,017594204	16001000000
Heat shock protein HSP 90-alpha	EL52;HSP90AA1	1,017594204	2,0717E+11
Dehydrogenase/reductase SDR family member 2, mitochondrial	DHRS2	1,01756314	40563000
COP9 signalosome complex subunit 3	COPS3	1,017552786	6612300000
Coiled-coil domain-containing protein 144A;Putative coiled-coil domain-containing protein 144C	CCDC144A;CCDC144CP	1,017532078	394180000
Glutamine-rich protein 1	QRICH1	1,017521724	85562000
14-3-3 protein gamma;14-3-3 protein gamma, N-terminally processed	YWHAG	1,017469959	78448000000
Erlin-2	ERLIN2	1,017428551	6612500000
Serine/threonine-protein phosphatase PP1-alpha catalytic subunit;Serine/threonine-protein phosphatase	PPP1CA	1,017397497	37079000000
Cullin-associated NEDD8-dissociated protein 1	CAND1	1,017397497	43948000000
14-3-3 protein zeta/delta	YWHAZ	1,017397497	2,1501E+11
DNA-dependent protein kinase catalytic subunit	PRKDC	1,017376796	928370000
Probable cytosolic iron-sulfur protein assembly protein CIAO1	CIAO1	1,017335395	966640000
Rho guanine nucleotide exchange factor 7	ARHGEF7;KIAA0142	1,017304347	182400000
Protein arginine N-methyltransferase 1	PRMT1;HRMT1L2	1,017262952	40593000000
GRIP1-associated protein 1	GRIPAP1	1,017252604	55637000
ATP-dependent RNA helicase DDX39A	DDX39A;DDX39	1,017221561	6760900000
26S proteasome non-ATPase regulatory subunit 11	PSMD11	1,017200867	36180000000
Importin-4	IPO4	1,017097407	1297900000
Anamorsin	CIAPIN1	1,017014655	833410000
DnaJ homolog subfamily C member 2;DnaJ homolog subfamily C member 2, N-terminally processed	DNAJC2	1,0169526	2156200000
Alpha-centractin	ACTR1A	1,016869871	11465000000
Chloride intracellular channel protein 4	CLIC4	1,016756141	28409000000
Putative RNA-binding protein Luc7-like 2	LUC7L2	1,016714791	12650000000
Small acidic protein	C11orf58;SMAP	1,016683781	461050000
Catechol O-methyltransferase	COMT	1,016683781	491670000
General transcription factor IIH subunit 1	GTF2H1	1,016570093	61429000
		1,016549425	58704000
N-alpha-acetyltransferase 15, NatA auxiliary subunit	NAA15;NARG1	1,016549425	15314000000
E3 ubiquitin-protein ligase CHIP	STUB1	1,016549425	1482100000
Phospholipase A-2-activating protein	PLAA	1,016549425	2876400000
TATA box-binding protein-like protein 1	TBPL1	1,016394442	1468700000
UPF0668 protein C10orf76	C10orf76	1,016280819	124050000
Calmodulin	CALM1;CALM2;CALM3	1,016218853	15986000000
Polypeptide N-acetylgalactosaminyltransferase 2;Polypeptide N-acetylgalactosaminyltransferase 2 soluble form	GALNT2	1,016177547	52301000
Protein Simiate	FAM206A	1,01616722	144350000
Geranylgeranyl transferase type-2 subunit alpha	RABGGTA	1,016115593	276990000
Integrator complex subunit 10	INTS10	1,016105269	289890000
Secretory carrier-associated membrane protein 3	SCAMP3	1,016043324	800570000
Vacuolar protein sorting-associated protein 4B	VPS4B	1,015960743	2522100000
Deoxyhypusine synthase	DHPS	1,015919458	562110000
60S ribosomal protein L36	RPL36	1,015878176	12014000000
Kelch-like protein 9	KLHL9;DKFZp686L0695	1,015867856	26433000
Integrator complex subunit 3	INTS3	1,015764668	1419200000
Ubiquitin-conjugating enzyme E2 N	UBE2N	1,0156615	23031000000
26S proteasome non-ATPase regulatory subunit 14	PSMD14	1,015651185	13937000000
CCR4-NOT transcription complex subunit 3	CNOT3	1,015558354	219750000
Elongator complex protein 2	ELP2	1,015413984	189030000
Vesicular integral-membrane protein VIP36	LMAN2	1,015341815	16153000000
Etoposide-induced protein 2.4 homolog	EI24	1,015290271	82236000
Vesicle transport protein GOT1B	GOLT1B	1,015279963	2461700000
Potassium-transporting ATPase alpha chain 2	ATP12A	1,015269656	645710000
Interferon-inducible double-stranded RNA-dependent protein kinase activator A	PRKRA	1,015259348	556590000
Protein RER1	RER1	1,015238733	579150000
ER membrane protein complex subunit 1	EMC1	1,015228426	5906400000
Phosphofurin acidic cluster sorting protein 1	PACS1	1,01505324	1357400000
Mitochondrial import receptor subunit TOM22 homolog	MST065;TOMM22	1,014929615	1261300000
Protein FAM168A	FAM168A	1,014888413	155670000
PDZ domain-containing protein GIPC2	GIPC2	1,014641274	260110000
Coiled-coil domain-containing protein 43	CCDC43	1,014630979	1023500000
Essential MCU regulator, mitochondrial	SMDT1	1,01461039	60352000
NEDD8-activating enzyme E1 regulatory subunit	NAE1	1,014600095	1294600000
Adipocyte plasma membrane-associated protein	APMAP	1,014579508	1621200000
Protein SMG9	SMG9	1,014507457	125490000
OTU domain-containing protein 4	OTUD4	1,014486873	693670000
40S ribosomal protein S9	RPS9	1,014363386	1,1352E+11
60S ribosomal protein L32	RPL32	1,014342807	22097000000
Ribulose-phosphate 3-epimerase	RPE	1,014250216	629850000
Tumor protein p63-regulated gene 1-like protein	TPRG1L	1,014167926	300670000
E3 ubiquitin-protein ligase HERC2	HERC2	1,014126786	171920000
Vigilin	HDLBP	1,01408565	33189000000
Ribosomal protein S6 kinase;Ribosomal protein S6 kinase alpha-4	RPS6KA4	1,013951979	222560000
26S protease regulatory subunit 6A	PSMC3	1,01384918	13488000000
V-type proton ATPase subunit C 1	ATP6V1C1	1,01378751	3872500000
Costars family protein ABRACL	ABRACL	1,013633369	1264200000
Tyrosine-protein phosphatase non-receptor type;Tyrosine-protein phosphatase non-receptor type 1	PTPN1	1,01361282	326190000
Immunity-related GTPase family Q protein	IRGQ	1,013540907	23246000
Activated RNA polymerase II transcriptional coactivator p15	PC4;SUB1	1,013530634	1754000000
Condensin-2 complex subunit D3	NCAPD3	1,013510089	193470000
Diphosphoinositol polyphosphate phosphohydrolase 1	NUDT3	1,013469003	403310000
Nuclear receptor corepressor 2	NCOR2	1,01339711	147190000
Eukaryotic translation initiation factor 3 subunit B	EIF3B;EIF3S9	1,013335495	21399000000
Protein CutA	CUTA	1,013284155	141160000
40S ribosomal protein S24	RPS24	1,013212288	23517000000
Golgi apparatus protein 1	GLG1	1,013150696	10053000000
Methyltransferase-like protein 5	METTL5	1,013089111	82878000
Disks large homolog 1	DLG1	1,013037796	853710000
Intron-binding protein aquarius	AQR	1,012914662	2565000000
Histone chaperone ASF1A	ASF1A	1,012791557	1182700000
Desumoylating isopeptidase 1	DESI1	1,012688993	49846000
Translation initiation factor eIF-2B subunit delta	EIF2B4;DKFZp586J0119	1,012606957	1705400000
Acidic leucine-rich nuclear phosphoprotein 32 family member E	ANP32E	1,012535186	15909000000
26S protease regulatory subunit 4	PSMC1	1,012514681	9760100000
Ras-related protein Rab-11B;Ras-related protein Rab-11A	RAB11B;RAB11A	1,012494178	34820000000
Cyclin-dependent kinase 4	CDK4	1,012381425	307520000
Syntaxin-6	STX6	1,012371176	376150000
40S ribosomal protein S29	RPS29	1,012330182	756590000
Mitochondrial fission factor	MFF	1,012319934	317230000
Tubulin beta-4B chain;Tubulin beta-4A chain	TUBB2C;TUBB4B;TUBB4A	1,012186728	1,7485E+11
SWI/SNF-related matrix-associated actin-dependent regulator of chromatin subfamily D member 2	SMARCD2	1,012176483	499740000
Small glutamine-rich tetratricopeptide repeat-containing protein alpha	SGTA	1,012115017	1556900000
GDP-fucose protein O-fucosyltransferase 2	POFUT2	1,012104773	276800000
Centrosome-associated protein CEP250	CEP250	1,012043315	400340000
DNA polymerase beta	POLB	1,012002348	510430000
Pre-rRNA-processing protein TSR1 homolog	TSR1	1,012002348	1058600000
COMM domain-containing protein 10	COMMD10	1,011992106	404530000
DNA polymerase;DNA polymerase alpha catalytic subunit	POLA1	1,011951143	1168600000

40S ribosomal protein S16	RPS16	1,011869226	64739000000
Leucine-rich repeat-containing protein 59	LRRC59	1,011746375	17166000000
Protein phosphatase 1 regulatory subunit 15A	PPP1R15A	1,011613321	268710000
Integrator complex subunit 8	INTS8	1,011613321	306610000
Tripartite motif-containing protein 4	TRIM4	1,011562155	11327000000
Electron transfer flavoprotein-ubiquinone oxidoreductase, mitochondrial	ETFDH	1,011541691	339500000
Ras-related protein Rab-35	RAB35	1,011398461	2942500000
EF-hand domain-containing protein D2	EFHD2	1,011388231	11305000000
Ethanolamine-phosphate cytidylyltransferase	PCYT2	1,011296178	718980000
Arf-GAP with Rho-GAP domain, ANK repeat and PH domain-containing protein 1	ARAP1	1,011275724	540670000
Golgi reassembly-stacking protein 2	GORASP2	1,011234819	517310000
Myomegalin	PDE4DIP	1,011193917	21393000
Guanylate kinase	DKFZp666D023;GUK1	1,011142794	1131700000
Mitochondrial import receptor subunit TOM34	TOMM34	1,011122346	1311800000
UPF0696 protein C11orf68	C11orf68	1,010948573	1815200000
Dehydrogenase/reductase SDR family member 7	DKFZp564H1664;DHRS7	1,010897475	724250000
Glutaredoxin-3	GLRX3	1,010825946	10630000000
Small integral membrane protein 20	SMIM20	1,010764643	39645000
Cytosolic Fe-S cluster assembly factor NUBP2	NUBP2;NUBP1	1,010723779	1139300000
40S ribosomal protein S19	RPS19	1,010662489	56948000000
Rap1 GTPase-GDP dissociation stimulator 1	RAP1GDS1	1,010642061	1435300000
Mitogen-activated protein kinase;Mitogen-activated protein kinase 3	MAPK3;DKFZp686O0215	1,010631847	581060000
Dihydropteridine reductase	QDPR	1,010560356	4218300000
Serine/threonine-protein kinase 4;Serine/threonine-protein kinase 4 37kDa subunit;Serine/threonine-protein kinase 4 18kDa s	STK4;DKFZp686A2068	1,010519508	896320000
3(2),5-bisphosphate nucleotidase 1	BPNT1	1,010488875	826810000
Serine/threonine-protein phosphatase 1 regulatory subunit 10	PPP1R10	1,010448033	354750000
		1,010315319	295820000
60S ribosomal protein L3	RPL3;rpl3	1,010254079	50026000000
SAM and SH3 domain-containing protein 3	SASH3	1,010049997	530880000
Protein FAM60A	FAM60A	1,009988789	37680000
C-terminal-binding protein 1	CTBP1	1,009937788	6305700000
SUMO-activating enzyme subunit 2	UBA2	1,009896991	10677000000
Alcohol dehydrogenase class 4 mu/sigma chain	ADH7	1,009784815	3548400000
Nuclear pore complex protein Nup133	NUP133	1,009764422	40525000
2,5-phosphodiesterase 12	PDE12	1,009733834	193740000
ER lumen protein-retaining receptor 1;ER lumen protein-retaining receptor	KDELR1	1,009713443	1043000000
Tyrosine-protein kinase JAK1	JAK1	1,009703248	539120000
Protein FAM49B	FAM49B;DKFZp686B04128	1,009652276	19012000000
Syntenin-1	SDCBP	1,009580923	2716100000
von Willebrand factor A domain-containing protein 9	VWA9	1,009570731	171960000
tRNA (cytosine(34)-C(5))-methyltransferase	NSUN2	1,009448437	11644000000
Serine/threonine-protein kinase N1	PKN1	1,009295613	1652400000
T-complex protein 1 subunit gamma	CCT3	1,00922431	79168000000
Protein pelota homolog	PELO	1,009000283	485300000
Coiled-coil domain-containing protein 6	CCDC6	1,008857771	88797000
Synembryn-A	RIC8A	1,008827238	98611000
Protein SEC13 homolog	SEC13	1,008796707	3994300000
Chromodomain-helicase-DNA-binding protein 1	CHD1	1,008664427	131500000
Cell division control protein 42 homolog	CDC42	1,008583042	30398000000
Calcium uniporter protein, mitochondrial	MCU	1,008471158	2747200000
Dihydrofolate reductase	DYR;DHFR	1,008389803	2534900000
Ubiquitin-40S ribosomal protein S27a;Ubiquitin;40S ribosomal protein S27a;Polyubiquitin-B;Ubiquitin;Polyubiquitin-C;Ubiquitin	RPS27A;HEL112;UBB;UBC;UbC;DI	1,008328796	1154500000
Stathmin	STMN1	1,008277962	10411000000
Putative 60S ribosomal protein L39-like 5;60S ribosomal protein L39	RPL39P5;RPL39	1,00825763	2501100000
Kanadaptin	SLC4A1AP	1,008064516	328200000
Alcohol dehydrogenase [NADP(+)]	AKR1A1	1,008054354	8862500000
Protein bicaudal D homolog 2	BICD2	1,007973067	220940000
Cap-specific mRNA (nucleoside-2-O-)-methyltransferase 1	CMTR1	1,007942588	484840000
Spectrin alpha chain, non-erythrocytic 1	SPTAN1	1,00791211	60160000
Transmembrane protein 263	TMEM263	1,007810532	2004700000
Biogenesis of lysosome-related organelles complex 1 subunit 2	BLOC1S2	1,007637895	67130000
Puromycin-sensitive aminopeptidase	NPEPPS	1,007576979	5539300000
Phosphoglucomutase-2	PGM2	1,007556675	2915400000
Periodic tryptophan protein 1 homolog	PWP1	1,007414571	104010000
Cell division cycle protein 16 homolog	CDC16	1,007373978	388640000
Trafficking protein particle complex subunit 6B	TRAPPC6B	1,007252216	612160000
Heterogeneous nuclear ribonucleoprotein F;Heterogeneous nuclear ribonucleoprotein F, N-terminally processed	HNRNPF	1,007160914	22852000000
Serine/threonine-protein phosphatase PP1-gamma catalytic subunit;Serine/threonine-protein phosphatase	PPP1CC	1,007120341	1391400000
Poly(A) RNA polymerase, mitochondrial	MTPAP;GOLGA2;DKFZp686K0319	1,007029063	95304000
Surfeit locus protein 4	SURF4	1,007029063	10250000000
Charged multivesicular body protein 6	CHMP6	1,006907385	153970000
Bridging integrator 2	BIN2	1,006816145	2135600000
Cytosolic acyl coenzyme A thioester hydrolase	ACOT7	1,006653983	12330000000
Ribosomal protein S6 kinase;Ribosomal protein S6 kinase alpha-1	RPS6KA1	1,006623583	482800000
Serine/threonine-protein phosphatase 6 regulatory ankyrin repeat subunit B	ANKRD44;LOC91526	1,006502003	81514000
Transketolase	TKT	1,006420966	49919000000
Upstream-binding protein 1	UBP1	1,006339942	149150000
Down syndrome critical region protein 3	DSCR3	1,006329815	146130000
Dynein light chain 1, cytoplasmic;Dynein light chain 2, cytoplasmic	DNCL1;DYNLL1;DYNLL2	1,006329815	4589800000
Probable ATP-dependent RNA helicase DDX20	DDX20	1,006299434	600080000
Serine/threonine-protein kinase TAO3	TAOK3	1,006015976	1777800000
Nuclear autoantigenic sperm protein	NASP	1,005894542	5899400000
Alpha-actinin-3	ACTN3	1,005854071	1387400000
Signal transducer and activator of transcription;Signal transducer and activator of transcription 6	STAT6	1,005823719	240740000
Zinc finger CCCH domain-containing protein 11A	ZC3H11A	1,005732676	326380000
CAP-Gly domain-containing linker protein 1	CLIP1	1,005520306	2023600000
RING finger protein 219	RNF219	1,005439427	197130000
Eukaryotic translation initiation factor 6	EIF6	1,00529792	11831000000
U5 small nuclear ribonucleoprotein 200 kDa helicase	SNRNP200	1,005247391	29977000000
26S proteasome non-ATPase regulatory subunit 8	PSMD8	1,005004925	7753700000
Coiled-coil domain-containing protein 25	CCDC25	1,004833248	2427200000
Putative heat shock protein HSP 90-beta 4	HSP90AB4P	1,004823151	1821700000
Signal transducer and activator of transcription 3;Signal transducer and activator of transcription	STAT3	1,004762575	2753900000
Docking protein 3	DOK3	1,004762575	79945000
Proteasome subunit beta type;Proteasome subunit beta type-6	PSMB6	1,004459802	8297400000
Bifunctional lysine-specific demethylase and histidyl-hydroxylase MINA	MINA	1,004369005	124200000
BRISC and BRCA1-A complex member 1	BABAM1	1,00431857	958130000
pre-mRNA 3 end processing protein WDR33	WDR33	1,004288311	1133100000
60S ribosomal protein L18a	RPL18A	1,004278225	33750000000
Phosphatidylinositol 5-phosphate 4-kinase type-2 beta	PIP4K2B	1,004046307	47941000
Trafficking protein particle complex subunit 8	TRAPPC8;KIAA1012	1,004046307	515800000
Ubiquitin-60S ribosomal protein L40;Ubiquitin;60S ribosomal protein L40	UBA52	1,004005984	1,5938E+11
Cilia- and flagella-associated protein 20	CFAP20	1,003935427	1878800000
Programmed cell death protein 10	PDCD10	1,003602935	9567800000
NEDD4-binding protein 1	N4BP1	1,003562647	84526000
40S ribosomal protein S11	RPS11	1,003532434	72443000000
Serine/threonine-protein kinase MST4	RBM4B;MST4	1,003482083	3157300000
3-phosphoinositide-dependent protein kinase 1;Putative 3-phosphoinositide-dependent protein kinase 2	PDPK1;PkB-like 2;PkB-like 1;PDPK2	1,003381395	204580000

Cleavage and polyadenylation specificity factor subunit 5	NUDT21		1,003351193	15811000000
Ubiquitin carboxyl-terminal hydrolase 24	USP24		1,00330086	2284200000
Cell differentiation protein RCD1 homolog	RQCD1		1,003200209	2318200000
Caseinolytic peptidase B protein homolog	CLPB		1,003039209	203910000
Rho GTPase-activating protein 30	ARHGAP30		1,003029148	642890000
Epidermal growth factor receptor substrate 15	EPS15		1,002978847	1538700000
Glutamate--cysteine ligase catalytic subunit	GCLC		1,002968788	509160000
Trafficking protein particle complex subunit 11	TRAPPC11		1,002918493	612520000
Transitional endoplasmic reticulum ATPase	VCP;DKFZp434K0126		1,002908434	1,0938E+11
4F2 cell-surface antigen heavy chain	SLC3A2		1,002908434	2923700000
Cleavage stimulation factor subunit 3	CSTF3		1,002827975	1387500000
Ubiquitin-protein ligase E3C	UBE3C		1,002546468	1579200000
Calcineurin subunit B type 1	PPP3R1		1,002536417	204040000
Diphthamide biosynthesis protein 1	DPH1		1,002486166	219060000
	BCR/ABL fusion		1,002224939	5717700000
40S ribosomal protein S23	RPS23		1,002154632	44632000000
Testis-expressed sequence 10 protein	TEX10		1,002134547	253720000
Integrator complex subunit 1	INTS1;DKFZP586J0619		1,002074294	11707000000
N-alpha-acetyltransferase 25, NatB auxiliary subunit	NAA25		1,002024089	1661600000
Eukaryotic translation initiation factor 5A-1;Eukaryotic translation initiation factor 5A-1-like	EIF5A;EIF5AL1		1,001963849	1,0269E+11
Mediator of RNA polymerase II transcription subunit 30	MED30		1,001883541	269060000
Signal recognition particle 9 kDa protein	SRP9;DKFZp564M2223		1,001833355	755520000
Phosphatidylinositol transfer protein beta isoform	PITPNB		1,001803246	4008300000
Alpha-mannosidase 2	MAN2A1		1,001763103	585070000
Septin-2		02-Sep	1,001642694	22885000000
Prefoldin subunit 2	PFDN2		1,001532344	4434900000
COMM domain-containing protein 2	COMMD2		1,001502253	1498600000
Histidine--tRNA ligase, cytoplasmic	HARS;HRS		1,001472164	7485700000
Tyrosine-protein phosphatase non-receptor type 7	PTPN7		1,001331771	1634300000
Ubiquitin-conjugating enzyme E2 variant 2	UBE2V2		1,001211466	11030000000
Cytochrome c oxidase assembly factor 7	COA7		1,001191418	121150000
Squamous cell carcinoma antigen recognized by T-cells 3	SART3		1,001171371	3665000000
Protein FAM160B1;Protein FAM160B2	FAM160B1;FAM160B2		1,001091189	48445000
ERO1-like protein alpha	ERO1L		1,001001001	10972000000
Tubulin alpha-1C chain	TUBA1C;KLK9		1,000950903	1230200000
Conserved oligomeric Golgi complex subunit 1	ADCK1;COG1		1,000840706	192880000
TELO2-interacting protein 1 homolog	KIAA0406;TTI1		1,000720519	26794000
Peroxiredoxin-2	PRDX2		1,000720519	3921000000
40S ribosomal protein S15a	hCG_1994130;RPS15A		1,000680463	38095000000
			1,00060036	24204000000
Protein phosphatase 1 regulatory subunit 12;Protein phosphatase 1 regulatory subunit 12A	PPP1R12A		1,000380144	347280000
AP-1 complex subunit sigma-1A	AP1S1		1,000160026	810830000
	ACT		0,99990001	1402900000
Thioredoxin reductase 1, cytoplasmic	GML;TXNRD1		0,99990001	2207300000
Eukaryotic translation initiation factor 3 subunit C;Eukaryotic translation initiation factor 3 subunit C-like protein	EIF3C;EIF3CL		0,99970009	25296000000
60S ribosomal protein L22	RPL22		0,99970009	18401000000
26S proteasome non-ATPase regulatory subunit 6	PSMD6		0,99970009	26420000000
ATP-dependent Clp protease ATP-binding subunit clpX-like, mitochondrial	CLPX;DKFZp586J151		0,99950025	600970000
Rho GTPase-activating protein 31	ARHGAP31		0,99950025	168610000
Spermidine synthase	SRM		0,99940036	18468000000
C-1-tetrahydrofolate synthase, cytoplasmic;Methylenetetrahydrofolate dehydrogenase;Methenyltetrahydrofolate cyclohydrol	MTHFD1		0,99930049	10319000000
PAB-dependent poly(A)-specific ribonuclease subunit PAN3	PAN3		0,999100809	34824000
THO complex subunit 2	THOC2		0,999000999	2684900000
Calponin-2	CNN2		0,998901209	406460000
Ovarian cancer-associated gene 2 protein	OVCA2		0,998701688	694150000
	PSMA3		0,998701688	73547000
Neurolysin, mitochondrial	NLN		0,998502247	119550000
COP9 signalosome complex subunit 6	COPS6		0,998502247	6658800000
Ran-binding protein 10	RANBP10		0,998502247	35442000
Zinc finger matrin-type protein 2	ZMAT2		0,998402556	119340000
Maestro heat-like repeat-containing protein family member 1	MROH1		0,998302885	283060000
Calnexin	CANX		0,998302885	19908000000
Rab GDP dissociation inhibitor beta	GDI2		0,998302885	50337000000
Rab3 GTPase-activating protein catalytic subunit	RAB3GAP1		0,998302885	1064500000
Nucleolar transcription factor 1	UBTF		0,998203234	1185300000
Mitogen-activated protein kinase 9;Mitogen-activated protein kinase 10	MAPK9;MAPK10		0,998103603	366500000
Coronin;Coronin-1C	CORO1C		0,998103603	2944000000
40S ribosomal protein S15	RPS15		0,998003992	11775000000
SWI/SNF complex subunit SMARCC2	SMARCC2		0,998003992	2764200000
Protein PRRC1	PRRC1		0,997904401	910040000
40S ribosomal protein S18	RPS18		0,997904401	80983000000
UDP-N-acetylhexosamine pyrophosphorylase-like protein 1	UAP1L1		0,997904401	291020000
ER membrane protein complex subunit 2	EMC2		0,997804829	3073400000
AP-2 complex subunit beta	AP2B1;DKFZp781K0743		0,997605746	17208000000
T-complex protein 1 subunit alpha	TCP1		0,997506234	85647000
AP-2 complex subunit mu	AP2M1		0,99730727	4476500000
Liprin-beta-1	PPFIBP1		0,99730727	290330000
E3 ubiquitin-protein ligase UBR2	UBR2		0,99730727	567000000
Tubulin alpha-1B chain	TUBA1B		0,997207818	2,0919E+11
Splicing factor, proline- and glutamine-rich	SFPQ		0,997008973	33313000000
Structural maintenance of chromosomes protein;Structural maintenance of chromosomes protein 2	SMC2		0,99690958	7913100000
Peroxiredoxin-1	PRDX1		0,996710854	70916000000
DNA mismatch repair protein Msh2	MSH2		0,996611521	2115700000
Acetyl-CoA carboxylase 2;Biotin carboxylase	ACACB		0,996512207	286550000
Condensin complex subunit 1	NCAPD2		0,996512207	4583400000
Thioredoxin-related transmembrane protein 4	TMX4		0,996412914	488630000
Putative phospholipase B-like 2;Putative phospholipase B-like 2 32 kDa form;Putative phospholipase B-like 2 45 kDa form	PLBD2		0,99631364	860430000
Bis(5-nucleosyl)-tetraphosphatase [asymmetrical]	NUDT2		0,99631364	756910000
DNA polymerase delta subunit 2	POLD2		0,996115151	777740000
	ANKRD54		0,995916741	147420000
			0,995916741	239400000
Nuclear export mediator factor NEMF	NEMF		0,995817566	1232300000
Acyl-coenzyme A thioesterase 9, mitochondrial	ACOT9		0,995619275	608780000
Protocadherin Fat 3	FAT3		0,995619275	2273000000
Cyclin-dependent kinase 12	CDK12		0,995520159	308290000
Actin, alpha cardiac muscle 1;Actin, gamma-enteric smooth muscle;Actin, aortic smooth muscle	ACTC1;ACTA2;ACTG2		0,995520159	65740000000
Ras-related protein Ral-B	RALB		0,995321987	405260000
Extended synaptotagmin-2	ESYT2		0,995321987	1384500000
General transcription factor IIE subunit 1	GTF2E1		0,99522293	125740000
THO complex subunit 5 homolog	THOC5		0,995123893	297010000
Heterogeneous nuclear ribonucleoprotein L	HNRNPL		0,995123893	2162100000
Argininosuccinate lyase	ASL		0,994925878	2717600000
U6 snRNA-associated Sm-like protein LSm4	LSM4		0,994925878	1309000000
N-alpha-acetyltransferase 10	NAA10		0,994431185	4194800000
Muskelin	MKLN1		0,994431185	74145000
Charged multivesicular body protein 1b	CHMP1B		0,994431185	1021900000
Enoyl-CoA delta isomerase 2, mitochondrial	ECI2;HCA64;PECI		0,994332306	137680000
TNF receptor-associated factor 6	TRAF6		0,994332306	10535000

ERI1 exoribonuclease 3	PRNPIP;ERI3	0,994332306	170560000
Pachytene checkpoint protein 2 homolog	TRIP13	0,994332306	2899900000
Proline-serine-threonine phosphatase-interacting protein 1	PSTPIP1	0,994332306	286850000
Integrator complex subunit 5	INTS5	0,994233446	580680000
Multifunctional methyltransferase subunit TRM112-like protein	TRMT112	0,994035785	1369900000
GMP reductase;GMP reductase 2	GMPR2	0,993936984	1534100000
Inosine triphosphate pyrophosphatase	ITPA	0,993739442	5574700000
39S ribosomal protein L43, mitochondrial	MRPL43	0,9936407	1225900000
Putative deoxyribonuclease TATDN1	TATDN1	0,9936407	236430000
EKC/KEOPS complex subunit TPRKB	TPRKB	0,993541977	243390000
Centrosomal protein of 170 kDa	CEP170	0,993443274	247100000
Heterogeneous nuclear ribonucleoprotein U	HNRNPU;HNRPU	0,993443274	26935000000
Choline/ethanolaminephosphotransferase 1	CEPT1	0,993245928	1002800000
Glycine--tRNA ligase	GARS	0,993245928	20022000000
N-alpha-acetyltransferase 38, NatC auxiliary subunit	NAA38	0,993245928	178650000
O-phosphoseryl-tRNA(Sec) selenium transferase	SEPSECS	0,993147284	160590000
AP-1 complex subunit beta-1	AP1B1	0,993048659	4226900000
Fatty aldehyde dehydrogenase;Aldehyde dehydrogenase;Aldehyde dehydrogenase family 3 member B2;Aldehyde dehydrog	ALDH3A2;ALDH3B2;ALDH3A1;DKF	0,992950055	318990000
Proteasome assembly chaperone 4	PSMG4	0,992950055	196700000
Neurobeachin-like protein 2	NBEAL2	0,992851469	2261900000
SHC-transforming protein 1	SHC1	0,992752904	930380000
Sodium/potassium-transporting ATPase subunit alpha-3	ATP1A3	0,992654358	1910700000
CD2-associated protein	CD2AP	0,992654358	286110000
RNA polymerase-associated protein CTR9 homolog	CTR9	0,992654358	656120000
FACT complex subunit SPT16	SUPT16H	0,992555831	9392400000
26S proteasome non-ATPase regulatory subunit 2	PSMD2;DKFZp564A2282	0,992555831	27823000000
Ubiquitin carboxyl-terminal hydrolase 34	USP34	0,992555831	1270700000
Proline-rich AKT1 substrate 1	AKT1S1	0,992457324	59122000
Ran-specific GTPase-activating protein	RANBP1	0,992358837	21266000000
Endoplasmic reticulum aminopeptidase 1	ERAP1	0,992260369	384140000
THO complex subunit 7 homolog	THOC7	0,992260369	86968000
E3 ubiquitin-protein ligase HUWE1	HUWE1	0,992260369	21436000000
Ras-related protein Rab-6B	RAB6B	0,992063492	1624400000
Vacuolar protein sorting-associated protein 28 homolog	VPS28	0,992063492	732410000
ESF1 homolog	ESF1	0,992063492	430860000
Kaptin	KPTN	0,991965083	181760000
RNA-binding protein with serine-rich domain 1	RNPS1	0,991965083	811340000
Heterogeneous nuclear ribonucleoprotein M	HNRNPM;ORF	0,991965083	18951000000
Heterogeneous nuclear ribonucleoprotein H3	HNRNPH3	0,991965083	136410000
Titin	TTN;DKFZp451N061	0,991866693	379510000
26S proteasome non-ATPase regulatory subunit 3	PSMD3	0,991866693	10914000000
Protein kinase C and casein kinase substrate in neurons protein 2	PACSIN2	0,991866693	1395300000
SUMO-activating enzyme subunit 1;SUMO-activating enzyme subunit 1, N-terminally processed	SAE1	0,991768323	14479000000
Coatomer subunit gamma-2;Coatomer subunit gamma	COPG2	0,991768323	2718600000
Phosphatidylinositol 4-kinase type 2-alpha	PI4K2A	0,991669972	174670000
Negative elongation factor C/D	TH1L;NELFCD	0,991669972	374670000
Alcohol dehydrogenase class-3	ADH5	0,991473329	10811000000
COMM domain-containing protein 6	COMMD6	0,991375037	41508000
Protein-associating with the carboxyl-terminal domain of ezrin	SCYL3	0,991375037	155040000
Transportin-3	TNPO3	0,991178511	2319300000
Dynactin subunit 3	DCTN3	0,990982063	583680000
COMM domain-containing protein 1	C2orf5;COMMD1	0,990883868	237930000
ATP synthase subunit d, mitochondrial	ATP5H	0,990785693	899570000
U4/U6.U5 tri-snRNP-associated protein 1	SART1	0,990785693	555470000
Syntaxin-binding protein 2	STXBP2;ZNF14	0,990785693	2877000000
26S proteasome non-ATPase regulatory subunit 13	PSMD13	0,990687537	16300000000
Translation initiation factor eIF-2B subunit gamma	EIF2B3	0,990687537	771280000
C2 domain-containing protein 5	KIAA0528;C2CD5	0,990589401	585810000
	HZGJ;WDR92	0,990589401	1810200000
26S proteasome non-ATPase regulatory subunit 4	PSMD4	0,990589401	5924300000
Prefoldin subunit 4	PFDN4	0,990589401	953450000
MKL/myocardin-like protein 2;Phosphatase and actin regulator	MKL2	0,990197049	80058000
Ras-related GTP-binding protein C;Ras-related GTP-binding protein D	RRAGC;RRAGD	0,98990299	2756300000
Activity-dependent neuroprotector homeobox protein	ADNP	0,98990299	190020000
Destrin	DSTN	0,989805008	8228600000
MAGUK p55 subfamily member 6	MPP6	0,989805008	799900000
T-complex protein 1 subunit epsilon	CCT5	0,989805008	551180000
Nucleosome assembly protein 1-like 4	NAP1L4	0,989805008	1294100000
Elongator complex protein 3	ELP3	0,989805008	746680000
Serine/arginine-rich splicing factor 1	SRSF1	0,989805008	12374000000
		0,989609104	15625000000
Mitotic spindle assembly checkpoint protein MAD1	MAD1L1	0,989609104	69954000
Phosphoribosyl pyrophosphate synthase-associated protein 1	PRPSAP1	0,989609104	4326300000
Alpha-(1,6)-fucosyltransferase	FUT8	0,989511181	1453100000
NADH dehydrogenase [ubiquinone] 1 alpha subcomplex subunit 3	NDUFA3	0,989413278	208570000
Ezrin;Tyrosine-protein kinase receptor	EZR;EZR-ROS1	0,989315394	3524200000
B-cell CLL/lymphoma 7 protein family member C;B-cell CLL/lymphoma 7 protein family member B;B-cell CLL/lymphoma 7 pro	BCL7C;BCL7B;BCL7A	0,989315394	250690000
THUMP domain-containing protein 3	THUMPD3	0,989217529	169350000
Rho GDP-dissociation inhibitor 1	ARHGDIA	0,989119683	62869000000
Coiled-coil-helix-coiled-coil-helix domain-containing protein 5	CHCHD5	0,988924051	59903000
Jouberin	AHI1	0,988826263	27988000
40S ribosomal protein S27;40S ribosomal protein S27-like	RPS27L	0,988728495	3895400000
WASH complex subunit FAM21C;WASH complex subunit FAM21A;WASH complex subunit FAM21B	FAM21A;FAM21C;DKFZp434O171;	0,988630746	257060000
Uridine-cytidine kinase 2	UCK2	0,988533017	1539200000
Reticulocalbin-2	RCN2	0,988533017	577570000
Golgin subfamily A member 4	GOLGA4	0,988239945	70966000
		0,988239945	151890000
STE20-like serine/threonine-protein kinase	SLK	0,988239945	10813000000
Mediator of RNA polymerase II transcription subunit 12	MED12;TNRC11	0,988239945	723820000
Non-POU domain-containing octamer-binding protein	NONO	0,988239945	8839900000
Protein diaphanous homolog 3	DIAPH3	0,988142292	84029000
Proteasome subunit alpha type-5;Proteasome subunit alpha type	PSMA5	0,988142292	45065000000
Peptidyl-prolyl cis-trans isomerase;FK506-binding protein 15	FKBP15	0,988142292	1330800000
Cytoplasmic dynein 1 light intermediate chain 1	DYNC1LI1;DKFZp686A1525	0,988142292	1888600000
116 kDa U5 small nuclear ribonucleoprotein component	EFTUD2;U5-116KD;SNRP116	0,98804466	13965000000
MORC family CW-type zinc finger protein 2	MORC2	0,987947046	514080000
EH domain-containing protein 1	EHD1	0,987947046	3235500000
Hermansky-Pudlak syndrome 1 protein	HPS1	0,987751877	1724500000
Thioredoxin domain-containing protein 12	TXNDC12	0,987654321	3046600000
Ragulator complex protein LAMTOR2	LAMTOR2	0,987654321	626330000
Histone deacetylase 4	HDAC4;MGC16025	0,987556785	27417000
Syntaxin-4	STX4;STX4A	0,987459267	780370000
Anaphase-promoting complex subunit 1	ANAPC1	0,987459267	1074500000
Protein FAM114A2	FAM114A2	0,987459267	543390000
Tubulin beta-8 chain	TUBB8	0,987459267	2435900000
Proteasome assembly chaperone 1	PSMG1	0,987361769	1718200000
Ras suppressor protein 1	RSU1	0,987361769	7570200000
von Willebrand factor A domain-containing protein 5A	VWA5A	0,987264291	720630000

Glia maturation factor beta	GMFB	0,987264291	4730300000
	HCLS1	0,987166831	1830900000
Clathrin heavy chain 1	CLTC	0,987166831	2,028E+11
		0,987069391	17227000000
Zinc finger protein 330	ZNF330	0,98697197	315900000
ADP-ribosylation factor-like protein 1	ARL1	0,986874568	6935900000
Beta-centractin	ACTR1B	0,986777186	1638900000
Ras-related protein Rab-5A	RAB5A	0,986777186	2453700000
Activating signal cointegrator 1 complex subunit 2	ASCC2	0,986679822	183410000
Voltage-gated potassium channel subunit beta-2	KCNAB2	0,986582478	2307600000
Cocaine esterase	CES2	0,986485153	935140000
Epididymis-specific alpha-mannosidase	MAN2B2	0,986387848	212710000
Cleavage stimulation factor subunit 1	CSTF1	0,986290561	1798500000
ADP-sugar pyrophosphatase	NUDT5	0,986290561	1297500000
Acetoacetyl-CoA synthetase	AACS	0,986193294	722130000
Macrophage erythroblast attacher	MAEA	0,986096046	628540000
Transportin-2	TNPO2 variant protein;TNPO2	0,986096046	2400000000
Peroxisomal membrane protein PEX16	PEX16	0,985998817	1225600000
Cohesin subunit SA-1	STAG1 variant protein;STAG1;DKFZ	0,985998817	36854000
Prostaglandin E synthase 3	PTGES3	0,985998817	24905000000
SAFB-like transcription modulator	SLTM	0,985804416	96099000
Oxysterol-binding protein 1;Oxysterol-binding protein	OSBP	0,985707245	4827000000
Mediator of RNA polymerase II transcription subunit 16	MED16	0,985512959	142370000
Paired amphipathic helix protein Sin3a	SIN3A	0,985512959	3214800000
DNA mismatch repair protein Msh6	MSH6;GTBP	0,985512959	5063200000
DnaJ homolog subfamily B member 11	DNAJB11	0,985415845	9865600000
Ataxin-3	ATXN3	0,985415845	365330000
X-ray repair cross-complementing protein 6	XRCC6	0,985415845	396930000
Aminoacyl tRNA synthase complex-interacting multifunctional protein 2	AIMP2	0,985415845	7751200000
UDP-glucose 4-epimerase	GALE	0,985221675	340090000
U1 small nuclear ribonucleoprotein A	SNRPA	0,984833563	1486000000
ADP-ribosylation factor-like protein 8A	ARL8A	0,984833563	2085600000
Ras GTPase-activating protein 1	RASA1	0,984736583	1628600000
Ubiquinone biosynthesis protein COQ4 homolog, mitochondrial	COQ4	0,98454268	102300000
Transforming acidic coiled-coil-containing protein 1	TACC1;DKFZp686K18126	0,98454268	121440000
Emerin	EMD	0,98454268	426870000
26S proteasome non-ATPase regulatory subunit 1	PSMD1	0,984445757	23764000000
COMM domain-containing protein 7	COMMD7	0,984348853	916790000
Guanine nucleotide-binding protein-like 1	HSR1;GNL1	0,984348853	443440000
COP9 signalosome complex subunit 7b	COPS7B	0,984058256	985230000
Mitogen-activated protein kinase kinase kinase 7	MAP3K7	0,984058256	126960000
AP-2 complex subunit alpha-2	AP2A2	0,984058256	3588700000
Formin-binding protein 1	FNBP1	0,984058256	907150000
Serum paraoxonase/lactonase 3	PON3	0,984058256	547670000
Programmed cell death protein 4	PDCD4	0,983961429	693630000
Methylmalonyl-CoA mutase, mitochondrial	MUT	0,98386462	347500000
Translin	TSN	0,983767831	18947000000
Syntaxin-7	STX7	0,983767831	1744300000
Kinase suppressor of Ras 1	KSR1	0,98367106	118100000
Large neutral amino acids transporter small subunit 1	SLC7A5;lat1	0,98367106	4021800000
	HSP90Bf	0,98367106	284300000
Pre-mRNA-processing-splicing factor 8	PRPF8	0,983574309	22409000000
Protein lin-7 homolog C;Protein lin-7 homolog A	LIN7C;LIN7A	0,983574309	1513400000
RNA-binding protein 12	DKFZp667H197;RBM12	0,983574309	2425600000
Structural maintenance of chromosomes protein;Structural maintenance of chromosomes protein 4	SMC4	0,983574309	8886300000
Vesicle-fusing ATPase	NSF	0,983574309	20471000000
Bcl-2-binding component 3	BBC3	0,983477577	303020000
Supervillin	SVIL	0,983380863	313470000
DET1- and DDB1-associated protein 1	DDA1	0,983284169	70433000
p21-activated protein kinase-interacting protein 1	PAK1IP1	0,983284169	329990000
Serine/threonine-protein phosphatase 4 regulatory subunit 3B	SMEK2	0,983090838	1342800000
Phosphoribosylformylglycinamidine synthase	PFAS	0,9829942	12573000000
Arginine and glutamate-rich protein 1	ARGLU1	0,982897582	1926100000
4-trimethylaminobutyraldehyde dehydrogenase	ALDH9A1	0,982607841	3659500000
COMM domain-containing protein 5	COMMD5	0,982607841	82529000
SAGA-associated factor 29 homolog	CCDC101	0,982607841	69498000
Stress-70 protein, mitochondrial	HSPA9	0,982318271	84515000000
SUN domain-containing protein 2	SUN2;UNC84B	0,981932443	299130000
UPF0554 protein C2orf43	C2orf43	0,981932443	108920000
Histone acetyltransferase KAT7	KAT7	0,981932443	18480000
Metastasis-associated protein MTA1	MTA1	0,981739643	374440000
Malonyl-CoA decarboxylase, mitochondrial	MLYCD	0,981643271	139520000
Dystonin	DST	0,981643271	142600000
A-kinase anchor protein 9	AKAP9	0,981450584	1908900000
Protein transport protein Sec31A	SEC31A	0,981450584	4780800000
Tubulin beta-2B chain	TUBB2B;DKFZp566F223	0,981354269	1549900000
Ubiquitin conjugation factor E4 A	UBE4A	0,981257973	1563000000
Serine/threonine-protein kinase Nek9	NEK9	0,981257973	477660000
RNA-binding motif protein, X chromosome;RNA-binding motif protein, X chromosome, N-terminally processed;RNA binding m	RBMX;RBMXL1	0,981257973	3592200000
Nuclear pore complex protein Nup160	NUP160	0,981065437	1567000000
26S proteasome non-ATPase regulatory subunit 12	PSMD12	0,981065437	6159500000
Dynein light chain Tctex-type 1	DYNLT1	0,980969198	572010000
UPF0505 protein C16orf62	C16orf62	0,980969198	1056400000
Probable E3 ubiquitin-protein ligase HERC4	HERC4	0,980776775	2312400000
26S protease regulatory subunit 6B	PSMC4	0,980776775	24229000000
Mimitin, mitochondrial	NDUFAF2	0,980680592	47874000
6-phosphogluconolactonase	PGLS	0,980680592	12160000000
Proteasome assembly chaperone 3	PSMG3	0,980680592	1688200000
Ras-related protein Rab-1B	RAB1B	0,980488283	13126000000
Protein virilizer homolog	KIAA1429	0,980488283	207780000
LETM1 and EF-hand domain-containing protein 1, mitochondrial	LETM1	0,980296049	1094600000
Cell division cycle and apoptosis regulator protein 1	CCAR1	0,980296049	6483300000
Phosphatidylserine decarboxylase proenzyme;Phosphatidylserine decarboxylase alpha chain;Phosphatidylserine decarboxy	PISD	0,980199961	84188000
Peptidyl-prolyl cis-trans isomerase A;Peptidyl-prolyl cis-trans isomerase A, N-terminally processed	PPIA	0,980199961	524000000
Thioredoxin domain-containing protein 5	TXNDC5;STRF8;DKFZp666I134	0,980103891	2074700000
Serine/threonine-protein kinase WNK1	WNK1	0,979911808	1472400000
Histone-lysine N-methyltransferase SMYD3	SMYD3	0,979911808	320680000
Ras-related protein Rab-4A	RAB4A	0,979815795	188030000
Phenylalanine--tRNA ligase alpha subunit	FARSLA;FARSA	0,979815795	2119000000
NEDD8-conjugating enzyme Ubc12	UBE2M	0,979623824	13141000000
DNA-directed RNA polymerase;DNA-directed RNA polymerase II subunit RPB2	POLR2B	0,979623824	4745600000
Kinetochore-associated protein 1	KNTC1	0,979623824	71350000
Acylpyruvase FAHD1, mitochondrial	FAHD1	0,97933601	364710000
Nicalin	NCLN	0,97924011	1357300000
Ubiquitin-conjugating enzyme E2 L3	UBE2L3	0,97924011	8189500000
MIP18 family protein FAM96A	FAM96A	0,979144228	140160000
V-type proton ATPase subunit B, brain isoform	ATP6V1B2	0,979048365	6152800000
CCR4-NOT transcription complex subunit 1	CNOT1	0,978856695	18509000000

Heat shock 70 kDa protein 14	HSPA14	0,978856695	1414200000
26S proteasome non-ATPase regulatory subunit 9	PSMD9	0,978856695	1753000000
Serine/threonine-protein phosphatase PGAM5, mitochondrial	PGAM5	0,978665101	708610000
Nesprin-1	SYNE1	0,978665101	1232000000
SAP domain-containing ribonucleoprotein	CIP29;SARNP;LOC402290	0,978569332	5791400000
Thimet oligopeptidase	THOP1	0,978473581	3169800000
Coiled-coil domain-containing protein 134	CCDC134	0,97837785	392510000
Acidic fibroblast growth factor intracellular-binding protein	FIBP	0,97837785	709770000
Glucose-6-phosphate 1-dehydrogenase	G6PD	0,978090767	7012400000
Translation initiation factor eIF-2B subunit alpha	EIF2B1	0,978090767	1930700000
Snurportin-1	SNUPN	0,97799511	243770000
AP-2 complex subunit alpha-1	AP2A1	0,97799511	8739100000
WASH complex subunit 7	KIAA1033	0,977899472	1344100000
Phenylalanine--tRNA ligase beta subunit	FARSB;FARSLB	0,977803853	6296900000
WD repeat-containing protein 3	WDR3	0,977708252	48135000
Putative adenosylhomocysteinase 3;Adenosylhomocysteinase	AHCYL2	0,977708252	6328800000
Epidermal growth factor receptor substrate 15-like 1	EPS15L1;SPATA22	0,977421562	238920000
Protein AHNAK2	AHNAK2	0,977326036	1703500000
40S ribosomal protein S30	FAU	0,977230529	5315400000
Sacsin	SACS	0,977230529	50524000
Serine palmitoyltransferase 3	SPTLC3	0,97703957	53586000
Transmembrane protein 33	SHINC3;TMEM33	0,97703957	4600400000
Myotrophin	DKFZp761E1322;MTPN	0,976944119	2936400000
E3 ubiquitin-protein ligase RNF114	RNF114;ZNF313	0,976944119	611210000
Histone-arginine methyltransferase CARM1	CARM1	0,976848686	2090600000
Cyclic AMP-dependent transcription factor ATF-1	EWSR1/ATF1 fusion;CREB1;CREM	0,976753272	221960000
Nucleobindin-1	NUCB1	0,976657877	3719800000
Trafficking protein particle complex subunit 12	TRAPPC12;CGI-87	0,976467142	150300000
mRNA cap guanine-N7 methyltransferase	RNMT	0,976371802	1085500000
Clathrin light chain A	CLTA	0,976276481	4266100000
Differentially expressed in FDCP 6 homolog	DEF6	0,97599063	608610000
Protein phosphatase 1A	PPM1A	0,97599063	883170000
V-type proton ATPase subunit G 1;V-type proton ATPase subunit G 2	ATP6V1G1;ATP6V1G2-DDX39B;AT	0,975800156	1474300000
La-related protein 1	LARP1	0,975800156	7457600000
GTP-binding protein SAR1a	SAR1A;SARA1	0,975609756	5722700000
Coatomer subunit epsilon	COPE	0,975609756	402440000
Signal recognition particle 54 kDa protein	SRP54	0,97541943	6004100000
Protein Niban	FAM129A	0,975324295	2842700000
	EEF1A	0,975229179	214760000
TFIIH basal transcription factor complex helicase XPB subunit	ERCC3	0,975229179	236690000
Vesicle transport protein SEC20	BNIP1	0,975134081	1146900000
Neutral cholesterol ester hydrolase 1	NCEH1	0,975134081	569250000
Multifunctional protein ADE2;Phosphoribosylaminoimidazole-succinocarboxamide synthase;Phosphoribosylaminoimidazole c	PAICS	0,975134081	15321000000
Choline-phosphate cytidylyltransferase A	PCYT1A;hCG_2002711	0,974848898	614090000
Serine/arginine-rich splicing factor 2	SRSF2;SFRS2	0,974848898	17980000000
Wings apart-like protein homolog	WAPAL	0,974373965	1527400000
26S proteasome non-ATPase regulatory subunit 7	PSMD7	0,974373965	20041000000
Phosphate carrier protein, mitochondrial	SLC25A3	0,974184121	53033000000
V-type proton ATPase subunit H	ATP6V1H	0,974089227	1101700000
Serine/arginine repetitive matrix protein 2	SRRM2;KIAA0324	0,973994351	4638100000
RNA polymerase-associated protein RTF1 homolog	RTF1	0,973994351	586540000
Protein transport protein Sec24C	SEC24C	0,973994351	5989200000
Ras-related protein Rab-1A	RAB1A	0,973994351	56794000000
Glutaredoxin-related protein 5, mitochondrial	GLRX5	0,973804655	1098200000
Translin-associated protein X	TSNAX	0,973804655	1994300000
EH domain-containing protein 4	EHD4	0,973709834	2853700000
Disabled homolog 2-interacting protein	DAB2IP	0,973615033	295230000
Eukaryotic peptide chain release factor GTP-binding subunit ERF3A;Eukaryotic peptide chain release factor GTP-binding su	GSPT1;GSPT2	0,973520249	14617000000
Signal peptidase complex subunit 1	SPCS1	0,973425484	431110000
Pre-mRNA-processing factor 6	PRPF6	0,973425484	832620000
Integrin-linked kinase-associated serine/threonine phosphatase 2C	ILKAP;ILKAP3	0,973425484	3840000000
Septin-7	SEPT7;Nbla02942;DKFZp686F172	0,973330738	17264000000
Phosphoribosyl pyrophosphate synthase-associated protein 2	PRPSAP2	0,973330738	7611100000
Proteasome subunit alpha type-1;Proteasome subunit alpha type	PSMA1	0,97323601	46597000000
General vesicular transport factor p115	USO1	0,97323601	7380100000
CAAX prenyl protease 1 homolog	ZMPSTE24	0,9731413	2419200000
Proteasome subunit alpha type-7;Proteasome subunit alpha type	PSMA7	0,973046609	45502000000
Integrator complex subunit 6	INTS6	0,973046609	175060000
Nck-associated protein 1-like	NCKAP1L	0,972951936	3139300000
Rho GTPase-activating protein 5	ARHGAP5	0,972951936	204020000
Serine/threonine-protein phosphatase 6 regulatory subunit 2	PPP6R2	0,972951936	645050000
UPF0556 protein C19orf10	C19orf10	0,972857282	1578600000
Exportin-7	XPO7	0,972857282	4984100000
Sodium/potassium-transporting ATPase subunit beta-3	ATP1B3	0,972668028	403850000
Vacuolar-sorting protein SNF8	SNF8	0,972668028	1964200000
Transmembrane protein 147	TMEM147	0,972573429	64742000
ATP-dependent RNA helicase DHX8	DHX8	0,972573429	138130000
Protein disulfide-isomerase A5	PDIA5	0,972384286	281500000
Tubulin gamma-1 chain;Tubulin gamma-2 chain	TUBG1;TUBG2	0,972195217	1616500000
AP-4 complex subunit epsilon-1	AP4E1	0,972006221	29627000
TBC1 domain family member 1	TBC1D1	0,97191175	452980000
Inositol monophosphatase 1	IMPA1	0,97191175	5167900000
Zinc transporter 7	SLC30A7	0,971628449	667800000
Band 4.1-like protein 2	EPB41L2	0,971628449	939570000
KH domain-containing, RNA-binding, signal transduction-associated protein 1	KHDRBS1	0,971534052	1716700000
Lamina-associated polypeptide 2, isoforms beta/gamma;Thymopoietin;Thymopentin;Lamina-associated polypeptide 2, isofor	TMPO	0,971439674	8339700000
Cofilin-1	CFL1	0,971439674	3,1089E+11
Poly [ADP-ribose] polymerase 1	PARP1	0,971250971	5624900000
SH3 domain-binding glutamic acid-rich-like protein	SH3BGRL	0,971156648	3882700000
Cytosolic non-specific dipeptidase	CNDP2	0,971156648	2867800000
Cell cycle and apoptosis regulator protein 2	CCAR2	0,971156648	3008100000
Hermansky-Pudlak syndrome 6 protein	HPS6	0,971062342	16711000
CCR4-NOT transcription complex subunit 10	CNOT10	0,970968055	612970000
GPI transamidase component PIG-T	PIGT	0,970873786	227230000
Serine/arginine-rich splicing factor 6	SRSF6	0,970685304	6294600000
Serine/arginine-rich splicing factor 10	SRSF10	0,970685304	966300000
Tumor susceptibility gene 101 protein	TSG101	0,97059109	1364100000
U2 snRNP-associated SURP motif-containing protein	U2SURP	0,97059109	4088300000
Sorting nexin-15	SNX15;hCG_2044837	0,970496894	177590000
THO complex subunit 4	ALYREF	0,970402717	8557900000
Growth hormone-inducible transmembrane protein	GHITM	0,970308558	1886100000
BAG family molecular chaperone regulator 2	BAG2	0,970308558	1624500000
Serine/threonine-protein kinase SMG1	SMG1	0,970308558	1805800000
Protein diaphanous homolog 1	DIAPH1	0,970214417	34398000000
Transducin beta-like protein 2	TBL2	0,970214417	852500000
Atlastin-3	ATL3	0,970120295	1809600000
Protein transport protein Sec23B	SEC23B	0,970026191	5291700000
Queuine tRNA-ribosyltransferase	QTRT1	0,969932105	88242000

Tropomyosin alpha-3 chain	TPM3;DKFZp686J1372	0,969932105	96451000000
Cyclin-L1	CCNL1	0,969838037	45779000
PRKCA-binding protein	PICK1	0,969743988	87643000
Nuclear cap-binding protein subunit 1	NCBP1	0,969649956	4927400000
Proteasome subunit alpha type;Proteasome subunit alpha type-2	PSMA2	0,969555943	26212000000
Dedicator of cytokinesis protein 8	DOCK8	0,969461949	8933200000
Ubiquitin carboxyl-terminal hydrolase 7;Ubiquitin carboxyl-terminal hydrolase	USP7	0,969367972	13669000000
Cytochrome b-c1 complex subunit 1, mitochondrial	UQCRC1	0,969180074	12352000000
tRNA-dihydrouridine(47) synthase [NAD(P)(+)]-like;tRNA-dihydrouridine(47) synthase [NAD(P)(+)]	DUS3L	0,968992248	426030000
Vacuolar protein-sorting-associated protein 25	VPS25	0,968992248	8343300000
Probable ATP-dependent RNA helicase DDX41	DDX41	0,968898363	100380000
GDP-mannose 4,6 dehydratase	GMDS	0,968804495	2367400000
Uridine-cytidine kinase 1	UCK1	0,968804495	55067000
Ribosomal RNA processing protein 1 homolog A	RRP1	0,968616815	87701000
Integrin beta-1	ITGB1	0,968616815	2374200000
Proteasome assembly chaperone 2	PSMG2	0,968523002	1061500000
Proteasome activator complex subunit 4	PSME4	0,968335431	1598000000
H/ACA ribonucleoprotein complex subunit 4	DKC1	0,968335431	1207800000
Lysosome-associated membrane glycoprotein 1	LAMP1	0,968335431	2835000000
Cell division cycle protein 23 homolog	CDC23	0,968335431	1542900000
Fatty acid synthase;[Acyl-carrier-protein] S-acetyltransferase;[Acyl-carrier-protein] S-malonyltransferase;3-oxoacyl-[acyl-ca	FASN	0,968241673	18387000000
GrpE protein homolog;GrpE protein homolog 1, mitochondrial	GRPEL1	0,967866822	6113500000
Vacuolar protein sorting-associated protein 29	VPS29;DKFZp667O202	0,967679505	12279000000
Serine hydroxymethyltransferase;Serine hydroxymethyltransferase, cytosolic	SHMT1	0,967585873	3274900000
Vacuolar protein sorting-associated protein 51 homolog	VPS51	0,967585873	277390000
Protein asunder homolog	ASUN	0,967585873	274150000
Profilin-1	PFN1	0,96749226	19762000000
Glycolipid transfer protein	GLTP	0,96749226	1487100000
Serine palmitoyltransferase 2	SPTLC2	0,967398665	453610000
U4/U6.U5 small nuclear ribonucleoprotein 27 kDa protein	hCG_1994066;SNRNP27	0,967398665	109290000
Putative small integral membrane protein 11-like	SMIM11P1	0,967305088	53885000
WD repeat-containing protein 74	WDR74	0,967305088	167510000
Catalase	CAT	0,967211529	1247100000
Platelet-activating factor acetylhydrolase IB subunit beta	PAFAH1B2	0,967211529	8856800000
Target of rapamycin complex 2 subunit MAPKAP1	MAPKAP1	0,967211529	17709000
Cyclin-dependent kinase 11A;Cyclin-dependent kinase 11B	CDK11B;CDK11A;CDC2L1;CDC2L	0,967211529	610380000
Activating signal cointegrator 1 complex subunit 1	ASCC1	0,967211529	239400000
Annexin A4;Annexin	ANXA4	0,967024466	12024000000
Septin-8	08-Sep	0,966930961	877020000
Vacuolar protein sorting-associated protein 35	VPS35;DKFZp686O2462	0,966650556	16269000000
RNA 3-terminal phosphate cyclase	RTCA	0,966650556	481830000
Very long-chain specific acyl-CoA dehydrogenase, mitochondrial	ACADVL	0,966557124	608850000
Pyridoxal phosphate phosphatase	PDXP	0,966557124	536070000
Transformer-2 protein homolog beta	DKFZp686F18120;TRA2B	0,966557124	2910700000
General transcription factor II-I	GTF2I	0,966370313	5985600000
Developmentally-regulated GTP-binding protein 2	DRG2	0,966370313	5515600000
E3 ubiquitin-protein ligase HECTD3	HECTD3	0,966090233	74488000
Interleukin enhancer-binding factor 3	ILF3	0,966090233	1355200000
Rho GTPase-activating protein 1	ARHGAP1	0,965996909	2229600000
Splicing factor U2AF 35 kDa subunit	U2AF1	0,965903603	8438300000
Ras-related protein Ral-A	RALA;RALB	0,965903603	2989300000
Argininosuccinate synthase	ASS;ASS1	0,965717045	766590000
Vacuolar protein sorting-associated protein 26A	VPS26A	0,965623793	6830900000
RuvB-like 1	RUVBL1	0,965530559	22892000000
Coatomer subunit delta	ARCN1;DKFZp686M09245	0,965250965	7647600000
PITH domain-containing protein 1	PITHD1	0,965157803	1961800000
Echinoderm microtubule-associated protein-like 3	EML3	0,965157803	117440000
tRNA-splicing endonuclease subunit Sen34	TSEN34	0,965157803	84055000
Ras-related protein Rab-3D	RAB3D	0,965064659	359000000
Arfaptin-2	ARFIP2	0,965064659	197960000
CDP-diacylglycerol--inositol 3-phosphatidyltransferase	CDIPT	0,964878425	2842100000
FGFR1 oncogene partner 2	FGFR1OP2	0,964692263	112440000
cAMP-dependent protein kinase catalytic subunit gamma	PRKACG	0,964692263	893380000
Pantothenate kinase 3;Pantothenate kinase 1	PANK3;PANK1	0,964599209	91175000
Sodium/potassium-transporting ATPase subunit alpha-1	ATP1A1	0,964413155	31610000000
Ankyrin repeat and FYVE domain-containing protein 1	ANKFY1;DKFZp686M19106	0,964320154	3129100000
Tubulin alpha-8 chain	TUBA8;DKFZp686L04275	0,964320154	3817100000
N(4)-(beta-N-acetylglucosaminyl)-L-asparaginase;Glycosylasparaginase alpha chain;Glycosylasparaginase beta chain	AGA	0,964134207	627640000
Coatomer subunit zeta-1	COPZ1	0,964134207	5352900000
Tubulin beta chain	TUBB;XTP3TPATP1	0,963948332	46888000000
Phosphatidylcholine transfer protein	PCTP	0,963855422	52728000
Far upstream element-binding protein 1	FUBP1	0,963855422	14966000000
Stathmin-2;Stathmin	STMN2	0,963669654	3110200000
Coatomer subunit beta	COPB2	0,963391137	14487000000
UDP-glucose:glycoprotein glucosyltransferase 1	UGGT1	0,963298333	18330000000
14 kDa phosphohistidine phosphatase	PHP14;PHPT1	0,963112781	413060000
Peptidyl-prolyl cis-trans isomerase;Peptidyl-prolyl cis-trans isomerase FKBP3	FKBP3	0,963020031	7215600000
E3 ubiquitin-protein ligase RNF123	RNF123	0,963020031	461990000
UPF0598 protein C8orf82	C8orf82	0,962927299	40713000
Protein flightless-1 homolog	FLII	0,962927299	7729300000
LanC-like protein 1	LANCL1	0,962741889	1135400000
BolA-like protein 2	BOLA2B;BOLA2	0,962649211	671040000
WD repeat-containing protein mio	MIOS	0,962649211	134890000
Interferon regulatory factor 2-binding protein-like	IRF2BPL	0,96255655	302300000
Wiskott-Aldrich syndrome protein	WAS	0,962371283	1985600000
Cdc42-interacting protein 4	STP;TRIP10	0,962371283	153940000
N-acetyltransferase 10	NAT10	0,962278676	1799100000
Suppressor of G2 allele of SKP1 homolog	SUGT1	0,962093515	2278800000
Transcription elongation factor B polypeptide 2	TCEB2	0,962000962	5543200000
Transforming protein RhoA	RHOA;RHOC;ARHA	0,961908426	40522000000
RNA-binding protein 7	RBM7	0,961908426	533370000
Complement component 1 Q subcomponent-binding protein, mitochondrial	C1QBP	0,961815908	1430300000
Deoxynucleoside triphosphate triphosphohydrolase SAMHD1	SAMHD1	0,961815908	2328400000
Pseudouridylate synthase 7 homolog	PUS7	0,961815908	1797600000
Ubiquitin carboxyl-terminal hydrolase 5	USP5	0,961630926	5276700000
AP-1 complex subunit mu-1	AP1M1	0,961630926	9278200000
Transmembrane protein 165	TMEM165	0,961538462	810860000
Serine/threonine-protein phosphatase 2B catalytic subunit alpha isoform;Serine/threonine-protein phosphatase	PPP3CA	0,961538462	3070300000
COMM domain-containing protein 3	COMMD3;COMMD3-BMI1	0,961538462	985780000
Signal recognition particle 14 kDa protein	SRP14	0,961538462	2380400000
Mediator of RNA polymerase II transcription subunit 1	MED1	0,961446015	39946000
Polyglutamine-binding protein 1	PQBP1	0,961353586	1035900000
Protein archease	ZBTB8OS	0,961353586	679150000
COMM domain-containing protein 4	COMMD4	0,961168781	1077600000
CDGSH iron-sulfur domain-containing protein 2	CISD2	0,960799385	3190300000
Mediator of RNA polymerase II transcription subunit 4	MED4	0,960799385	413470000
DAZ-associated protein 1	DAZAP1;DAZAP1/MEF2D fusion	0,960799385	4574800000
NEDD8	NEDD8;NEDD8-MDP1	0,96070708	9929800000

E3 ubiquitin-protein ligase UBR1	UBR1	0,96070708	1234700000
Ras-related protein Rab-5B	RAB5B	0,960614793	15131000000
Protein misato homolog 1	MSTO1	0,960614793	101140000
Serine/threonine-protein kinase 38-like	STK38L	0,960614793	228510000
Vesicle-associated membrane protein-associated protein B/C	VAPB	0,960522524	1686400000
Endoplasmic reticulum-Golgi intermediate compartment protein 2	ERGIC2;RLN3	0,960522524	353770000
Serine/threonine-protein phosphatase 6 regulatory ankyrin repeat subunit A	ANKRD28	0,960430273	915060000
Tumor protein D54	TPD52L2;DKFZp686A1765	0,960338039	4007800000
Small integral membrane protein 15	SMIM15	0,959969281	221340000
Phosphoinositide phospholipase C;1-phosphatidylinositol 4,5-bisphosphate phosphodiesterase gamma-1	DKFZp434N101;PLCG1 variant pro	0,959969281	57012000
Ras-related protein Rab-6A	RAB6A	0,959877136	28338000000
Putative ATP-dependent RNA helicase DHX57	DHX57	0,959785008	225850000
Protein SMG8	SMG8	0,959785008	284490000
Receptor expression-enhancing protein 4	REEP4	0,959785008	9345800
Protein transport protein Sec61 subunit gamma	SEC61G	0,959600806	935030000
Ras-related C3 botulinum toxin substrate 1;Ras-related C3 botulinum toxin substrate 3	RAC1;RAC3	0,959232614	17014000000
Extended synaptotagmin-1	ESYT1	0,959232614	22670000000
Small nuclear ribonucleoprotein E	SNRPE	0,959232614	4893300000
Protein phosphatase 1 regulatory subunit 11	PPP1R11	0,959232614	148410000
Cytoplasmic dynein 1 intermediate chain 2	DYNC1I2	0,95914061	1870600000
Cell growth-regulating nucleolar protein	LYAR	0,95914061	182490000
HEAT repeat-containing protein 5B	HEATR5B	0,95914061	1256800000
Terminal uridylyltransferase 4	ZCCHC11	0,959048624	106520000
Signal transducer and activator of transcription;Signal transducer and activator of transcription 5A	STAT5A	0,959048624	481370000
		0,959048624	402880000
Coatomer subunit beta	COPB1	0,958956655	23061000000
Golgin subfamily A member 2	GOLGA2	0,958680855	332330000
Signal recognition particle subunit SRP72	SRP72	0,958497077	6518400000
		0,958313368	186810000
Copper chaperone for superoxide dismutase;Superoxide dismutase [Cu-Zn]	CCS	0,958221541	355730000
Nuclear migration protein nudC	NUDC	0,958037938	12501000000
Proline-, glutamic acid- and leucine-rich protein 1	PELP1	0,958037938	572750000
Transmembrane protein 258	TMEM258	0,957854406	111760000
N-terminal kinase-like protein	SCYL1	0,957854406	383110000
Sphingosine kinase 2	SPHK2	0,957854406	135040000
Dihydroorotate dehydrogenase (quinone), mitochondrial	DHODH	0,957762666	1035100000
Isopentenyl-diphosphate Delta-isomerase 1	IDI1	0,957762666	1038500000
Bcl-2 homologous antagonist/killer	BAK;DKFZp686D0345;BAK1	0,957670944	2051300000
Glycogen synthase kinase-3 alpha	DKFZp686D0638;GSK3A	0,957487553	206600000
Methionine--tRNA ligase, cytoplasmic	MARS	0,957487553	3653000000
NSFL1 cofactor p47	NSFL1C	0,957395883	10464000000
Septin-11	11-Sep	0,957395883	14816000000
Probable global transcription activator SNF2L2	SMARCA2	0,957395883	174780000
Integrin beta;Integrin beta-2	ITGB2	0,957212597	778510000
Peptidyl-prolyl cis-trans isomerase B	PPIB	0,95712098	48273000000
Transcription elongation factor A protein 1	TCEA1	0,95712098	18083000000
Calcium homeostasis endoplasmic reticulum protein	CHERP	0,957029381	2787700000
Nucleolar protein 56	NOP56	0,956754688	2088400000
SPRY domain-containing protein 4	SPRYD4	0,956754688	326540000
Isochorismatase domain-containing protein 1	ISOC1	0,956754688	3770600000
Trafficking protein particle complex subunit 3	TRAPPC3	0,956663159	1186800000
Leukotriene A(4) hydrolase;Leukotriene A-4 hydrolase	LTA4H	0,956663159	11032000000
DNA polymerase;DNA polymerase epsilon catalytic subunit A	POLE;POLE1	0,956663159	96934000
Serine/threonine-protein phosphatase 2A 55 kDa regulatory subunit B delta isoform;Serine/threonine-protein phosphatase 2A	PPP2R2D	0,956663159	63954000
Lysine--tRNA ligase	KARS	0,956571647	6846400000
Ancient ubiquitous protein 1	DKFZp686P12272;AUP1	0,956571647	269310000
Josephin-2	JOSD2	0,956388676	19179000
Serine/threonine-protein kinase 24;Serine/threonine-protein kinase 24 36 kDa subunit;Serine/threonine-protein kinase 24 12 I	STK24;HEL-S-95	0,956388676	1003600000
Translocon-associated protein subunit alpha	SSR1	0,956297217	11703000000
Dolichyl-diphosphooligosaccharide--protein glycosyltransferase 48 kDa subunit	DDOST	0,956297217	24564000000
	LRRFIP1	0,956297217	942550000
Vacuolar protein sorting-associated protein 13B	VPS13B	0,956022945	188360000
Adenylosuccinate lyase	ADSL	0,955931555	3034300000
Trafficking protein particle complex subunit 1	TRAPPC1	0,955748829	335800000
WD repeat-containing protein 7	WDR7	0,955657492	2147500000
		0,955657492	135060000
E3 ubiquitin-protein ligase NEDD4	NEDD4	0,955474871	8684000000
Golgin subfamily A member 3	GOLGA3	0,955474871	527700000
Protein O-glucosyltransferase 1	POGLUT1	0,955383587	1541500000
Uncharacterized protein C1orf106	C1orf106	0,955292319	973170000
ADP-ribosylation factor-like protein 3	ARL3	0,955292319	3616800000
Diphosphoinositol polyphosphate phosphohydrolase 2	NUDT4	0,95520107	121940000
GTP-binding protein Rheb	RHEB	0,95520107	873070000
Cyclin-dependent-like kinase 5	CDK5	0,955109838	2913300000
Eukaryotic translation initiation factor 4E	EIF4E	0,955109838	12739000000
Centromere/kinetochore protein zw10 homolog	ZW10	0,955018623	391350000
Ras-related protein Rab-2B	DKFZp313C1541;RAB2B	0,954927426	79282000
Integrator complex subunit 2	INTS2	0,954927426	388170000
Exocyst complex component 3	EXOC3;DKFZp762K123	0,954836246	351640000
WD repeat and FYVE domain-containing protein 1	WDFY1	0,954836246	174040000
AP-1 complex subunit gamma-1	AP1G1	0,954836246	3927000000
Arf-GAP with coiled-coil, ANK repeat and PH domain-containing protein 2	ACAP2	0,954745083	973610000
Nodal modulator 2;Nodal modulator 3;Nodal modulator 1	NOMO3;NOMO2;NOMO1	0,954653938	6848000000
Charged multivesicular body protein 2a	CHMP2A	0,954653938	1211200000
CAD protein;Glutamine-dependent carbamoyl-phosphate synthase;Aspartate carbamoyltransferase;Dihydroorotase	CAD	0,954653938	52130000000
Hematopoietic lineage cell-specific protein	HCLS1	0,95456281	134510000
CDGSH iron-sulfur domain-containing protein 1	CISD1	0,9544717	1083800000
Tyrosine-protein kinase ABL1	BCR/ABL fusion;ABL1	0,954380607	950340000
Protein FAM50A;Protein FAM50B	FAM50A;FAM50B	0,954198473	1829700000
U3 small nucleolar RNA-associated protein 18 homolog	UTP18	0,954107432	75974000
Valine--tRNA ligase	VARS	0,954107432	39341000000
Coiled-coil domain-containing protein 132	CCDC132;FLJ20097;DKFZp313I24	0,954107432	205590000
H/ACA ribonucleoprotein complex subunit 3	NOP10	0,953925403	173960000
Cytochrome c oxidase assembly factor 3 homolog, mitochondrial	COA3	0,953652489	285910000
Signal recognition particle subunit SRP68	SRP68	0,953652489	5754900000
Trafficking protein particle complex subunit 10	TRAPPC10	0,953561552	15328000
Tripeptidyl-peptidase 2	TPP2	0,953470633	24494000000
FACT complex subunit SSRP1	SSRP1	0,953470633	3937000000
Malate dehydrogenase;Malate dehydrogenase, mitochondrial	MDH2	0,953197979	1,2636E+11
Nuclear envelope phosphatase-regulatory subunit 1	FLJ38101;CNEP1R1	0,953197979	226370000
Cysteine--tRNA ligase, cytoplasmic	CARS	0,953197979	4409700000
Actin-related protein 2/3 complex subunit 3	ARPC3	0,952925481	13092000000
ATP-binding cassette sub-family F member 1	ABCF1	0,952925481	4378500000
U6 snRNA-associated Sm-like protein LSm6	LSM6	0,952834683	2793800000
Prothymosin alpha;Prothymosin alpha, N-terminally processed;Thymosin alpha-1	PTMA;PTMAP7	0,952834683	1942800000
Iron-sulfur cluster assembly 2 homolog, mitochondrial	ISCA2	0,952653139	459490000
NAD-dependent malic enzyme, mitochondrial	ME2	0,952653139	2950500000
Rab11 family-interacting protein 1;Rab11 family-interacting protein 2	RAB11FIP1;RAB11FIP2	0,952653139	4397100000

Dynactin subunit 1	DCTN1;DKFZp686I0746;DKFZp686	0,952562393	9292500000
Protein DENND6A	DENND6A	0,952562393	14182000
	ACT	0,952471664	1180100000
DDB1- and CUL4-associated factor 7	DCAF7	0,952380952	172820000
Coatomer subunit gamma-1	COPG1;COPG	0,952380952	15090000000
Talin-2	TLN2	0,952380952	160080000
Thymidylate kinase	DTYMK	0,952108921	1028600000
Cysteine protease ATG4B	ATG4B;DKFZp686G0859	0,951927653	719730000
Copine-3	CPNE3	0,951927653	455740000
ATP synthase subunit e, mitochondrial	ATP5I	0,951837045	2204300000
14-3-3 protein epsilon	YWHAE;YWHAE/FAM22B fusion;YW	0,951837045	2,8722E+11
Integrin alpha-L	ITGAL	0,951746455	171680000
Kinesin-like protein KIF13B	KIF13B	0,951655881	325050000
LDLR chaperone MESD	MESDC2	0,951565325	4737400000
Mediator of RNA polymerase II transcription subunit 18	MED18	0,951474786	382720000
Ras-related protein Rab-27A	RAB27A	0,951384264	295190000
Inositol monophosphatase 2	IMPA2	0,951384264	1120500000
Elongator complex protein 1	DKFZp781H1425;IKBKAP;IKBKAP v	0,951384264	2337700000
Matrin-3	MATR3;DKFZp686K23100	0,95129376	1452800000
Centrobin	CNTROB	0,951203272	197460000
Dynein light chain roadblock-type 1;Dynein light chain roadblock-type 2	DYNLRB1;DYNLRB2	0,951022349	832870000
Phosphatidylglycerophosphatase and protein-tyrosine phosphatase 1	PTPMT1	0,950841495	33214000
Stress-induced-phosphoprotein 1	STIP1	0,950660709	31649000000
UV excision repair protein RAD23 homolog A	RAD23A	0,950660709	367010000
Ubiquitin-like-conjugating enzyme ATG3	ATG3	0,950660709	928420000
WASH complex subunit strumpellin	KIAA0196	0,950570342	1821200000
Vacuolar protein sorting-associated protein 11 homolog	VPS11;DKFZp564P2364	0,950479992	245660000
HIV Tat-specific factor 1	HTATSF1	0,950118765	197800000
Proteasome subunit beta type;Proteasome subunit beta type-7	PSMB7	0,949938254	11036000000
Ankyrin repeat and EF-hand domain-containing protein 1	ANKRD5;ANKEF1	0,949848024	1333600000
Leucyl-cystinyl aminopeptidase;Leucyl-cystinyl aminopeptidase, pregnancy serum form	LNPEP	0,949667616	3088000000
RuvB-like 2	RUVBL2	0,949667616	23117000000
Transmembrane protein 189	TMEM189	0,949667616	66057000
Homeobox protein CDX-1	CDX1	0,949577438	1216000000
Sarcoplasmic/endoplasmic reticulum calcium ATPase 2	ATP2A2	0,949487277	24011000000
Sorting nexin-3	SNX3	0,949397133	4384100000
TBC1 domain family member 14	TBC1D14	0,949216896	117810000
Golgi-specific brefeldin A-resistance guanine nucleotide exchange factor 1	GBF1	0,949216896	6593100000
N-acylneuraminate cytidylyltransferase	CMAS	0,949126803	2934900000
DCC-interacting protein 13-alpha	APPL1	0,949126803	862530000
E3 UFM1-protein ligase 1	UFL1	0,949126803	802130000
Heterogeneous nuclear ribonucleoprotein R	HNRNPR;HNRPR;DKFZp686A132	0,949126803	2286000000
Mitochondrial import inner membrane translocase subunit Tim17-B	TIMM17B	0,949036728	243250000
Vitamin K epoxide reductase complex subunit 1-like protein 1	VKORC1L1	0,948946669	545390000
Cytochrome c oxidase copper chaperone	COX17	0,948856628	56053000
Proteasome subunit alpha type-4;Proteasome subunit alpha type;Proteasome subunit beta type	PSMA4	0,948856628	26554000000
Sorting nexin-6;Sorting nexin-6, N-terminally processed	SNX6	0,948766603	5326800000
Calpain-1 catalytic subunit	CAPN1	0,948586606	2616000000
Drebrin-like protein	DBNL	0,948586606	600850000
Leucine-rich repeat-containing protein 40	LRRC40	0,948586606	584970000
Ras-related protein Rab-21	RAB21	0,948496633	11717000000
Serine/arginine-rich splicing factor 7	SRSF7	0,948496633	3697900000
Transmembrane 9 superfamily member 2	TM9SF2	0,948316738	1389000000
Vacuolar protein sorting-associated protein 53 homolog	HCCS1;VPS53	0,948316738	158960000
Ubiquitin-conjugating enzyme E2 G1;Ubiquitin-conjugating enzyme E2 G1, N-terminally processed	UBE2G1	0,948047023	1710500000
Acetyl-CoA carboxylase 1;Biotin carboxylase	ACACA	0,948047023	9537300000
V-type proton ATPase subunit D	VATD;ATP6V1D	0,947867299	2053000000
Mitochondrial import inner membrane translocase subunit Tim23;Putative mitochondrial import inner membrane translocase s	TIMM23;TIMM23B	0,947687642	1419500000
Sigma non-opioid intracellular receptor 1	hCG_20471;SIGMAR1	0,947597839	2027100000
Histone-lysine N-methyltransferase SETD2	SETD2	0,947149081	15098000
SNARE-associated protein Snapin	SNAPIN	0,947059381	432320000
Dolichyl-diphosphooligosaccharide--protein glycosyltransferase subunit 1	RPN1	0,94688003	24991000000
Major vault protein	MVP	0,946790381	11655000000
General transcription factor IIF subunit 1	GTF2F1	0,946790381	480810000
NudC domain-containing protein 2	NUDCD2	0,946790381	4093600000
Urotensin-2	UTS2	0,946700748	9069200000
Adenosine deaminase	ADA	0,946521533	242960000
Protein LZIC	LZIC	0,946431952	906450000
Phosphatidate phosphatase LPIN1	LPIN1;DKFZp781P1796	0,946342387	63900000
RNA-binding protein 25	RBM25	0,946252839	6715600000
Sec1 family domain-containing protein 2	SCFD2	0,946163308	58919000
Mitogen-activated protein kinase kinase kinase MLT	pk;ZAK	0,946073794	437760000
Pre-mRNA-processing factor 39	PRPF39	0,945984297	213020000
Protein disulfide-isomerase A6	PDIA6	0,945805353	48318000000
Rab3 GTPase-activating protein non-catalytic subunit	RAB3GAP2	0,945805353	2361400000
Adenosylhomocysteinase	AHCY	0,945715907	74236000000
TBC1 domain family member 8B	TBC1D8B	0,945715907	406110000
Glucosidase 2 subunit beta	PRKCSH	0,945179584	1832100000
Transcription elongation factor B polypeptide 1	TCEB1	0,945179584	6348600000
Rab proteins geranylgeranyltransferase component A 1	CHM	0,945179584	42622000
OCIA domain-containing protein 1	OCIAD1	0,945090256	1636100000
Ataxin-10	ATXN10	0,945000945	166040000
Kelch domain-containing protein 4	KLHDC4	0,945000945	216790000
Exocyst complex component 1	EXOC1	0,945000945	162160000
	ACT	0,944911651	3760400000
Bcl-2-like protein 13	BCL2L13;DKFZp451F173	0,944733113	135930000
Rab GTPase-activating protein 1	RABGAP1	0,944643869	1026200000
Beta-adrenergic receptor kinase 1	ADRBK1	0,944465433	575700000
Lupus La protein	SSB	0,944465433	5760100000
Biogenesis of lysosome-related organelles complex 1 subunit 1	BLOC1S1	0,944287063	155380000
Ufm1-specific protease 2	UFSP2	0,944197904	120570000
ADP-ribosylation factor 1;ADP-ribosylation factor 3	ARF1;ARF3	0,944197904	71464000000
THO complex subunit 1	THOC1	0,944197904	135920000
Ribosomal protein S6 kinase alpha-5;Ribosomal protein S6 kinase	RPS6KA5	0,944108761	441200000
Glycerophosphodiester phosphodiesterase 1	GDE1	0,944019636	167270000
Peptidyl-prolyl cis-trans isomerase-like 1	PPIL1	0,943930527	2256700000
Condensin complex subunit 2	NCAPH	0,943752359	513260000
Dolichyl-diphosphooligosaccharide--protein glycosyltransferase subunit STT3A	STT3A	0,943663301	10012000000
Chitinase domain-containing protein 1	CHID1	0,943663301	736600000
Sorting nexin-2	SNX2	0,943485234	6642600000
NADH dehydrogenase [ubiquinone] 1 alpha subcomplex subunit 13	NDUFA13	0,943396226	1061300000
Target of Myb protein 1	TOM1;UNQ1844	0,943396226	30500000
Dehydrogenase/reductase SDR family member 4	DHRS4;DHRS4L2	0,943307235	2179700000
1-phosphatidylinositol 4,5-bisphosphate phosphodiesterase gamma-2;Phosphoinositide phospholipase C	PLCG2	0,943218261	7289900000
Ribose-phosphate pyrophosphokinase 2	PRPS2	0,943218261	4273200000
Cleavage and polyadenylation specificity factor subunit 1	CPSF1	0,943129303	1500600000
Ras-related protein Rap-2b	DKFZp547A0616;RAP2B	0,943129303	5262800000
Endoplasmic reticulum resident protein 44	ERP44	0,943040362	14087000000

Serrate RNA effector molecule homolog	SRRT	0,943040362	10464000000
Solute carrier family 25 member 40	SLC25A40	0,942951438	117880000
Phosphatidylinositol glycan anchor biosynthesis class U protein	PIGU	0,942862531	384790000
Protein S100;Protein S100-A6	S100A6	0,94277364	3548700000
Akirin-2	AKIRIN2	0,942151875	667960000
Flotillin-1	FLOT1	0,941885655	51517000
Presqualene diphosphate phosphatase	PPAPDC2	0,941885655	162700000
Bifunctional 3-phosphoadenosine 5-phosphosulfate synthase 1;Sulfate adenylyltransferase;Adenylyl-sulfate kinase	PAPSS1	0,941796949	235940000
Kinesin light chain 4	KLC4	0,94144229	73470000
HRAS-like suppressor 3	PLA2G16	0,941353667	112050000
Proteasome-associated protein ECM29 homolog	ECM29;KIAA0368	0,941353667	19462000000
Ras GTPase-activating-like protein IQGAP1	IQGAP1	0,94126506	50432000000
Dolichyl-diphosphooligosaccharide--protein glycosyltransferase subunit 2	RPN2	0,941176471	12701000000
Pre-mRNA-splicing factor 18	PRPF18;LOC101928524	0,941176471	128830000
Testin	TES	0,941176471	2306000000
Vacuolar protein sorting-associated protein 26B	VPS26B	0,941087898	3213100000
Sister chromatid cohesion protein PDS5 homolog A	PDS5A	0,940999341	19237000000
Transmembrane emp24 domain-containing protein 5	TMED5	0,940822279	3918000000
Cell cycle control protein 50A	TMEM30A	0,940733772	123340000
Target of EGR1 protein 1	TOE1	0,940733772	88737000
Acylphosphatase;Acylphosphatase-1	FBN3;ACYP1	0,940733772	558060000
Dehydrogenase/reductase SDR family member 11	DHRS11	0,940645283	648970000
Mitochondrial dicarboxylate carrier	SLC25A10	0,94055681	197500000
Coatomer subunit alpha;Xenin;Proxenin	COPA	0,940468353	52739000000
mRNA-capping enzyme;Polynucleotide 5-triphosphatase;mRNA guanylyltransferase	RNGTT	0,94029149	130880000
Peptidyl-prolyl cis-trans isomerase;Peptidyl-prolyl cis-trans isomerase G	PPIG	0,94029149	232180000
Tyrosine--tRNA ligase, cytoplasmic;Tyrosine--tRNA ligase, cytoplasmic, N-terminally processed	YARS	0,940203084	14339000000
		0,940114694	16295000
Non-specific lipid-transfer protein	SCP2	0,940026321	1781700000
NHL repeat-containing protein 3	NHLRC3	0,939761301	44654000
Ribose-phosphate pyrophosphokinase 1	PRPS1	0,939584704	36126000000
Protein transport protein Sec61 subunit alpha isoform 1	SEC61A1	0,939584704	22051000000
Phosphoinositide 3-kinase regulatory subunit 4	PIK3R4	0,939408173	1992000000
TAF6-like RNA polymerase II p300/CBP-associated factor-associated factor 65 kDa subunit 6L	TAF6L	0,939319932	98371000
Rab GTPase-binding effector protein 1	RABEP1	0,939231708	125650000
Histone deacetylase 3	HDAC3	0,93905531	370530000
Striatin-interacting protein 1	STRIP1;FAM40A	0,93905531	146680000
Bifunctional glutamate/proline--tRNA ligase;Glutamate--tRNA ligase;Proline--tRNA ligase	EPRS	0,938967136	52610000000
Conserved oligomeric Golgi complex subunit 7	COG7	0,938878978	271470000
Protein lunapark	LNP;KIAA1715	0,938614605	386460000
V-type proton ATPase 116 kDa subunit a isoform 1	DKFZp686N0561;ATP6V0A1	0,938526513	582020000
Fatty acid-binding protein, epidermal	FABP5	0,938262338	3180100000
		0,938086304	141700000
Galactokinase	GALK;GALK1	0,938086304	5881600000
Rho-associated protein kinase;Rho-associated protein kinase 1	ROCK1	0,938086304	4182100000
Unconventional myosin-Va	MYO5A	0,937822376	868470000
Inositol 1,4,5-trisphosphate receptor type 1	ITPR1	0,937734434	4469200000
GPI transamidase component PIG-S	PIGS	0,937558597	1555600000
Sec1 family domain-containing protein 1	SCFD1	0,937470704	1635300000
GPI-anchor transamidase	PIGK	0,937294967	2770400000
RNA polymerase II-associated protein 3	RPAP3	0,937294967	151930000
Heme oxygenase 2	HMOX2	0,937207123	1787100000
ER degradation-enhancing alpha-mannosidase-like protein 2	EDEM2	0,937119295	18965000
Cullin-2	CUL2	0,937119295	3413500000
Frataxin, mitochondrial;Frataxin intermediate form;Frataxin(56-210);Frataxin(78-210);Frataxin mature form	FXN	0,937031484	828680000
Nucleoside diphosphate kinase;Nucleoside diphosphate kinase B;Putative nucleoside diphosphate kinase	NME1-NME2;NME2;NME1;NME2P1	0,937031484	1,1435E+11
Moesin	MSN	0,93694369	73602000000
tRNA-splicing ligase RtcB homolog	RTCB	0,93694369	4229100000
Mitochondrial import inner membrane translocase subunit Tim9	TIMM9	0,93676815	2342300000
C-myc promoter-binding protein	DENND4A	0,936592676	330950000
Glycerol-3-phosphate dehydrogenase 1-like protein	GPD1L	0,936504963	4958000000
Nuclear pore membrane glycoprotein 210	NUP210	0,936417268	2121500000
Vacuolar protein sorting-associated protein 18 homolog	VPS18	0,936329588	322150000
N-alpha-acetyltransferase 30	NAA30	0,936329588	177640000
Acyl-coenzyme A thioesterase 1;Acyl-coenzyme A thioesterase 2, mitochondrial	ACOT1;ACOT2	0,936241925	1704700000
Small subunit processome component 20 homolog	UTP20	0,936241925	362050000
Programmed cell death 6-interacting protein	DRIP4;PDCD6IP	0,936154278	20511000000
Acyl-CoA desaturase	SCD	0,935979034	60696000
Coiled-coil domain-containing protein 127	CCDC127	0,935716291	386290000
RNA-binding protein 26	RBM26	0,935716291	2148600000
Small nuclear ribonucleoprotein Sm D3	SNRPD3	0,935628743	13264000000
VIP36-like protein	LMAN2L	0,935541211	341360000
Probable E3 ubiquitin-protein ligase HECTD4	HECTD4	0,935366196	518540000
Proteasome activator complex subunit 1	PSME1	0,935366196	33647000000
	DKFZp686I14200	0,935191247	478910000
Mitochondrial import inner membrane translocase subunit Tim10	TIMM10	0,935016363	614750000
Volume-regulated anion channel subunit LRRC8C	LRRC8C	0,934928945	65927000
Unconventional myosin-Ic	MYO1C	0,934928945	383960000
Cytochrome c1, heme protein, mitochondrial	CYC1	0,93475416	8365100000
GTP cyclohydrolase 1	GCH1	0,934666791	1458000000
Actin-related protein 2	ACTR2	0,934579439	37165000000
R3H domain-containing protein 1	R3HDM;R3HDM1	0,934579439	77833000
Proteasome subunit alpha type-3;Proteasome subunit alpha type	PSMA3	0,934579439	37814000000
Signal recognition particle 19 kDa protein	SRP19	0,934579439	1346700000
Polyadenylate-binding protein 2	PABPN1	0,934404784	6636100000
Endoplasmic reticulum metallopeptidase 1	KIAA1815;ERMP1	0,934317481	1576800000
Calpain small subunit 1	CAPNS1	0,934317481	6756000000
SWI/SNF-related matrix-associated actin-dependent regulator of chromatin subfamily A containing DEAD/H box 1	SMARCAD1	0,934317481	41488000
Unconventional myosin-XVIIIa	MYO18A	0,934230194	1752900000
Kinesin light chain 2	KLC2	0,934142924	706340000
Leucine-rich repeat protein SHOC-2	SHOC2	0,934142924	313140000
Low molecular weight phosphotyrosine protein phosphatase	ACP1	0,934142924	8752000000
Actin-related protein 3	ACTR3	0,93405567	40537000000
Serine/threonine-protein kinase PRP4 homolog	PRPF4B	0,93405567	386070000
N-acetylglucosamine-6-sulfatase	GNS;DKFZp686E12166	0,933968432	146890000
Proteasome subunit alpha type-6;Proteasome subunit alpha type	PSMA6	0,933968432	42919000000
Protein RCC2	RCC2	0,93388121	20663000000
Oligoribonuclease, mitochondrial	REXO2	0,933794005	3633600000
Delta-1-pyrroline-5-carboxylate dehydrogenase, mitochondrial	ALDH4A1	0,933619643	148280000
Core-binding factor subunit beta	CBFB	0,933619643	3665700000
Mediator of RNA polymerase II transcription subunit 24	MED24	0,933532487	237160000
Proteasome subunit beta type-4	PSMB4	0,933358223	18010000000
Tyrosine-protein kinase BTK	BTK;BTK kinase deficient isoform 2	0,933271115	4122200000
ATP-binding cassette sub-family D member 3	ABCD3	0,933271115	573330000
Armadillo repeat-containing protein 6	ARMC6	0,933184024	183400000
Sorting nexin-12	SNX12	0,933096949	1835000000
Proteasome subunit beta type-2;Proteasome subunit beta type	PSMB2	0,933096949	26787000000
WD repeat-containing protein 37	WDR37	0,933096949	243040000

Ras-related protein Rab-14	RAB14	0,932661817	24679000000
Arf-GAP with Rho-GAP domain, ANK repeat and PH domain-containing protein 3	ARAP3	0,932661817	827930000
Ribosomal protein S6 kinase alpha-3	RPS6KA3	0,932574839	6214100000
Disco-interacting protein 2 homolog B	DIP2B	0,932314003	923100000
RNA polymerase II-associated protein 1	RPAP1	0,932227091	91636000
Mitochondrial import inner membrane translocase subunit Tim13	TIMM13	0,932140194	2052700000
Vesicle-associated membrane protein 7	VAMP7	0,932140194	2149800000
Apoptotic chromatin condensation inducer in the nucleus	ACIN1;DKFZp667N107	0,932140194	923970000
Serine/threonine-protein kinase OSR1	OXSR1	0,931879601	2249800000
Vesicle-trafficking protein SEC22b	SEC22B	0,931879601	11947000000
Histone deacetylase 8	HDAC8	0,931792769	130810000
Syntaxin-binding protein 5	STXBP5;Nbla04300	0,931705954	544370000
Heterogeneous nuclear ribonucleoprotein A3	HNRNPA3	0,931619154	26513000000
Dedicator of cytokinesis protein 2	DOCK2	0,931532371	19484000000
Liprin-alpha-1	PPFIA1	0,931358853	1188000000
Pyridoxal kinase	PDXK	0,931272118	306410000
Magnesium transporter protein 1	MAGT1	0,931185399	554340000
Eukaryotic translation initiation factor 4E-binding protein 2	EIF4EBP2	0,931185399	74192000
	ZSCAN29	0,931185399	379340000
Mesencephalic astrocyte-derived neurotrophic factor	MANF	0,931098696	17419000000
Thyroid adenoma-associated protein	THADA;GITA/3p fusion;GITA/7p fus	0,931098696	650650000
Protein S100-A11;Protein S100-A11, N-terminally processed;Protein S100	S100A11	0,93101201	2324000000
DIS3-like exonuclease 2	DIS3L2	0,930838686	124320000
Translocon-associated protein subunit gamma	SSR3	0,93057882	2979900000
Delta-1-pyrroline-5-carboxylate synthase;Glutamate 5-kinase;Gamma-glutamyl phosphate reductase	ALDH18A1	0,930319099	21447000000
Heparan-alpha-glucosaminide N-acetyltransferase	HGSNAT	0,930319099	89838000
Alpha-N-acetylglucosaminidase;Alpha-N-acetylglucosaminidase 82 kDa form;Alpha-N-acetylglucosaminidase 77 kDa form	NAGLU;ufHSD2	0,930146033	63750000
Actin-related protein 2/3 complex subunit 1B	ARPC1B	0,930146033	26430000000
Ceramide synthase 6	CERS6	0,930059524	588580000
WASH complex subunit CCDC53	CCDC53	0,929886554	311100000
Transducin-like enhancer protein 4;Transducin-like enhancer protein 1	TLE4;TLE1	0,929800093	58025000
ER degradation-enhancing alpha-mannosidase-like protein 3	EDEM3	0,929713648	51184000
PRA1 family protein 3	ARL6IP5	0,929627219	903970000
Mediator of RNA polymerase II transcription subunit 14	MED14	0,929540807	485160000
Acylamino-acid-releasing enzyme	APEH	0,929195317	1111200000
B-cell receptor-associated protein 31	BCAP31	0,929108984	6841100000
Probable ergosterol biosynthetic protein 28	C14orf1	0,929022668	828320000
Vacuolar protein sorting-associated protein 16 homolog	VPS16	0,928936368	435080000
DNA topoisomerase;DNA topoisomerase 3-beta-1	TOP3B	0,928677563	81473000
Tubulin alpha-1A chain	TUBA1A	0,928677563	3294900000
Fumarate hydratase, mitochondrial	FH	0,928591327	8026700000
39S ribosomal protein L48, mitochondrial	MRPL48	0,928418903	628040000
Ras-related protein Rab-7a	RAB7A	0,928418903	46665000000
GDH/6PGL endoplasmic bifunctional protein;Glucose 1-dehydrogenase;6-phosphogluconolactonase	H6PD	0,928074246	190090000
Transformer-2 protein homolog alpha	TRA2A;HSU53209	0,928074246	402320000
YLP motif-containing protein 1	YLPM1;FLJ00353	0,928074246	292600000
Dolichyl-phosphate beta-glucosyltransferase	ALG5	0,927988122	393020000
Signal peptidase complex catalytic subunit SEC11C	SEC11C;SEC11L3	0,927815921	996630000
Golgi resident protein GCP60	ACBD3	0,927471712	114820000
Arginine--tRNA ligase, cytoplasmic	RARS	0,927471712	10334000000
Ectonucleoside triphosphate diphosphohydrolase 5	ENTPD5	0,9273857	286650000
Inactive phospholipase C-like protein 2;Phosphoinositide phospholipase C	PLCL2	0,9273857	390660000
Sterol-4-alpha-carboxylate 3-dehydrogenase, decarboxylating	NSDHL	0,927299703	1270500000
WD repeat-containing protein 11	WDR11;DKFZp434L1715	0,927213723	306500000
Translation initiation factor eIF-2B subunit epsilon	EIF2B5	0,927213723	1190200000
ADP-ribosylation factor 6	ARF6	0,927127758	4518000000
Splicing factor 45	RBM17	0,926955877	1077400000
TBC1 domain family member 13	TBC1D13	0,926698174	96263000
Serine/threonine-protein kinase TAO1	TAOK1	0,926612305	285310000
DnaJ homolog subfamily C member 13	DNAJC13	0,926354794	3976500000
UBX domain-containing protein 6	UBXN6	0,926354794	28540000
Thyroid hormone receptor-associated protein 3	THRAP3	0,926354794	2666400000
Beta-hexosaminidase;Beta-hexosaminidase subunit alpha	HEXA	0,926268989	96754000
V-type proton ATPase subunit F	ATP6V1F	0,926268989	1202900000
Xaa-Pro dipeptidase	PEPD	0,926183199	287860000
Endophilin-A2	SH3GL1	0,926183199	755250000
Brefeldin A-inhibited guanine nucleotide-exchange protein 1	ARFGEF1	0,926183199	3272100000
60 kDa SS-A/Ro ribonucleoprotein	TROVE2	0,926097425	89114000
RNA-binding protein 8A	RBM8A	0,926097425	5137700000
	MYO1C	0,926011668	149370000
Tubulin alpha chain-like 3	TUBAL3	0,926011668	83219000
Cytochrome b-c1 complex subunit Rieske, mitochondrial;Cytochrome b-c1 complex subunit 11;Putative cytochrome b-c1 co	UQCRFS1;UQCRFS1P1	0,925925926	2094900000
Dual specificity mitogen-activated protein kinase kinase 2	MAP2K2;DKFZp686L02273	0,925925926	1096300000
Carnitine O-palmitoyltransferase 2, mitochondrial	CPT2	0,925668796	146050000
Nucleoside diphosphate kinase A	NME1	0,925668796	18966000000
FAS-associated factor 2	FAF2	0,925583117	1012500000
39S ribosomal protein L9, mitochondrial	MRPL9	0,925497455	573110000
Spermatogenesis-defective protein 39 homolog	C14orf133;VIPAS39	0,925497455	113410000
Engulfment and cell motility protein 1	ELMO1	0,925497455	3870800000
tRNA (uracil-5-)-methyltransferase homolog A	TRMT2A	0,925411808	422270000
Neurogenic locus notch homolog protein 2;Notch 2 extracellular truncation;Notch 2 intracellular domain	NOTCH2	0,925326177	52821000
Pantothenate kinase 2, mitochondrial	PANK2;hCG_39342	0,925326177	142060000
	BRL	0,925240563	3346800000
Ubiquitin carboxyl-terminal hydrolase BAP1	BAP1	0,925240563	91933000
60 kDa heat shock protein, mitochondrial	HSPD1	0,925154963	73134000000
Serine/threonine-protein phosphatase;Serine/threonine-protein phosphatase 2A catalytic subunit beta isoform	PPP2CB	0,92506938	571710000
Sideroflexin-3	SFXN3	0,924983813	9373600000
B-cell receptor-associated protein 29	BCAP29	0,924983813	2267200000
Protein transport protein Sec16A	SEC16A	0,924983813	538390000
Cleft lip and palate transmembrane protein 1-like protein	CLPTM1L	0,924812725	307840000
Small nuclear ribonucleoprotein F	SNRPF	0,924727205	4956000000
Ubiquinone biosynthesis monooxygenase COQ6	COQ6	0,924556213	131890000
Endoplasmin	TRA1;HSP90B1	0,924470741	1,1437E+11
Mitochondrial ornithine transporter 1	SLC25A15	0,924043615	122440000
N-lysine methyltransferase SMYD2	SMYD2	0,923958237	52460000
ER lumen protein-retaining receptor 2	KDELR2	0,923958237	3465900000
BET1 homolog	BET1;DKFZp781C0425	0,923872875	504730000
cAMP-dependent protein kinase catalytic subunit beta	PRKACB	0,923872875	713210000
Isoleucine--tRNA ligase, mitochondrial	IARS2	0,923702198	1211300000
WD repeat-containing protein 91	WDR91	0,923531585	54573000
Uncharacterized protein C17orf62	C17orf62	0,923446302	44918000
Transcription factor Dp-1	TFDP1	0,923446302	379860000
Tyrosine-protein kinase receptor;Kinesin-1 heavy chain	KIF5B-RET(NM_020630)_K23;R12;	0,922934933	12469000000
Sequestosome-1	SQSTM1;OSIL	0,922934933	1880300000
		0,922679461	821830000
Hypoxia up-regulated protein 1	HYOU1	0,922594335	33434000000
Trimethylguanosine synthase	TGS1	0,922339052	82172000
Proteasome subunit beta type;Proteasome subunit beta type-1	PSMB1	0,921998894	25975000000

Aldo-keto reductase family 1 member B15	LOC402299;AKR1B15	0,921913893	664530000
Mitochondrial import receptor subunit TOM20 homolog	TOMM20	0,92174394	685130000
Hsc70-interacting protein;Putative protein FAM10A5;Putative protein FAM10A4	ST13;ST13P5;ST13P4;hCG_19906:	0,92174394	17699000000
ATPase family AAA domain-containing protein 1	ATAD1	0,921658986	7324400000
	EEF1A1	0,921149595	141190000
39S ribosomal protein L39, mitochondrial	MRPL39	0,921064751	410520000
Tuberin	TSC2	0,921064751	27283000
Ras-related protein Rab-22A	RAB22A	0,920979923	663710000
Probable 28S rRNA (cytosine(4447)-C(5))-methyltransferase	NOP2	0,920979923	199600000
Regulation of nuclear pre-mRNA domain-containing protein 1A	RPRD1A	0,920979923	442970000
Eukaryotic translation elongation factor 1 epsilon-1	EEF1E1;EEF1E1-BLOC1S5	0,920979923	2974300000
Inositol hexakisphosphate and diphosphoinositol-pentakisphosphate kinase 2	PPIP5K2	0,920810313	525950000
Retinol dehydrogenase 11	RDH11	0,920725532	1024300000
Enhancer of rudimentary homolog	ERH	0,920640766	3475400000
Tetratricopeptide repeat protein 1	TTC1	0,920471281	1105900000
AP-3 complex subunit delta-1	AP3D1	0,920301859	3078500000
		0,920217171	555780000
Tyrosine--tRNA ligase, mitochondrial	YARS2	0,920217171	132010000
NADH dehydrogenase [ubiquinone] 1 beta subcomplex subunit 3	NDUFB3	0,920047842	266080000
Nucleolar and coiled-body phosphoprotein 1	NOLC1	0,920047842	96228000
Annexin A2;Annexin;Putative annexin A2-like protein	ANXA2;ANXA2P2	0,919963201	68820000000
Vacuolar protein sorting-associated protein 41 homolog	HVPS41;VPS41	0,919793966	308630000
AMP deaminase 2	AMPD2	0,919793966	2647400000
Ubiquinol-cytochrome-c reductase complex assembly factor 2	UQCC2	0,91954023	44630000
Vacuolar protein-sorting-associated protein 36	VPS36	0,91954023	3241600000
Chromatin assembly factor 1 subunit B	CHAF1B	0,919202133	632470000
Syntaxin-12	STX12	0,919202133	800090000
Transmembrane emp24 domain-containing protein 4	TMED4	0,918864284	1979200000
Tripartite motif-containing protein 16-like protein;Tripartite motif-containing protein 16	TRIM16;TRIM16L	0,918864284	500570000
Thioredoxin-dependent peroxide reductase, mitochondrial	PRDX3	0,918695452	18040000000
AP-2 complex subunit sigma	AP2S1	0,91861106	1455100000
Phosphatidylinositide phosphatase SAC1	SACM1L	0,918357976	1658400000
H/ACA ribonucleoprotein complex non-core subunit NAF1	NAF1	0,918273646	127280000
		0,918273646	1471000000
Polyadenylate-binding protein-interacting protein 1	PAIP1	0,918189331	222090000
FYN-binding protein	FYB	0,918189331	286730000
Syntaxin-17	STX17;DKFZp762D1813	0,918020747	295250000
ATP synthase subunit beta, mitochondrial;ATP synthase subunit beta	ATP5B	0,917852226	1,6696E+11
V-type proton ATPase subunit E 1	ATP6V1E1	0,917767988	6140600000
YEATS domain-containing protein 4	YEATS4	0,917767988	63198000
Protein NipSnap homolog 3A;Protein NipSnap homolog 3B	NIPSNAP3A;NIPSNAP3B	0,91759956	296760000
Glyceraldehyde-3-phosphate dehydrogenase	GAPDH	0,91759956	3,2685E+11
NADH-ubiquinone oxidoreductase chain 1	NADH1;nd1;ND1;ndh1;ROPN1B;M1	0,917178758	108690000
Polypeptide N-acetylgalactosaminyltransferase 1;Polypeptide N-acetylgalactosaminyltransferase 1 soluble form	GALNT1	0,917178758	198510000
Paraspeckle component 1	PSPC1	0,916842395	2687400000
UPF0568 protein C14orf166	C14orf166	0,916758343	7803600000
Cytochrome b-c1 complex subunit 8	UQCRQ	0,916674306	429230000
Spliceosome RNA helicase DDX39B	DDX39B;hCG_2005638	0,916338312	48970000000
Triple functional domain protein	TRIO	0,916254352	13099000
Plasma membrane calcium-transporting ATPase 1;Calcium-transporting ATPase	ATP2B1	0,916086479	2869900000
Eukaryotic initiation factor 4A-II	EIF4A2	0,916002565	1018300000
Hepatoma-derived growth factor-related protein 2;Hepatoma-derived growth factor-related protein 3	HDGFRP2;HDGFRP3	0,915834783	209030000
CDK5 regulatory subunit-associated protein 3	CDK5RAP3	0,915834783	117070000
Cytosol aminopeptidase	LAP3	0,915667063	4410700000
Pre-mRNA-processing factor 40 homolog A	PRPF40A	0,915583227	2709000000
Protein FAM107B	FAM107B	0,915415599	2481800000
Calcium-transporting ATPase;Sarcoplasmic/endoplasmic reticulum calcium ATPase 3	ATP2A3	0,915415599	3142200000
WD repeat and FYVE domain-containing protein 3	WDFY3	0,915248032	60353000
Vacuolar protein sorting-associated protein 13C	VPS13C	0,915080527	4611800000
RAD50-interacting protein 1	RINT1	0,914913083	62451000
7-methylguanosine phosphate-specific 5-nucleotidase	NT5C3B	0,914829384	1437900000
Cytoplasmic dynein 2 heavy chain 1	DYNC2H1	0,914829384	451980000
GRB2-related adapter protein	GRAP	0,914829384	20024000
V-type proton ATPase catalytic subunit A	ATP6V1A	0,914745701	6768600000
Growth factor receptor-bound protein 2	GRB2	0,914745701	10249000000
Protein DJ-1	PARK7	0,914662032	25190000000
ATP-dependent RNA helicase DDX50	mcdrh;DDX50;DKFZp761E0323	0,914662032	64210000
Legumain	LGMN	0,914494742	451190000
Proteasome subunit beta type-3	PSMB3	0,914494742	19363000000
Uracil phosphoribosyltransferase homolog	UPRT;RP11-311P8.3	0,914411119	341670000
3-5 exoribonuclease 1	ERI1	0,914327512	262040000
Small nuclear ribonucleoprotein Sm D2	SNRPD2	0,914327512	12985000000
Interferon-induced 35 kDa protein	IFI35	0,914327512	128620000
Transmembrane emp24 domain-containing protein 10	TMED10	0,913909706	13220000000
Actin-related protein 2/3 complex subunit 5	ARPC5	0,913909706	9324200000
Fatty acyl-CoA reductase 1	FAR1	0,91382619	1050900000
CD44 antigen	CD44	0,91374269	28894000
Aspartate aminotransferase;Aspartate aminotransferase, cytoplasmic	GIG18;GOT1	0,912908527	31190000000
U1 small nuclear ribonucleoprotein 70 kDa	SNRNP70	0,912825194	3525600000
TBC1 domain family member 23	TBC1D23	0,912658574	129540000
RNA-binding protein 14	RBM14	0,912492016	22111000
cTAGE family member 5	CTAGE5	0,911909539	369310000
Protein NRDE2 homolog	C14orf102;NRDE2	0,911826388	606620000
Fas apoptotic inhibitory molecule 1	FAIM	0,911826388	1024800000
Epidermal growth factor receptor kinase substrate 8-like protein 2	EPS8L2	0,911660133	45099000
Translocating chain-associated membrane protein;Translocating chain-associated membrane protein 1	TRAM1	0,911577028	673490000
Signal transducer and activator of transcription;Signal transducer and activator of transcription 2	STAT2;DKFZp686H05229	0,911577028	414560000
Mitochondrial carrier homolog 2	MTCH2	0,911493939	5985200000
MLN64 N-terminal domain homolog	STARD3NL	0,911410864	55900000
Activating signal cointegrator 1 complex subunit 3	ASCC3	0,91124476	3186200000
Pterin-4-alpha-carbinolamine dehydratase 2	PCBD2	0,911078717	83978000
GEM-interacting protein	GMIP	0,911078717	682790000
Tropomyosin alpha-3 chain	TPM3;HEL-S-82p	0,910995718	908200000
Utrophin	UTRN	0,910746812	259930000
Protein-arginine deiminase type-3;Protein-arginine deiminase type-4	PADI3;PADI4	0,910415149	503190000
Ras-related protein Rab-5C	RAB5C	0,910332271	9014900000
Kinesin-like protein KIF11	KIF11	0,910249408	1454900000
Oxysterol-binding protein-related protein 11	OSBPL11	0,910083728	133020000
2-oxoisovalerate dehydrogenase subunit alpha, mitochondrial	BCKDHA	0,909918107	516220000
Tryptophan--tRNA ligase, cytoplasmic;T1-TrpRS;T2-TrpRS	WARS	0,90966979	9984000000
Membrane-associated progesterone receptor component 2	PGRMC2	0,90966979	1615000000
Septin-1	01-Sep	0,909090909	5104000000
ADP-ribosylation factor-like protein 8B	ARL8B	0,908677874	9745000000
Sperm-specific antigen 2	SSFA2	0,908430233	89310000
Zinc transporter SLC39A7	SLC39A7	0,908347716	189160000
Vesicle-associated membrane protein 4	VAMP4	0,908100254	490720000
General transcription factor 3C polypeptide 3	GTF3C3	0,908100254	82514000
Anaphase-promoting complex subunit 5	ANAPC5	0,908017797	277380000

Transmembrane 9 superfamily member 4	TM9SF4	0,907852928	703390000
Phosphatidylethanolamine-binding protein 1;Hippocampal cholinergic neurostimulating peptide	PEBP1	0,907523369	10853000000
Polymerase delta-interacting protein 2	POLDIP2	0,907441016	1060200000
NADH dehydrogenase [ubiquinone] flavoprotein 2, mitochondrial	NDUFV2	0,907358679	1603200000
Vasodilator-stimulated phosphoprotein	VASP	0,907276356	4055300000
Cyclin-Y;Cyclin-Y-like protein 1;Cyclin-Y-like protein 2	CCNY;CCNYL1;CCNYL2	0,907194049	220610000
Bifunctional polynucleotide phosphatase/kinase;Polynucleotide 3-phosphatase;Polynucleotide 5-hydroxyl-kinase	PNKP	0,906700517	88706000
Ras-related protein Rab-10	RAB10	0,906618314	11280000000
RNA-binding protein PNO1	PNO1	0,906618314	839260000
Protein sel-1 homolog 1	SEL1L	0,90628965	1358600000
Septin-6	06-Sep	0,906207522	1343000000
ADP-ribosylation factor-like protein 15	ARL15	0,905961225	47053000
Histidine triad nucleotide-binding protein 2, mitochondrial	HINT2	0,905797101	520580000
E3 ubiquitin-protein ligase TRIM23	TRIM23	0,905797101	771260000
		0,905715062	32072000
Ras association domain-containing protein 2	RASSF2	0,905715062	911540000
Transmembrane emp24 domain-containing protein 2	RNP24;TMED2	0,905551028	8177500000
Gephyrin;Molybdopterin adenylyltransferase;Molybdopterin molybdenumtransferase	GPHN;GPHRYN	0,905551028	3403200000
Unconventional myosin-IXb	MYO9B variant protein;MYO9B	0,905141202	657790000
N-terminal Xaa-Pro-Lys N-methyltransferase 1;N-terminal Xaa-Pro-Lys N-methyltransferase 1, N-terminally processed	NTMT1	0,905059281	584590000
Apoptosis regulator BAX	BAX	0,904895485	7495300000
Actin, cytoplasmic 1;Actin, cytoplasmic 1, N-terminally processed	PS1TP5BP1;ACTB	0,904731747	26299000000
Small nuclear ribonucleoprotein-associated protein;Small nuclear ribonucleoprotein-associated proteins B and B;Small nuclea	SNRPN;SNRPB	0,9046499	21384000000
CCR4-NOT transcription complex subunit 7	CNOT7	0,904486252	481660000
5-3 exoribonuclease 2	XRN2	0,90440445	2533300000
Fragile X mental retardation syndrome-related protein 2	FXR2	0,904322662	512610000
Proto-oncogene vav	VAV1	0,904077389	909020000
Calcium-binding mitochondrial carrier protein Aralar2	SLC25A13	0,903587241	2577500000
Unconventional myosin-Ic	MYO1C	0,903587241	3749800000
Lipopolysaccharide-responsive and beige-like anchor protein	LRBA	0,903505602	1875300000
Actin-related protein 2/3 complex subunit 2	ARPC2	0,903423977	37578000000
Mitotic spindle-associated MMXD complex subunit MIP18	FAM96B	0,903423977	763380000
E3 ubiquitin-protein ligase synoviolin	SYVN1;FLJ00221	0,903260771	324660000
Serine/threonine-protein kinase mTOR	MTOR	0,903179191	2176500000
Coiled-coil domain-containing protein 22	CCDC22	0,903097625	422650000
Transmembrane emp24 domain-containing protein 9	TMED9	0,903016074	13288000000
DNA-binding protein SMUBP-2	IGHMBP2	0,903016074	152000000
[Pyruvate dehydrogenase [acetyl-transferring]]-phosphatase 2, mitochondrial	PDP2	0,902934537	14713000
U1 small nuclear ribonucleoprotein C	SNRPC	0,902608539	999150000
N-acetylgalactosamine kinase	GALK2	0,902445628	563130000
	FLJ44955	0,902445628	61193000
Protein unc-13 homolog D	UNC13D	0,902445628	920730000
Glycogen debranching enzyme;4-alpha-glucanotransferase;Amylo-alpha-1,6-glucosidase	AGL	0,902282775	672640000
Poly(ADP-ribose) glycohydrolase	PARG	0,902119982	98561000
UNC119-binding protein C5orf30	C5orf30	0,902038607	53996000
Dolichyl-diphosphooligosaccharide--protein glycosyltransferase subunit DAD1	DAD1	0,901957247	8363600000
3-hydroxyacyl-CoA dehydrogenase type-2	HSD17B10	0,901957247	5345900000
Slit homolog 2 protein;Slit homolog 2 protein N-product;Slit homolog 2 protein C-product	SLIT2	0,901794571	1527000000
Rho GDP-dissociation inhibitor 2	ARHGDIB	0,901550667	20584000000
WD repeat- and FYVE domain-containing protein 4	hCG_1745555;WDFY4	0,901306895	775050000
Ras-related protein Rab-2A	RAB2A	0,901225667	16637000000
Signal peptidase complex subunit 2	SPCS2	0,901225667	11434000000
Pre-mRNA 3-end-processing factor FIP1	FIP1L1	0,901225667	337910000
Inactive hydroxysteroid dehydrogenase-like protein 1	HSDL1	0,901063255	397210000
Alpha-mannosidase 2C1	MAN2C1	0,901063255	415840000
GON-4-like protein	GON4L	0,90098207	826890000
Tubulin-specific chaperone D	TBCD	0,900576369	1082300000
AP-3 complex subunit sigma-1	AP3S1	0,900576369	1535000000
	TUBB6	0,900414191	1663900000
GTP:AMP phosphotransferase AK3, mitochondrial	AK3	0,900333123	7690500000
Cytochrome b-c1 complex subunit 2, mitochondrial	UQCRC2	0,900252071	6517100000
Dynamin-1-like protein	DNM1L	0,900171032	6971300000
Histone deacetylase complex subunit SAP18	SAP18	0,900171032	397810000
Staphylococcal nuclease domain-containing protein 1	SND1	0,900090009	22049000000
Protein RMD5 homolog A	RMND5A	0,900009	229920000
NEDD4-binding protein 2	N4BP2	0,899928006	85481000
RNA 3-terminal phosphate cyclase-like protein	RCL1	0,899766061	74280000
Small nuclear ribonucleoprotein Sm D1	SNRPD1	0,899442346	9115100000
Peptidyl-tRNA hydrolase 2, mitochondrial	PTRH2	0,899361453	1110600000
Dolichyl-diphosphooligosaccharide--protein glycosyltransferase subunit STT3B	STT3B	0,899118864	973960000
Cyclin-dependent kinase 13	CDK13	0,899038029	58407000
Vam6/Vps39-like protein	VPS39	0,89895721	568630000
Protein jagunal homolog 1	JAGN1	0,898714838	1516800000
HD domain-containing protein 2	HDDC2	0,898714838	43152000
Exocyst complex component 8	EXOC8	0,898634076	190520000
Interferon-induced very large GTPase 1	GVINP1	0,898553329	70915000
Protein BRICK1	BRK1	0,898472597	249960000
MOB-like protein phocein	HSPE1-MOB4;MOB4	0,898069151	19837000000
Peptidyl-prolyl cis-trans isomerase;Peptidyl-prolyl cis-trans isomerase FKBP2	FKBP2	0,898069151	5569700000
Cytosolic 5-nucleotidase 3A	NT5C3A	0,898069151	11879000000
Retinol dehydrogenase 13	RDH13	0,898069151	230170000
ATP synthase subunit alpha, mitochondrial	ATP5A1	0,897907875	1,5628E+11
Rab-like protein 6	RABL6	0,897907875	183680000
78 kDa glucose-regulated protein	HSPA5	0,897907875	2,089E+11
Zinc finger protein 638	ZNF638	0,897907875	67631000
Eukaryotic translation initiation factor 4E type 3	EIF4E3	0,897827258	387560000
Vesicle-associated membrane protein-associated protein A	VAPA	0,897827258	14511000000
39S ribosomal protein L22, mitochondrial	MRPL22	0,897585495	545260000
Glycogen [starch] synthase, muscle	GYS1	0,897504936	2454700000
ATP-dependent zinc metalloprotease YME1L1	YME1L1;FTSH	0,897424392	337830000
ADP-ribosylation factor 4	ARF4	0,897424392	1591200000
Delta(24)-sterol reductase	DHCR24;Nbla03646	0,897263347	553650000
Calumenin	CALU	0,897263347	409900000
Vacuolar fusion protein MON1 homolog A	MON1A	0,897182846	281540000
Calpain-2 catalytic subunit	CAPN2	0,895977063	1732000000
Putative helicase MOV-10	MOV10	0,895896793	321170000
N-acetyltransferase 10	NAT10	0,895656068	175320000
Mitochondrial import receptor subunit TOM70	TOMM70A	0,895255148	4554000000
Multidrug resistance-associated protein 1	ABCC1;DKFZp781G125;MRP	0,89509488	12102000000
Protein Hikeshi	C11orf73	0,895014768	488470000
Protein OS-9	OS9;OR7D2	0,894774517	91863000
Long-chain-fatty-acid--CoA ligase 4	FACL4;ACSL4	0,894774517	1408900000
Protein phosphatase 1 regulatory subunit 7	PPP1R7	0,894694462	6725700000
Mannosyl-oligosaccharide 1,2-alpha-mannosidase IB	MAN1A2	0,894454383	109470000
Fibronectin type-III domain-containing protein 3A	FNDC3A	0,894374385	337950000
ATP synthase subunit O, mitochondrial	ATP5O	0,894294402	6729700000
UPF0160 protein MYG1, mitochondrial	C12orf10;MST024	0,894214433	2661400000
Bromodomain-containing protein 1	BRD1;DKFZp434B094	0,894214433	272230000

Neuroblastoma-amplified sequence	NBAS	0,894134478	288590000
RWD domain-containing protein 1	RWDD1	0,893974611	463500000
Cytochrome P450 20A1	CYP20A1	0,893894699	866080000
Hydroxymethylglutaryl-CoA lyase, mitochondrial;3-hydroxymethyl-3-methylglutaryl-CoA lyase, cytoplasmic	HMGCL;HMGCLL1;DKFZp434G14	0,893734918	534110000
Tyrosine-protein phosphatase non-receptor type;Tyrosine-protein phosphatase non-receptor type 6	PTPN6	0,893734918	2986500000
ATP-binding cassette sub-family B member 7, mitochondrial	ABCB7	0,893655049	1186100000
Endoribonuclease Dicer	DICER1	0,893575194	164260000
DNA-directed RNA polymerase III subunit RPC1;DNA-directed RNA polymerase	POLR3A	0,893495354	427230000
GPI ethanolamine phosphate transferase 3	PIGO	0,893495354	57856000
Vacuole membrane protein 1	TMEM49;VMP1	0,893335716	189370000
Mitochondrial carnitine/acylcarnitine carrier protein	SLC25A20	0,893255918	1668000000
Vacuolar protein sorting-associated protein 33B	VPS33B	0,893255918	564970000
ATP-dependent RNA helicase A	DHX9	0,893176134	8256000000
Citrate synthase;Citrate synthase, mitochondrial	CS	0,89301661	26902000000
1-phosphatidylinositol 4,5-bisphosphate phosphodiesterase beta-3	PLCB3	0,892697733	1810500000
Dynamin-2	DNM2	0,892538379	10014000000
Synaptosomal-associated protein;Synaptosomal-associated protein 29	SNAP29	0,892538379	269040000
Calcium-binding mitochondrial carrier protein Aralar1	SLC25A12	0,892379083	7514100000
Gamma-soluble NSF attachment protein	NAPG	0,892379083	2767500000
Succinyl-CoA ligase [ADP-forming] subunit beta, mitochondrial	SUCLA2;DKFZp686D0880;DKFZp5	0,892299456	3066300000
Bcl-2-like protein 1	BCL2L1	0,892299456	1388300000
Acyl-protein thioesterase 2	LYPLA2	0,892299456	6259400000
60S ribosome subunit biogenesis protein NIP7 homolog	NIP7	0,892140244	944570000
cAMP-dependent protein kinase catalytic subunit alpha	PRKACA	0,892140244	8924600000
Mitogen-activated protein kinase 12	MAPK12;DKFZp686M02159	0,892140244	2122400000
	CREBBP	0,891901534	542960000
Neudesin	NENF	0,891821992	230420000
Membrane-associated progesterone receptor component 1	PGRMC1	0,891742465	4576000000
Barrier-to-autointegration factor;Barrier-to-autointegration factor, N-terminally processed	BANF1	0,891583452	5895000000
HEAT repeat-containing protein 3	HEATR3	0,891503967	555990000
GTP-binding protein SAR1b	SAR1B;DKFZp434B2017	0,891424496	11617000000
Fructose-bisphosphate aldolase C	ALDOC	0,891424496	21589000000
Hypoxanthine-guanine phosphoribosyltransferase	HPRT1	0,891186169	29595000000
FUN14 domain-containing protein 2	FUNDC2	0,890709896	1015700000
Serine/threonine-protein kinase A-Raf	ARAF	0,890709896	187850000
Selenocysteine lyase	SCLY	0,890551251	433570000
ATP-dependent RNA helicase DDX1	DDX1	0,89047195	9823500000
NADH dehydrogenase [ubiquinone] 1 alpha subcomplex subunit 8	NDUFA8	0,890392663	1517800000
Echinoderm microtubule-associated protein-like 4;Tyrosine-protein kinase receptor	EML4;EML4-ALK variant 6;EML4-AL	0,89031339	2200300000
Phosphatidylinositol 4,5-bisphosphate 3-kinase catalytic subunit delta isoform	PIK3CD	0,890154887	1202600000
Protein THEM6	THEM6	0,890075656	2244900000
MMS19 nucleotide excision repair protein homolog	MMS19	0,889838049	2134900000
Aminopeptidase B	RNPEP	0,889363216	1082700000
U7 snRNA-associated Sm-like protein LSm11	LSM11	0,889284126	122940000
Dolichol-phosphate mannosyltransferase subunit 1	DPM1	0,889046942	4759900000
Neurofibromin;Neurofibromin truncated	NF1	0,888967908	762180000
Ras-related C3 botulinum toxin substrate 2	RAC2	0,888967908	41402000000
Kinase D-interacting substrate of 220 kDa	KIDINS220	0,888888889	268820000
Heterogeneous nuclear ribonucleoprotein U-like protein 1	HNRNPUL1	0,888888889	2972800000
E3 ubiquitin-protein ligase RNF213	RNF213	0,888809884	1515700000
Peptide-N(4)-(N-acetyl-beta-glucosaminyl)asparagine amidase	NGLY1	0,888651915	301270000
Cathepsin Z	CTSZ	0,888572952	3746100000
Fructose-bisphosphate aldolase A	ALDOA	0,888572952	3,2726E+11
Autophagy-related protein 2 homolog B	ATG2B	0,888178346	52741000
Phostensin	PPP1R18	0,887941751	33542000
Bcl-2-associated transcription factor 1	BCLAF1	0,887862914	921130000
Aldo-keto reductase family 1 member B10	AKR1B10	0,887705282	7198300000
Actin-related protein 2/3 complex subunit 4	ARPC4-TTLL3;ARPC4	0,887390185	30567000000
Polyribonucleotide nucleotidyltransferase 1, mitochondrial	PNPT1	0,887311446	2572100000
	TPP2	0,887311446	101760000
Methylthioribulose-1-phosphate dehydratase	APIP	0,887311446	500880000
Adaptin ear-binding coat-associated protein 1	NECAP1	0,88715401	135030000
Density-regulated protein	DENR	0,887075313	2493800000
Tetratricopeptide repeat protein 9C	TTC9C	0,88691796	583460000
Neutral alpha-glucosidase AB	GANAB	0,886839305	29060000000
CAAX prenyl protease 2	RCE1	0,886839305	106510000
DDB1- and CUL4-associated factor 8	DCAF8	0,886682036	239830000
Enhancer of mRNA-decapping protein 4	EDC4	0,886603422	3007800000
Lysophospholipid acyltransferase LPCAT4	LPCAT4	0,886524823	66377000
SUZ domain-containing protein 1	SZRD1	0,886446237	584840000
Heat shock protein 75 kDa, mitochondrial	TRAP1	0,886053518	7799900000
Protein disulfide-isomerase	P4HB	0,886053518	51763000000
Early endosome antigen 1	EEA1	0,886053518	4775900000
Four and a half LIM domains protein 3	FHL3	0,885975016	1060500000
10 kDa heat shock protein, mitochondrial	EPFP1;HSPE1	0,885896527	33909000000
Pinin	PNN	0,885896527	525250000
Very-long-chain (3R)-3-hydroxyacyl-CoA dehydratase 2	PTPLB	0,885818053	247010000
Ubinuclein-2	UBN2	0,885818053	175360000
Glucosamine 6-phosphate N-acetyltransferase	GNPNAT1	0,885739593	5206800000
Persulfide dioxygenase ETHE1, mitochondrial	ETHE1	0,885504295	988790000
RNA-binding protein 10	DKFZp686E2459;RBM10	0,88542589	302610000
Phosphoserine aminotransferase	PSAT1	0,885190759	71759000000
Phosphoacetylglucosamine mutase	PGM3	0,885190759	453430000
Latrophilin-2	LPHN2	0,884642604	3750100000
Single-stranded DNA-binding protein 2;Single-stranded DNA-binding protein 3;Single-stranded DNA-binding protein 4	SSBP4;SSBP2;SSBP3	0,884407889	80694000
Dolichol-phosphate mannosyltransferase subunit 3	DPM3	0,884329678	288040000
NAD(P) transhydrogenase, mitochondrial	NNT	0,883782589	4662600000
Mitochondrial glutamate carrier 1;Mitochondrial glutamate carrier 2	SLC25A22;SLC25A18	0,883782589	1158600000
BAG family molecular chaperone regulator 4	BAG4	0,883782589	8585900
Disintegrin and metalloproteinase domain-containing protein 17	ADAM17	0,883782589	24489000
Malignant T-cell-amplified sequence 1	MCTS1	0,88354833	5121100000
MIT domain-containing protein 1	MITD1	0,883314195	169550000
Macrophage-capping protein	CAPG	0,882768362	28652000000
Prohibitin	PHB;HEL-S-54e	0,882612533	98651000000
Glyoxalase domain-containing protein 4	C17orf25;GLOD4	0,882612533	3841600000
Protein NDRG1	TRG14;NDRG1	0,882534639	331680000
Ras-related protein Rab-4B	RAB4B	0,882534639	2007000000
	NME1-NME2	0,882378893	270530000
PIH1 domain-containing protein 1	PIH1D1	0,882378893	54886000
Cytochrome b-c1 complex subunit 7		0,882067566	744600000
Huntingtin	HTT;HD	0,881989769	1207700000
NFU1 iron-sulfur cluster scaffold homolog, mitochondrial	NFU1	0,881600987	2315000000
GPI ethanolamine phosphate transferase 1	PIGN	0,881523272	195900000
Manganese-transporting ATPase 13A1	ATP13A1	0,881367883	1487800000
Dihydrolipoyllysine-residue succinyltransferase component of 2-oxoglutarate dehydrogenase complex, mitochondrial	DLST;E2k	0,881290209	4189600000
Protein disulfide-isomerase A4	PDIA4	0,880979649	15899000000
Solute carrier family 35 member B1	SLC35B1	0,880746873	175600000
GTPase NRas	NRAS	0,880669309	548510000

Serine--tRNA ligase, cytoplasmic	SARS	0,880591758	18405000000
Dedicator of cytokinesis protein 5	DOCK5;DKFZp779M164;DKFZp78	0,880591758	978990000
Prohibitin-2	PHB2	0,880359187	83200000000
Apoptosis-inducing factor 1, mitochondrial	AIFM1	0,880359187	2302600000
Ribonuclease 3	DROSHA	0,880204207	17137000
Asparagine synthetase [glutamine-hydrolyzing];Asparagine synthetase	ASNS	0,880049283	8629200000
Solute carrier family 2, facilitated glucose transporter member 1	SLC2A1	0,879894413	592800000
[3-methyl-2-oxobutanoate dehydrogenase [lipoamide]] kinase, mitochondrial	BCKDK	0,879816998	155090000
Peroxisome assembly factor 2	PEX6	0,879584836	39703000
Tumor protein D52	TPD52	0,879507476	526690000
AP-1 complex subunit gamma-like 2	AP1G2	0,879507476	278430000
Glycerol kinase;Putative glycerol kinase 3	GK;GK3P	0,879043601	640710000
E3 ubiquitin-protein ligase SMURF1	DKFZp564H223;SMURF1	0,878966336	1120500000
ADP/ATP translocase 2;ADP/ATP translocase 2, N-terminally processed	SLC25A5	0,878889084	3,4198E+11
Reticulon-4-interacting protein 1, mitochondrial	RTN4IP1;NIMP	0,878889084	220450000
39S ribosomal protein L44, mitochondrial	MRPL44	0,878657411	598930000
ATP-dependent RNA helicase SUPV3L1, mitochondrial	SUPV3L1	0,878657411	327700000
Malate dehydrogenase, cytoplasmic;Malate dehydrogenase	MDH1	0,878657411	15469000000
Sentrin-specific protease 3	SENP3	0,878657411	32349000
Autophagy-related protein 101	ATG101	0,878503031	28621000
Microtubule-associated protein 1A;MAP1A heavy chain;MAP1 light chain LC2	MAP1A	0,878425861	586750000
Small nuclear ribonucleoprotein G;Putative small nuclear ribonucleoprotein G-like protein 15	SNRPG;SNRPGP15	0,878425861	4464300000
Acetyl-CoA acetyltransferase, cytosolic	ACAT2	0,878348704	3149100000
Pyroglutamyl-peptidase 1	PGPEP1	0,878348704	349280000
G1/S-specific cyclin-D2	CCND2	0,878271562	235400000
ES1 protein homolog, mitochondrial	C21orf33	0,878117316	2911300000
GDP-L-fucose synthase	TSTA3	0,878117316	991310000
Proteasome activator complex subunit 2	PSME2	0,877963126	13121000000
Peroxisomal carnitine O-octanoyltransferase	CROT	0,877731941	26245000
Trifunctional enzyme subunit alpha, mitochondrial;Long-chain enoyl-CoA hydratase;Long chain 3-hydroxyacyl-CoA dehydro	HADHA	0,877654906	4274000000
Structural maintenance of chromosomes flexible hinge domain-containing protein 1	SMCHD1	0,877500878	2419200000
D-3-phosphoglycerate dehydrogenase	PHGDH;PGDH3	0,877423883	40428000000
Dihydroxyacetone phosphate acyltransferase	GNPAT	0,877346903	144870000
MICOS complex subunit MIC19	CHCHD3	0,877346903	4462300000
Protein FAM98B	FAM98B	0,877346903	679810000
Sorcin	SRI	0,877192982	23056000000
	ATP5H	0,876885303	2796100000
Mitochondrial carrier homolog 1	PIG60;MTCH1	0,876424189	786630000
Putative ATP-dependent RNA helicase DHX30	DHX30	0,876040298	417070000
Gamma-tubulin complex component 3	TUBGCP3	0,876040298	295960000
Zinc finger ZZ-type and EF-hand domain-containing protein 1	ZZEF1	0,875886835	656100000
Protein TBRG4	TBRG4	0,875656743	101460000
NF-kappa-B inhibitor-interacting Ras-like protein 2	NKIRAS2	0,87535014	243760000
Transmembrane protein 43	TMEM43;FLJ00144	0,875043752	2917200000
Armadillo repeat-containing protein 1	ARMC1	0,874967189	735980000
Pre-mRNA-splicing factor CWC22 homolog	CWC22	0,874967189	210320000
Gamma-tubulin complex component 2	TUBGCP2	0,874814102	178500000
2-oxoglutarate dehydrogenase, mitochondrial	OGDH	0,874584572	9200100000
L-fucose kinase	FUK	0,874431619	88205000
Ral GTPase-activating protein subunit beta	RALGAPB	0,87427872	431090000
Rho GTPase-activating protein 25	KIAA0053;ARHGAP25	0,873973082	524740000
Myotubularin-related protein 5	SBF1	0,873591334	515250000
Transcription elongation factor B polypeptide 3	TCEB3	0,873438728	59912000
Aspartyl aminopeptidase	DNPEP	0,873286176	362990000
Ribosome-recycling factor, mitochondrial	MRRF	0,873133677	165260000
Vesicle transport through interaction with t-SNAREs homolog 1A	VTI1A	0,872981231	1264900000
Glutathione S-transferase omega-1	GSTO1	0,872981231	8685600000
Ubiquinol-cytochrome-c reductase complex assembly factor 1	UQCC1;UQCC	0,872752662	454240000
Tetratricopeptide repeat protein 7A	TTC7A	0,872600349	141750000
Serine protease HTRA2, mitochondrial	HTRA2	0,872524213	976580000
Rho GTPase-activating protein 9	ARHGAP9	0,872448089	114400000
		0,872371979	80562000
Reticulon-4;Reticulon	RTN4;Nbla00271;NOGOC	0,872067672	11272000000
ATP synthase F(0) complex subunit C2, mitochondrial;ATP synthase F(0) complex subunit C1, mitochondrial;ATP synthase	ATP5G2;ATP5G1;ATP5G3	0,871915599	1573400000
NF-kappa-B essential modulator	IKBKG	0,871839582	279880000
Tumor necrosis factor alpha-induced protein 8-like protein 2	TNFAIP8L2	0,871004268	139100000
Protein ERGIC-53	LMAN1	0,870700914	1393500000
Ceramide synthase 2	CERS2	0,870625109	1116900000
1-phosphatidylinositol 4,5-bisphosphate phosphodiesterase beta-2;Phosphoinositide phospholipase C	PLCB2	0,86979212	123290000
Glutamate dehydrogenase;Glutamate dehydrogenase 1, mitochondrial;Glutamate dehydrogenase 2, mitochondrial	GLUD1;GLUD2	0,869716472	9017400000
Alpha-actinin-2	ACTN2	0,86948961	279690000
Melanoma inhibitory activity protein 3	MIA3	0,869338433	360510000
Autophagy protein 5	ATG5	0,868809731	156090000
Arginase-1	ARG1	0,868734254	380220000
Transcriptional activator protein Pur-beta	PURB	0,868507903	2714100000
Amyloid beta A4 precursor protein-binding family B member 1-interacting protein	APBB1IP	0,868281671	147010000
Protein LYRIC	MTDH	0,868206286	3019600000
Glycerol-3-phosphate dehydrogenase, mitochondrial	GPD2	0,86798021	2713000000
Calumenin	CALU	0,86798021	8062000000
AT-rich interactive domain-containing protein 1B	ARID1B	0,86798021	57481000
Constitutive coactivator of PPAR-gamma-like protein 1	FAM120A	0,867528412	2834500000
Kinesin-like protein KIF13A	KIF13A	0,867377917	16543000
Retinol dehydrogenase 14	RDH14	0,867302689	540070000
Inorganic pyrophosphatase 2, mitochondrial	PPA2	0,867077083	624970000
Cancer-related nucleoside-triphosphatase	C1orf57;NTPCR	0,867001907	313750000
Actin-binding protein anillin	ANLN	0,866851595	18773000
Zinc-binding alcohol dehydrogenase domain-containing protein 2	ZADH2	0,866776458	456740000
Nuclear cap-binding protein subunit 2	NCBP2	0,866701335	139490000
NADH dehydrogenase [ubiquinone] 1 alpha subcomplex subunit 5	NDUFA5	0,866551127	601380000
Pyruvate kinase PKM;Pyruvate kinase	PKM;PKM2	0,866325912	1,9973E+11
Macrophage migration inhibitory factor	MIF	0,866250866	9288400000
TFIIH basal transcription factor complex helicase XPD subunit	ERCC2	0,866250866	194050000
Cytoplasmic FMR1-interacting protein 2	CYFIP2;DKFZp761H087	0,866100814	9064100000
Probable aminopeptidase NPEPL1	NPEPL1	0,866025808	709980000
Protein odr-4 homolog	ODR4	0,865576041	233140000
Synaptosomal-associated protein;Synaptosomal-associated protein 23	SNAP23	0,865501125	916330000
Glutamine--tRNA ligase	QARS	0,865501125	2301600000
2-oxoisovalerate dehydrogenase subunit beta, mitochondrial	BCKDHB	0,865351333	784650000
NADH-cytochrome b5 reductase 3;NADH-cytochrome b5 reductase 3 membrane-bound form;NADH-cytochrome b5 reduct	CYB5R3	0,865201592	2958900000
Aldehyde dehydrogenase family 16 member A1	ALDH16A1	0,864154857	562240000
Signal peptidase complex subunit 3	SPCS3	0,86400553	6907000000
Voltage-dependent anion-selective channel protein 2	VDAC2	0,863930886	31273000000
Protein YIPF3;Protein YIPF3, 36 kDa form III	YIPF3	0,863856254	20941000
Translocon-associated protein subunit delta	SSR4	0,863781636	7941400000
		0,863632438	760820000
Galectin-1	LGALS1	0,863259669	35413000000
Mitochondrial import receptor subunit TOM40 homolog	TOMM40	0,863110651	3877700000
Gamma-enolase;Enolase	ENO2	0,863036161	22011000000

Annexin;Annexin A5	ANXA5	0,862887221	56975000000
Coiled-coil domain-containing protein 178	CCDC178;C18orf34	0,862663906	2172600000
GMP reductase 1	GMPR	0,862291972	470240000
Hyaluronan synthase 2	HAS2	0,862068966	728790000
Protein FAM118B	FAM118B	0,862068966	676910000
Protein mago nashi homolog;Protein mago nashi homolog 2	MAGOH;MAGOHB	0,861994656	5533500000
Pyridoxal-dependent decarboxylase domain-containing protein 1;Putative pyridoxal-dependent decarboxylase domain-conta	PDXDC1;PDXDC2P	0,861994656	387950000
Phosphatidylinositol 4-kinase alpha	PI4KA;PIK4CA variant protein;hCG_	0,861920359	5614400000
ATP-dependent (S)-NAD(P)H-hydrate dehydratase	FLJ10769;CARKD	0,860807437	434910000
Protein canopy homolog 2	CNPY2	0,860807437	3384500000
PDZ domain-containing protein 8	PDZD8	0,860733345	673680000
Oxygen-dependent coproporphyrinogen-III oxidase, mitochondrial	CPOX	0,860141063	1327300000
		0,860141063	246000000
NADH-cytochrome b5 reductase 1	CYB5R1	0,860141063	2486300000
Isocitrate dehydrogenase [NAD] subunit gamma, mitochondrial	IDH3G;hCG_2004980	0,860067085	11347000000
Multidrug resistance-associated protein 4	ABCC4;MOAT-B	0,860067085	396210000
7-dehydrocholesterol reductase	DHCR7	0,85999312	735310000
Procollagen-lysine,2-oxoglutarate 5-dioxygenase 3	PLOD3;DKFZp564O1822	0,859623485	1002000000
Rapamycin-insensitive companion of mTOR	RICTOR	0,859549596	239090000
Isoleucine--tRNA ligase, cytoplasmic	DKFZp686L0869;IARS;DKFZp686L	0,85947572	16922000000
Elongation of very long chain fatty acids protein;Elongation of very long chain fatty acids protein 5	ELOVL5	0,859254167	504420000
Procollagen-lysine,2-oxoglutarate 5-dioxygenase 1	PLOD1	0,858811405	552270000
H(+)/Cl(-) exchange transporter 5;H(+)/Cl(-) exchange transporter 4	CLCN5;CLCN4	0,858516484	161780000
Isocitrate dehydrogenase [NAD] subunit alpha, mitochondrial	IDH3A	0,858442785	32118000000
Long-chain fatty acid transport protein 4	SLC27A4	0,858295425	39809000
Syntaxin-binding protein 3	STXBP3	0,858148116	193920000
DnaJ homolog subfamily C member 11	DNAJC11	0,858000858	303250000
Zinc finger MYM-type protein 4	ZMYM4	0,857706493	83434000
Vacuolar protein sorting-associated protein 52 homolog	VPS52	0,857632933	243920000
ATP synthase subunit epsilon-like protein, mitochondrial;ATP synthase subunit epsilon, mitochondrial	ATP5EP2;ATP5E	0,857559386	2807600000
Heterogeneous nuclear ribonucleoprotein U-like protein 2	HNRNPUL2-BSCL2;HNRNPUL2	0,857485851	1743800000
Thioredoxin domain-containing protein 9	TXNDC9	0,857485851	55252000
Integrin alpha-4	ITGA4	0,85741233	327980000
Cytoplasmic dynein 1 heavy chain 1	DYNC1H1	0,857265324	1,1924E+11
Mitochondrial import receptor subunit TOM5 homolog	TOMM5	0,856971463	388720000
		0,856971463	1460500000
Golgin subfamily A member 5	GOLGA5	0,856824608	51266000
		0,856751199	355760000
Elongation factor Ts;Elongation factor Ts, mitochondrial	TSFM	0,856751199	1127400000
Ubiquitin-conjugating enzyme E2 H	UBE2H	0,856531049	1219500000
NADH dehydrogenase [ubiquinone] flavoprotein 1, mitochondrial	NDUFV1	0,856311012	3415200000
DNA polymerase;DNA polymerase delta catalytic subunit	POLD1	0,856164384	4870600000
Lymphocyte cytosolic protein 2	LCP2	0,856164384	1087200000
Vesicle transport protein SFT2A	SFT2D1	0,855944535	48490000
Glycerol kinase 2	GK2	0,855724799	129550000
Asparagine--tRNA ligase, cytoplasmic	NARS	0,855431993	16118000000
Pre-mRNA-splicing factor ATP-dependent RNA helicase PRP16	DHX38	0,855285665	959630000
Transmembrane protein C16orf54	C16orf54	0,855066268	83775000
Translocation protein SEC62	SEC62;TLOC1	0,85499316	456010000
	ATP5B	0,854554777	136000000
ATP synthase subunit gamma;ATP synthase subunit gamma, mitochondrial	ATP5C1	0,854481757	12894000000
A disintegrin and metalloproteinase with thrombospondin motifs 14	ADAMTS14	0,854408749	744970000
ADP-ribosylation factor GTPase-activating protein 3	ARFGAP3	0,854262771	27801000
Glycosylphosphatidylinositol anchor attachment 1 protein	GPAA1	0,854043898	189900000
Trifunctional enzyme subunit beta, mitochondrial;3-ketoacyl-CoA thiolase	HADHB	0,853752241	5660200000
Endoplasmic reticulum-Golgi intermediate compartment protein 3	ERGIC3	0,853606487	474340000
Tuftelin-interacting protein 11	TFIP11	0,853606487	44418000
TATA-binding protein-associated factor 2N	TAF15	0,853460783	55511000
Ubiquitin-protein ligase E3A	UBE3A	0,85338795	1931000000
Zinc transporter 6	SLC30A6	0,853315129	73078000
ATP synthase F(0) complex subunit B1, mitochondrial	ATP5F1;hCG_39985	0,853096741	7524200000
UMP-CMP kinase	CMPK1;CMPK	0,85302397	21399000000
Importin-13	IPO13	0,85302397	74342000
Voltage-dependent anion-selective channel protein 3	VDAC3	0,852733009	31740000000
Phosphatidylinositol 3,4,5-trisphosphate-dependent Rac exchanger 1 protein	PREX1	0,852514919	9755300
		0,852442247	94316000
Golgin subfamily B member 1	GOLGB1	0,852442247	203970000
AP-3 complex subunit beta-1	AP3B1	0,851716208	4468900000
Hypermethylated in cancer 2 protein	HIC2	0,851498638	2204000000
Lambda-crystallin homolog	CRYL1	0,851353652	201480000
TraB domain-containing protein	RP3-402G11.12;TRABD	0,851208716	521760000
UTP--glucose-1-phosphate uridylyltransferase	UGP2	0,85106383	1125100000
Lipoamide acyltransferase component of branched-chain alpha-keto acid dehydrogenase complex, mitochondrial	DBT	0,850918993	1158100000
Cytochrome b5	CYB5A	0,850774205	2813300000
Voltage-dependent anion-selective channel protein 1	VDAC1	0,850051003	42952000000
Amino-terminal enhancer of split	AES	0,849762067	58693000
Anion exchange protein 2	SLC4A2	0,84932903	440320000
Receptor-type tyrosine-protein phosphatase;Receptor-type tyrosine-protein phosphatase alpha	PTPRA	0,849184783	256980000
RAC-alpha serine/threonine-protein kinase	AKT1	0,848824378	166210000
	HO1	0,848752334	561380000
Vacuolar protein sorting-associated protein 45	VPS45	0,848752334	221020000
Acyl-CoA synthetase family member 2, mitochondrial	ACSF2	0,848608282	245080000
Peroxisomal biogenesis factor 3	PEX3	0,848536275	40275000
Cyclic AMP-responsive element-binding protein 1	CREB1	0,848176421	68192000
Group XV phospholipase A2	PLA2G15	0,847744998	754110000
Ribonuclease P protein subunit p25-like protein	RPP25L	0,846740051	159160000
Alanine--tRNA ligase, cytoplasmic	AARS	0,84645336	23434000000
Alkyldihydroxyacetonephosphate synthase, peroxisomal	AGPS	0,84609527	1005500000
Succinate dehydrogenase [ubiquinone] flavoprotein subunit, mitochondrial	SDHA	0,845809016	10094000000
Ras-related protein Rab-18	RAB18	0,845737483	6102000000
Ferritin;Ferritin heavy chain;Ferritin heavy chain, N-terminally processed	FTH1	0,845665962	13243000000
Protein transport protein Sec24D	SEC24D	0,844951415	485540000
Apolipoprotein O	APOO	0,844737287	257860000
Dihydrolipoyl dehydrogenase;Dihydrolipoyl dehydrogenase, mitochondrial	DLD	0,844380647	8739400000
NTF2-related export protein 1	NXT1	0,844238075	28405000
Stromal interaction molecule 1	STIM1	0,843597098	580950000
UPF0317 protein C14orf159, mitochondrial	C14orf159;C14orf159 variant protei	0,843525938	366430000
Arf-GAP with coiled-coil, ANK repeat and PH domain-containing protein 1	ACAP1	0,843525938	1113800000
Rho guanine nucleotide exchange factor 1	ARHGEF1	0,843383655	3149800000
ADP-ribosylation factor-like protein 2	hCG_23373;ARL2	0,843028157	2588400000
Serine beta-lactamase-like protein LACTB, mitochondrial	LACTB	0,842886042	15079000
SLIT-ROBO Rho GTPase-activating protein 2;SLIT-ROBO Rho GTPase-activating protein 2C	SRGAP2;SRGAP2C	0,842815002	557430000
DNA repair protein complementing XP-G cells	BIVM-ERCC5;ERCC5	0,842530963	34968000
Purine nucleoside phosphorylase;S-methyl-5-thioadenosine phosphorylase	MTAP	0,842176183	28380000000
Conserved oligomeric Golgi complex subunit 6	COG6	0,842176183	67814000
Conserved oligomeric Golgi complex subunit 5	COG5	0,841892575	449310000
Phosphorylase b kinase regulatory subunit beta	PHKB	0,841467519	151360000
Probable asparagine--tRNA ligase, mitochondrial	NARS2	0,841396719	29894000

Mitochondrial coenzyme A transporter SLC25A42	SLC25A42	0,841396719	73564000
Protein-methionine sulfoxide oxidase MICAL1	MICAL1	0,841396719	1085900000
Succinyl-CoA ligase [ADP/GDP-forming] subunit alpha, mitochondrial	SUCLG1	0,840760047	9607900000
3-ketoacyl-CoA thiolase, mitochondrial	ACAA2	0,840336134	2511100000
Galectin-related protein	LGALSL	0,840124338	409510000
5(3)-deoxyribonucleotidase, cytosolic type	NT5C	0,839630563	2195100000
Very-long-chain (3R)-3-hydroxyacyl-CoA dehydratase 3	PTPLAD1	0,839560071	1326900000
Mitochondrial Rho GTPase;Mitochondrial Rho GTPase 1	TMEM91;RHOT1	0,838855801	374240000
Single-stranded DNA-binding protein;Single-stranded DNA-binding protein, mitochondrial	SSBP1	0,838785439	1228800000
Round spermatid basic protein 1	RSBN1	0,838293235	75988000
39S ribosomal protein L13, mitochondrial	MRPL13	0,838152711	841640000
Nck-associated protein 1	NCKAP1	0,838152711	174940000
Dimethyladenosine transferase 1, mitochondrial	TFB1M	0,838082467	54050000
Small integral membrane protein 12	C1orf212;SMIM12	0,838012235	51105000
Serine/threonine-protein kinase N2	PKN2	0,837942014	82125000
Mitochondrial-processing peptidase subunit beta	PMPCB;DKFZp586I1223	0,837871806	2976900000
Coronin-1B;Coronin	CORO1B;DKFZp762I166	0,837731423	4460100000
Alpha-enolase;Enolase	ENO1	0,83766125	3,2479E+11
Striatin-3	STRN3	0,83766125	336120000
Succinate dehydrogenase [ubiquinone] iron-sulfur subunit, mitochondrial	SDHB	0,8374508	8298800000
Dehydrogenase/reductase SDR family member 1	DHRS1	0,837100285	1498400000
Translational activator of cytochrome c oxidase 1	TACO1	0,836890116	429730000
Dedicator of cytokinesis protein 10	DOCK10;DKFZp781A1532	0,836610056	4637800000
Protein phosphatase 1F	PPM1F	0,836470096	139230000
Echinoderm microtubule-associated protein-like 2	EML2	0,836120401	1039000000
Medium-chain specific acyl-CoA dehydrogenase, mitochondrial	ACADM;DKFZp686M24262	0,835770999	232880000
NADH dehydrogenase [ubiquinone] 1 alpha subcomplex subunit 12	NDUFA12	0,835561497	236030000
Late secretory pathway protein AVL9 homolog	AVL9	0,835561497	31219000
Long-chain specific acyl-CoA dehydrogenase, mitochondrial	ACADL	0,835142809	2243300000
Aspartate aminotransferase, mitochondrial;Aspartate aminotransferase	GOT2	0,834933623	48610000000
Regulatory-associated protein of mTOR	RPTOR	0,83423709	392110000
Zinc finger Ran-binding domain-containing protein 2	ZRANB2	0,834097923	762440000
Sorting and assembly machinery component 50 homolog	SAMM50	0,83388926	1206500000
39S ribosomal protein L27, mitochondrial	MRPL27	0,833263895	391260000
Mitochondrial 2-oxoglutarate/malate carrier protein	SLC25A11	0,832986256	24371000000
Dedicator of cytokinesis protein 11	DOCK11	0,832986256	1946100000
DNA ligase;DNA ligase 3	LIG3	0,832847506	341250000
Glutathione S-transferase P	GSTP1	0,832639467	1461500000
Threonine--tRNA ligase, cytoplasmic	TARS	0,832292967	14362000000
Probable arginine--tRNA ligase, mitochondrial	RARS2;RARSL	0,832223702	142170000
NADH dehydrogenase [ubiquinone] iron-sulfur protein 4, mitochondrial	NDUFS4	0,832085206	1018300000
Monoacylglycerol lipase ABHD12	ABHD12	0,831808351	416820000
Methylthioribose-1-phosphate isomerase	MRI1	0,831739167	1558300000
Aspartate--tRNA ligase, mitochondrial	DARS2	0,831462543	57300000
LYR motif-containing protein 4	LYRM4	0,831255195	114590000
Peroxisomal membrane protein PMP34	SLC25A17	0,831186103	134930000
Methyltransferase-like protein 7A	METTL7A	0,831047951	107020000
Transmembrane emp24 domain-containing protein 7	TMED7	0,830978893	6467400000
ADP/ATP translocase 3;ADP/ATP translocase 3, N-terminally processed	SLC25A6	0,830633774	24067000000
Treacle protein	TCOF1	0,830495806	321140000
Superoxide dismutase;Superoxide dismutase [Mn], mitochondrial	SOD2	0,830357884	3970300000
Cat eye syndrome critical region protein 5	CECR5	0,829462508	218810000
Nucleoside diphosphate kinase;Nucleoside diphosphate kinase 3	c371H6.2;NME3	0,828706389	469720000
Erlin-1	SPFH1;ERLIN1	0,828706389	450460000
Huntingtin-interacting protein 1	HIP1	0,82863772	475650000
Up-regulated during skeletal muscle growth protein 5	USMG5	0,828088771	499160000
2-aminoethanethiol dioxygenase	ADO	0,826993053	410010000
Vacuolar protein sorting-associated protein 13A	VPS13A	0,826787929	1548200000
Aspartyl/asparaginyl beta-hydroxylase	ASPH	0,826036676	2231900000
Procollagen galactosyltransferase 1	COLGALT1	0,825968448	1716500000
39S ribosomal protein L14, mitochondrial	MRPL14	0,825559316	33444000
Multiple coagulation factor deficiency protein 2	MCFD2	0,825491167	502360000
Aminoacylase-1	ACY1;ABHD14A;ABHD14A-ACY1	0,825014438	748790000
Metaxin-1	MTX1	0,824810294	466440000
Retinoblastoma-binding protein 5	RBBP5	0,824742268	689850000
Unconventional prefoldin RPB5 interactor 1	URI1;C19orf2	0,824674254	20439000
Ubiquitin carboxyl-terminal hydrolase;Ubiquitin carboxyl-terminal hydrolase 8	USP8	0,824538259	306650000
Acylglycerol kinase, mitochondrial	AGK;FLJ10842	0,824470278	782710000
Synaptosomal-associated protein 25	SNAP25	0,824266403	188990000
Smith-Magenis syndrome chromosomal region candidate gene 8 protein	SMCR8	0,823723229	209230000
Negative elongation factor E	NELF-E;NELFE;RDBP	0,823113013	270360000
Isocitrate dehydrogenase [NAD] subunit beta, mitochondrial	IDH3B	0,822909809	11931000000
Succinyl-CoA:3-ketoacid-coenzyme A transferase;Succinyl-CoA:3-ketoacid coenzyme A transferase 1, mitochondrial	OXCT;OXCT1	0,822571358	3411100000
Peroxisomal membrane protein PEX14	PEX14	0,822097994	198990000
Inositol 1,4,5-trisphosphate receptor type 3	ITPR3	0,821895291	1582300000
Mitochondrial inner membrane protein COX18	COX18	0,821692687	44952000
Alpha-aminoadipic semialdehyde dehydrogenase	ALDH7A1	0,821557673	70706000
Stomatin-like protein 2, mitochondrial	STOML2	0,821018062	7636700000
Glucokinase	GCK	0,820815891	2212900000
Proteasome subunit beta type;Proteasome subunit beta type-8	PSMB8	0,820546484	3932000000
Long-chain-fatty-acid--CoA ligase 1	ACSL1	0,8200082	425640000
Ras GTPase-activating-like protein IQGAP2	IQGAP2	0,819873739	2773800000
RAC-beta serine/threonine-protein kinase	AKT2	0,819739323	84513000
3-ketoacyl-CoA thiolase, peroxisomal	ACAA1	0,819739323	582720000
Synaptojanin-2-binding protein	SYNJ2BP	0,818866689	1446800000
39S ribosomal protein L16, mitochondrial	MRPL16	0,818732602	470550000
Small integral membrane protein 4	SMIM4	0,817929004	75477000
Integrin alpha-6;Integrin alpha-6 heavy chain;Integrin alpha-6 light chain;Processed integrin alpha-6	ITGA6	0,817795224	255670000
NADH dehydrogenase [ubiquinone] 1 alpha subcomplex assembly factor 4	NDUFAF4	0,817728351	274910000
2,3-cyclic-nucleotide 3-phosphodiesterase	CNP	0,817661488	42225000
Protein CMSS1	CMSS1;hCG_2023567	0,817460966	91409000
	TRPC3	0,817260543	134640000
Leucine--tRNA ligase, cytoplasmic	LARS	0,817126982	16830000000
Iron-sulfur cluster assembly enzyme ISCU, mitochondrial	ISCU	0,817060217	627490000
Helicase with zinc finger domain 2	PRIC285;HELZ2	0,816993464	1032100000
Cysteine desulfurase, mitochondrial	NFS1	0,81679327	2044000000
Signal-induced proliferation-associated 1-like protein 1	SIPA1L1	0,81672656	83705000
Mitochondrial-processing peptidase subunit alpha	PMPCA	0,816659861	1456600000
Triosephosphate isomerase	TPI1	0,816659861	1,4015E+11
39S ribosomal protein L11, mitochondrial	MRPL11	0,816326531	492630000
Peptidyl-prolyl cis-trans isomerase NIMA-interacting 4	PIN4	0,816259897	80771000
Protein aurora borealis	C13orf34;BORA	0,816193275	230630000
DNA mismatch repair protein Msh3	MSH3	0,815527646	144420000
Mitochondrial pyruvate carrier 2	MPC2	0,815394651	65351000
Telomerase protein component 1	TEP1	0,815195239	543720000
ADP/ATP translocase 1	SLC25A4	0,814929509	5888200000
Dynamin-binding protein	DNMBP	0,814863103	109060000
Electron transfer flavoprotein subunit alpha, mitochondrial	ETFA	0,814398567	25996000000

Pyruvate dehydrogenase E1 component subunit beta, mitochondrial	PDHB	0,814067079	3850300000
Thromboxane-A synthase	TBXAS1;hCG_14925	0,813802083	129120000
Peroxisomal membrane protein 11B	PEX11B	0,813404913	736430000
Thymocyte nuclear protein 1	THYN1	0,813404913	664830000
Ribosyldihydronicotinamide dehydrogenase [quinone]	NQO2	0,812942037	1505600000
Citrate lyase subunit beta-like protein, mitochondrial	CLYBL	0,812743823	1029100000
ATP-binding cassette sub-family D member 1	ABCD1;ALD	0,812545706	19659000
Farnesyl pyrophosphate synthase	FDPS	0,812545706	1905200000
LisH domain and HEAT repeat-containing protein KIAA1468	KIAA1468	0,812545706	396740000
UV radiation resistance-associated gene protein	UVRAG	0,812281699	13956000
SRA stem-loop-interacting RNA-binding protein, mitochondrial	SLIRP	0,81214976	115870000
Calcium and integrin-binding protein 1	CIB1	0,811951932	67802000
NADH dehydrogenase [ubiquinone] iron-sulfur protein 7, mitochondrial	NDUFS7	0,811886011	1002400000
WD repeat-containing protein 81	WDR81	0,811886011	44408000
Mannose-1-phosphate guanyltransferase beta	GMPPB	0,811161583	2214300000
Phosphoglycerate kinase 1;Phosphoglycerate kinase	PGK1	0,810766986	2,1112E+11
Lysophosphatidylcholine acyltransferase 1	AYTL2;LPCAT1	0,810766986	33871000
Cleavage and polyadenylation specificity factor subunit 4	CPSF4	0,810175808	174350000
Receptor expression-enhancing protein 5	REEP5	0,809978941	860240000
Phosphoglucomutase-1	PGM1	0,809716599	3733400000
TBC1 domain family member 10B	TBC1D10B	0,809716599	19952000
Nucleotide exchange factor SIL1	SIL1	0,80925791	130470000
Receptor-type tyrosine-protein phosphatase C	PTPRC	0,809126952	12276000000
Leucine-rich PPR motif-containing protein, mitochondrial	LRPPRC	0,808472795	4479600000
		0,808276754	27153000
Lamin-B receptor	LBR	0,808146113	1072200000
Pyruvate dehydrogenase protein X component, mitochondrial	PDHX	0,80795023	286890000
Protein NipSnap homolog 2	GBAS	0,807363152	1561800000
Beta-galactosidase	GLB1	0,807297974	97317000
tRNA (guanine(26)-N(2))-dimethyltransferase	TRMT1;SEMA4B	0,807167649	177100000
ATP-dependent 6-phosphofructokinase, platelet type	PFKP	0,807167649	8710300000
Hexokinase-3	HK3	0,806711843	22604000000
Uncharacterized protein C2orf47, mitochondrial	C2orf47	0,806126562	565400000
NADH dehydrogenase [ubiquinone] iron-sulfur protein 3, mitochondrial	NDUFS3;DKFZp586K0821	0,80567193	5748800000
Serine/arginine repetitive matrix protein 1	SRRM1	0,80554213	850780000
Glutamine--fructose-6-phosphate aminotransferase [isomerizing] 1	GFPT1	0,805347507	7731200000
Oxysterol-binding protein;Oxysterol-binding protein-related protein 8	DKFZp686C0249;OSBPL8	0,804699445	1358400000
Serine/threonine-protein kinase D;Serine/threonine-protein kinase D2	PRKD2	0,804569957	675320000
Long-chain-fatty-acid--CoA ligase 5	ACSL5	0,804246421	600850000
Signal peptidase complex catalytic subunit SEC11A	SPC18;SEC11A	0,803923145	6054100000
Mannose-1-phosphate guanyltransferase alpha	GMPPA	0,803858521	1006400000
Peroxisomal multifunctional enzyme type 2;(3R)-hydroxyacyl-CoA dehydrogenase;Enoyl-CoA hydratase 2	HSD17B4	0,803406443	1977400000
Presequence protease, mitochondrial	PITRM1	0,803212851	2671900000
NADH-ubiquinone oxidoreductase 75 kDa subunit, mitochondrial	NDUFS1	0,802890405	5208300000
Dynamin-like 120 kDa protein, mitochondrial;Dynamin-like 120 kDa protein, form S1	OPA1	0,80237503	787100000
Glyoxylate reductase/hydroxypyruvate reductase	GRHPR	0,802181935	1043900000
Long-chain-fatty-acid--CoA ligase 3	ACSL3	0,802053256	432680000
NADH dehydrogenase [ubiquinone] iron-sulfur protein 2, mitochondrial	NDUFS2	0,801988933	4783300000
Inosine-5-monophosphate dehydrogenase;Inosine-5-monophosphate dehydrogenase 1	IMPDH;DKFZp781N0678;IMPDH1	0,801667468	788290000
L-2-hydroxyglutarate dehydrogenase, mitochondrial	L2HGDH	0,801474713	329940000
Leucine-rich repeat and calponin homology domain-containing protein 4	LRCH4	0,801089482	38820000
Monofunctional C1-tetrahydrofolate synthase, mitochondrial	MTHFD1L	0,8	14426000000
	IARS	0,79987202	1410400000
Pyruvate dehydrogenase E1 component subunit alpha, somatic form, mitochondrial;Pyruvate dehydrogenase E1 component	PDHA1;PDHA1/LOC79064	0,799680128	12276000000
MICOS complex subunit MIC60	IMMT	0,799360512	6533900000
Bystin	BYSL	0,799232737	850710000
Acyl-CoA dehydrogenase family member 9, mitochondrial	ACAD9	0,798977309	882890000
	CNOT1	0,798977309	68114000
NADPH:adrenodoxin oxidoreductase, mitochondrial	FDXR	0,798275724	515830000
Elongation factor Tu, mitochondrial	TUFM	0,798148296	11370000000
Purine nucleoside phosphorylase	PNP	0,797957229	5347900000
Protein FAM91A1	FAM91A1	0,797829903	93761000
Alpha-galactosidase A	GLA	0,797384579	285220000
Transmembrane protein 11, mitochondrial	TMEM11	0,797130331	1528100000
Protein disulfide-isomerase TMX3	TMX3	0,797130331	4728400000
NADH dehydrogenase [ubiquinone] iron-sulfur protein 8, mitochondrial	NDUFS8	0,797066794	1676600000
Dual specificity mitogen-activated protein kinase kinase 1	MAP2K1	0,797066794	10281000000
Electron transfer flavoprotein subunit beta	ETFB	0,796431985	16906000000
Poly(A)-specific ribonuclease PARN	PARN	0,796431985	388020000
Wiskott-Aldrich syndrome protein family member 1	WASF1	0,795798186	170140000
Mitochondrial import inner membrane translocase subunit TIM44	TIMM44;hTIM44	0,795291872	1343000000
Biogenesis of lysosome-related organelles complex 1 subunit 3	BLOC1S3	0,795165394	59077000
	ACON	0,794975753	7205400000
	MRPL12	0,794659886	1873000000
Growth arrest and DNA damage-inducible proteins-interacting protein 1	GADD45GIP1	0,79358781	25630000000
Actin-related protein 2/3 complex subunit 5;Actin-related protein 2/3 complex subunit 5-like protein	ARPC5L	0,793210121	2585600000
Succinate dehydrogenase [ubiquinone] cytochrome b small subunit, mitochondrial	UNQ5809;SDHD	0,793021412	1479300000
NADH-ubiquinone oxidoreductase chain 5	ND5	0,792832792	178950000
JmjC domain-containing protein 7	JMJD7	0,792581438	147380000
		0,792455821	121380000
CDKN2AIP N-terminal-like protein	CDKN2AIPNL	0,792204706	128410000
Coiled-coil domain-containing protein 88B	CCDC88B	0,792141952	2847200000
Phospholipase DDHD2	DDHD2;TUBD1	0,79195375	114560000
Enoyl-CoA hydratase, mitochondrial	ECHS1	0,791139241	2521700000
Phosphoglycerate kinase		0,790951515	221970000
Cytochrome c-type heme lyase	DKFZp779I1858;HCCS	0,790201501	1050300000
Dephospho-CoA kinase domain-containing protein	DCAKD	0,78976465	613280000
Annexin;Annexin A6	ANXA6	0,789515238	5011500000
Ectopic P granules protein 5 homolog	EPG5	0,789515238	22976000
Son of sevenless homolog 1	SOS1	0,789079145	784450000
Peptidyl-prolyl cis-trans isomerase FKBP1A;Peptidyl-prolyl cis-trans isomerase	FKBP1A	0,787773751	15354000000
28S ribosomal protein S29, mitochondrial	DAP3;MRPS29	0,787401575	557580000
Kynurenine--oxoglutarate transaminase 3	hCG_23341;CCBL2	0,786905886	654780000
	HUWE1	0,786534529	20225000
Nicotinamide phosphoribosyltransferase	NAMPT;NAMPTL	0,785916378	2865000000
Histone H2B type 2-E;Histone H2B type 1-J;Histone H2B type 1-B;Histone H2B type 1-O;Histone H2B type 3-B	HIST2H2BE;HIST1H2BJ;HIST1H2E	0,785422557	47187000
N-acetyl-D-glucosamine kinase	NAGK	0,785175879	272370000
NADPH--cytochrome P450 reductase	DKFZp686G04235;POR	0,784436774	11988000000
Isochorismatase domain-containing protein 2, mitochondrial	ISOC2	0,784375245	253840000
Nucleoside diphosphate kinase;Nucleoside diphosphate kinase, mitochondrial	NME4	0,784190715	89249000
Cyclin-dependent kinase 6	CDK6	0,784129224	7734000000
NADH dehydrogenase [ubiquinone] 1 beta subcomplex subunit 8, mitochondrial	NDUFB8	0,784006272	447840000
Short-chain specific acyl-CoA dehydrogenase, mitochondrial	ACADS	0,78369906	2958500000
Transcription initiation factor TFIID subunit 5	TAF5	0,78235018	86260000
Dihydropyrimidinase-related protein 2	DPYSL2	0,780640125	3967400000
Acyl-coenzyme A oxidase;Peroxisomal acyl-coenzyme A oxidase 1	ACOX1	0,779970361	199290000
Triggering receptor expressed on myeloid cells 2	TREM2	0,779970361	1320400000
39S ribosomal protein L41, mitochondrial	MRPL41	0,779362481	165580000

Prolactin regulatory element-binding protein	PREB	0,778937529	505030000
55 kDa erythrocyte membrane protein	MPP1	0,778755549	604220000
Acyl carrier protein, mitochondrial;Acyl carrier protein	NDUFAB1	0,777725929	227230000
Cytochrome c	CYCS	0,776940409	8482300000
Retinoid-inducible serine carboxypeptidase	SCPEP1	0,77579519	480270000
NF-X1-type zinc finger protein NFXL1	URCC5;NFXL1	0,775434243	56244000
Decaprenyl-diphosphate synthase subunit 2	PDSS2	0,774293457	19176000
Mitochondrial import inner membrane translocase subunit TIM14	DNAJC19	0,773754256	200800000
DENN domain-containing protein 2D	DENND2D	0,772678102	30387000
Lanosterol synthase	LSS	0,772379702	78659000
Aldehyde dehydrogenase, mitochondrial	ALDH2	0,772320049	11239000000
Coronin-1A;Coronin	CORO1A	0,772081532	30336000000
NADH dehydrogenase [ubiquinone] 1 alpha subcomplex subunit 7	NDUFA7	0,771128933	250220000
Acetyl-CoA acetyltransferase, mitochondrial	ACAT1	0,771069473	2481700000
Serine hydroxymethyltransferase;Serine hydroxymethyltransferase, mitochondrial	SHMT2;DKFZp686P09201	0,770297335	44889000000
Helicase ARIP4	RAD54L2	0,768462307	40850000
Core-binding factor subunit beta	CBFB	0,76810815	331070000
ATPase family AAA domain-containing protein 3A;ATPase family AAA domain-containing protein 3B	ATAD3A;ATAD3B	0,76799017	1824700000
Serine palmitoyltransferase 1	SPTLC1	0,767931193	169610000
39S ribosomal protein L15, mitochondrial	MRPL15	0,767106474	579500000
RAS guanyl-releasing protein 2	RASGRP2	0,766812361	35140000
Signal transducer and activator of transcription 1-alpha/beta;Signal transducer and activator of transcription	STAT1	0,766812361	2711400000
Coiled-coil domain-containing protein 58	CCDC58	0,766577233	2920900000
Mitochondrial Rho GTPase 2	RHOT2	0,76581406	221220000
39S ribosomal protein L20, mitochondrial	MRPL20	0,765755418	170490000
L-lactate dehydrogenase;L-lactate dehydrogenase A chain	LDHA	0,765345171	1,1128E+11
Acetyltransferase component of pyruvate dehydrogenase complex;Dihydrolipoyllysine-residue acetyltransferase componen	DLAT	0,765169485	2123700000
E3 ubiquitin-protein ligase MYCBP2	MYCBP2	0,765110941	1544300000
Peroxiredoxin-5, mitochondrial	PRDX5	0,764117063	12444000000
ATP-dependent Clp protease proteolytic subunit, mitochondrial;ATP-dependent Clp protease proteolytic subunit	CLPP	0,763358779	2396400000
DnaJ homolog subfamily C member 10	DNAJC10	0,762660159	664570000
Histone deacetylase 10	HDAC10	0,761730652	68456000
Hexokinase-1	HK1	0,760340633	7009300000
Methylglutaconyl-CoA hydratase, mitochondrial	AUH	0,759705234	370270000
Sodium bicarbonate cotransporter 3	SLC4A7	0,7583801	386190000
Aconitate hydratase, mitochondrial	ACO2	0,758150114	28923000000
Methylosome protein 50	WDR77	0,758035173	750980000
Poly(rC)-binding protein 4	PCBP4	0,757346259	44920000
Short/branched chain specific acyl-CoA dehydrogenase, mitochondrial	ACADSB	0,75694497	1204200000
Plastin-2	LCP1	0,755629439	75843000000
BCL2/adenovirus E1B 19 kDa protein-interacting protein 3-like	BNIP3L	0,755458185	100300000
Aldo-keto reductase family 1 member C2	AKR1C2	0,753352418	2874100000
2,4-dienoyl-CoA reductase, mitochondrial	DECR1;DECR	0,753068755	728250000
NADH dehydrogenase [ubiquinone] 1 alpha subcomplex subunit 9, mitochondrial	NDUFA9	0,752445448	1386400000
Mitochondrial fission regulator 1-like	MTFR1L	0,752162467	75565000
Adenylate kinase isoenzyme 1	AK1	0,752105897	1271500000
cAMP-dependent protein kinase type I-alpha regulatory subunit;cAMP-dependent protein kinase type I-alpha regulatory subu	DKFZp779L0468;PRKAR1A	0,751653638	6405800000
GRB2-associated-binding protein 2	GAB2	0,751653638	25953000
		0,751258358	107030000
ATP-dependent 6-phosphofructokinase, muscle type	PFKM	0,748559024	7995400000
Caspase-8;Caspase-8 subunit p18;Caspase-8 subunit p10	CASP8	0,747831289	52540000
Pyrroline-5-carboxylate reductase 2;Pyrroline-5-carboxylate reductase	PYCR2;P5CR2;DKFZp434J218	0,746770219	2875500000
Cob(I)yrinic acid a,c-diamide adenosyltransferase, mitochondrial	MMAB	0,744878957	125630000
1,2-dihydroxy-3-keto-5-methylthiopentene dioxygenase	ADI1	0,744878957	474190000
Acid ceramidase;Acid ceramidase subunit alpha;Acid ceramidase subunit beta	ASAH1	0,744657085	231980000
Nucleolar protein 6	NOL6	0,744213738	52482000
Transmembrane protein 160	TMEM160	0,743660296	198890000
Bis(5-adenosyl)-triphosphatase	FHIT	0,743273376	311530000
Coronin-7;Coronin	CORO7;hCG_1787779	0,743107676	2074600000
Tricarboxylate transport protein, mitochondrial	SLC25A1	0,74261102	7809600000
Chloride intracellular channel protein 5;Chloride intracellular channel protein 6	CLIC5;CLIC6	0,742004897	40985000
Signal-induced proliferation-associated protein 1	SIPA1	0,741729714	703880000
Protein FAM65A	FAM65A	0,741564702	118210000
Protein S100-A4	S100A4	0,741234897	1150100000
Endoplasmic reticulum-Golgi intermediate compartment protein 1	ERGIC1	0,738497895	4956500000
39S ribosomal protein L21, mitochondrial	MRPL21	0,73806185	445440000
AFG3-like protein 2	AFG3L2	0,737735153	1526300000
3-hydroxyisobutyrate dehydrogenase, mitochondrial	HIBADH	0,736756797	1954200000
Inositol monophosphatase 3	IMPAD1	0,735510444	23837000
Microtubule-associated protein 1S;MAP1S heavy chain;MAP1S light chain	MAP1S	0,735240056	139320000
Ras-related protein R-Ras	RRAS	0,735023888	184030000
Phosphoglycerate mutase;Phosphoglycerate mutase 1	PGAM1	0,735023888	1,5868E+11
von Willebrand factor A domain-containing protein 8	VWA8	0,734537976	170030000
Pyrroline-5-carboxylate reductase;Pyrroline-5-carboxylate reductase 1, mitochondrial	PYCR1;PIG45	0,733514267	4714700000
Rho GTPase-activating protein 4	ARHGAP4;C1	0,732600733	306390000
Beta-enolase;Enolase	ENO3	0,732118017	37260000000
Ras-related protein Rab-3A	RAB3A	0,731582413	79791000
TOM1-like protein 1	TOM1L1	0,729660708	28210000
ATP-dependent 6-phosphofructokinase, liver type	PFKL	0,728438228	4025700000
Inositol 1,4,5-trisphosphate receptor type 2	ITPR2	0,72663857	1052100000
Zinc finger protein-like 1	ZFPL1	0,726480203	94178000
Phosphorylase b kinase regulatory subunit alpha, liver isoform	PHKA2	0,725320955	34046000
Carbonic anhydrase 13	CA13	0,725215752	204840000
Clathrin heavy chain 2	CLTCL1	0,724847782	1940000000
Methylcrotonoyl-CoA carboxylase beta chain, mitochondrial	MCCC2	0,724112962	91198000
FCH domain only protein 1	FCHO1	0,722752241	55731000
Protein SZT2	SZT2	0,721396624	32800000
Very-long-chain enoyl-CoA reductase	TECR	0,720928556	4086200000
DNA polymerase delta subunit 3	POLD3	0,720253529	529920000
28S ribosomal protein S16, mitochondrial	MRPS16	0,719579765	507060000
Serine/threonine-protein kinase/endoribonuclease IRE1;Serine/threonine-protein kinase;Endoribonuclease	ERN1	0,717978173	104630000
Ras-related protein Rab-24	RAB24	0,71751453	1004000000
Protein UXT	UXT	0,717102904	154640000
Mitochondrial import inner membrane translocase subunit Tim8 B	TIMM8B	0,71653769	147830000
39S ribosomal protein L23, mitochondrial	MRPL23	0,716281069	257280000
Protein canopy homolog 4	CNPY4	0,715461115	641100000
Valine--tRNA ligase, mitochondrial	VARS2	0,714234698	46749000
39S ribosomal protein L28, mitochondrial	MRPL28	0,713623064	260930000
Nephrocystin-4	NPHP4	0,713623064	76097000
LETM1 domain-containing protein 1	LETMD1	0,71347032	317550000
Succinyl-CoA ligase [GDP-forming] subunit beta, mitochondrial	SUCLG2	0,71331764	4740000000
Septin-9	09-Sep	0,712961643	12487000000
Putative RNA-binding protein 15	RBM15	0,712707576	8427100
Hexokinase-2	HK2;DKFZp686M1669	0,712301446	12048000000
Ribosome production factor 2 homolog	RPF2	0,710883628	102550000
28S ribosomal protein S12, mitochondrial	MRPS12	0,709622481	185800000
E3 ubiquitin-protein ligase RBBP6	RBBP6	0,708315625	39818000
Mitochondrial chaperone BCS1	BCS1L	0,707163567	256350000

Glycerol-3-phosphate dehydrogenase [NAD(+)], cytoplasmic	GPD1	0,707063565	37393000
Glyceraldehyde-3-phosphate dehydrogenase, testis-specific	GAPDHS	0,706963591	755610000
Fatty acid desaturase 1	FADS1	0,704771302	60329000
DmX-like protein 1	DMXL1	0,703828829	55370000
Mitochondrial import receptor subunit TOM40B	TOMM40L	0,703531729	413490000
Fumarylacetoacetate hydrolase domain-containing protein 2A;Fumarylacetoacetate hydrolase domain-containing protein 2B	FAHD2A;FAHD2B	0,701114772	49765000
Regulator of microtubule dynamics protein 1	RMDN1	0,701016474	110690000
ALS2 C-terminal-like protein	FLJ00189;ALS2CL	0,697885407	42407000
Nucleoredoxin	NXN	0,697398703	38335000
PRA1 family protein 2	PRAF2	0,697204211	190650000
O-acetyl-ADP-ribose deacetylase MACROD1	MACROD1	0,697107006	206580000
Dehydrogenase/reductase SDR family member 7B	DHRS7B	0,696621386	243530000
Iron-sulfur cluster co-chaperone protein HscB, mitochondrial	HSCB	0,695894224	178730000
Protein NipSnap homolog 1	NIPSNAP1	0,694396222	675120000
General transcription factor 3C polypeptide 2	GTF3C2	0,694010688	16916000
NADH dehydrogenase [ubiquinone] 1 alpha subcomplex subunit 11	NDUFA11	0,692568737	49773000
Phosphatidylinositol 3,4,5-trisphosphate 5-phosphatase 1	INPP5D	0,69151511	996770000
NADH dehydrogenase [ubiquinone] 1 alpha subcomplex assembly factor 3	DKFZp564J0123;C3orf60;NDUFAF	0,689132382	31266000
E3 ubiquitin-protein ligase HACE1	HACE1	0,688989941	27367000
28S ribosomal protein S10, mitochondrial	MRPS10	0,688847558	256250000
Neuroguidin	NGDN	0,688657806	151950000
39S ribosomal protein L49, mitochondrial	MRPL49	0,687190764	164680000
Proteasome subunit beta type;Proteasome subunit beta type-9	PSMB9	0,686766019	1280900000
39S ribosomal protein L2, mitochondrial	MRPL2	0,686624554	241030000
28S ribosomal protein S28, mitochondrial	MRPS28	0,686436024	147300000
Phosphatidylinositol 4,5-bisphosphate 3-kinase catalytic subunit gamma isoform	PIK3CG	0,681431005	1366200000
Apoptosis regulator Bcl-2	bcl-2;BCL2	0,681338148	1000200000
Isocitrate dehydrogenase [NADP], mitochondrial;Isocitrate dehydrogenase [NADP]	IDH2	0,67999456	17021000000
HLA class I histocompatibility antigen, alpha chain E;HLA class I histocompatibility antigen, B-38 alpha chain;HLA class I histo	HLA-B;HLA-C;HLA-A;HLA-E;HLA-C	0,674763833	2437300000
Alpha-ketoglutarate-dependent dioxygenase alkB homolog 3	ALKBH3	0,670106547	277850000
Diablo homolog, mitochondrial	DIABLO	0,66983723	344110000
RNA demethylase ALKBH5	ALKBH5	0,669747505	188290000
Propionyl-CoA carboxylase beta chain, mitochondrial	PCCB;DKFZp451E113	0,668583272	1040500000
Nucleoside diphosphate kinase;Nucleoside diphosphate kinase 6	NME6	0,664672649	20660000
28S ribosomal protein S34, mitochondrial	MRPS34	0,660283922	1246200000
28S ribosomal protein S9, mitochondrial	MRPS9	0,659804698	846440000
28S ribosomal protein S22, mitochondrial	MRPS22	0,65802461	313090000
28S ribosomal protein S25, mitochondrial	MRPS25	0,657764915	491180000
Adseverin	SCIN	0,656340247	335230000
[Pyruvate dehydrogenase (acetyl-transferring)] kinase isozyme 3, mitochondrial	PDK3	0,653125204	1820400000
Tyrosine-protein phosphatase non-receptor type 9	PTPN9	0,651635605	320500000
Receptor-type tyrosine-protein phosphatase;Receptor-type tyrosine-protein phosphatase epsilon	PTPRE	0,648340249	499240000
28S ribosomal protein S35, mitochondrial	MRPS35	0,647123536	268080000
Protein FAM162A	FAM162A	0,646746863	440020000
Cyclin-dependent kinase 4 inhibitor C	CDKN2C	0,646579594	333640000
Transient receptor potential cation channel subfamily V member 2	TRPV2	0,645411127	124350000
Antigen peptide transporter 1	TAP1	0,64441294	25279000
Alanine aminotransferase 2	GPT2	0,643666323	670080000
Advillin	AVIL	0,640040963	5419700000
Protein diaphanous homolog 2	DIAPH2	0,636496722	1179100000
Coiled-coil-helix-coiled-coil-helix domain-containing protein 7	CHCHD7	0,636415707	24394000
ATP synthase subunit delta, mitochondrial	ATP5D	0,636213259	4094500000
Ral GTPase-activating protein subunit alpha-2	RALGAPA2;hCG_22457	0,635404753	245420000
Adenylate kinase 4, mitochondrial	AK4	0,632471064	835550000
Pyruvate carboxylase, mitochondrial	PC	0,630954634	6191800000
Proline dehydrogenase 1, mitochondrial	PRODH	0,628930818	428760000
Threonine--tRNA ligase, mitochondrial	TARS2	0,627706986	357460000
DNA repair protein XRCC1	XRCC1;FOXH1	0,627352572	166690000
Protein enabled homolog	ENAH	0,627234523	963040000
Ribonucleoside-diphosphate reductase subunit M2;Ribonucleoside-diphosphate reductase subunit M2 B	RRM2;hCG_23833;DKFZp686M052	0,620116582	106560000
Probable inactive tRNA-specific adenosine deaminase-like protein 3	hCG_22695;ADAT3	0,617932398	35527000
	SMCR7L	0,611620795	30689000
39S ribosomal protein L24, mitochondrial	MRPL24	0,607718019	444170000
Isobutyryl-CoA dehydrogenase, mitochondrial	ACAD8	0,606980273	203970000
Propionyl-CoA carboxylase alpha chain, mitochondrial	PCCA	0,602518527	1770000000
Phosphoenolpyruvate carboxykinase [GTP], mitochondrial	PCK2	0,598515681	4855800000
Phosphotriesterase-related protein	PTER	0,59477785	298090000
Mitogen-activated protein kinase kinase kinase 2;Mitogen-activated protein kinase kinase kinase 3	MAP3K2;MAP3K3	0,592592593	53431000
ATP synthase mitochondrial F1 complex assembly factor 2	ATPAF2	0,588685465	475830000
Ribosome biogenesis protein BMS1 homolog	BMS1	0,58844298	17105000
E3 ubiquitin-protein ligase RNF31	RNF31	0,584692744	51772000
Proteasome subunit beta type;Proteasome subunit beta type-10	PSMB10	0,583226408	113280000
MDS1 and EVI1 complex locus protein EVI1;PR domain zinc finger protein 16	EVI1;MECOM;PRDM16	0,58281851	113350000
Probable ATP-dependent RNA helicase DDX58	DDX58	0,581598232	718610000
28S ribosomal protein S23, mitochondrial	MRPS23	0,581462961	280360000
Glucosylceramidase	GBA	0,577433884	27647000
Interferon-induced transmembrane protein 1;Interferon-induced transmembrane protein 2;Interferon-induced transmembrane	IFITM2;IFITM3;IFITM1	0,576934172	5095700000
Acetyl-coenzyme A transporter 1	SLC33A1	0,574844792	198460000
HIG1 domain family member 1A, mitochondrial;HIG1 domain family member 1C	HIGD1A;HIGD1C	0,572672088	15530000
Epimerase family protein SDR39U1	SDR39U1	0,571102227	49903000
TATA element modulatory factor	TMF1	0,568698817	159460000
Lon protease homolog, mitochondrial;Lon protease homolog	LONP1	0,567923671	849180000
Lon protease homolog, mitochondrial	LONP1	0,564748405	4207900000
Putative tyrosine-protein phosphatase auxilin	DNAJC6	0,558378469	428430000
Methionine synthase	MTR;DKFZp686A22123	0,556637907	21504000
Protein tweety homolog 3	TTYH3	0,555740802	14713000
Glycogenin-1	GYG1	0,547705116	72147000
Glycogenin-2	GYG2	0,543183053	159550000
[Pyruvate dehydrogenase (acetyl-transferring)] kinase isozyme 1, mitochondrial	PDK1	0,537056928	35095000
Leukocyte receptor cluster member 8	LENG8	0,531914894	61923000
Phosphatidylinositol 3,4,5-trisphosphate 3-phosphatase and dual-specificity protein phosphatase PTEN	PTEN;PTH2	0,531180283	113490000
Glutamine synthetase	PIG59;GLUL	0,526011257	1316100000
Tyrosine-protein kinase receptor;Insulin-like growth factor 1 receptor;Insulin-like growth factor 1 receptor alpha chain;Insulin	IGF1R	0,517491203	170180000
DNA-directed RNA polymerase		0,515012618	33765000
Dual specificity tyrosine-phosphorylation-regulated kinase 1A;Dual specificity tyrosine-phosphorylation-regulated kinase 1B	DYRK1A;DYRK1B	0,514827018	116670000
Protein-glutamine gamma-glutamyltransferase E;Protein-glutamine gamma-glutamyltransferase E 50 kDa catalytic chain;Pro	TGM3	0,511796919	66407000
Prolyl 4-hydroxylase subunit alpha-1	P4HA1	0,511378164	17083000
Estradiol 17-beta-dehydrogenase 8	HSD17B8	0,509580106	177320000
Probable phospholipid-transporting ATPase IF	ATP11B	0,492829333	29112000
Protein phosphatase 1B	PPM1B	0,489141068	63300000
Neuroplastin	DKFZp566H1924;NPTN	0,483185157	250410000
Pescadillo homolog	PES1	0,479800403	177620000
Napsin-A	NAPSA	0,467420772	1188000000
2-5-oligoadenylate synthase 3	OAS3	0,453967678	169650000
Ig alpha-1 chain C region;Ig alpha-2 chain C region	SNC73;DKFZp686G21220;DKFZp6	0,44780798	95591000
Protein RIC1 homolog	KIAA1432	0,428210508	14087000
	ZNF551	0,426730392	41329000
Protein SMG5	SMG5	0,42073376	10476000

Eukaryotic translation initiation factor 2-alpha kinase 4	EIF2AK4	0,386847195	22039000
Tetraspanin-14	TSPAN14	0,354459095	214980000
Mitochondrial pyruvate carrier 1	BRP44L;MPC1	0,35180299	759610000
Actin-related protein 3B;Actin-related protein 3C	ACTR3B;ACTR3C	0,31201248	366210000
	SLC12A1	0,274642278	40646000
Trimethyllysine dioxygenase, mitochondrial	TMLHE	0,27000027	54399000
Protein piccolo	LOC392742;PCLO	0,121756706	480070000
Neutrophil defensin 3;HP 3-56;Neutrophil defensin 2;Neutrophil defensin 1;HP 1-56;Neutrophil defensin 2	DEFA3;DEFA1		20071000
Ig kappa chain C region	IGK@;IGKC		7789700
Cystatin-A;Cystatin-A, N-terminally processed	CSTA		129420000
Prolactin-inducible protein	PIP		150950000
Lipocalin-1;Putative lipocalin 1-like protein 1	LCN1;LCN1P1		71785000
Galectin-7	LGALS7		9488500
FYVE, RhoGEF and PH domain-containing protein 2	FGD2		15323000
Histone deacetylase 6	DKFZp566E044;HDAC6		42810000
Myelin-associated neurite-outgrowth inhibitor	FAM168B		12583000
Breast cancer type 2 susceptibility protein	BRCA2		0
GRIP and coiled-coil domain-containing protein 1	GCC1		5840700
40S ribosomal protein S27	LOC392748		112600000
N-alpha-acetyltransferase 16, NatA auxiliary subunit	NARG1L;NAA16		13354000
CCR4-NOT transcription complex subunit 1	CNOT1		18959000
Intraflagellar transport protein 25 homolog	HSPB11		10087000
Alpha-endosulfine	ENSA		10023000
	PPP2R4		0
Calcium-transporting ATPase	ATP2B4		12580000
Abhydrolase domain-containing protein 16A	BAT5;ABHD16A		5113200
Receptor-type tyrosine-protein phosphatase O	PTPRO		26926000
Probable G-protein coupled receptor 63	GPR63		9607900
WW domain-containing oxidoreductase	WWOX		18773000
			8731300
Protein FAN	NSMAF		29190000
Leucine-rich repeat flightless-interacting protein 2	LRRFIP2		14003000
Alcohol dehydrogenase 1B;Alcohol dehydrogenase 1A;Alcohol dehydrogenase 1C	ADH1B;ADH1A;ADH1C		49635000
tRNA-splicing endonuclease subunit Sen15	C1orf19;TSEN15		32063000
Cell division cycle-associated protein 3	CDCA3		0
			0
Protein kinase C eta type;Protein kinase C epsilon type	PRKCH;PRKCE		30338000
Keratin, type I cytoskeletal 18	KRT18		9418300
			9887800
Spectrin beta chain, erythrocytic	SPTB		32064000
Protein FAM133B	FAM133B;MGC40405		30970000
Uncharacterized protein KIAA2013	LOC728138;KIAA2013		11857000
			8095800
F-box only protein 4	FBXO4		17798000
G1/S-specific cyclin-D3	CCND3		17856000
Low density lipoprotein receptor adapter protein 1	LDLRAP1		33013000
Dedicator of cytokinesis protein 7	DOCK7		0
Volume-regulated anion channel subunit LRRC8D	DKFZp666L2010;DKFZp547I0910;LRRC8D		3051000
Succinyl-CoA:3-ketoacid-coenzyme A transferase;Succinyl-CoA:3-ketoacid coenzyme A transferase 2, mitochondrial	OXCT2		32532000
Branched-chain-amino-acid aminotransferase;Branched-chain-amino-acid aminotransferase, mitochondrial	BCAT2		4333900
	RGS20		24488000
MAP kinase-interacting serine/threonine-protein kinase 1	MKNK1;DKFZp686E14208		0
			41992000
Leucine-rich repeat-containing protein 15	LRRC15		119860000
Dedicator of cytokinesis protein 9	DOCK9;DKFZp686N04132		60947000
Serine/threonine-protein kinase 3;Serine/threonine-protein kinase 3 36kDa subunit;Serine/threonine-protein kinase 3 20kDa s	STK3		0
			1992400
			18351000
GRAM domain-containing protein 1C	GRAMD1C		0
WD repeat and SOCS box-containing protein 1	WSB1		10132000
			0
Methylcrotonoyl-CoA carboxylase subunit alpha, mitochondrial	MCCC1;DKFZp686B20267		7712000
Hypoxia-inducible factor 1-alpha inhibitor	HIF1AN		20960000
			17126000
Protein phosphatase Slingshot homolog 2	SSH2		9609800
Oxysterol-binding protein;Oxysterol-binding protein 2	OSBP2		16321000
ATP synthase subunit gamma;ATP synthase subunit gamma, mitochondrial	ATP5C1		0
Beta-1,4-galactosyltransferase 1;Lactose synthase A protein;N-acetyllactosamine synthase;Beta-N-acetylglucosaminylglyc	B4GALT1		6200400
Semaphorin-3E	SEMA3E		3140400
			84362000
Ribonucleoside-diphosphate reductase;Ribonucleoside-diphosphate reductase large subunit	RRM1		9775800
			8844200
			12949000
Collagen alpha-1(VI) chain	COL6A1		0
			4972800
Glyceraldehyde-3-phosphate dehydrogenase			0
			73820000
Myocilin;Myocilin, N-terminal fragment;Myocilin, C-terminal fragment	MYOC		64972000
			48921000
Erythrocyte band 7 integral membrane protein	STOM		19534000
Myosin light chain 6B	MYL6B		8243800
Zinc finger protein 346	ZNF346		17980000
			240690000
Exostosin-2	EXT2		4846900
			5412200000
Tryptophan--tRNA ligase, mitochondrial	WARS2		0
Tropomyosin alpha-1 chain	TPM1		31148000
Putative uncharacterized protein C10orf128	hCG_1653562;C10orf128		13492000
Collagen alpha-3(VI) chain	COL6A3;DKFZp686K04147		11303000
Gamma-glutamylcyclotransferase	GGCT		2382900
Apolipoprotein B-100;Apolipoprotein B-48	APOB		20912000
Anaphase-promoting complex subunit 16	C10orf104;ANAPC16		11604000
O-acetyl-ADP-ribose deacetylase 1	OARD1		14338000
Serine/Arginine-related protein 53	RSRC1		64947000
	USP4		14518000
LIM domain-containing protein 1	LIMD1		2780400
Spectrin beta chain, non-erythrocytic 4	SPTBN4;SPNB4		406280000
Zinc finger protein 780B	ZNF780B		0
	AP2M1		0
			9070400
Ephrin type-B receptor 3	EPHB3		58195000
Dihydropyrimidinase-related protein 4	DPYSL4		60935000
GPI ethanolamine phosphate transferase 2	PIGG		6276400
DNA polymerase kappa	POLK		36794000
Putative sodium-coupled neutral amino acid transporter 9	SLC38A9		5389200
Probable ATP-dependent RNA helicase DDX4	DDX4		89423000
Leucine zipper protein 1	LUZP1		24174000
	ENO3		0

	DIAPH1	33345000
Alpha-1-antitrypsin;Short peptide from AAT	SERPINA1	2213300
Cyclic nucleotide-gated cation channel alpha-3	CNGA3	13476000
F-box only protein 3	FBXO3	17829000
	RPS3	0
Choline kinase alpha	CHKA	13672000
Leucine-rich repeat-containing protein 14	LRRC14	4602400
Epiplakin	EPPK1	231630000
Calcium uptake protein 1, mitochondrial	MICU1	24280000
Podocalyxin	PODXL	24077000
Protein C10	C12orf57	10609000
RIMS-binding protein 2	RIMBP2	22521000
Syntaxin-10	STX10	0
	BAG6	72073000
	NAP1L1	71301000
Iron-sulfur protein NUBPL	NUBPL	18528000
	CFAP44;DKFZp434A2017	4878300
AT-rich interactive domain-containing protein 2	ARID2	77418000
NF-kappa-B inhibitor alpha	IkBa;NFKBIA	36378000
Ubiquitin-conjugating enzyme E2 variant 1	UBE2V1;UBE2V2	0
	SNW1	7712000
Tyrosine-protein kinase receptor	PPFIBP1;PPFIBP1-ALK	9311300
NFX1-type zinc finger-containing protein 1	ZNFX1	12801000
	DDX39B	0
Glycine N-acyltransferase	GLYAT	54285000
Ras-related protein Rab-40C	RAB40C	19033000
Mediator of RNA polymerase II transcription subunit 31	MED31	10474000
Pro-interleukin-16;Interleukin-16	IL16	29905000
Tyrosine-protein kinase receptor	TPM3;TPM3-ROS1	0
28S ribosomal protein S7, mitochondrial	MRPS7	20353000
Protein FAM210A	FAM210A	12598000
Transcription elongation factor 1 homolog	ELOF1	36266000
	ATP5A1	27687000
NADH-ubiquinone oxidoreductase chain 1	NADH1;ND1	10288000
	EIF1AY	46077000
	KHDRBS3	37270000
DNA-directed RNA polymerase;DNA-directed RNA polymerase, mitochondrial	POLRMT	2502300
Histone-lysine N-methyltransferase 2D	AAD10;KMT2D	24242000
High affinity copper uptake protein 1	SLC31A1	0
Kinesin heavy chain isoform 5C	KIF5C	15757000
Microtubule-associated serine/threonine-protein kinase 3	MAST3	37167000
Cullin-associated NEDD8-dissociated protein 2	CAND2	79922000
Serine/threonine-protein kinase 17B	STK17B	29784000
Dual specificity protein phosphatase 14	DUSP14	21214000
Antithrombin-III	SERPINC1	1985600
GTPase HRas;GTPase HRas, N-terminally processed	HRAS;c-bas/has	21312000
Polymeric immunoglobulin receptor;Secretory component	PIGR	18042000
Protein kinase C beta type	PRKCB	31185000
Phosphoglycerate kinase 2	PGK2	104790000
Coiled-coil domain-containing protein 175	CCDC175	0
POTE ankyrin domain family member J	POTEJ	17337000
Aldo-keto reductase family 1 member C4	AKR1C4	0
Azurocidin	AZU1	1713100
Serpin B3;Serpin B4	SERPINB3;SCCA2;SERPINB4	4935700
Centrin-2	CETN2	3139800
Histidine ammonia-lyase	HAL	25076000
Casein kinase I isoform epsilon	CSNK1E	14709000
39S ribosomal protein L12, mitochondrial	MRPL12;MRPL7/L12	0
Cyclin-dependent kinases regulatory subunit;Cyclin-dependent kinases regulatory subunit 1	CKS1B	11698000
Actin, alpha skeletal muscle	ACTA1	86511000
Adenylyl cyclase-associated protein 1	CAP1	9749900
Mitogen-activated protein kinase kinase kinase kinase 2	MAP4K2	4089400
Tyrosine-protein phosphatase non-receptor type 13	DKFZp686J1497;PTPN13	0
Glutamate receptor ionotropic, NMDA 2B	GRIN2B	0
Eukaryotic translation initiation factor 4E-binding protein 1	EIF4EBP1	23322000
Sodium/potassium-transporting ATPase subunit alpha-4	ATP1A4	120260000
Tubulin alpha-3C/D chain;Tubulin alpha-3E chain	TUBA2;TUBA3C;TUBA3E	6748400
Plakophilin-1	PKP1	57284000
Tubulin beta-2A chain	TUBB2A	39544000
Plectin	PLEC	111130000
		0
Keratin-associated protein 11-1	KRTAP11-1	65981000
Serpin B12	SERPINB12	37279000
Magnesium transporter NIPA1	NIPA1	10961000
KH homology domain-containing protein 1	KHDC1	18477000
Keratin-associated protein 13-1;Keratin-associated protein 13-2	KRTAP13-1;KRTAP13-2	44827000
Elongation factor 1-alpha		0
	HSP90AB1	11222000
	EIF3I	0
Beta/gamma crystallin domain-containing protein 3	CRYBG3	0
AMMECR1-like protein	AMMECR1L	11379000
	GH1	27706000
		0
		0
		39847000
F-box only protein 50	NCCRP1	86685000
		208170000
Probable proline--tRNA ligase, mitochondrial	PARS2	15544000
MOB kinase activator 1B	MOB1B;MOBKL1A;MOB4A	35793000
Desmoglein-4	DSG4	40351000
Valacyclovir hydrolase	BPHL	0
Xyloside xylosyltransferase 1	XXYLT1	17438000
Transmembrane protein 163	TMEM163	0
Probable tRNA pseudouridine synthase 1	TRUB1	24880000
Mitochondrial import receptor subunit TOM6 homolog	TOMM6	0
Guanine nucleotide-binding protein G(i) subunit alpha-2	GNAI2	11013000
HAUS augmin-like complex subunit 1	HAUS1	0
Protein S100-A16	S100A16	7504000
Sideroflexin-2	SFXN2	17592000
Spermatid perinuclear RNA-binding protein	STRBP	9320300
RNA-binding protein 4B;RNA-binding protein 4	RBM4B;RBM4	0
N-alpha-acetyltransferase 11	NAA11	6749500
Egl nine homolog 1	EGLN1	18171000
Cytosolic 5-nucleotidase 3A	NT5C3A	33243000
Vertnin	VRTN	36567000
Protein S100-A14	S100A14	5160600
Acyl-coenzyme A thioesterase 13;Acyl-coenzyme A thioesterase 13, N-terminally processed	ACOT13	24686000
NAD-dependent protein deacylase sirtuin-5, mitochondrial	SIRT5	49750000

Hexaprenyldihydroxybenzoate methyltransferase, mitochondrial	COQ3	0
EH domain-containing protein 3	EHD3	66631000
E3 ubiquitin-protein ligase RNF181	RNF181	0
ATP-binding cassette sub-family D member 2	ABCD2	70714000
		12227000
